# Who Benefits from National Estuaries? Applying the FEGS Classification System to Identify Ecosystem Services and their Beneficiaries

**DOI:** 10.3390/ijerph16132351

**Published:** 2019-07-03

**Authors:** Susan Harrell Yee, Angelica Sullivan, Kathleen C. Williams, Kirsten Winters

**Affiliations:** 1 United States Environmental Protection Agency, National Health and Environmental Effects Research Laboratory, Gulf Ecology Division, Gulf Breeze, FL 32561, USA; 2 United States Environmental Protection Agency, National Health and Environmental Effects Research Laboratory, Mid-Continent Ecology Division, Duluth, MN 55804, USA; 3 Oak Ridge Institute for Science and Education, United States Environmental Protection Agency, National Health and Environmental Effects Research Laboratory, Western Ecology Division, Corvallis, OR 97333, USA; 4 Department of Electrical Engineering and Computer Science, Oregon State University, Corvallis, OR 97331, USA

**Keywords:** estuary management, final ecosystem goods and services, document analysis, beneficiaries

## Abstract

In spite of their perceived value, the widespread implementation of ecosystem services assessments has been limited because of perceptions of being too technical, too expensive, or requiring special expertise. For example, federal estuary management programs have widely used ecosystem services concepts to frame management issues and communicate with stakeholders. Yet, indicators assessed, monitored, and reported in estuarine management still have traditionally focused on ecological conditions, with weak connections, if any, to social or economic outcomes. Approaches are needed which expand the range of ecosystem services that can be considered, link ecosystem services explicitly to different stakeholder groups, facilitate effective communication with economists and other social scientists, and expand the array of available valuation techniques. We applied the concept of final ecosystem goods and services to review the broad suite of ecosystem services and their beneficiaries relevant to the management of two federal programs for estuary management, the National Estuary Program (NEP) and the National Estuarine Research Reserve System (NERRS). The Final Ecosystem Goods and Services Classification System provided a structured framework for connecting ecosystem services to their beneficiaries and the environments providing them. Document analysis of management plans assessed the degree to which these programs consider ecosystem services, their beneficiaries, and habitats within the estuarine watershed. The hierarchical list of final ecosystem goods and services generated from document analysis serves as a tool for defining management goals, identifying stakeholders, developing meaningful indicators, and conducting valuation studies in estuarine management planning efforts. Though developed here for estuarine management, the keyword hierarchy and final ecosystem goods and services approach have broad applicability and transferability to other environmental management scenarios.

## 1. Introduction

There is growing recognition that linking environmental protection and management decisions to the social and economic value of ecosystems can lead to actions that not only meet conservation goals, but additionally create social and economic returns on investment that gain greater stakeholder support than decisions focused mainly on ecological endpoints [1,2,3,4]. Yet, the indicators assessed, monitored, and reported in environmental management traditionally have focused on ecological or biophysical attributes, with weak connections, if any, to social or economic outcomes [5].

The National Estuary Program (NEP) and National Estuarine Research Reserve System (NERRS) are two federally-funded programs that aim to preserve ecologically, socially, and economically important estuaries within the United States by promoting stewardship, monitoring, and management. The NEPs, established under a 1987 revision to the Clean Water Act (CWA), are under federal guidance of the U.S. Environmental Protection Agency (US EPA) and are mandated to protect the biological, physical, and chemical integrity of the estuary, including water quality, fish and wildlife, recreational activities, and other designated uses [6,7]. The NERRS was first established through the Coastal Zone Management Act of 1972 as a partnership between coastal states and the National Oceanic and Atmospheric Administration (NOAA). The NERRS is mandated to protect estuarine resources to ensure a stable environment for research, enhance public understanding of estuaries, and provide opportunities for public education [8].

Federal estuary management programs have successfully used ecosystem services concepts to frame management issues and communicate with stakeholders [9,10]. A few programs have leveraged monetary valuation studies to calculate the dollar value of the estuary to the local economy and market improvements to stakeholders. However, widespread implementation of ecosystem services assessments in estuary management has been limited due to concerns of being too technical and nuanced to convey to stakeholders, or requiring funding and expertise to implement beyond commonly-valued resources such as fisheries [9,10]. As a result, estuary management criteria are still often limited to a narrow set of indirect proxies for ecosystem services, such as seagrass cover or water quality [9]. An analysis of just how common ecosystem services concepts are in estuarine management plans, including what stakeholders are likely to benefit or be impacted by decisions, can lay the groundwork for ecosystem services assessments by demonstrating the breadth of ecosystem services that can be considered, linking ecosystem services explicitly to different stakeholder groups, and expanding the perception of available assessment techniques.

Numerous frameworks have been proposed for cataloging ecosystem services (e.g., [1,11,12]). The final ecosystem goods and services approach, in particular, is motivated by the need for indicators that facilitate social and economic interpretation of ecological condition [13]. Along a continuum of ecological production, intermediate ecosystem goods and services (e.g., habitat quality) require additional steps to reach the ecological features (e.g., harvestable fish) directly experienced by beneficiaries [11]. Final ecosystem goods and services, in contrast, identify and measure the biophysical attributes that are most directly relevant to human well-being [11,13,14].

Final ecosystem goods and services require the identification of both the good or service and the beneficiary, as well as the environmental context. As such, the approach can bring clarity to environmental management by taking intermediate ecosystem services, such as water quality, and asking “for what?”, “for whom?”, “by what?”, or “where?”. The answers to these questions can affect which indicators or decision alternatives should be under consideration, reveal new or under-represented stakeholders to target for outreach or participation in the decision process, and may expose multiple complementary objectives or potential tradeoffs to be considered [14].

To test the hypothesis that final ecosystem goods and services can bring clarity to environmental management and elucidate the ecosystem contributions to human well-being, we systematically analyzed estuary management plans. We utilize the Final Ecosystem Goods and Services Classification System (FEGS-CS; [11]) to provide a structured framework for identifying ecosystems (Environmental Class), the potential ecosystem services they provided (FEGS Class), and their beneficiaries (Beneficiary Class). Our primary result was a comprehensive list of final ecosystem goods and services for estuarine watersheds, derived from estuary management plans. This list can serve as a starting point for identifying ecosystem services and their beneficiaries for both new estuarine applications where ecosystem services have not previously been considered, or to update and expand existing plans where ecosystem services may have been only briefly mentioned or narrowly considered.

The application of this framework to demonstrates the number of ecosystem services and types of beneficiaries who are potentially benefitting from national estuarine programs illustrates that natural resource managers are already implicitly using ecosystem goods and services in planning. Additionally, we assess the degree to which estuarine management programs may indirectly or directly impact provisioning of ecosystem services in habitats and beneficiaries throughout the watershed, not just the estuary. Though developed and applied here for estuarine management, we discuss the broad applicability and transferability of the final ecosystem goods and services approach to environmental management, including defining management goals, identifying stakeholders, developing meaningful indicators, and conducting valuation studies.

## 2. Methods

### 2.1. Theoretical Framework: Final Ecosystem Goods and Services Classification System

**Figure 1:**
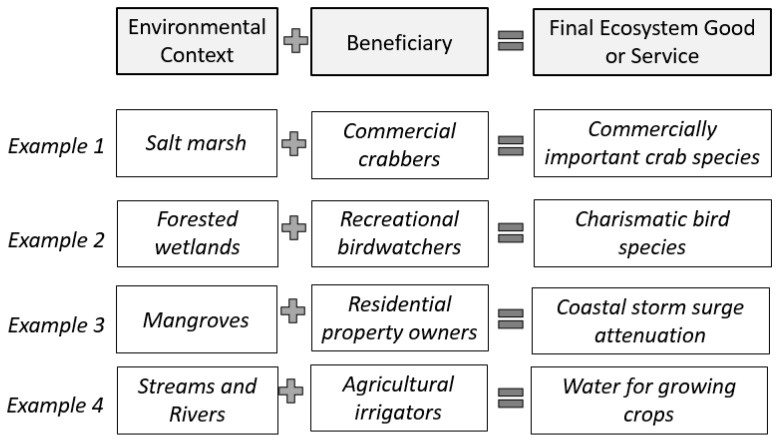
Illustration and examples of the three elements needed to define final ecosystem services.

The Final Ecosystem Goods and Services Classification (FEGS-CS) system provides a framework to standardize and define ecosystem services with explicit connections to both the landscape and specific beneficiaries [11]. FEGS are defined by an Environmental Class that supplies a type of good or service to a Beneficiary Class (Figure 1; Table 1).

The connection between all three categories (i.e., FEGS type, Environmental Class, Beneficiary Class) is important because determining what to measure will depend on all three—“water” from “streams” to an “agricultural irrigator” is not the same as “water” from “streams” to a “industrial processor”. An irrigator, for example, may require low concentrations of salinity, chemical, and pathogens during the growing season to ensure crops and their consumers are not harmed. An industrial processor that uses water primarily for cooling, in contrast, may care primarily about water quantity and temperature. Management actions to protect water resources may benefit both, but not necessarily, depending on the specific biophysical attributes of “water quality” and the degree to which they improve. The classes in the FEGS-CS strive to be comprehensive and non-duplicative. The FEGS-CS is designed to untangle intermediate from final ecosystem goods and services, and provides a foundation for identifying, measuring, and mapping indicators that are meaningful and relevant to stakeholders.

### 2.2. Sampling Method: NEP and NERRS Management Plans

To test the applicability of ecosystem goods and services in management, we applied the FEGS-CS as a structured framework, or codebook, to organize a document analysis of the NEP and NERRS management plans. NEP and NERRS documents provide a representative sample to better understand how management organizations utilize ecosystem goods and services because they are geographically distributed throughout the coastal zones of the United States, including Alaska, Hawaii, and the Great Lakes (Table 2); nearly every biophysical environment in the FEGS-CS is represented. and each NEP or NERRS site develops their own comprehensive management plan approximately every ten years. Recent management plans were obtained for each site from the US EPA (https://www.epa.gov/nep; accessed September 2017) or NOAA host websites (https://coast.noaa.gov/nerrs/; accessed September 2017) for document analysis (Table 2).

### 2.3. Document Analysis

We reviewed a sample of management plans to develop a list of keyword concepts to describe each category of Beneficiary (Table 3), Environment (Table 4), and FEGS (Table 5) for document coding. Environment class keywords were largely derived from the US Geological Survey’s (USGS) National Land Cover Dataset (NLCD, [15]), with the addition of “groundwater” and “atmosphere” classes (Table 4). Beneficiary categories overlap the North American Industrial Classification System (NAICS, [16]) of economic activity, with the addition of a number of beneficiaries that tend to utilize the environment with little economic impact, including subsistence, learning, and non-use values (Table 3; [11]). An additional category “all humans” was included to represent ecosystem services that broadly benefit everyone’s health, safety, or quality of life, without being tied to a particular economic or social activity. FEGS keywords categorize the components of the environment with which beneficiaries most directly interact (Table 5). For most types of FEGS, beneficiaries directly interact with a physical good (e.g., air to breathe, water to drink, land on which to build, flora to view, fauna to hunt). The FEGS type “presence of the environment” was defined by services that provide indirect benefits (e.g., buffering air pollutants, regulating flooding, opportunities to interact with nature) but that still may be directly relevant to certain beneficiaries. Theoretically, there are over 16,700 possible combinations of FEGS in the FEGS-CS, defined by combinations of a Beneficiary category (47), Environment category (17), and FEGS type (21). There are also additional possibilities of unclear or unspecified classes of Beneficiary, Environment, or FEGS (e.g., “people benefit”, “nature”, “natural resources”) that could not otherwise be assigned to a specific type. In reality, certain FEGS combinations are unlikely or impossible. 

To ensure consistency in how management plans were reviewed, we developed an automated process using a script written in *R* [17] to search each document for the keyword concepts associated with each Beneficiary, Environment, and FEGS category (Table 3, Table 4 and Table 5). We developed a comprehensive list of keywords associated with each concept, such as a list of frequently mentioned types of fauna (Supplementary Table S1). Keywords could be paired with associated words to distinguish phrases describing ecosystem services (e.g., “benefit”, “enjoy”, “hunt”) from those merely describing ecological condition. Keywords could also be paired with words to exclude false hits (e.g., “hunt” but not “shunt”). We analyzed all 28 NEP and 29 NERRS management plans to identify the range of biophysical environments and potential FEGS in coastal zones of the United States.

The R-script was then applied to read each sentence in each document, and flag any sentences containing valid keywords and any associated paired words for each Beneficiary, Environment, or FEGS category (Supplementary Table S1). The next step was to connect which FEGS was being supplied by which Environment to which Beneficiary for each sentence. Many sentences, for example, contained multiple concepts (e.g., “opportunities for recreational hunting in forests and commercial fishing in open water”). For each sentence, the R-script first generated all possible combinations of Beneficiary/Environment/FEGS classes based on the categories assigned to that sentence. If a particular class was not mentioned in a sentence, the category was assigned as “unspecified”. Then for each category, only the most likely combination was retained based on the overall frequency of that combination across all documents (e.g., “Recreational Hunting/Fauna/Forest” is more likely than “Recreational Hunting/Fish/Open Ocean”). The script is adaptive as combinations excluded for one sentence might be retained for another sentence, depending on the specific combinations of categories assigned to that sentence. Repeating this process for each sentence and each document led to a master list of possible FEGS. Each combination in this master list was then manually checked by reviewing at least two example sentences assigned to that combination, randomly drawn from all documents. Any false hits, typically arising from multi-concept sentences (e.g., “hunting, agriculture, and fishing”) were excluded from the master list. The culled master list of FEGS was then used to count the number of documents that mention each combination of Beneficiary, Environment, and type of FEGS (i.e., out of 28 NEPs or 29 NERRs).

The document analysis was developed iteratively, comparing automated results to a manual read by two of the co-authors for a random sample of sentences and documents to check (1) for missing concepts that did not get assigned to a category and needed to be added to the keyword list, (2) for false hits that could be minimized with paired associated words or exclusion words, (3) that the most likely FEGS combinations assigned to each sentence were indeed applicable to that sentence, and (4) that valid FEGS combinations were not being excluded. At each iteration, the keyword list and culled master list of FEGS were revised, until any further iterations produced minimal changes to the final FEGS counts across documents (i.e., <5% change in counts). In an independent verification, a third co-author provided a manual read of a random selection of management plan pages to identify FEGS combinations based on their familiarity with FEGS concepts, but without reference to the detailed master keyword list. The document analysis identified FEGS consistent with the independent manual read.

Document analysis was used to assess the frequency that ecosystem goods or services, their beneficiaries, and the biophysical environment providing them are considered in NEP and NERRS management plans. Because documents vary widely in length and structure (i.e., discussion of goals relative to discussion of planned actions), counts of sentences do not necessarily reflect that a particular ecosystem service or beneficiary is more important to one national estuary than another. Therefore, we utilized a presence or absence approach to our analysis on whether a particular class, or combination of Beneficiary + Environment + FEGS classes, was mentioned at all in each document, rather than the frequency of times it was mentioned. The document analysis did not distinguish whether mentions of ecosystem services were related to goal setting or merely background information. For purposes of the analysis, we assume that if a concept was mentioned, then it was “important”, regardless of whether it was specifically identified as a goal. Indeed, some mentions of ecosystem services may reflect resource uses that could be considered a stress on the ecosystem, such that the management goal might be to reduce rather than protect such uses.

Logistic regressions were conducted using general linear models in *R* (function “glm”; family binomial) to test whether the frequency of plans mentioning each category of Beneficiary, Environment, or FEGS differed between NEP and NERRS. A two-sample *t*-test was used to compare the total number of sentences per document mentioning ecosystem services concepts between NEP and NERRS plans. In particular, we predicted NERRS plans would have a heavier focus on ecosystem services for research and education, while NEP plans would have a stronger focus on ecosystem services related to recreation, fishing, or other designated uses as mandated under their respective programs. However, we expect the combined comprehensive list of FEGS may broaden the perceived potential benefits of estuarine management programs.

## 3. Results

### 3.1. Frequencies of FEGS-CS Classes in Management Plans

The 28 NEP and 29 NERRS management plans varied in length, ranging from 615 to 26,195 sentences searched for each document in the analysis, with a total of over 303,000 sentences reviewed. NEP and NERRS plans did not differ significantly in length (*t*-test, *t* = 0.402, *p* = 0.689, df = 55; mean = 5316 sentences per plan). Discussion of ecosystem services concepts was common in plans, with an average of 22.4% of sentences in each document identified as containing keywords or phrases related to ecosystem goods and services, with NEP and NERRS again not significantly different (*t*-test, *t* = 1.05, *p* = 0.296, df = 55).

In total, more than 5807 potential combinations of FEGS type (Table 5), Beneficiary (Table 3), and Environment (Table 4) were identified in sentences, including many combinations in which one or more of these classes were unspecified or unclear in the documents. Of the potential FEGS combinations, 1614 were determined to be valid, in terms of being applicable to at least one management plan (Supplementary Table S2). There were an additional 664 valid combinations for which the beneficiary, environment, or ecosystem service was unclear or not specified (e.g., statements such as “natural resources are important”). On average, each document mentioned 270 specific FEGS combinations (min = 68, max = 602 per document), with an average of an additional 155 combinations for which the beneficiary, environment, or ecosystem service was unclear or not specified. Examples of the most common combinations for who is benefitting from each type of FEGS in which environment are given in Table 6.

**Figure 2:**
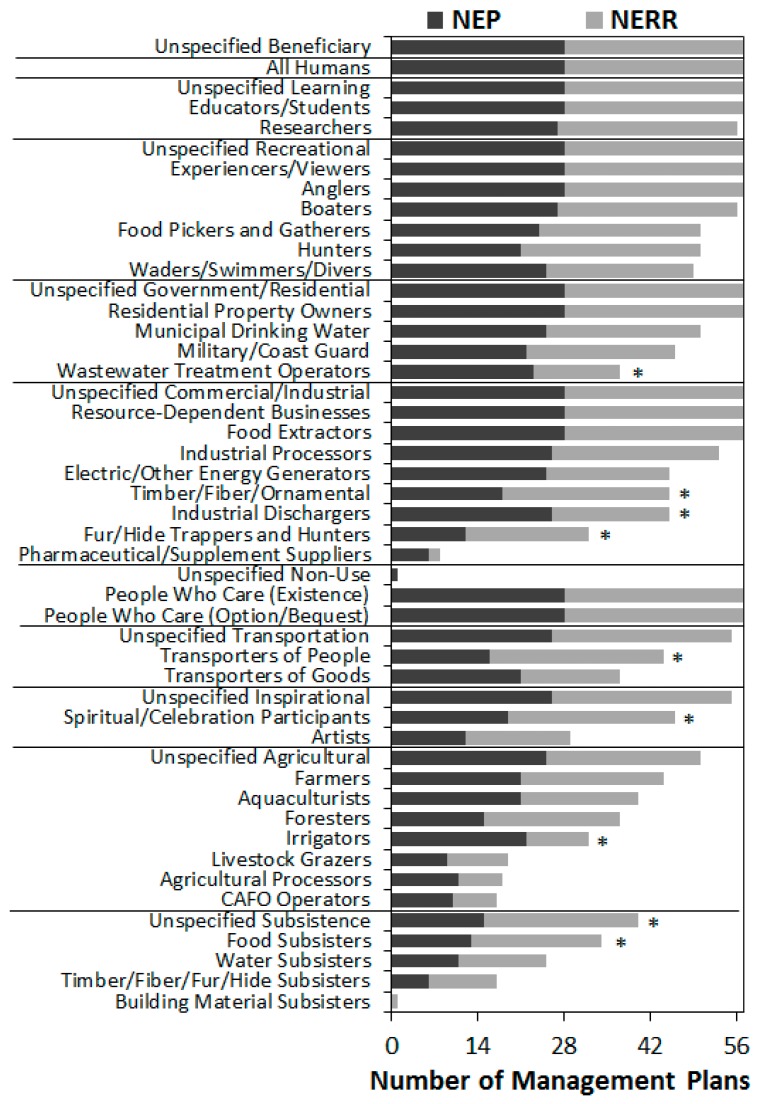
Number of NEP or NERR management plans mentioning each beneficiary category. Asterisks denote categories for which counts differed significantly (logistic regression; *p* < 0.05) between NEP and NERR.

All categories of Beneficiaries were mentioned by at least one management plan (Figure 2). On average, each management plan mentioned 35 Beneficiary types (min = 21, max = 42 per document) out of the 47 possible types listed in Table 3. Beneficiaries mentioned by all or almost all NEP and NERRS plans included educators and students, researchers, experiencers and viewers, recreational anglers, boaters, residential property owners, resource-dependent businesses, food extractors, and people who care either for existence or option value. All or almost all management plans also broadly discussed benefits for learning, recreation, government, residents, commercial businesses, industry, transportation, and inspiration, although plans may or may not have identified specific beneficiary categories. Broad discussion of agricultural or subsistence benefits were also common, but to a lesser degree. The least common beneficiaries identified in plans included commercial fur trappers or hunters, pharmaceutical or supplement suppliers, and a number of agricultural or subsistence beneficiaries (e.g., Confined Animal Feeding Operations (CAFOs), building material subsisters), which may indicate that these are not common activities in coastal zones.

**Figure 3:**
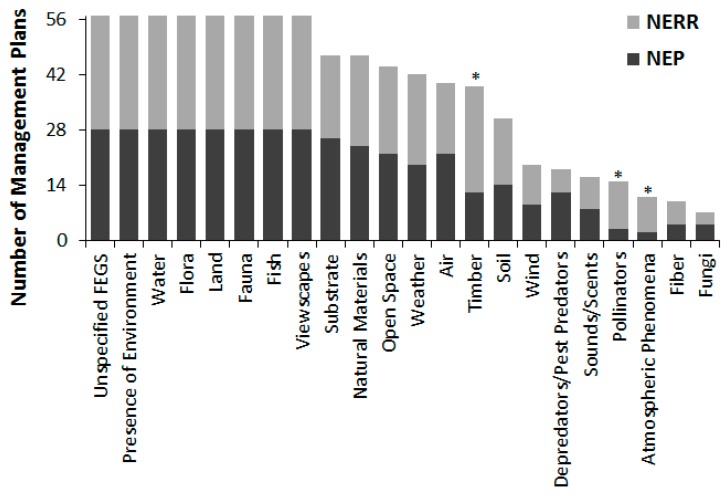
Number of NEP or NERR management plans mentioning each FEGS type. Asterisks denote categories for which counts differed significantly (logistic regression; *p* < 0.05) between NEP and NERR.

Each management plan on average mentioned 14 types of FEGS (min = 9, max = 19 per document) out of 21 possible types listed in Table 5. The most common types of FEGS, discussed by all 28 NEPS plans and all 29 NERRS plans, included presence of the environment, water, land, flora, fauna, fish, and viewscapes (Figure 3). Each type of FEGS was mentioned by at least one management plan, although fungi, atmospheric phenomena, fiber, pollinators, sounds and scents, depredators, and wind were the least common.

**Figure 4:**
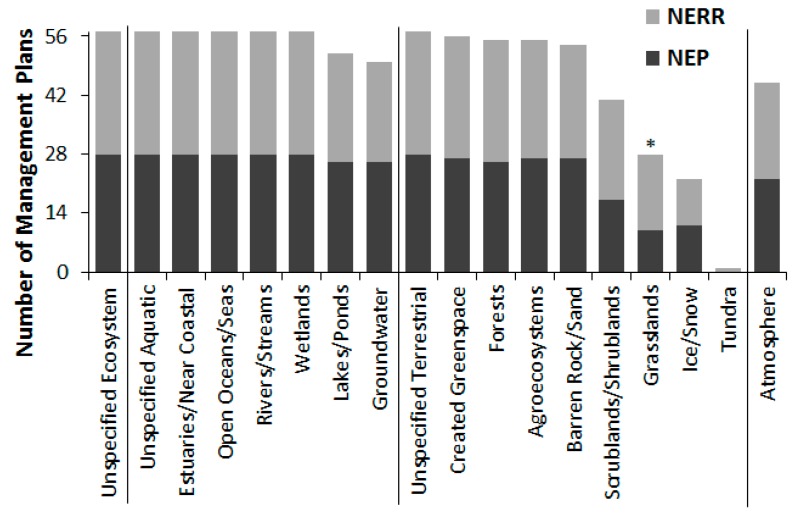
Number of NEP or NERR management plans mentioning each category of environment. Asterisks denote categories for which counts differed significantly (logistic regression; *p* < 0.05) between NEP and NERR.

Despite the obvious focus of NEP and NERRS management plans on estuary ecosystems, all other types of environments were identified as providing benefits relevant to at least one of the estuary management programs (Figure 4). Of the 17 types of environments listed in Table 4, on average 14 types were mentioned in each management plan (min = 10, max = 16 per document), reflecting the reality that some estuaries are extensive and include terrestrial environments. Environments commonly mentioned included open oceans, rivers and streams, lakes, wetlands, groundwater, created greenspace, forests, barren rock, agroecosystems, and atmosphere. Even tundra and ice/snow were identified as providing ecosystem services relevant to a few management programs.

### 3.2. Who Benefits from FEGS and Where?

The key to a final ecosystem goods and services approach is connecting the Environmental Class that supplies a type of FEGS to a Beneficiary Class. The most common ecosystem services mentioned were benefits of aquatic environments to provide water resources to the local community or fish to commercial food extractors, and the presence of estuary and coastal environments that provide opportunities for educators and students (Table 6). Plans also commonly mentioned the existence value of flora and fauna in the estuary and the availability of land for development or protection by the local community.

**Figure 5:**
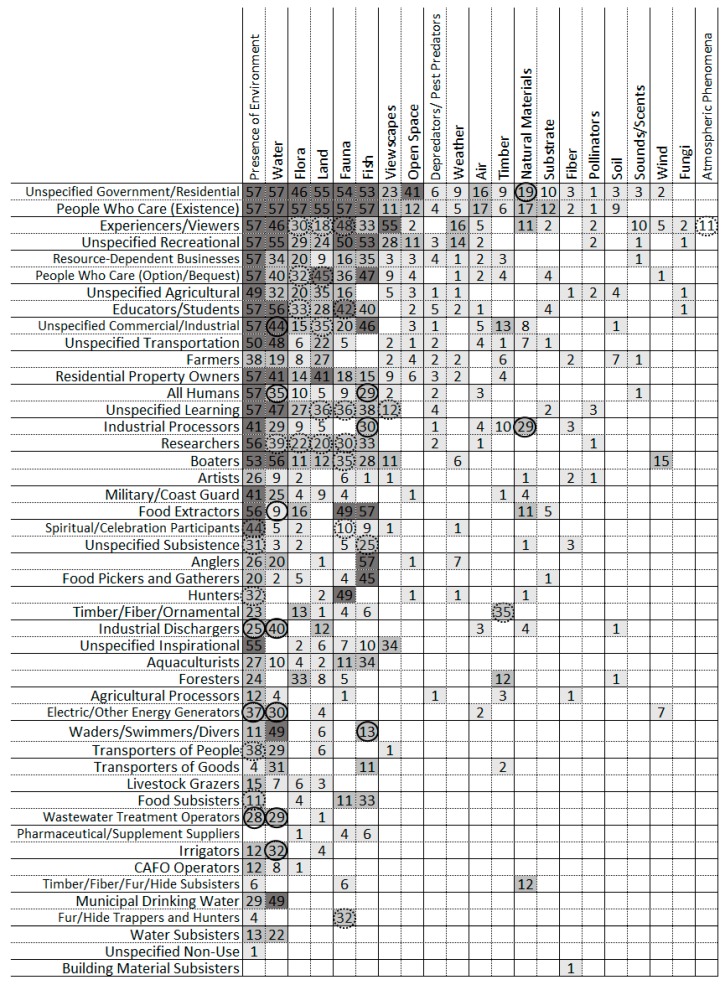
Number of management plans mentioning each type of FEGS with each category of Beneficiary. Circles indicate a particular combination was significantly (logistic regression; *p* < 0.05) more common in either NEP plans (solid) or NERR plans (dashed).

**Figure 6:**
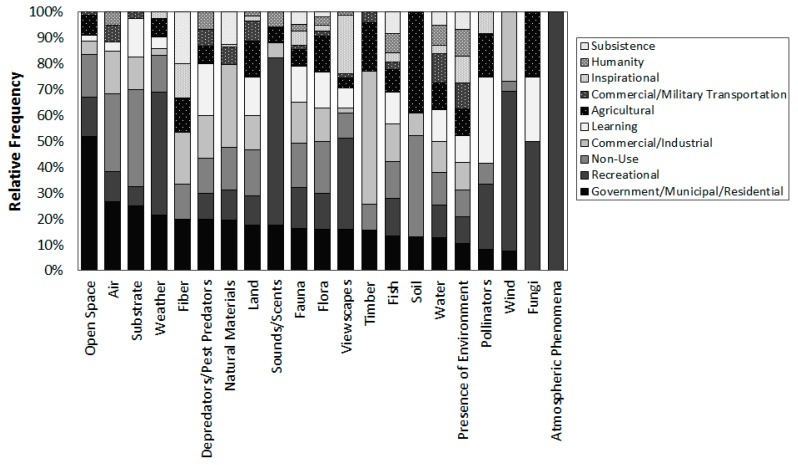
Relative frequency by which documents linked each type of FEGS to different types of Beneficiaries.

Each type of FEGS often was linked to multiple beneficiaries (Figure 5 and Figure 6). The presence of the environment had the widest array of potential beneficiaries, followed by water, flora, land, and fauna. Almost all management plans linked these five ecosystem services to government and residential beneficiaries and people who care (existence value), and many plans identified them as also benefitting various classes of recreation, commercial and industrial, learning, agriculture, transportation, and option/bequest value. In contrast, other types of FEGS were identified to have only a few beneficiaries. Recreational users, for example, were the dominant beneficiaries of sounds and scents, wind, fungi, and atmospheric phenomena.

Similarly, some beneficiaries, including specific types of subsisters (e.g., building material, water, timber or fur), commercial fur and hide hunters, and municipal drinking water operators, were linked to only one or a few types of ecosystem services (Figure 5). Other beneficiaries, including experiencers and viewers, resource-dependent businesses, and people who care, were identified as benefitting from many different types of ecosystem services. Anglers, for example, were universally identified as benefitting from fish (all 57 plans), but several plans also identified anglers as benefitting from opportunities to be in nature, water, land to access fishing areas, scenic open vistas, and pleasant weather.

**Figure 7:**
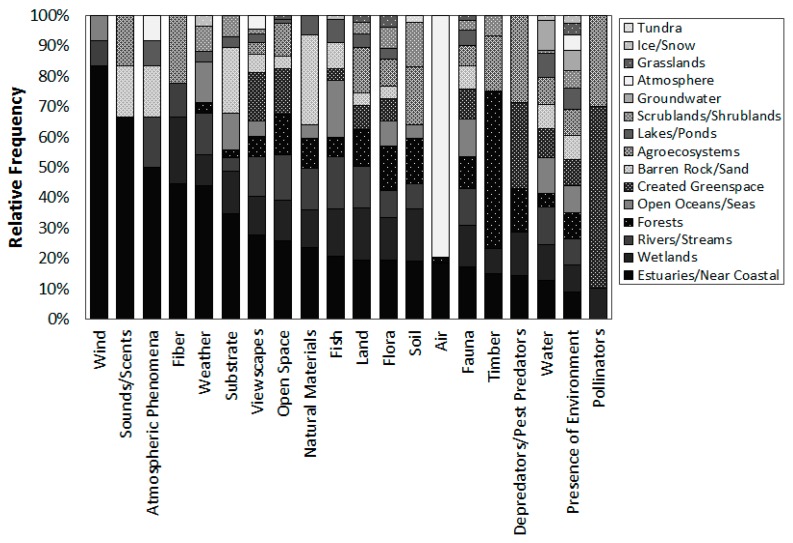
Relative frequency by which documents linked each type of FEGS to different types of Environments.

Planning documents attributed estuaries and near coastal marine environments with providing all types of ecosystem services (Figure 7). A number of FEGS, including wind, sounds and scents, atmospheric phenomena, fiber, weather, and substrate, were largely attributed to estuaries, near coastal marine, and wetland environments. Other FEGS, including viewscapes, open space, presence of the environment, flora, fauna, and water, were identified as being provided by many different types of estuarine and non-estuarine environments within the broader watershed and adjoining seas, including both terrestrial and aquatic. Pollinators, timber, and depredators were largely associated with terrestrial environments, particularly created greenspace, forests, and agroecosystems.

### 3.3. Similarities across NEPs and NERRs

In general, NEP and NERRS management plans were very similar in their discussion of types of ecosystem services, environments providing them, and who is benefitting. Subsisters, particularly food subsisters, were significantly more likely to be mentioned in NERRS plans than NEP plans, as were celebration participants and commercial timber, fiber, or fur industries. On the other hand, NEP plans were significantly more likely to mention wastewater treatment operators, industrial dischargers, or irrigators (Figure 2). The frequency of all other Beneficiary categories was not significantly different between NEP and NERRS plans. Similarly, the frequency of Environment and FEGS categories was almost universally comparable between NEP and NERRS plans, with NERRS plans only slightly more likely to mention timber, pollination, or atmospheric phenomena (Figure 3) or grassland environments (Figure 4).

NEP plans and NERRS plans were also similar in the frequencies in which types of FEGS were connected to classes of beneficiaries (Figure 5). NEP plans were significantly more likely to mention multiple benefits of fish, including public health, industrial processers, and swimmers and divers, or to mention multiple benefits of water, including to commercial businesses, industrial discharges, irrigation, wastewater treatment, and energy generation. In contrast, NERRS plans were significantly more likely to mention multiple benefits of flora, fauna, fish, or land to option/bequest value, learning, education, researchers, or experiencers and viewers.

## 4. Discussion

### 4.1. Relevance of FEGS to Estuary Management Programs

Discussion of ecosystem goods and services and their beneficiaries was common throughout both NEP and NERR management plans. Presence of the environment, water, flora, land, fauna, fish, and viewscapes were universally common across all plans, often attributed as benefitting a variety of types of beneficiaries, and deriving from a variety of environments. Many of the FEGS identified in the document analysis reflected the mandated goals of the respective programs to protect estuarine water quality and habitat for recreation or other designated uses, or provide opportunities for learning, education, and research. In many cases, however, plans discussed the use of ecosystem goods and services by beneficiaries as a stressor to be managed (e.g., use of water by industrial dischargers; presence of the environment to buffer agricultural runoff). In other cases, ecosystem goods and services were mentioned merely to provide additional context for the report, but management goals and actions were still focused on estuarine health.

In total, more than 1600 unique FEGS combinations were identified across plans, where a single combination is a certain type of good or service provided by a certain class of environment benefitting a certain class of beneficiary. An additional 600 FEGS combinations made reference to unspecified beneficiaries, environment, or types of ecosystem services. These were often broad statements (e.g., “natural resources are important”), sometimes but not always followed subsequently with clarifying information. The large number of FEGS combinations illustrates several motivations behind the final ecosystem goods and services approach. First, broad characterizations of ecosystem goods and services such as “water quality and quantity” can mean different things to different beneficiaries, or in different environments. Second, one-to-one relationships between beneficiaries and ecosystem services (e.g., anglers benefit from fish) were uncommon. Instead, most types of beneficiaries benefitted from multiple types of ecosystem goods and services, and in various environments. Third, a structured approach, such as the FEGS-CS, can reveal less commonly considered types of FEGS (e.g., sounds and scents, pollinators, atmospheric phenomena), beneficiaries (e.g., subsistence, agriculture), or their combinations (e.g., hunters use of natural materials) that could be integrated into future planning or assessment efforts.

The NEP and NERRS management plans were remarkably similar in which beneficiaries, environments, and types of FEGS were relevant to program management. NERRS plans were more likely to mention a greater variety of ecosystem goods and services benefitting learning, educators, and researchers, reflecting their mandate under the Coastal Zone Management Act [8] to protect and provide opportunities for learning and education. NERRS plans were also more likely to mention benefits of ecosystem goods and services to subsisters or traditional users. NEP plans, in contrast, were more likely to mention benefits of water or fish to a greater variety of municipal, industrial, and agricultural beneficiaries, in line with their focus on water quality, habitat, and designated uses [6]. For the most part, however, the frequency of FEGS, their beneficiaries, and the environments providing them were not significantly different between NEP and NERRS plans.

Both NEP and NERR management plans also universally recognized the importance of a variety of environments in the broader watershed, not just the estuary itself, in providing ecosystem goods and services to beneficiaries. A watershed management approach considers how land-based stressors, surface water runoff, and groundwater impact estuaries and connected coastal ecosystems [18], and allows a broader consideration of issues and goals. It can also be more holistic, integrated, and collaborative [19,20]. Decisions to protect coastal ecosystem goods and services may have negative impacts for the use of other ecosystem goods and services in the watershed [21,22]. Management actions to protect coastal resources may gain broader stakeholder support if they consider and respond to concerns of beneficiaries in both the coastal zone and the watershed [22,23,24].

### 4.2. Demonstration of FEGS in Management Plans

There is a perception that actively integrating final ecosystem goods and services into environmental management is challenging because of the impression that formal assessments of ecosystem services require significant information from economic and social sciences, are saddled with uncertainty, and are embedded within a decision environment of multiple conflicting stakeholder perspectives and limited resources [20,25]. This study demonstrates that final ecosystem goods and services concepts are already part of estuarine and coastal management planning. We will demonstrate how one NERRS, the Lake Superior National Estuarine Research Reserve (LSNERR), included ecosystem services in their management plan by using FEGS as an analytical tool.

The LSNERR is located on the South Shore of Lake Superior and along the St. Louis River estuary in Superior, Wisconsin. LSNERR protects around 17,000 acres of a combination of habitats including a freshwater bay mouth sand bar complex, freshwater estuarine wetlands, forest, and red clay bluffs [26]. Like other NERRs, LSNERR has a research, stewardship, training, and educational programming. The LSNERR has a strong community and stakeholder education component that includes a museum and classroom facility called the Estuarium, an annual research conference, and a teacher education program about estuaries. Moreover, the LSNERR conducts environmental research throughout the estuary and conducts a System Wide Monitoring Program (SWMP).

Although all FEGS can signal a value for a specific environmental feature, this discussion will focus on the general “Presence of the Environment” because it emerged as an important theme in the study of all management plans, including the LSNERR management plan [26]. For this demonstration, we identify the beneficiary-environment combinations that appeared in 10 sentences or more. Moreover, Presence of the Environment is a broad category that includes interacting with nature for recreation and regulating services, which are important components of the NERR mission (e.g., education, recreation, and climate change adaptation). This FEGS provides an opportunity to how the interests of different types of people can be included in environmental management. People can be general (i.e., people who care) or more specific (i.e., governments and municipal officials), but the idea is to identify and characterize the different types of people and attach them to valued environmental features.

The LSNERR management plan identified several different populations of beneficiaries, including educators and students, experiencers and viewers, governmental agencies, learners, people who care, and researchers (Table 7), and connected them directly to environments providing benefits. For example, experiencers and viewers were associated as benefitting from created greenspace. The LSNERR manages the Superior Municipal Forest, which is a site for recreation and is managed as a park. People go to the forest to ski, hike, hunt, ride all-terrain vehicles (ATV), canoe, or kayak [27]. At the same time, governments and municipal entities were related to the estuaries, forests, and wetlands. Governmental agencies are some of the most important NERR clients in the Coastal Training Program, as the NERR provides technical support through programming. Finally, the LSNERR also reaches out the larger community to identify and connect with people who care about the environment.

This demonstration illustrates how FEGS can be an intuitive method to connect beneficiaries with environmental features. We contend that FEGS can be an intentional method for connecting people and the environment, and a roadmap for managers to consider the environment they manage with the people they serve.

### 4.3. Integrating FEGS into the Decision Process

Our study demonstrates that final ecosystem goods and services concepts can be useful in identifying goals and communicating potential benefits of estuarine management, even if a formal assessment or monetary valuation of ecosystem services was not conducted. Technical assessments or cost-benefit analysis could be done, but are not required. Instead, FEGS can help ensure that “what is important” is clearly established upfront, ultimately leading to activities and projects that have a higher likelihood of acceptance across a variety of stakeholders. Indeed, FEGS concepts can be valuable throughout the decision process, from early planning stages to implementation, with variable levels of commitment to resources and time [14], including to:

- Broaden the decision context and stakeholder inclusion: Identification of FEGS can help ensure that a fuller suite of potential benefits and costs are under consideration, or that key issues or stakeholders are not overlooked [14]. A process that is more inclusive of a broad range of potential beneficiaries can also provide innovative ideas and insights into what is feasible [20,28].
- Define objectives with reduced ambiguity: By explicitly linking ecosystem services to the environment providing them and the beneficiary, FEGS also help to reduce ambiguity and confusion about what is really meant by management objectives. For example, water resources were universally mentioned across management plans, however the multiple types of beneficiaries benefiting from those water resources would each have their own perspectives on what exactly should be protected and why.
- Identify meaningful indicators for comparing options or monitoring success: FEGS metrics precisely define biophysical measures of the environment tied to a specific beneficiary. Water quality for recreational anglers, for example, may be reasonably measured by water visibility or water depth to operate a boat. Water quality for industrial processors, in contrast, may be represented by water quantity, temperature or presence of biofouling organisms.
- Develop creative management actions: Management actions proposed to improve water quality for swimming may be very different from actions proposed to improve water quality for agricultural uses. Identification and assessment of FEGS may lead to clear win-win or low risk management actions, especially where multiple beneficiaries are using similar types of FEGS in similar ways.
- More precisely define what is needed for ecosystem services assessments: Because final ecosystem goods and services are essentially a cause-and-effect flow between the environment and a beneficiary, FEGS can form the conceptual basis for estimating consequences of alternative actions on stakeholder objectives. Group deliberations or the use of graphical diagrams (e.g., influence diagrams; conceptual models) may be sufficient to determine that benefits would clearly be higher under one action than another [29]. Where uncertainties are too great, assessments may rely on expert judgments. If greater precision is needed empirical data may be collected, quantitative predictive models applied (e.g., [30]), or economic valuation studies conducted (e.g., [31,32,33]). Alternatively, an assessment of “how many people benefit” as beneficiaries of FEGS may be part of a rapid approach for comparing the potential benefits of management options [34]. A FEGS approach helps to avoid the fallacy of collecting certain kinds of data or applying models solely for reasons of familiarity or convenience [14].
- Evaluate trade-offs and common-ground across stakeholders: Actions will likely create tradeoffs across FEGS, particularly if use of FEGS by one beneficiary impairs the use of another beneficiary. One benefit of a FEGS approach is that stakeholders rarely represent a single beneficiary type, but instead are combinations of multiple beneficiaries. By directly connecting the environment to the ways stakeholders use it, FEGS sets the stage to uncover commonalities across disparate stakeholder groups. Stakeholders may be more willing to accept a small loss in something “very important” to prevent a large loss in something “less important”. The frequencies of FEGS combinations across management plans may provide a rough approximation for the weight of importance of different types of FEGS for each beneficiary.

## 5. Conclusions

The document analysis presented here illustrates the broad range of ecosystem goods and services and their beneficiaries that are relevant to estuary management programs, because the programs are already using the concepts. Due to nature of our analysis, it is possible that uncommon or unusual FEGS or beneficiaries, of specific relevance to an individual estuary, may have been missed in the automated search and validation process. However, our goal was to facilitate comparison across a large number of documents, identify common concepts relevant across programs, and provide a framework that could assist in future estuarine management planning and implementation. As such, the hierarchical list of FEGS generated from document analysis can serve as a foundational tool for defining management goals, identifying stakeholders, developing meaningful indicators, and evaluating potential tradeoffs in estuarine management planning efforts. This study did not take the additional step of identifying or recommending how FEGS could being measured. Indeed, biophysical measures of ecosystem services with direct relevance to beneficiaries are broadly lacking [35]. However, efforts are underway to identify and recommend FEGS metrics for different ecosystems (e.g., [36]).

Though developed here for estuarine management, the keyword hierarchy and final ecosystem goods and services approach have broad applicability and transferability to other environmental management scenarios. The developed keyword hierarchy, in particular, covers a far broader suite of environments than just estuarine ecosystems, and could serve as a starting point for identifying FEGS and beneficiaries in other systems (e.g., forest management, brownfield revitalization), with a comparable review of management plans or stakeholder engagement employed to identify additional concepts that may be unique to those systems.

Although environmental managers are increasingly following recommendations to consider ecosystem services in planning [1], ambiguity in how ecosystem services are referenced and monitored (e.g., water quality, habitat) may not resonate with stakeholders and fosters ambiguity about whether management actions are achieving desired results. A final ecosystem goods and services approach can facilitate increased understanding of how biophysical processes integrate with social and economic well-being to give decision-makers a way to communicate with stakeholders using relatable language. FEGS set the stage for identifying biophysical measures that are meaningful to both managers and stakeholders. Identification of FEGS can help ensure the key stakeholders, key objectives, and creative alternatives are not overlooked, and prioritize monitoring and assessment based on what is most relevant to connect management actions to objectives.

## Figures and Tables

**Table 1:** Categories of Environments, Beneficiaries, and Final Ecosystem Goods and Services (FEGS) in the FEGS Classification System (FEGS-CS) (from [11]), and example questions to identify them.

Categories		
Beneficiary Class: Who might be Impacted by Environmental Management Decisions?	FEGS Class: How are They Benefitting from the Environment?	Environment Class: What Ecosystems are Providing Those Benefits?
Agricultural	Water	Aquatic Ecosystems
Commercial/Industrial	Flora	Rivers and Streams
Government/Municipal/Residential	Presence of Environment	Wetlands
Commercial/Military Transportation	Fauna	Lakes and Ponds
Subsistence	Fiber	Estuaries/Near coastal/Marine
Recreational	Natural materials	Open Ocean and Sea
Inspirational	Open space	Groundwater
Learning	Viewscapes	
Non-use	Sounds and scents	Terrestrial Ecosystems
Humanity	Fish	Forests
	Soil	Agroecosystems
	Pollinators	Created Greenspace
	Depredators/Pest Predators	Grasslands
	Timber	Scrubland/Shrubland
	Fungi	Barren/Rock and Sand
	Substrate	Tundra
	Land	Ice and Snow
	Air	
	Weather	Atmosphere
	Wind	
	Atmospheric Phenomena	

**Table 2:** List of National Estuary Program (NEP) and the National Estuarine Research Reserve System (NERRS) management plans and approval year included in document review. For plans intended as an addendum to a prior version, both years were included.

National Estuary Program	National Estuarine Research Reserve System
Albemarle–Pamlico, North Carolina (2012)	Ashepoo–Combahee–Edisto, South Carolina (2011)
Barataria–Terrebonne, Louisiana (1996)	Apalachicola, Florida (2014)
Barnegat Bay, New Jersey (2002)	Chesapeake Bay, Maryland (2008)
Buzzards Bay, Massachusetts (2013)	Chesapeake Bay, Virginia (2008)
Casco Bay, Maine (2006)	Delaware NERR (2013)
Charlotte Harbor, Florida (2013)	Elkhorn Slough, California (2006)
Coastal Bend Bays and Estuaries, Texas (2016)	Grand Bay, Alabama (2013)
Delaware Inland Bays, Delaware (2012)	Great Bay, New Hampshire (2006)
Galveston Bay, Texas (1995)	Guana Tolomato Matanzas, Florida (2009)
Indian River Lagoon, Florida (2008)	He’eia, Hawai’i (2016)
Long Island Sound, New York-Connecticut (2015)	Hudson River, New York (2009)
Lower Columbia Estuary, Oregon (1999, 2011)	Jacques Cousteau, New Jersey (2009)
Maryland Coastal Bays, Maryland (2015)	Jobos Bay, Puerto Rico (2017)
Massachusetts Bays, Massachusetts (2003)	Kachemak Bay, Alaska (2012)
Mobile Bay, Alabama (2013)	Lake Superior, Wisconsin (2010)
Morro Bay, California (2012)	Mission-Aransas, Texas (2015)
Narragansett Bay, Rhode Island (2012)	Narragansett Bay, Rhode Island (2010)
New York—New Jersey Harbor (1996)	North Carolina NERR (2009)
Delaware Estuary, Delaware (1996, 2014)	North Inlet-Winyah Bay, South Carolina (2011)
Peconic Bay, New York (2001)	Old Woman Creek, Ohio (2011)
Piscataqua Region, New Hampshire (2010)	Padilla Bay, Washington (2008)
Puget Sound, Washington (2016)	Rookery Bay, Florida (2012)
San Francisco Estuary, California (2016)	San Francisco Bay, California (2011)
San Juan Bay, Puerto Rico (2000)	Sapelo Island, Georgia (2008)
Santa Monica Bay, California (2008)	South Slough, Oregon (2006)
Sarasota Bay, Florida (2014)	Tijuana River, California (2010)
Tampa Bay, Florida (2013)	Waquoit Bay, Massachusetts (2013)
Tillamook Bay, Oregon (1999)	Weeks Bay, Alabama (2007)
	Wells NERR, Maine (2013)

**Table 3:** Example keywords used to code each category of beneficiary (full list in Supplementary Table S1).

Beneficiary Class	Example Keywords for Coding
Agricultural	agriculture; agricultural
Irrigators	irrigator; irrigation; watering
Confined Animal Feeding Operations (CAFO) Operators	confined animal feeding lots or operations
Livestock Grazers	ranches; livestock grazing; pasture
Agricultural Processors	agricultural mills/processing; farm commodities or goods
Aquaculturalists	aquaculture; aquafarming; hatcheries
Farmers	farming; sugar/coffee plantation; crops orchards
Foresters	forestry; tree farm; silviculture; tree plantation
Commercial/Industrial	commercial; industry; business; commerce
Food Extractors	commercial/artisanal fishing or hunting (meat)
Timber/Fiber/Ornamental Extractors	timber industry; logging; shell mining; aquarium industry
Industrial Processors	manufacturing; factories; mining; oil/gas industry
Industrial Dischargers	industrial/manufacturing discharge; landfills
Electric/Other Energy Generators	power plant; electricity; renewable energy
Resource-Dependent Businesses	tourism; local shops; marina/waterfront; landscaping
Pharmaceutical/Supplement Suppliers	pharmaceuticals; food supplements; biotechnology
Fur/Hide Trappers and Hunters	commercial hunting/trapping (e.g., skin, hide, fur)
Government/Municipal/Residential	infrastructure; public use; community; residents
Municipal Drinking Water Operators	drinking water; public/municipal water supply or wells
Wastewater Treatment Plant Operators	wastewater/sewage treatment; treatment plant
Residential Property Owners	home/land owner; private property; residential development
Military/Coast Guard	military; Air Force; Army; Coast Guard; Marines; Navy
Commercial/Military Transportation	transportation; highways/roads; navigation
Transporters of Goods	ports or shipping of cargo; commodities; containers
Transporters of People	cruise ships; ferries; airport; harbor; passenger; parking
Subsistence	subsistence; tribal or traditional use; indigenous people
Water Subsisters	water for subsistence; cistern; rain garden; rain barrel; wells
Food Subsisters	subsistence hunting; subsistence fishing or food gathering
Timber/Fiber/Fur/Hide Subsisters	subsistence trapping; subsistence wood gathering; firewood
Building Material Subsisters	subsistence material gathering
Recreational	recreation; vacation; tourism; amenities; athletic;
Experiencers/Viewers	hiking; biking; camping; sightseeing; trails; birdwatching
Food Pickers/Gatherers	berry picking; recreational harvesting, including shellfish
Hunters	hunting; recreational hunting; sport hunting
Anglers	recreational fishing; sport fishing; catch and release fishing
Waders/Swimmers/Divers	snorkeling; scuba diving; swimming; wading; diving; bathing
Boaters	canoe; kayak; rowing; sailing; jet ski; surfing; watercraft
Inspirational	inspire; cultural/historic significance; treasure;
Spiritual/Ceremonial Participants	festival; observance; ceremony; wedding; spiritual
Artists	author; poet; painter; sculptor; pottery; photography
Learning	learn; nature/interpretive center; nature programs
Educators/Students	education; student; schools; field trips; teachers; teaching
Researchers	science; research; data collection
Non-Use	non-use values or resources
People Who Care (Existence)	conservation; unique/endangered species; right to exist
People Who Care (Option/Bequest)	heritage; landtrust; future generation; sustainability
All Humans	humanity; public health/safety; quality of life; welfare

**Table 4:** Example keywords used to code each sub-class of ecosystem (full list in Supplementary Table S1).

Ecosystem	Example Keywords for Coding
Aquatic	aquatic; water; benthic;
Rivers/Streams	river; creek; canal; stream; channel; riparian
Wetlands	wetland; bog; floodplain; marsh; fen; swamp; slough; salt hay
Lakes/Ponds	lake; pond; reservoir; vernal pool; flooded quarry;
Estuaries/Near-coastal Marine	estuary; tidal; reef; shipwreck; seagrass; mangrove; lagoon; delta; mudflat; bay; shore; coast; sound
Open Oceans/Seas	ocean; open water; continental shelf; deep water; sea; kelp forest; marine
Groundwater	groundwater; aquifer; geyser; underground reservoir
Terrestrial	terrestrial; upland; island; shell mound; mountain; land; watershed
Forests	forest; tree (e.g., oak, elm); wood; pineland
Agroecosystems	orchard; vineyard; crops; pasture; hay; agroecosystem; agricultural/silvicultural lands; plantations
Created Greenspace	park; trail; greenspace; airfield; athletic field; lawn; golf course; greenway; garden
Grasslands	prairie; grassland
Scrublands/Shrublands	sageland; dune; scrub; shrub; chaparral
Barren/Rock/Sand	quarry; barren; desert; beach; rock; sand; mining area
Tundra	tundra; alpine
Ice/Snow	glacier; snow; ice
Atmosphere	atmosphere; sky; clouds; air; wind

**Table 5:** Definitions used to code each category of FEGS and develop keywords (full list in Supplementary Table S1).

FEGS Class	Example Keywords for Coding
Air	fresh air for breathing; a medium to receive/dilute/transport emissions or ameliorate odors
Atmospheric Phenomena	aesthetic value of clouds, eclipses, sunrise, sunsets, rainbows, or twilight
Depredators/Pest Predators	biological control; natural pest/pathogen control or approaches that may leverage it (e.g., organic gardening, integrated pest management, agricultural environmental management)
Fauna	wildlife or animals (e.g., birds, mammals, reptiles, insects) that are unique ^1^, enjoyed as a resource ^2^, or identified for conservation ^3^
Fiber	fiber (e.g., salt hay, grass, reeds) harvested or collected for subsistence, building materials, or other benefits (e.g., products, milling, industry, pottery)
Fish	wild fish or shellfish (e.g., salmon, oyster, crab, grouper) that are unique ^1^, enjoyed as a resource ^2^, or identified for conservation ^3^
Flora	terrestrial or aquatic vegetation, including plant parts (e.g., flowers, branches), that are unique ^1^, enjoyed as a resource ^2^, or identified for conservation ^3^
Fungi	wild fungi/mushrooms that are unique ^1^, enjoyed as a resource ^2^, or identified for conservation ^3^
Land	availability of land for residential/commercial development; land identified for protection (e.g., preserve, restore, conserve, easement, trust); unspecified public lands
Natural Materials	natural materials collected as a resource for artistic or recreational use (e.g., ornaments, jewelry, firewood), consumption, or redistribution (e.g., fill, dredge) including rocks, shells, clay, acorns, honey, maple syrup, sand
Open Space	open space (e.g., greenspace, nature preserves, wildlands) that is available to enjoy, appreciate, or other opportunities (e.g., aesthetics, recreation, scenery)
Pollinators	bees, butterflies, or other animals (e.g., bats, birds) that distribute pollen for plants (e.g., flowers, flora, crops, farms, agriculture, gardens)
Presence of Environment	opportunities to enjoy interaction with nature (e.g., camping, hiking, swimming, trails, nature appreciation) or non-use value (e.g., existence, bequest); regulating services that purify/filter/buffer air or water pollutants; erosion or flood control; shoreline or natural hazard protection (e.g., wave attenuation); sound or temperature regulation (e.g., shading)
Soil	availability of soil (e.g., dirt, sediment) for farming, gardening, or other uses
Sounds/Scents	natural noises and smells available to hear and enjoy, including bird songs, croaking, chirping, rustling, splashing, thunder, ocean waves, flowers, or berries
Substrate	natural substrate (e.g., bedrock, sand, oyster reef, beaches) available as a surface or support for construction, renourishment, stabilization, or other uses
Timber	natural wood (e.g., timber, logs, lumber) for household, commercial, or industrial uses
Viewscapes	opportunities for scenic (e.g., beautiful, inspirational, spectacular) views (e.g., vista, landscape, overlook) or aesthetically/visually pleasing sights
Water	a resource for consumption (e.g., drinking), industry (e.g., cooling, hydroelectricity), households (e.g., rain barrels), agriculture (e.g., irrigation); a medium to receive & dilute discharges (e.g., wastewater, ballast), or for transportation (e.g., boat or ship navigation)
Weather	weather (e.g., climate, rain, temperature, snow, seasons, sun, fog) available to enjoy (e.g., for recreation, tourism, sunbathing) or favorable for activities (e.g., agriculture)
Wind	wind available to enjoy (e.g., boating, surfing, recreation), or as a resource for commercial or household uses (e.g., energy, electricity, power)
Unspecified	ecosystem services; natural resources; beneficial uses; living resources; renewable resources

^1^ Unique: e.g., charismatic, beautiful, special, biodiversity; ^2^ Enjoyed as a resource: e.g., observe, sightsee, hunt, harvest, gather, collect, consume, subsistence, learning; ^3^ Identified for conservation: e.g., protect, restore, conserve, preserve, endangered, threatened.

**Table 6:** List of the most common Beneficiary and Environment Classes associated with each type of FEGS, out of 1614 identified combinations, and the number of NEP and NERR plans that mentioned them.

FEGS Type	Beneficiary	Environment	No.	Example Phrase
Air	Government/Municipal/Residential	Atmosphere	16	“protecting the air our residents breathe”
Atmospheric Phenomena	Experiencers/Viewers	Estuaries/Near Coastal Marine	6	“gazing at stunning coastal sunsets”
Depredators/Pest Predators	Government/Municipal/Residential	Created Greenspace	3	“implement integrated pest management on public lands”
Fauna, Flora	People Who Care (Existence)	Estuaries/Near Coastal Marine	55, 56	“protect rare and endangered species in the estuary”
Fiber	Artists	Terrestrial	2	“fibers from the area used to temper pottery”
Fish	Food Extractors	Aquatic	57	“the waters provide shellfish for commercial fisheries”
Fungi	Experiencers/Viewers	Rivers and Streams	1	“collect mushrooms along the streambank”
Land	Government/Municipal/Residential	Terrestrial	53	“set up a public land trust”
Natural Materials	Industrial Processors	Barren Rock/Sand	19	“sand mining in the area”
Open Space	Government/Municipal/Residential	Terrestrial	31	“open spaces for public use”
Pollinators	Agricultural	Agroecosystems	2	“pollination of agricultural plants”
Presence of Environment	Educators/Students	Estuaries/Near Coastal Marine	57	“a natural lab for students to learn about the estuary”
Soil	Farmers	Agroecosystems	7	“rich agricultural soils preserved for farming”
Sounds/Scents	Experiencers/Viewers	Aquatic	6	“enjoy migratory songbirds near the water”
Substrate	People Who Care (Existence)	Estuaries/Near Coastal Marine	10	“protect and restore oyster reefs”
Timber	Timber/Fiber/Ornamental Extractors	Forests	27	“timber harvest from forests by logging companies”
Viewscapes	Experiencers/Viewers	Estuaries/Near Coastal Marine	46	“a panoramic view of the bay from the observation tower”
Water	Government/Municipal/Residential	Aquatic	57	“the community depends on natural systems for water resources”
Weather	Experiencers/Viewers	Estuaries/Near Coastal Marine	8	“visitors come to the shore to sunbathe”
Wind	Boaters	Aquatic	11	“sailing and windsurfing”

**Table 7:** Example phrases from the Lake Superior National Estuarine Research Reserve (LSNERR) associating beneficiaries of the presence of the environment with different types of ecosystems.

Beneficiary	Environment	Example Phrases
Anglers, Boaters, Hunters, Resource-Dependent Businesses, Recreators	Estuaries/Near Coastal Marine; Wetlands	“Freshwater estuaries and their associated coastal wetlands are locally important for activities such as hunting, fishing, boating and tourism”
Government/Municipal/Residential	Estuaries/Near Coastal Marine	“Freshwater estuaries are important components of their surrounding communities”; “Become a model for long-term community involvement and inter-governmental cooperation”
Commercial/Industrial	Rivers/Streams	“ongoing maintenance dredging and industrial and commercial activities still result in changes to the river”
Educators/Students; Experiencers/Viewers; Learning;	Forests; Created Greenspace	“the Superior Municipal Forest, with its extensive trail network, outdoor classroom, and other resources, will be an important part of LSNERR educational programming”
Experiencers/Viewers	Created Greenspace; Ice/Snow	“unpaved trail system includes... cross-country ski trails… snowmobiling, ATV riding, and skijoring (skiing with dogs)”
Inspirational	Estuaries/Near Coastal Marine	“cultural significance of the St. Louis River Freshwater Estuary”
Experiencers/Viewers; Inspirational; Recreational; Resource-Dependent Business;	Rivers/Streams; Lakes/Ponds	“visitors each year, drawn in large part by the beauty and natural amenities of the St. Louis River and Lake Superior, contribute … to the local economy”
People Who Care (Existence)	Forest; Rivers/Streams	“Streambank Protection Area… recognizes the value of the land for conservation, rather than timber production”
People Who Care (Option/Bequest)	Estuaries/Near Coastal Marine	“Sustainable use of the coastal environment”; “promote stewardship”
Researchers	Estuaries/Near Coastal Marine	“the reserve will provide opportunities for research and monitoring”; “long-term protection of the Reserve’s estuarine resources necessary to ensure a stable environment for research”

## Supplementary Materials

The following are available online at https://www.mdpi.com/1660-4601/16/13/2351/s1, Table S1: Keywords used to code types of FEGS, Beneficiaries, and Environments; Table S2: Counts of FEGS identified in NEP and NERR reviews.

## References

[1] Millennium Ecosystem Assessment (2005). Ecosystems and Human Well-Being: Synthesis.

[2] Arkema K.K., Verutes G.M., Wood S.A., Clarke-Samuels C., Rosado S., Canto M., Rosenthal A., Ruckelshaus M., Guannel G., Toft J. (2015). Embedding ecosystem services in coastal planning leads to better outcomes for people and nature. Proc. Natl. Acad. Sci. USA.

[3] Olander L., Johnson R.J., Tallis H., Kagan J., Maguire L., Polasky S., Urban D.L., Boyd J., Wainger L.A., Palmer M. (2015). Best Practices for Integrating Ecosystem Services into Federal Decision Making: National Ecosystems Partnership.

[4] Posner S.M., McKenzie E., Ricketts T.H. (2016). Policy impacts of ecosystem services knowledge. Proc. Nat. Acad. Sci. USA.

[5] Boyd J., Banzhaf S. (2007). What are ecosystem services? The need for standardized environmental accounting units. Ecol. Econ..

[6] US Code US Federal Water Pollution Control Act, 33 USC 1330 Section 320. http://uscode.house.gov/statviewer.htm?volume=101&page=61#.

[7] US Code Estuaries and Clean Waters Act of 2000. http://uscode.house.gov/statviewer.htm?volume=114&page=1972.

[8] US Code Coastal Zone Management Act of 1972, Amended 2005, *16 USC 1461 Section 315*. https://coast.noaa.gov/czm/act/sections/#315.

[9] Martin L. (2014). The use of ecosystem services information by the U.S. national estuary programs. Ecosyst. Serv..

[10] Guo J., Kildow J. (2015). The gap between science and policy: Assessing the use of nonmarket valuation in estuarine management based on a case study of US federally managed estuaries. Ocean Coast. Manag..

[11] Landers D., Nahlik A. (2013). Final Ecosystem Goods and Services Classification System (FEGS-CS). EPA/600/R-13/ORD-004914.

[12] United States Environmental Protection Agency (2015). National Ecosystem Services Classification System (NESCS): Framework Design and Policy Application. EPA-800-R-15-002.

[13] Boyd J.W., Ringold P.L., Krupnick A.J., Johnston R.J., Weber M., Hall K. (2015). Ecosystem Services Indicators: Improving the Linkage between Biophysical and Economic Analyses. RFF DP 15-40.

[14] Yee S., Bousquin J., Bruins R., Canfield T.J., DeWitt T.H., de Jesús-Crespo R., Dyson B., Fulford R., Harwell M., Hoffman J. (2017). Practical Strategies for Integrating Final Ecosystem Goods and Services into Community Decision-Making, EPA/600/R-17/266.

[15] Homer C., Huang C., Yang L., Wylie B., Coan M. (2004). Development of a 2001 National Landcover Database for the United States. Photogramm. Eng. Remote Sens..

[16] US Census Bureau (2017). North American Industry Classification System.

[17] The R Project for Statistical Computing. www.r-project.org.

[18] U.S. Environmental Protection Agency (2008). Handbook for Developing Watershed Plans to Restore and Protect Our Waters. EPA 841-B-08-002.

[19] Ohlson D.W., Serveiss V.B. (2007). The integration of ecological risk assessment and structured decision making into watershed management. Integr. Environ. Assess. Manag..

[20] Bradley P., Fisher W., Dyson B., Yee S., Carriger J., Gambirazzio G., Bousquin J., Huertas E. (2016). Application of a Structured Decision Process for Informing Watershed Management Options in Guánica Bay, Puerto Rico. EPA/600/R-15/248.

[21] Raudsepp-Hearne C., Peterson G.D., Bennett E.M. (2010). Ecosystem service bundles for analyzing tradeoffs in diverse landscapes. Proc. Natl. Acad. Sci. USA.

[22] Smith A., Yee S.H., Russell M., Awkerman J., Fisher W.S. (2017). Linking ecosystem service supply to stakeholder concerns on both land and sea: An example from Guánica Bay watershed, Puerto Rico. Ecol. Indic..

[23] Productivity Commission (2003). Industries, Land Use and Water Quality in the Great Barrier Reef Catchment, Research Report.

[24] Roebeling P.C. (2006). Efficiency in Great Barrier Reef water pollution control: A case study for the Douglas Shire. Nat. Resour. Model..

[25] National Academy of Sciences (2013). Environmental Decisions in the Face of Uncertainty.

[26] Lake Superior National Estuarine Research Reserve (2010). Lake Superior National Estuarine Research Reserve Management Plan 2010–2015.

[27] Superior Municipal Forest. https://www.ci.superior.wi.us/224/Superior-Municipal-Forest.

[28] Gregory R.L., Failing M., Harstone G., Long T., McDaniels D.O. (2012). Structured Decision-Making: A Practical Guide to Environmental Management Choices.

[29] Marcot B.G., Thompson M.P., Runge M.C., Thompson F.R., McNulty S., Cleaves D., Tomosy M., Fisher L.A., Bliss A. (2012). Recent advance in applying decision science to managing national forests. For. Ecol. Manag..

[30] Tallis H.T., Ricketts T., Guerry A.D., Wood S.A., Sharp R., Nelson E., Ennaanay D., Wolny S., Olwero N., Vigerstol K. (2013). InVEST 3.0.0 User’s Guide.

[31] Birol E., Karousakis K., Koundouri P. (2006). Using economic valuation techniques to inform water resources management: A survey and critical appraisal of available techniques and an application. Sci. Total Environ..

[32] Johnston R.J., Wainger L.A., Johnston R.J., Rolfe J., Rosenberger R.S., Brouwer R. (2015). Benefit Transfer for Ecosystem Service Valuation: An Introduction to Theory and Methods. Benefit Transfer of Environmental and Resource Values: A Guide for Researchers and Practitioners.

[33] Yoskowitz D.W., Werner S.R., Carollo C., Santos C., Washburn T., Isaksen G.H. (2016). Gulf of Mexico offshore ecosystem services: Relative valuation by stakeholders. Mar. Policy.

[34] Mazzotta M., Bousquin J., Berry W., Ojo C., McKinney R., Hyckha K., Druschke C.G. (2018). Evaluating the ecosystem services and benefits of wetland restoration using the Rapid Benefit Indicators Approach. Integr. Environ. Assess. Manag..

[35] Tashie A., Ringold P. (2019). A critical assessment of available ecosystem services data according to the Final Ecosystem Goods and Services framework. Ecosphere.

[36] Angradi T.R., Ringold P.L., Hall K. (2018). Water clarity measures as indicators of recreational benefits provided by U.S. lakes: Swimming and aesthetics. Ecol. Indic..

