# Supplementary material for: Who Benefits from National Estuaries? Applying the FEGS Classification System to Identify Ecosystem Services and their Beneficiaries
Source: Int J Environ Res Public Health. 2019 Jul 3;16(13):2351. doi: 10.3390/ijerph16132351 (PMC6651245; doi:10.3390/ijerph16132351)
Supplement: Supplementary file 1 [file ijerph-16-02351-s001.zip › S1_Synonym List.xlsx.pdf]

AppendixA_Synonym List.xlsx

This excel file contains the keywords used to flag sentences in documents.

**Category**	This is the Classification category from the FEGS Classification System
**Class**	This is the Class from the FEGS Classification System
**SubClass**	This is the Subclass from the FEGS Classification System
**Word_type**	Each subclass has associated keywords that are searched for, depending on the type of word. "Include" are words that must be included in a sentence for it to be flagged. "Near" words must also be included in the sentence and modify the 'include' words in some way. "Exclude" words cause the flagged sentence to be rejected.
**S1-S15**	These are sets of keywords that are searched (depending on the word type) to flag sentences in the document search. The symbol "|" indicates "OR". Within a column for a given subclass, particular "near", "include" and "exclude" words are considered in combination. Different columsn (S1, S2, etc.) may represent other keyword combinations that are also considered (i.e., the sentence is flagged if it contains the keywords in column S1 or S2 or S3, etc.)

Category	Class	SubClass	Word_type	S1
Beneficiary	Beneficiary	Unknown Beneficiary	include	resource|mitigat|economic use|economic opportunity|economic opportunities|bounty|bountiful|benefit|social value|economic value|valuable
Beneficiary	Beneficiary	Unknown Beneficiary	near	ecosystem|environment|natur|estuar|renewable|bay|ecological|field|aquatic|marine|water|park|coast|shore|land|wetland|the reserve|floodplain|biological|basin|region| area|watershed
Beneficiary	Beneficiary	Unknown Beneficiary	exclude	Department|sewer service|office|division|commission|bay scallop|lander|landing|landmark|southland|parking|parker|economical|protection agency|environmentally|temperature value|biomass value|the regional|provid.* no clue|provid.* notice|provid.* basis|provid.*expertise|valuable tool
Beneficiary	Agricultural	Agricultural	include	Agricultur
Beneficiary	Agricultural	Agricultural	exclude	department.*agricultur
Beneficiary	Agricultural	Irrigators	include	irrigat|watering
Beneficiary	Agricultural	CAFO Operators	include	CAFO|livestock.*production|animal production|animal.*feed.*operation|animal.*feed.*lot|feed.*lot.*animal|feed.*operation.*animal|confined.*animal.*production|confined.*animal.*operation
Beneficiary	Agricultural	CAFO Operators	near	
Beneficiary	Agricultural	CAFO Operators	exclude	reproduction
Beneficiary	Agricultural	Livestock Grazers	include	ranch|grazing|cattle.*range|graze|movement
Beneficiary	Agricultural	Livestock Grazers	near	livestock|cattle|hog|poultry|cows|pig|sheep|goat|duck
Beneficiary	Agricultural	Livestock Grazers	exclude	branch|actions|escuela|fennessey|rush.*ranch|quahog|pigment|pigeon|pathogen|hog hammock|sheepshead
Beneficiary	Agricultural	Agricultural Processo	include	processor|processing|production|products
Beneficiary	Agricultural	Agricultural Processo	near	agricultur|agro|farm|food|grain|meat|sugar|grain|vegetable|fruit
Beneficiary	Agricultural	Agricultural Processo	exclude	seafood|oyster|shellfish|fish|in the process
Beneficiary	Agricultural	Aquaculturists	include	aquacultur|aquafarm|shellfish garden|oyster garden|shellfish farm|oyster farm|fish farm|aqua-farm|aquatic farm
Beneficiary	Agricultural	Aquaculturists	near	
Beneficiary	Agricultural	Aquaculturists	exclude	
Beneficiary	Agricultural	Farmers	include	crops|orchard|farm|cropland|vineyard|crop nutrient
Beneficiary	Agricultural	Farmers	near	
Beneficiary	Agricultural	Farmers	exclude	farm shellfish|farm fish|farm oyster|farm clam|farm mussel|farm shrimp|farm shellfish|farmed fish|farmed oyster|farmed clam|farmed mussel|farmed shrimp|farming shellfish|farming fish|farming oyster|farming clam|farming mussel|farming shrimp|shellfish farm|fish farm|oyster farm|clam farm|mussel farm|shrimp farm|aqua farm|farm market|farmer.* market|management.*plan|service.*agency|nrcs|aquafarm|aquatic farm|farm bill|macrops|orchard grass|orchard oriole
Beneficiary	Agricultural	Foresters	include	forester|forestry|tree.*farm|silviculture
Beneficiary	Agricultural	Foresters	near	
Beneficiary	Agricultural	Foresters	exclude	braintree|Division of Parks & Forestry|Forestry Service|Forestry Association|Forestry Division|Division of|Department of|Forestry Agency|Forestry Assistance|Forestry council|Society of
Beneficiary	Commerical_Industri	Commerical_Industri	include	commercial|industry|industrial|business

Category	Class	SubClass	Word_type	S2	S3
Beneficiary	Beneficiary	Unknown Beneficiary	include	beneficial use|living resource|natural feature|natural resource|ecosystem service|ecological service|water resource|air resource|ecological resource|valuable resource|resource value	clean air|clean water|air quality|water quality
Beneficiary	Beneficiary	Unknown Beneficiary	near		
Beneficiary	Beneficiary	Unknown Beneficiary	exclude	department	recreational water quality|drinking water quality|clean water act|clean air act
Beneficiary	Agricultural	Agricultural	include		
Beneficiary	Agricultural	Agricultural	exclude		
Beneficiary	Agricultural	Irrigators	include		
Beneficiary	Agricultural	CAFO Operators	include	feed.*operation|feed.*lot|confined|production	livestock
Beneficiary	Agricultural	CAFO Operators	near	livestock|cattle|hog|poultry|cows|pig|chicken|sheep|goat|duck	manage
Beneficiary	Agricultural	CAFO Operators	exclude	migrat|wildlife|quahog|pigment|pigeon|pathogen|reproduction|hog hammock|sheepshead	
Beneficiary	Agricultural	Livestock Grazers	include	pasture	ranching
Beneficiary	Agricultural	Livestock Grazers	near		
Beneficiary	Agricultural	Livestock Grazers	exclude		branch|rush.*ranch
Beneficiary	Agricultural	Agricultural Processo	include	mill	commodity|commodities|goods
Beneficiary	Agricultural	Agricultural Processo	near	agricult|farm|grain|food	agricult|farm
Beneficiary	Agricultural	Agricultural Processo	exclude	million|millimeter|millennia|milliliter|millirem|milligram|miller|mill.*creek	
Beneficiary	Agricultural	Aquaculturists	include	hatchery|hatcheries|culturing|grower|culture|cultivat|garden	hatchery|hatcheries|culturing|grower|growing|culture|cultivat
Beneficiary	Agricultural	Aquaculturists	near	shellfish|fish|oyster|clam|scallop|mussel|shrimp|aquatic.*plant|meat|crab|salmon|oyster|pike|crab|lobster|mullet|mussel|bass|herring|grouper|snapper|alewife|flounder|abalone|scallop|clam|geoduck|crawfish|crayfish|crustacea	aqua
Beneficiary	Agricultural	Aquaculturists	exclude	fishing|fisher|agricultur|ambassador|spike|native culture|cultures	fishing|fisher|agricultur|ambassador|spike|native culture|cultures
Beneficiary	Agricultural	Farmers	include	plantation	bog|farm|harvest|grower|growing
Beneficiary	Agricultural	Farmers	near	sugar|coffee	rice|vegetable|cranberr
Beneficiary	Agricultural	Farmers	exclude	implantation	farm shellfish|farm fish|farm oyster|farm clam|farm mussel|farm shrimp|farm shellfish|farmed fish|farmed oyster|farmed clam|farmed mussel|farmed shrimp|farming shellfish|farming fish|farming oyster|farming clam|farming mussel|farming shrimp|shellfish farm|fish farm|oyster farm|clam farm|mussel farm|shrimp farm|aqua farm|farm market|farmer.* market|management.*plan|service.*agency|nrcs|aquafarm|aquatic farm|farm bill|macrops|orchard grass|orchard oriole|bog fern|bog moss|bog club|boge|Harvest brodiaea
Beneficiary	Agricultural	Foresters	include	plantation|farm|grower|growing	forest manage|forest indust
Beneficiary	Agricultural	Foresters	near	pine|wood|pulp|tree|timber|oak| elm|lumber|cedar|pine|oak|cypress| elm| ash|maple|hickory|hardwood	
Beneficiary	Agricultural	Foresters	exclude	transplant|braintree|implantation|cash|category|sewage|sanitary|acquatic|management.*area|fish|wash|dnr|mcbp|trash|shoreline|pineda|pinellas|legend|pine.*island|pine.*barren|vash|elmer|elm st|ashe|coal ash|mash|sash|cash|bash|overwhelm|oakdale|oakley|croak|coal.*ash|delmarva|fashion|pineland|barrier.*island|water.*pollution|soak|pine.*beach|over.*whelmed|barrier.*beach|ashore|sand.*pine|shorebird|percent|metals|oakland|silverside|hash|develm|hairstreak|crash|pine.*hills|ocean.*pine|oak.*harbor|oak.*ridge|pine.*seed|woodfern|sandalwoods|wood satyr|wood borer|wood nymph	
Beneficiary	Commerical_Industri	Commerical_Industri	include	commerce	

Category	Class	SubClass	Word_type	S4	S5
Beneficiary	Beneficiary	Unknown Beneficiary	include		
Beneficiary	Beneficiary	Unknown Beneficiary	near		
Beneficiary	Beneficiary	Unknown Beneficiary	exclude		
Beneficiary	Agricultural	Agricultural	include		
Beneficiary	Agricultural	Agricultural	exclude		
Beneficiary	Agricultural	Irrigators	include		
Beneficiary	Agricultural	CAFO Operators	include	feeding|feedlot|feed lot	operator|operation|farm
Beneficiary	Agricultural	CAFO Operators	near	cattle|livestock|cow|poultry|hog|pig|chicken|sheep|goat|duck	cattle|livestock|cow|poultry|hog|pig|chicken|sheep|goat|duck
Beneficiary	Agricultural	CAFO Operators	exclude	migrat|wildlife|quahog|pigment|pigeon|pathogen|hog hammock|sheepshead	power plant|aquacultur|marina|boat|watercraft|quahog|pigment|pigeon|pathogen|hog hammock|sheepshead
Beneficiary	Agricultural	Livestock Grazers	include		
Beneficiary	Agricultural	Livestock Grazers	near		
Beneficiary	Agricultural	Livestock Grazers	exclude		
Beneficiary	Agricultural	Agricultural Processo	include		
Beneficiary	Agricultural	Agricultural Processo	near		
Beneficiary	Agricultural	Agricultural Processo	exclude		
Beneficiary	Agricultural	Aquaculturists	include		
Beneficiary	Agricultural	Aquaculturists	near		
Beneficiary	Agricultural	Aquaculturists	exclude		
Beneficiary	Agricultural	Farmers	include		
Beneficiary	Agricultural	Farmers	near		
Beneficiary	Agricultural	Farmers	exclude		
Beneficiary	Agricultural	Foresters	include		
Beneficiary	Agricultural	Foresters	near		
Beneficiary	Agricultural	Foresters	exclude		
Beneficiary	Commerical_Industri	Commerical_Industri	include		

Category	Class	SubClass	Word_type	S6	S7
Beneficiary	Beneficiary	Unknown Beneficiary	include		
Beneficiary	Beneficiary	Unknown Beneficiary	near		
Beneficiary	Beneficiary	Unknown Beneficiary	exclude		
Beneficiary	Agricultural	Agricultural	include		
Beneficiary	Agricultural	Agricultural	exclude		
Beneficiary	Agricultural	Irrigators	include		
Beneficiary	Agricultural	CAFO Operators	include		
Beneficiary	Agricultural	CAFO Operators	near		
Beneficiary	Agricultural	CAFO Operators	exclude		
Beneficiary	Agricultural	Livestock Grazers	include		
Beneficiary	Agricultural	Livestock Grazers	near		
Beneficiary	Agricultural	Livestock Grazers	exclude		
Beneficiary	Agricultural	Agricultural Processo	include		
Beneficiary	Agricultural	Agricultural Processo	near		
Beneficiary	Agricultural	Agricultural Processo	exclude		
Beneficiary	Agricultural	Aquaculturists	include		
Beneficiary	Agricultural	Aquaculturists	near		
Beneficiary	Agricultural	Aquaculturists	exclude		
Beneficiary	Agricultural	Farmers	include		
Beneficiary	Agricultural	Farmers	near		
Beneficiary	Agricultural	Farmers	exclude		
Beneficiary	Agricultural	Foresters	include		
Beneficiary	Agricultural	Foresters	near		
Beneficiary	Agricultural	Foresters	exclude		
Beneficiary	Commerical_Industri	Commerical_Industri	include		

Category	Class	SubClass	Word_type	S8	S9
Beneficiary	Beneficiary	Unknown Beneficiary	include		
Beneficiary	Beneficiary	Unknown Beneficiary	near		
Beneficiary	Beneficiary	Unknown Beneficiary	exclude		
Beneficiary	Agricultural	Agricultural	include		
Beneficiary	Agricultural	Agricultural	exclude		
Beneficiary	Agricultural	Irrigators	include		
Beneficiary	Agricultural	CAFO Operators	include		
Beneficiary	Agricultural	CAFO Operators	near		
Beneficiary	Agricultural	CAFO Operators	exclude		
Beneficiary	Agricultural	Livestock Grazers	include		
Beneficiary	Agricultural	Livestock Grazers	near		
Beneficiary	Agricultural	Livestock Grazers	exclude		
Beneficiary	Agricultural	Agricultural Processo	include		
Beneficiary	Agricultural	Agricultural Processo	near		
Beneficiary	Agricultural	Agricultural Processo	exclude		
Beneficiary	Agricultural	Aquaculturists	include		
Beneficiary	Agricultural	Aquaculturists	near		
Beneficiary	Agricultural	Aquaculturists	exclude		
Beneficiary	Agricultural	Farmers	include		
Beneficiary	Agricultural	Farmers	near		
Beneficiary	Agricultural	Farmers	exclude		
Beneficiary	Agricultural	Foresters	include		
Beneficiary	Agricultural	Foresters	near		
Beneficiary	Agricultural	Foresters	exclude		
Beneficiary	Commerical_Industri	Commerical_Industri	include		

Category	Class	SubClass	Word_type	S10	S11
Beneficiary	Beneficiary	Unknown Beneficiary	include		
Beneficiary	Beneficiary	Unknown Beneficiary	near		
Beneficiary	Beneficiary	Unknown Beneficiary	exclude		
Beneficiary	Agricultural	Agricultural	include		
Beneficiary	Agricultural	Agricultural	exclude		
Beneficiary	Agricultural	Irrigators	include		
Beneficiary	Agricultural	CAFO Operators	include		
Beneficiary	Agricultural	CAFO Operators	near		
Beneficiary	Agricultural	CAFO Operators	exclude		
Beneficiary	Agricultural	Livestock Grazers	include		
Beneficiary	Agricultural	Livestock Grazers	near		
Beneficiary	Agricultural	Livestock Grazers	exclude		
Beneficiary	Agricultural	Agricultural Processo	include		
Beneficiary	Agricultural	Agricultural Processo	near		
Beneficiary	Agricultural	Agricultural Processo	exclude		
Beneficiary	Agricultural	Aquaculturists	include		
Beneficiary	Agricultural	Aquaculturists	near		
Beneficiary	Agricultural	Aquaculturists	exclude		
Beneficiary	Agricultural	Farmers	include		
Beneficiary	Agricultural	Farmers	near		
Beneficiary	Agricultural	Farmers	exclude		
Beneficiary	Agricultural	Foresters	include		
Beneficiary	Agricultural	Foresters	near		
Beneficiary	Agricultural	Foresters	exclude		
Beneficiary	Commerical_Industri	Commerical_Industri	include		

Category	Class	SubClass	Word_type	S12	S13
Beneficiary	Beneficiary	Unknown Beneficiary	include		
Beneficiary	Beneficiary	Unknown Beneficiary	near		
Beneficiary	Beneficiary	Unknown Beneficiary	exclude		
Beneficiary	Agricultural	Agricultural	include		
Beneficiary	Agricultural	Agricultural	exclude		
Beneficiary	Agricultural	Irrigators	include		
Beneficiary	Agricultural	CAFO Operators	include		
Beneficiary	Agricultural	CAFO Operators	near		
Beneficiary	Agricultural	CAFO Operators	exclude		
Beneficiary	Agricultural	Livestock Grazers	include		
Beneficiary	Agricultural	Livestock Grazers	near		
Beneficiary	Agricultural	Livestock Grazers	exclude		
Beneficiary	Agricultural	Agricultural Processo	include		
Beneficiary	Agricultural	Agricultural Processo	near		
Beneficiary	Agricultural	Agricultural Processo	exclude		
Beneficiary	Agricultural	Aquaculturists	include		
Beneficiary	Agricultural	Aquaculturists	near		
Beneficiary	Agricultural	Aquaculturists	exclude		
Beneficiary	Agricultural	Farmers	include		
Beneficiary	Agricultural	Farmers	near		
Beneficiary	Agricultural	Farmers	exclude		
Beneficiary	Agricultural	Foresters	include		
Beneficiary	Agricultural	Foresters	near		
Beneficiary	Agricultural	Foresters	exclude		
Beneficiary	Commerical_Industri	Commerical_Industri	include		

Category	Class	SubClass	Word_type	S1
Beneficiary	Commerical_Industri	Commerical_Industri	near	
Beneficiary	Commerical_Industri	Commerical_Industri	exclude	commercial fish|commercial shellfish|commercial and sport fish|commercial and recreational fish|commercial pilot|commercial vessel|commercial ship|commercia boat
Beneficiary	Commerical_Industri	Food Extractors	include	hunt.*meat|trap.*meat|meat.*hunt|meat.*trap|hunt.*edible|trap.*edible|edible.*hunt|edible.*trap|hunt.*consumpt|trap.*consumpt|consumpt.*hunt|consump t.*trap|hunt.*eaten|trap.*eaten|eaten.*hunt|eaten.*trap
Beneficiary	Commerical_Industri	Food Extractors	near	extract|commercial|business|product|industry|commodity|commodities|goods|harvest|profit|buy|livelihood|job|dealer|market|corporation|sale|sell|income|ar tisan|profession|trade|trading|worker
Beneficiary	Commerical_Industri	Food Extractors	exclude	riparian|buffer|vegetat|extrapolat|strap|entrap|strap|sediment.*trap|contam.*trap|pollut.*trap|trap.*contam|trap.*pollut|nutrient.*trap|trap.*nutrient|sand.*t rap|trap.*sand|trap.*sediment|litter trap|imping|trap.*sediment|sediment.*trap|contam.*trap|pollut.*trap|trap.*contam|trap.*pollut|nutrient.*trap|trap.*nutrient|sand.*trap|trap.*sand|litter trap|fish trap|association|shunt|huntley|huntington|hunterdon|workshop|salem|mysella|economus|Harvest brodiaea
Beneficiary	Commerical_Industri	Timber and Fiber and	include	pine|wood|pulp|tree|timber|oak| elm|lumber|cedar|pine|oak|cypress| elm| ash|maple|hickory|hardwood|cordwood|reed|grass|salt hay|marsh hay|turpentine
Beneficiary	Commerical_Industri	Timber and Fiber and	near	extract|commercial|business|product|industry|commodity|commodities|goods|harvest|profit|buy|livelihood|job|dealer|market|corporation|sale|sell|income|ar tisan|profession|trade|trading|worker
Beneficiary	Commerical_Industri	Timber and Fiber and	exclude	grassroot|grass-root|reproduct|productivity|marshfield|marshall|braintree|park|sea grass|freed|mcreedy|sea-grass|eelgrass|oyster.*grass|seagrass|grassland|sea-grass|decreed|breed|agreed|haynes|hay.*barn|hayden|cottonwood|freed|cottontail|reedgrass|hayan|woody|lakewood|kirkwood|woodpecker|woods hole|wood dock|wood preserv|woodneck|woodbury|wood stake|wooden|wood tick|woodland|wood turtle|tree frog|woodbridge|treated lumber|treated wood|wood stove|reproduct|cash|category|sewage|sanitary|acquatic|management.*area|fish|wash|dnr|mcbp|trash|shoreline|pineda|pinellas|legend|pine.*island|pine.*ba rren|vash|elmer|elm st|ashe|coal ash|mash|sash|cash|bash|overwhelm|oakdale|oakley|croak|coal.*ash|delmarva|fashion|pineland|barrier.*island|water.*pollution|soak|pine.*beach|over.*whel med|barrier.*beach|ashore|sand.*pine|shorebird|percent|metals|oakland|silverside|hash|develm|hairstreak|crash|pine.*hills|ocean.*pine|oak.*harbor|oak.*rid ge|pine.*seed|workshop|salem|mysella|economus|woodfern|sandalwoods|wood satyr|wood borer|wood nymph|Harvest brodiaea
Beneficiary	Commerical_Industri	Industrial Processors	include	industr.*process|process.*industr|commercial.*process|process.*commercial|industr.*product|product.*industr
Beneficiary	Commerical_Industri	Industrial Processors	near	
Beneficiary	Commerical_Industri	Industrial Processors	exclude	in the process|farm|agricultur|commercial fish|commercial shellfish|commercial and sport fish|commercial and recreational fish|commercial pilot|commercial vessel|commercial ship|commercial boat
Beneficiary	Commerical_Industri	Industrial Discharger	include	industr|factory|factories|manufactur|facility|facilities
Beneficiary	Commerical_Industri	Industrial Discharger	near	discharg|waste|effluent|leach
Beneficiary	Commerical_Industri	Industrial Discharger	exclude	sewage.*treatment|treatment.*sewage|wastewater.*treatment|treatment.*wastewater|action|department|satisfactor
Beneficiary	Commerical_Industri	Electric and other En	include	power.*plant|power line|powerline
Beneficiary	Commerical_Industri	Electric and other En	near	
Beneficiary	Commerical_Industri	Electric and other En	exclude	office.*of|department|program|degrade.*habitat|electric eel|electric ray
Beneficiary	Commerical_Industri	Resource-Dependent	include	marina|tourism|resort|hotel|shop|restaurant|landscaper|gardener|landscaping|landscape industry
Beneficiary	Commerical_Industri	Resource-Dependent	near	

Category	Class	SubClass	Word_type	S2	S3
Beneficiary	Commerical_Industri	Commerical_Industri	near		
Beneficiary	Commerical_Industri	Commerical_Industri	exclude	secretary|department|chamber|noaa	
Beneficiary	Commerical_Industri	Food Extractors	include	fishing|shrimping|clamming|fisher	fisheries|fishery
Beneficiary	Commerical_Industri	Food Extractors	near	extract|commercial|business|product|industry|commodity|commodities|goods| harvest|profit|buy|livelihood|job|dealer|market|corporation|sale|sell|income|ar<br>tisan|profession|trade|trading|worker	
Beneficiary	Commerical_Industri	Food Extractors	exclude	kingfisher|workshop|salem|mysella|economus|newmarket|fort fisher|Harvest<br>brodiaea|Harvest brodiaea	
Beneficiary	Commerical_Industri	Timber and Fiber and	include	logging|logger|timber	shells|seahorse|aquarium|aquaria|ornamental|pet store|aquatic plant
Beneficiary	Commerical_Industri	Timber and Fiber and	near		extract|commercial|business|product|industry|commodity|commodities|goods| harvest|profit|buy|livelihood|job|dealer|market|corporation|sale|sell|income|ar<br>tisan|profession|trade|trading|worker
Beneficiary	Commerical_Industri	Timber and Fiber and	exclude	temperature.*log|log.*temperature|clogging|loggerhead|data<br>logg|datalogg|timber<br>snake|clogging|woody|invasive|nonnative|non.*native|manage|plan|goal|legisla<br>t|fish|threat.*watershed|watershed.*threat|threat.*habitat|habitat.*threat|har<br>mful|marine|http|sewage|sanitary|acquatic|plastic|management.*area|runoff|st<br>ormwater|dredg|greek|ocean|sea|coast|septic|university|mcbp|landed|landing| landmark|council|forestry.*service|forestablish|shoreline|geologic|forest.*stewar<br>dship|lakewood|aquatic.*structure|survey|perservativ|woodneck|woods.*hole|u<br>sda|forest.*service|kirkwood|toxic|toxin|barrier.*island|water.*pollution|beach<br>wood|treated.*timber|treated.*wood|hollywood|wood.*neck|barrier.*beach|sh<br>orebird|percent|metals|brentwood|silverside|loblolly|contamin|department.*of.<br>*forest|psu|project.*park.*water|forest.*comittee|baywood|aquatic.*inventory| wood.*stove|legend|habitat.*conversion.*loss.*forest.*cover|kelp.*forest|table|f<br>igure|characterized.*as|forest.*learning.*shelter|vincent|cdmo|unnaceptable|15.<br>*visitor|wooden.*marco|litter.*retard|treated lumber|rattle|snake|data|timber<br>creek|timberneck|timber support|joist|joe timberland	eggshell|shellstock|workshop|productivity|reproduct|salem|mysella|economus| Harvest brodiaea
Beneficiary	Commerical_Industri	Industrial Processors	include	manufact|textile|steel|sawmill|factory|factories|canneries|cannery|papermill|pa<br>per mill	mill
Beneficiary	Commerical_Industri	Industrial Processors	near		industr|business|product|commercial|company
Beneficiary	Commerical_Industri	Industrial Processors	exclude	steelhead|instruction|sawmill pond|satisfactor|geotext	reproduct|million|millimeter|millennia|milliliter|millirem|milligram|miller|mill.*cr<br>eek|agricult|farm|grain|food
Beneficiary	Commerical_Industri	Industrial Discharger	include	landfill|land fill	effluent
Beneficiary	Commerical_Industri	Industrial Discharger	near		
Beneficiary	Commerical_Industri	Industrial Discharger	exclude		
Beneficiary	Commerical_Industri	Electric and other En	include	solar.*power|solar.*electric|solar.*energy|energy.*solar|electric.*solar|power.*s<br>olar|turbine	wind.*power|wind.*electric|wind.*energy|energy.*wind|electric.*wind|power.*<br>wind
Beneficiary	Commerical_Industri	Electric and other En	near		
Beneficiary	Commerical_Industri	Electric and other En	exclude		storm energy|wave energy
Beneficiary	Commerical_Industri	Resource-Dependent	include	resource	waterfront
Beneficiary	Commerical_Industri	Resource-Dependent	near	business|industry|dependent|occupation|extraction	working|develop

Category	Class	SubClass	Word_type	S4	S5
Beneficiary	Commerical_Industri	Commerical_Industri	near		
Beneficiary	Commerical_Industri	Commerical_Industri	exclude		
Beneficiary	Commerical_Industri	Food Extractors	include	shellfish|fish|oyster|clam|scallop|mussel|shrimp|meat|crab|salmon|oyster|pike|crab|lobster|mullet|mussel|bass|herring|grouper|snapper|alewife|flounder|abalone|scallop|clam|geoduck|crawfish|crayfish|crustacea|seafood	deer|turkey|quail|waterfowl|duck|goose|geese
Beneficiary	Commerical_Industri	Food Extractors	near	extract|commercial|business|product|industry|commodity|commodities|goods|harvest|profit|buy|livelihood|job|dealer|market|corporation|sale|sell|income|artisan|profession|trade|trading|worker	extract|commercial|business|product|industry|commodity|commodities|goods|harvest|profit|buy|livelihood|job|dealer|market|corporation|sale|sell|income|artisan|profession|trade|trading|worker
Beneficiary	Commerical_Industri	Food Extractors	exclude	productivity|production|workshop|ambassador|salmon falls|salem|mysella|economus|newmarket|oyster creek|clami|spike|Harvest brodiaea	productivity|production|workshop|duck farm|salem|mysella|economus|Harvest brodiaea
Beneficiary	Commerical_Industri	Timber and Fiber and	include	shell mining	firewood
Beneficiary	Commerical_Industri	Timber and Fiber and	near		extract|commercial|business|product|industry|commodity|commodities|goods|harvest|profit|buy|livelihood|job|dealer|market|corporation|sale|sell|income|artisan|profession|trade|trading|worker
Beneficiary	Commerical_Industri	Timber and Fiber and	exclude		workshop|productivity|reproduct|salem|mysella|economus
Beneficiary	Commerical_Industri	Industrial Processors	include	chemical|iron|metal|oil|gas|petro|lime	mining|mine|quarry
Beneficiary	Commerical_Industri	Industrial Processors	near	industr|factory|factories|manufact|foundry|foundries|operation|refiner|facilit	
Beneficiary	Commerical_Industri	Industrial Processors	exclude	viron|soil|slime|blime|clime|elime|plime	data.*min|determining|examining|determine|examine|promine|mineral|flumine a|shells|minen|mineu|amine|plaquemine|ermin|minello|deter-min|deter-min|minea|post-min|division|omine|smine|reclaim.*min|min.*reclaim|min.*reclam|reclam.*min|under- min|lettuce
Beneficiary	Commerical_Industri	Industrial Discharger	include		
Beneficiary	Commerical_Industri	Industrial Discharger	near		
Beneficiary	Commerical_Industri	Industrial Discharger	exclude		
Beneficiary	Commerical_Industri	Electric and other En	include	hydro.*electric|dam.*electric|electric.*dam|dam.*energy|energy.*dam	renewable.*energy|renewable.*power|renewable.*electric|energy.*renewable|power.*renewable|electric.*renewable
Beneficiary	Commerical_Industri	Electric and other En	near		
Beneficiary	Commerical_Industri	Electric and other En	exclude	electric eel|electric ray|storm energy|wave energy	
Beneficiary	Commerical_Industri	Resource-Dependent	include	outfitter|tour.*boat|charter.*boat	fish|hunt|tour|recreat|trip
Beneficiary	Commerical_Industri	Resource-Dependent	near		charter|guide

Category	Class	SubClass	Word_type	S6	S7
Beneficiary	Commerical_Industri	Commerical_Industri	near		
Beneficiary	Commerical_Industri	Commerical_Industri	exclude		
Beneficiary	Commerical_Industri	Food Extractors	include	food.*eat|eat.*food|food source	plant foods|food supply
Beneficiary	Commerical_Industri	Food Extractors	near	human| we |people	
Beneficiary	Commerical_Industri	Food Extractors	exclude		
Beneficiary	Commerical_Industri	Timber and Fiber and	include		
Beneficiary	Commerical_Industri	Timber and Fiber and	near		
Beneficiary	Commerical_Industri	Timber and Fiber and	exclude		
Beneficiary	Commerical_Industri	Industrial Processors	include	process	
Beneficiary	Commerical_Industri	Industrial Processors	near	seafood|fish|shellfish	
Beneficiary	Commerical_Industri	Industrial Processors	exclude	meat|sugar|grain|vegetable|fruit|permit|eutrophication|degradation|review|bay view	
Beneficiary	Commerical_Industri	Industrial Discharger	include		
Beneficiary	Commerical_Industri	Industrial Discharger	near		
Beneficiary	Commerical_Industri	Industrial Discharger	exclude		
Beneficiary	Commerical_Industri	Electric and other En	include	utilities	energy|power|electric
Beneficiary	Commerical_Industri	Electric and other En	near		production|industr|generat|suppl|station
Beneficiary	Commerical_Industri	Electric and other En	exclude	water.*utilities	storm energy|wave energy|powerful|food suppl
Beneficiary	Commerical_Industri	Resource-Dependent	include	business|industr|econom	bait
Beneficiary	Commerical_Industri	Resource-Dependent	near	local	commercial|business|industry|commodity|commodities|goods|profit|buy|livelihood|job|dealer|market|corporation|sale|sell|income|artisan|profession|trade|trading|worker|fishery|fisheries

Category	Class	SubClass	Word_type	S8	S9
Beneficiary	Commerical_Industri	Commerical_Industri	near		
Beneficiary	Commerical_Industri	Commerical_Industri	exclude		
Beneficiary	Commerical_Industri	Food Extractors	include		
Beneficiary	Commerical_Industri	Food Extractors	near		
Beneficiary	Commerical_Industri	Food Extractors	exclude		
Beneficiary	Commerical_Industri	Timber and Fiber anc	include		
Beneficiary	Commerical_Industri	Timber and Fiber anc	near		
Beneficiary	Commerical_Industri	Timber and Fiber anc	exclude		
Beneficiary	Commerical_Industri	Industrial Processors	include		
Beneficiary	Commerical_Industri	Industrial Processors	near		
Beneficiary	Commerical_Industri	Industrial Processors	exclude		
Beneficiary	Commerical_Industri	Industrial Discharger	include		
Beneficiary	Commerical_Industri	Industrial Discharger	near		
Beneficiary	Commerical_Industri	Industrial Discharger	exclude		
Beneficiary	Commerical_Industri	Electric and other En	include	nuclear.*energy|nuclear.*power|nuclear.*electric|energy.*nuclear|power.*nuclea r|electric.*nuclear	
Beneficiary	Commerical_Industri	Electric and other En	near		
Beneficiary	Commerical_Industri	Electric and other En	exclude		
Beneficiary	Commerical_Industri	Resource-Dependent	include	marine-related business	artisan
Beneficiary	Commerical_Industri	Resource-Dependent	near		

Category	Class	SubClass	Word_type	S10	S11
Beneficiary	Commerical_Industri	Commerical_Industri	near		
Beneficiary	Commerical_Industri	Commerical_Industri	exclude		
Beneficiary	Commerical_Industri	Food Extractors	include		
Beneficiary	Commerical_Industri	Food Extractors	near		
Beneficiary	Commerical_Industri	Food Extractors	exclude		
Beneficiary	Commerical_Industri	Timber and Fiber and	include		
Beneficiary	Commerical_Industri	Timber and Fiber and	near		
Beneficiary	Commerical_Industri	Timber and Fiber and	exclude		
Beneficiary	Commerical_Industri	Industrial Processors	include		
Beneficiary	Commerical_Industri	Industrial Processors	near		
Beneficiary	Commerical_Industri	Industrial Processors	exclude		
Beneficiary	Commerical_Industri	Industrial Discharger	include		
Beneficiary	Commerical_Industri	Industrial Discharger	near		
Beneficiary	Commerical_Industri	Industrial Discharger	exclude		
Beneficiary	Commerical_Industri	Electric and other En	include		
Beneficiary	Commerical_Industri	Electric and other En	near		
Beneficiary	Commerical_Industri	Electric and other En	exclude		
Beneficiary	Commerical_Industri	Resource-Dependent	include		
Beneficiary	Commerical_Industri	Resource-Dependent	near		

Category	Class	SubClass	Word_type	S12	S13
Beneficiary	Commerical_Industri	Commerical_Industri	near		
Beneficiary	Commerical_Industri	Commerical_Industri	exclude		
Beneficiary	Commerical_Industri	Food Extractors	include		
Beneficiary	Commerical_Industri	Food Extractors	near		
Beneficiary	Commerical_Industri	Food Extractors	exclude		
Beneficiary	Commerical_Industri	Timber and Fiber and	include		
Beneficiary	Commerical_Industri	Timber and Fiber and	near		
Beneficiary	Commerical_Industri	Timber and Fiber and	exclude		
Beneficiary	Commerical_Industri	Industrial Processors	include		
Beneficiary	Commerical_Industri	Industrial Processors	near		
Beneficiary	Commerical_Industri	Industrial Processors	exclude		
Beneficiary	Commerical_Industri	Industrial Discharger	include		
Beneficiary	Commerical_Industri	Industrial Discharger	near		
Beneficiary	Commerical_Industri	Industrial Discharger	exclude		
Beneficiary	Commerical_Industri	Electric and other En	include		
Beneficiary	Commerical_Industri	Electric and other En	near		
Beneficiary	Commerical_Industri	Electric and other En	exclude		
Beneficiary	Commerical_Industri	Resource-Dependent	include		
Beneficiary	Commerical_Industri	Resource-Dependent	near		

Category	Class	SubClass	Word_type	S1
Beneficiary	Commerical_Industri	Resource-Dependent	exclude	coast.*guard|zostera|marina.*guidebook|vistamar.*marina|recreational.*boating|negative|workshop|first resort|last resort|resort to|grasshop|zostera|bishop
Beneficiary	Commerical_Industri	Pharmaceutical and I	include	pharmaceutical|medicin|vitamin|drug|medical|surgical
Beneficiary	Commerical_Industri	Pharmaceutical and I	near	species|discovery|research|harvest|development|testing|purpose
Beneficiary	Commerical_Industri	Pharmaceutical and I	exclude	
Beneficiary	Commerical_Industri	Fur_Hide Trappers ar	include	skin|hide| fur | furs |fur-bear|fur bear|furbear|pelt|tanning|tannery|tanneries
Beneficiary	Commerical_Industri	Fur_Hide Trappers ar	near	extract|commercial|business|product|industry|commodity|commodities|goods|harvest|profit|buy|livelihood|job|dealer|market|corporation|sale|sell|income|ar tisan|profession|trade|trading|worker
Beneficiary	Commerical_Industri	Fur_Hide Trappers ar	exclude	reproduct|askin|pickerel|sking|salem|mysella|economus|newmarket|sulfur|peltandra|pelto|apelt|peltu|pelta|pelte|provid.* no clue|provid.* notice|provid.* basis|provid.*expertise|workshop|skink|skin lesion|skin condition|furc|iskin|refur|pelti
Beneficiary	Government and Mu	Government and Mu	include	government|municipal|village|county|infrastructur|property| town| city|public access|the public|public use|urban|rural|public good|public purpose
Beneficiary	Government and Mu	Government and Mu	near	
Beneficiary	Government and Mu	Government and Mu	exclude	legend|turban|capacity|publicize|publicity|publication|townsend
Beneficiary	Government and Mu	Municipal Drinking W	include	drink.*water|water.*supply|water.*drink|supply.*water
Beneficiary	Government and Mu	Municipal Drinking W	exclude	watershed|wastewater|permit|division.*water|waterway|waterbod
Beneficiary	Government and Mu	Wastewater Treatme	include	water.*treatment|treatment.*water|sewage.*treatment|treatment.*sewage|treatment.*plant
Beneficiary	Government and Mu	Wastewater Treatme	exclude	watershed|wastewater|permit|division.*water|waterway|waterbod
Beneficiary	Government and Mu	Residential Property	include	resident.*develop|develop.*resident|homeowner|home owner|private.*land|private.*property|landowner|land owner|private owner
Beneficiary	Government and Mu	Residential Property	near	
Beneficiary	Government and Mu	Residential Property	exclude	president|acqui|restor|purchase|buy|resident.*species|resident.*wildlife|resident.*animal|property.*tax|tax.*property|tax.*resident|resident.*tax|developing.* ecommendation|island
Beneficiary	Government and Mu	Military_Coast Guarc	include	military|coast.*guard|navy|army|marines|air force
Beneficiary	Government and Mu	Military_Coast Guarc	exclude	marinesanc|marineserv|marinespa
Beneficiary	Commercial_Military	Commercial_Military	include	commercial.*transport|transport.*commercial|military.*transport|transport.*military
Beneficiary	Commercial_Military	Commercial_Military	near	
Beneficiary	Commercial_Military	Commercial_Military	exclude	sediment.*transport|transport.*sediment|contam.*transport|transport.*contam|nutrient.*transport|transport.*nutrient|cargo|goods|shipping|freight|commodit |container|people|commut
Beneficiary	Commercial_Military	Transporters of Gooc	include	goods.*transport|transport.*goods|shipping|shipped|cargo|freight|bulk.*commodit
Beneficiary	Commercial_Military	Transporters of Gooc	near	

Category	Class	SubClass	Word_type	S2	S3
Beneficiary	Commerical_Industri	Resource-Dependent	exclude	independent|interdependent	haddock
Beneficiary	Commerical_Industri	Pharmaceutical and I	include	supplement	biotechnology|bio-technology
Beneficiary	Commerical_Industri	Pharmaceutical and I	near	food|nutrition|nutrient	
Beneficiary	Commerical_Industri	Pharmaceutical and I	exclude	water.*supplement|supplement.*water|steelhead.*supplement|technical supplement|supplemental|supplemented	
Beneficiary	Commerical_Industri	Fur_Hide Trappers ar	include	nutria|beaver|deer|alligator|hogs|rabbit	hunt|trap
Beneficiary	Commerical_Industri	Fur_Hide Trappers ar	near	extract|commercial|business|product|industry|commodity|commodities|goods|harvest|profit|buy|livelihood|job|dealer|market|corporation|sale|sell|income|artisan|profession|trade|trading|worker	extract|commercial|business|product|industry|commodity|commodities|goods|harvest|profit|buy|livelihood|job|dealer|market|corporation|sale|sell|income|artisan|profession|trade|trading|worker
Beneficiary	Commerical_Industri	Fur_Hide Trappers ar	exclude	reproduct|deerfield|productiv|harvest.*crop|crop.*harvest|harvest data|production|workshop|salem|mysella|economus	reproduct|meat|sport|recreat|season|opportunit|edible|consumpt|eaten|riparian|buffer|vegetat|extrapolat|strap|entrap|strap|sediment.*trap|contam.*trap|pollut.*trap|trap.*contam|trap.*pollut|nutrient.*trap|trap.*nutrient|sand.*trap|trap.*sand|trap.*sediment|litter trap|fish trap|trap.*site|shunt|huntley|huntington|hunterdon|workshop|salem|mysella|economus|newmarket|productivity
Beneficiary	Government and Mu	Government and Mu	include	our communit|sustainable communit|local communit|waterfront communit|community plan|town|city|neighborhood|community outreach|community education	resident|families|children|citizen|family living
Beneficiary	Government and Mu	Government and Mu	near		
Beneficiary	Government and Mu	Government and Mu	exclude	presidential|biological.*communit|benthic.*communit|ocean.*city|residential.*land|habitat.*community|natural.*communities|resident.*shorebird|coral.*communit|capacity|plant.*communit|communit.*plant|ecologic.*communit|vegetat.*communit|animal.*communit|bottom.*communit|biotic.*communit|estuarine.*communit|oyster.*communit|communit.*type|meadow.*communit|tidal.*communit|communit.*native|native.*commut|communit.*fish|fish.*communit|wildlife.*communit|resident.*pod|population.*resident|reef.*communit|forest.*communit|marine.*communit|capacity	resident.*develop|develop.*resident|homeowner|home owner|private.*land|private.*property|landowner|land owner|president|acqui|restor|purchase|buy|resident.*species|resident.*wildlife|resident.*animal|property.*tax|tax.*property|tax.*resident|resident.*tax|developing.*recommendation
Beneficiary	Government and Mu	Municipal Drinking W	include	municipal well|public well	desal plant|desalination
Beneficiary	Government and Mu	Municipal Drinking W	exclude		
Beneficiary	Government and Mu	Wastewater Treatme	include		
Beneficiary	Government and Mu	Wastewater Treatme	exclude		
Beneficiary	Government and Mu	Residential Property	include	homes|homeowner|housing|home-owner|home owner	residents|residential|private well|domestic well
Beneficiary	Government and Mu	Residential Property	near		
Beneficiary	Government and Mu	Residential Property	exclude	visitor housing|housing a |housing the	
Beneficiary	Government and Mu	Military_Coast Guard	include		
Beneficiary	Government and Mu	Military_Coast Guard	exclude		
Beneficiary	Commercial_Military	Commercial_Military	include	ship|vessel|air|rail|pilot|captain|navigation|train|navigability	highway|interstate|road|transport.*infrastruct|infrastruct.*transport
Beneficiary	Commercial_Military	Commercial_Military	near	transport|commercial	develop|alter|construct|site|siting|abandon|assess|remov|need|build
Beneficiary	Commercial_Military	Commercial_Military	exclude	relationship|rship|nship|urship|sediment.*transport|transport.*sediment|contam.*transport|transport.*contam|nutrient.*transport|transport.*nutrient|cargo|goods|shipping|freight|commodit|container|people|commut|trail|pair|fair|air yam|air plant|chair|questionnaire|airborne|affair|millionaire|dairy|airshed|National.*Weather.*Service|impair|repair|belleair|air.*act|prair|clean.*air.*act|national.*air.*standard|regulat.*air|lair|Air Resource Specialist|Clean Air Bond|dair|www|Air Resources Lab|air rifle|air condition|airboat|stair|nair|bair|fair|air yam|air plant|air temp|hair	broad|nroad|roadbl|sediment.*transport|transport.*sediment|contam.*transport|transport.*contam|nutrient.*transport|transport.*nutrient
Beneficiary	Commercial_Military	Transporters of Good	include	port	vessel|barge|gundalow
Beneficiary	Commercial_Military	Transporters of Good	near	cargo|goods|shipping|freight|commodit|container	cargo|goods|shipping|freight|commodit|container

Category	Class	SubClass	Word_type	S4	S5
Beneficiary	Commerical_Industri	Resource-Dependent	exclude		
Beneficiary	Commerical_Industri	Pharmaceutical and I	include		
Beneficiary	Commerical_Industri	Pharmaceutical and I	near		
Beneficiary	Commerical_Industri	Pharmaceutical and I	exclude		
Beneficiary	Commerical_Industri	Fur_Hide Trappers ar	include		
Beneficiary	Commerical_Industri	Fur_Hide Trappers ar	near		
Beneficiary	Commerical_Industri	Fur_Hide Trappers ar	exclude		
Beneficiary	Government and Mu	Government and Mu	include	hazard|natural disaster|aquifer|development.*pattern|pattern.*development|coastal development|sprawl	open space
Beneficiary	Government and Mu	Government and Mu	near		
Beneficiary	Government and Mu	Government and Mu	exclude	hazardous	
Beneficiary	Government and Mu	Municipal Drinking W	include	municipal.*water|water.*municipal	
Beneficiary	Government and Mu	Municipal Drinking W	exclude	watershed|wastewater|permit|division.*water|waterway|waterbod|municipal park|municipalit	
Beneficiary	Government and Mu	Wastewater Treatme	include		
Beneficiary	Government and Mu	Wastewater Treatme	exclude		
Beneficiary	Government and Mu	Residential Property	include		
Beneficiary	Government and Mu	Residential Property	near		
Beneficiary	Government and Mu	Residential Property	exclude		
Beneficiary	Government and Mu	Military_Coast Guard	include		
Beneficiary	Government and Mu	Military_Coast Guard	exclude		
Beneficiary	Commercial_Military	Commercial_Military	include	navigable|navigat	
Beneficiary	Commercial_Military	Commercial_Military	near		
Beneficiary	Commercial_Military	Commercial_Military	exclude		
Beneficiary	Commercial_Military	Transporters of Good	include	train|railroad|railway	rail|air|transport
Beneficiary	Commercial_Military	Transporters of Good	near	cargo|goods|shipping|freight|commodit|container	cargo|goods|shipping|freight|commodit|container

Category	Class	SubClass	Word_type	S6	S7
Beneficiary	Commerical_Industri	Resource-Dependent	exclude		workshop|productivity|reproduct|salem|mysella|economus
Beneficiary	Commerical_Industri	Pharmaceutical and I	include		
Beneficiary	Commerical_Industri	Pharmaceutical and I	near		
Beneficiary	Commerical_Industri	Pharmaceutical and I	exclude		
Beneficiary	Commerical_Industri	Fur_Hide Trappers ar	include		
Beneficiary	Commerical_Industri	Fur_Hide Trappers ar	near		
Beneficiary	Commerical_Industri	Fur_Hide Trappers ar	exclude		
Beneficiary	Government and Mu	Government and Mu	include	watershed| area|region|community|communities|country|state	our region|our country|our county|our state|our area|our watershed|the people of|all the people|all of the people|of the people
Beneficiary	Government and Mu	Government and Mu	near	habitant|resident|occupant|living in|live in	
Beneficiary	Government and Mu	Government and Mu	exclude	presidential|biological.*communit|benthic.*communit|ocean.*city|residential.*land|habitat.*community|natural.*communities|resident.*shorebird|coral.*communit|capacity|plant.*communit|communit.*plant|ecologic.*communit|vegetat.*communit|animal.*communit|bottom.*communit|biotic.*communit|estuarine.*communit|oyster.*communit|communit.*type|meadow.*communit|tidal.*communit|communit.*native|native.*commut|communit.*fish|fish.*communit|wildlife.*communit|resident.*pod|population.*resident|reef.*communit|forest.*communit|marine.*communit	
Beneficiary	Government and Mu	Municipal Drinking W	include		
Beneficiary	Government and Mu	Municipal Drinking W	exclude		
Beneficiary	Government and Mu	Wastewater Treatme	include		
Beneficiary	Government and Mu	Wastewater Treatme	exclude		
Beneficiary	Government and Mu	Residential Property	include		
Beneficiary	Government and Mu	Residential Property	near		
Beneficiary	Government and Mu	Residential Property	exclude		
Beneficiary	Government and Mu	Military_Coast Guard	include		
Beneficiary	Government and Mu	Military_Coast Guard	exclude		
Beneficiary	Commercial_Military	Commercial_Military	include		
Beneficiary	Commercial_Military	Commercial_Military	near		
Beneficiary	Commercial_Military	Commercial_Military	exclude		
Beneficiary	Commercial_Military	Transporters of Good	include		
Beneficiary	Commercial_Military	Transporters of Good	near		

Category	Class	SubClass	Word_type	S8	S9
Beneficiary	Commerical_Industri	Resource-Dependent	exclude		
Beneficiary	Commerical_Industri	Pharmaceutical and I	include		
Beneficiary	Commerical_Industri	Pharmaceutical and I	near		
Beneficiary	Commerical_Industri	Pharmaceutical and I	exclude		
Beneficiary	Commerical_Industri	Fur_Hide Trappers a	include		
Beneficiary	Commerical_Industri	Fur_Hide Trappers a	near		
Beneficiary	Commerical_Industri	Fur_Hide Trappers a	exclude		
Beneficiary	Government and Mu	Government and Mu	include	quality of life|public health|human health|wellbeing|well being|well-being|general health|general welfare	safe
Beneficiary	Government and Mu	Government and Mu	near	habitant|resident|occupant|living in|live in	habitant|resident|occupant|living in|live in
Beneficiary	Government and Mu	Government and Mu	exclude	department of public health|public health department	boat|safeguard|pilot|harbor|port|vessel|ship
Beneficiary	Government and Mu	Municipal Drinking W	include		
Beneficiary	Government and Mu	Municipal Drinking W	exclude		
Beneficiary	Government and Mu	Wastewater Treatme	include		
Beneficiary	Government and Mu	Wastewater Treatme	exclude		
Beneficiary	Government and Mu	Residential Property	include		
Beneficiary	Government and Mu	Residential Property	near		
Beneficiary	Government and Mu	Residential Property	exclude		
Beneficiary	Government and Mu	Military_Coast Guard	include		
Beneficiary	Government and Mu	Military_Coast Guard	exclude		
Beneficiary	Commercial_Military	Commercial_Military	include		
Beneficiary	Commercial_Military	Commercial_Military	near		
Beneficiary	Commercial_Military	Commercial_Military	exclude		
Beneficiary	Commercial_Military	Transporters of Good	include		
Beneficiary	Commercial_Military	Transporters of Good	near		

Category	Class	SubClass	Word_type	S10	S11
Beneficiary	Commerical_Industri	Resource-Dependent	exclude		
Beneficiary	Commerical_Industri	Pharmaceutical and I	include		
Beneficiary	Commerical_Industri	Pharmaceutical and I	near		
Beneficiary	Commerical_Industri	Pharmaceutical and I	exclude		
Beneficiary	Commerical_Industri	Fur_Hide Trappers ar	include		
Beneficiary	Commerical_Industri	Fur_Hide Trappers ar	near		
Beneficiary	Commerical_Industri	Fur_Hide Trappers ar	exclude		
Beneficiary	Government and Mu	Government and Mu	include		
Beneficiary	Government and Mu	Government and Mu	near		
Beneficiary	Government and Mu	Government and Mu	exclude		
Beneficiary	Government and Mu	Municipal Drinking W	include		
Beneficiary	Government and Mu	Municipal Drinking W	exclude		
Beneficiary	Government and Mu	Wastewater Treatme	include		
Beneficiary	Government and Mu	Wastewater Treatme	exclude		
Beneficiary	Government and Mu	Residential Property	include		
Beneficiary	Government and Mu	Residential Property	near		
Beneficiary	Government and Mu	Residential Property	exclude		
Beneficiary	Government and Mu	Military_Coast Guarc	include		
Beneficiary	Government and Mu	Military_Coast Guarc	exclude		
Beneficiary	Commercial_Military	Commercial_Military	include		
Beneficiary	Commercial_Military	Commercial_Military	near		
Beneficiary	Commercial_Military	Commercial_Military	exclude		
Beneficiary	Commercial_Military	Transporters of Gooc	include		
Beneficiary	Commercial_Military	Transporters of Gooc	near		

Category	Class	SubClass	Word_type	S12	S13
Beneficiary	Commerical_Industri	Resource-Dependent	exclude		
Beneficiary	Commerical_Industri	Pharmaceutical and I	include		
Beneficiary	Commerical_Industri	Pharmaceutical and I	near		
Beneficiary	Commerical_Industri	Pharmaceutical and I	exclude		
Beneficiary	Commerical_Industri	Fur_Hide Trappers ar	include		
Beneficiary	Commerical_Industri	Fur_Hide Trappers ar	near		
Beneficiary	Commerical_Industri	Fur_Hide Trappers ar	exclude		
Beneficiary	Government and Mu	Government and Mu	include		
Beneficiary	Government and Mu	Government and Mu	near		
Beneficiary	Government and Mu	Government and Mu	exclude		
Beneficiary	Government and Mu	Municipal Drinking W	include		
Beneficiary	Government and Mu	Municipal Drinking W	exclude		
Beneficiary	Government and Mu	Wastewater Treatme	include		
Beneficiary	Government and Mu	Wastewater Treatme	exclude		
Beneficiary	Government and Mu	Residential Property	include		
Beneficiary	Government and Mu	Residential Property	near		
Beneficiary	Government and Mu	Residential Property	exclude		
Beneficiary	Government and Mu	Military_Coast Guard	include		
Beneficiary	Government and Mu	Military_Coast Guard	exclude		
Beneficiary	Commercial_Military	Commercial_Military	include		
Beneficiary	Commercial_Military	Commercial_Military	near		
Beneficiary	Commercial_Military	Commercial_Military	exclude		
Beneficiary	Commercial_Military	Transporters of Good	include		
Beneficiary	Commercial_Military	Transporters of Good	near		

Category	Class	SubClass	Word_type	S1
Beneficiary	Commercial_Military	Transporters of Gooc	exclude	
Beneficiary	Commercial_Military	Transporters of Peop	include	people.*transport|transport.*people
Beneficiary	Commercial_Military	Transporters of Peop	near	
Beneficiary	Commercial_Military	Transporters of Peop	exclude	sediment.*transport|transport.*sediment|contam.*transport|transport.*contam|nutrient.*transport|transport.*nutrient
Beneficiary	Subsistence	Subsistence	include	subsistence
Beneficiary	Subsistence	Subsistence	near	
Beneficiary	Subsistence	Subsistence	exclude	
Beneficiary	Subsistence	Water Subsisters	include	cistern|rain.*barrel
Beneficiary	Subsistence	Water Subsisters	near	
Beneficiary	Subsistence	Water Subsisters	exclude	drain|train|brain|grain
Beneficiary	Subsistence	Food Subsisters	include	food|hunt|gatherer|gathering|harvest
Beneficiary	Subsistence	Food Subsisters	near	subsist|tribal|tribe|indigenous.*people|indian population|indian people|indigenous.*cultur|native.*person|native american|traditional use|traditional sustainable use|sustenance
Beneficiary	Subsistence	Food Subsisters	exclude	web|chain|food.*species|flock|shelter|data|food waste|food bag
Beneficiary	Subsistence	Timber and Fiber anc	include	skin|hide| fur | furs |fur-bear|fur bear|furbear|pelt|tanning|feather
Beneficiary	Subsistence	Timber and Fiber anc	near	subsist|tribal|tribe|indigenous.*people|indian population|indian people|indigenous.*cultur|indigenous.*people|indian population|indian people|native.*person|native american|traditional use|traditional sustainable use|sustenance|traditional way|depend on the land|dependent on the land|dependent upon the land

<table>
<tr><th>Category</th><th>Class</th><th>SubClass</th><th>Word_type</th><th>S2</th><th>S3</th></tr>
<tr><td>Beneficiary</td><td>Commercial_Military</td><td>Transporters of Gooc</td><td>exclude</td><td>transport|support|report|opportunit|import|portion|sport|portuguese|airport|portray|export|porta|puport|portugal|disproportion|westport|port.*native|purported|cockroach|susan|port.*of</td><td></td></tr>
<tr><td>Beneficiary</td><td>Commercial_Military</td><td>Transporters of Peop</td><td>include</td><td>cruise|ferry|ferries|airplane|airport|airstrip|air.*runway|runway.*air|airfield</td><td>train|railroad|railway|gundalow</td></tr>
<tr><td>Beneficiary</td><td>Commercial_Military</td><td>Transporters of Peop</td><td>near</td><td></td><td>commut|people|travel|passenger</td></tr>
<tr><td>Beneficiary</td><td>Commercial_Military</td><td>Transporters of Peop</td><td>exclude</td><td></td><td>training|trained|constrain|entrain|strain|train.*about|train people</td></tr>
<tr><td>Beneficiary</td><td>Subsistence</td><td>Subsistence</td><td>include</td><td>subsist|tribal|tribe|indigenous.*people|indian population|indian people|indian population|indian people|indigenous.*people|indian population|indian people|indian population|indian people|native.*person|native american|traditional use|traditional sustainable use|sustenance|traditional way|depend on the land|dependent on the land|dependent upon the land|traditional knowledge|traditional ecological knowledge</td><td>traditional use|traditional sustainable use|traditional way</td></tr>
<tr><td>Beneficiary</td><td>Subsistence</td><td>Subsistence</td><td>near</td><td>resource|use|surviv</td><td></td></tr>
<tr><td>Beneficiary</td><td>Subsistence</td><td>Subsistence</td><td>exclude</td><td>because|horticultur|agricultur|alternativ|aqua.*cultur|monocultur|non-nativ|independent|interdependent</td><td></td></tr>
<tr><td>Beneficiary</td><td>Subsistence</td><td>Water Subsisters</td><td>include</td><td>water</td><td>store.*rain|rain.*store|storage.*rain|rain.*storage|collect.*rain|rain.*collect|collect.*rain|rain.*collect</td></tr>
<tr><td>Beneficiary</td><td>Subsistence</td><td>Water Subsisters</td><td>near</td><td>subsistence|tribal|tribe|indigenous.*people|indian population|indian people|indigenous.*cultur|indigenous.*people|indian population|indian people|native cultur|native person|native american|native village|traditional use|traditional sustainable use|sustenance|traditional way|depend on the land|dependent on the land|dependent upon the land</td><td>later use|reuse|re-use|drink|irrigat|water.*lawn|water.*grass|water.*yard</td></tr>
<tr><td>Beneficiary</td><td>Subsistence</td><td>Water Subsisters</td><td>exclude</td><td>horticultur|agricultur|alternativ|aqua.*cultur|monocultur|non-nativ|independent|interdependent|waterfront|waterfowl|watershed</td><td>restor|drain|train|brain|grain|rainbow|grass pickerel</td></tr>
<tr><td>Beneficiary</td><td>Subsistence</td><td>Food Subsisters</td><td>include</td><td>food|berries|berry|mushroom|fruit|vegetabl|herb|shellfish|clam|mussel|rice|hunt|fish|shrimp|salmon|oyster|pike|crab|lobster|mullet|mussel|bass|herring|grouper|snapper|alewife|flounder|abalone|scallop|clam|duck|turkey|deer|waterfowl|quail|honey</td><td></td></tr>
<tr><td>Beneficiary</td><td>Subsistence</td><td>Food Subsisters</td><td>near</td><td>subsistence|tribal|tribe|indigenous.*people|indian population|indian people|indigenous.*cultur|indigenous.*people|indian population|indian people|native cultur|native person|native american|native village|traditional use|traditional sustainable use|sustenance|traditional way|depend on the land|dependent on the land|dependent upon the land</td><td></td></tr>
<tr><td>Beneficiary</td><td>Subsistence</td><td>Food Subsisters</td><td>exclude</td><td>horticultur|agricultur|alternativ|aqua.*cultur|monocultur|non-nativ|ambassador|salmon falls|independent|interdependent|duck farm|clami|oyster creek</td><td></td></tr>
<tr><td>Beneficiary</td><td>Subsistence</td><td>Timber and Fiber anc</td><td>include</td><td>nutria|beaver|deer|alligator|hogs|rabbit</td><td>timber|fiber|wood|pulp|pine|oak|cypress| elm|ash|maple|hickory|hardwood|cedar|cordwood|reed|grass|salt hay|salt marsh hay|turpentine</td></tr>
<tr><td>Beneficiary</td><td>Subsistence</td><td>Timber and Fiber anc</td><td>near</td><td>subsistence|tribal|tribe|indigenous.*people|indian population|indian people|indigenous.*cultur|indigenous.*people|indian population|indian people|native cultur|native person|native american|native village|traditional use|traditional sustainable use|sustenance|traditional way|depend on the land|dependent on the land|dependent upon the land</td><td>subsistence|tribal|tribe|indigenous.*people|indian population|indian people|indigenous.*cultur|indigenous.*people|indian population|indian people|native cultur|native person|native american|native village|traditional use|traditional sustainable use|sustenance|traditional way|depend on the land|dependent on the land|dependent upon the land</td></tr>
</table>

<table>
<tr><th>Category</th><th>Class</th><th>SubClass</th><th>Word_type</th><th>S4</th><th>S5</th></tr>
<tr><td>Beneficiary</td><td>Commercial_Military</td><td>Transporters of Gooc</td><td>exclude</td><td>training|trained|constrain|entrain|strain|train.*about</td><td>trail|pair|fair|air yam|air plant|chair|questionnaire|airborne|affair|millionaire|dairy|airshed|National.*Weather.*Service|impair|repair|belleair|air.*act|prair|clean.*air.*act|national.*air.*standard|regulat.*air|lair|Air Resource Specialist|Clean Air Bond|dair|www|Air Resources Lab|air rifle|air condition|airboat|stair|nair|bair|fair|air yam|air plant|air temp|hair|sediment.*transport|transport.*sediment|contam.*transport|transport.*contam|nutrient.*transport|transport.*nutrient</td></tr>
<tr><td>Beneficiary</td><td>Commercial_Military</td><td>Transporters of Peop</td><td>include</td><td>rail.*transportation|transportation.*rail</td><td>parking</td></tr>
<tr><td>Beneficiary</td><td>Commercial_Military</td><td>Transporters of Peop</td><td>near</td><td>commut|people|travel|passenger</td><td>area|lot</td></tr>
<tr><td>Beneficiary</td><td>Commercial_Military</td><td>Transporters of Peop</td><td>exclude</td><td>trail</td><td></td></tr>
<tr><td>Beneficiary</td><td>Subsistence</td><td>Subsistence</td><td>include</td><td>depend on the land|dependent on the land|dependent upon the land</td><td></td></tr>
<tr><td>Beneficiary</td><td>Subsistence</td><td>Subsistence</td><td>near</td><td></td><td></td></tr>
<tr><td>Beneficiary</td><td>Subsistence</td><td>Subsistence</td><td>exclude</td><td></td><td></td></tr>
<tr><td>Beneficiary</td><td>Subsistence</td><td>Water Subsisters</td><td>include</td><td>water well|supply well|domestic well|private well|artesian well|shallow well|groundwater well</td><td></td></tr>
<tr><td>Beneficiary</td><td>Subsistence</td><td>Water Subsisters</td><td>near</td><td>swells|wells reserve|wells bay|town of wells</td><td></td></tr>
<tr><td>Beneficiary</td><td>Subsistence</td><td>Water Subsisters</td><td>exclude</td><td></td><td></td></tr>
<tr><td>Beneficiary</td><td>Subsistence</td><td>Food Subsisters</td><td>include</td><td></td><td></td></tr>
<tr><td>Beneficiary</td><td>Subsistence</td><td>Food Subsisters</td><td>near</td><td></td><td></td></tr>
<tr><td>Beneficiary</td><td>Subsistence</td><td>Food Subsisters</td><td>exclude</td><td></td><td></td></tr>
<tr><td>Beneficiary</td><td>Subsistence</td><td>Timber and Fiber anc</td><td>include</td><td>firewood|campfire</td><td></td></tr>
<tr><td>Beneficiary</td><td>Subsistence</td><td>Timber and Fiber anc</td><td>near</td><td></td><td></td></tr>
</table>

Category	Class	SubClass	Word_type	S6	S7
Beneficiary	Commercial_Military	Transporters of Gooc	exclude		
Beneficiary	Commercial_Military	Transporters of Peop	include		
Beneficiary	Commercial_Military	Transporters of Peop	near		
Beneficiary	Commercial_Military	Transporters of Peop	exclude		
Beneficiary	Subsistence	Subsistence	include		
Beneficiary	Subsistence	Subsistence	near		
Beneficiary	Subsistence	Subsistence	exclude		
Beneficiary	Subsistence	Water Subsisters	include		
Beneficiary	Subsistence	Water Subsisters	near		
Beneficiary	Subsistence	Water Subsisters	exclude		
Beneficiary	Subsistence	Food Subsisters	include		
Beneficiary	Subsistence	Food Subsisters	near		
Beneficiary	Subsistence	Food Subsisters	exclude		
Beneficiary	Subsistence	Timber and Fiber anc	include		
Beneficiary	Subsistence	Timber and Fiber anc	near		

Category	Class	SubClass	Word_type	S8	S9
Beneficiary	Commercial_Military	Transporters of Gooc	exclude		
Beneficiary	Commercial_Military	Transporters of Peop	include		
Beneficiary	Commercial_Military	Transporters of Peop	near		
Beneficiary	Commercial_Military	Transporters of Peop	exclude		
Beneficiary	Subsistence	Subsistence	include		
Beneficiary	Subsistence	Subsistence	near		
Beneficiary	Subsistence	Subsistence	exclude		
Beneficiary	Subsistence	Water Subsisters	include		
Beneficiary	Subsistence	Water Subsisters	near		
Beneficiary	Subsistence	Water Subsisters	exclude		
Beneficiary	Subsistence	Food Subsisters	include		
Beneficiary	Subsistence	Food Subsisters	near		
Beneficiary	Subsistence	Food Subsisters	exclude		
Beneficiary	Subsistence	Timber and Fiber anc	include		
Beneficiary	Subsistence	Timber and Fiber anc	near		

Category	Class	SubClass	Word_type	S10	S11
Beneficiary	Commercial_Military	Transporters of Gooc	exclude		
Beneficiary	Commercial_Military	Transporters of Peop	include		
Beneficiary	Commercial_Military	Transporters of Peop	near		
Beneficiary	Commercial_Military	Transporters of Peop	exclude		
Beneficiary	Subsistence	Subsistence	include		
Beneficiary	Subsistence	Subsistence	near		
Beneficiary	Subsistence	Subsistence	exclude		
Beneficiary	Subsistence	Water Subsisters	include		
Beneficiary	Subsistence	Water Subsisters	near		
Beneficiary	Subsistence	Water Subsisters	exclude		
Beneficiary	Subsistence	Food Subsisters	include		
Beneficiary	Subsistence	Food Subsisters	near		
Beneficiary	Subsistence	Food Subsisters	exclude		
Beneficiary	Subsistence	Timber and Fiber anc	include		
Beneficiary	Subsistence	Timber and Fiber anc	near		

Category	Class	SubClass	Word_type	S12	S13
Beneficiary	Commercial_Military	Transporters of Good	exclude		
Beneficiary	Commercial_Military	Transporters of Peop	include		
Beneficiary	Commercial_Military	Transporters of Peop	near		
Beneficiary	Commercial_Military	Transporters of Peop	exclude		
Beneficiary	Subsistence	Subsistence	include		
Beneficiary	Subsistence	Subsistence	near		
Beneficiary	Subsistence	Subsistence	exclude		
Beneficiary	Subsistence	Water Subsisters	include		
Beneficiary	Subsistence	Water Subsisters	near		
Beneficiary	Subsistence	Water Subsisters	exclude		
Beneficiary	Subsistence	Food Subsisters	include		
Beneficiary	Subsistence	Food Subsisters	near		
Beneficiary	Subsistence	Food Subsisters	exclude		
Beneficiary	Subsistence	Timber and Fiber and	include		
Beneficiary	Subsistence	Timber and Fiber and	near		

Category	Class	SubClass	Word_type	S1
Beneficiary	Subsistence	Timber and Fiber anc	exclude	askin|sking|salem|mysella|economus|newmarket|sulfur|peltandra|horticultur|agricultur|alternativ|aqua.*cultur|monocultur|non-nativ|pelto|apelt|peltu|pelta|pelte|provid.* no clue|provid.* notice|provid.* basis|provid.*expertise|pelti|refur|furc|iskin|skink|skin lesion|skin condition|independ
Beneficiary	Subsistence	Building Material Sut	include	grass|reed|mud|stone|rock|granit|limestone|shells|coral|rock|sand
Beneficiary	Subsistence	Building Material Sut	near	subsistence|tribal|tribe|indigenous.*people|indian population|indian people|indigenous.*cultur|indigenous.*people|indian population|indian people|native cultur|native person|native american|native village|traditional use|traditional sustainable use|sustenance|traditional way|depend on the land|dependent on the land|dependent upon the land
Beneficiary	Subsistence	Building Material Sut	exclude	grassroot|grass-root|independ|capstone|keystone|touchstone|horticultur|agricultur|alternativ|aqua.*cultur|monocultur|non-nativ|cornerstone|agreed|breed|milestone|rockport|rocky mountain|gabion|rockcress|rockfish|larock|rock crab|shamrock|rock shrimp|rock cancer|rockaway|rocky|Rock Cove|rockingham|bedrock|rock fish|rock jasmine|rockport|rockaway|rocky|Rock Cove|sediment|channel|hammock|legend|dune|management.*description|report|inventory|list.*structure|burnunit|burn.*unit|facilit|forest.*manag|gis|document|data|spit|transport|site|adverse|bedrock|clay creek|clayton|sandwich|runoff|sandwort|sandine|ersand|esand|dsand|sandy|sanddune|sand dune|sandspur|sander|ssand|sandwort|sandpiper|sand filter|esand|dsand|sand pine|sandusky|sand grass|sandplain|sandown|thousand|carex|carey|personal care|careo|careful|aquarium care|lawn care|wood.*smoke|sediment|channel|preservativ|pah|hab|hazard|highhigh
Beneficiary	Recreational	Recreational	include	recreation|vacation|amenities|visitor|tourist|tourism|visiting
Beneficiary	Recreational	Recreational	exclude	research|scien|student|teach
Beneficiary	Recreational	experiencrs and View	include	horseback rid|hike|hiking|rock climb|bike|biking|bicycl|camping|camper|camps|campground|birding|birder|outing|sunbath|beach.*comb|sightsee|shelling|beach.*activit|sight-see|sun-bath|beach.*goer|beach.*going|picnic|beach.*collect|collect.*beach|gather.*beach|beach.*gather|snowmobil|skiing|sledding|ski area|ski resort|snow sport|off-road vehicle|birdwatch|bird watch|wildlife view|view wildlife|encounter|whale sight|whale watch|nature tour|bird blind|watch bird

Category	Class	SubClass	Word_type	S2	S3
Beneficiary	Subsistence	Timber and Fiber anc	exclude	deerfield|productiv|harvest.*crop|crop.*harvest|harvest data|horticultur|agricultur|alternativ|aqua.*cultur|monocultur|non-nativ|independ	grassroot|grass-<br>root|independ|marshfield|marshall|braintree|park|woody|lakewood|kirkwood| woodpecker|woods hole|wood dock|wood preserv|woodneck|woodbury|wood<br>stake|wooden|wood tick|woodland|wood turtle|tree frog|woodbridge|treated<br>lumber|treated wood|wood<br>stove|reproduct|horticultur|agricultur|alternativ|aqua.*cultur|monocultur|non-<br>nativ|cash|category|sewage|sanitary|acquatic|management.*area|fish|wash|dnr<br>|mcbp|trash|shoreline|pineda|pinellas|legend|pine.*island|pine.*barren|vash|el<br>mer|elm st|ashe|coal<br>ash|mash|sash|cash|bash|overwhelm|oakdale|oakley|croak|coal.*ash|delmarva<br>|fashion|pineland|barrier.*island|water.*pollution|soak|pine.*beach|over.*whel<br>med|barrier.*beach|ashore|sand.*pine|shorebird|percent|metals|oakland|silver<br>side|hash|develm|hairstreak|crash|pine.*hills|ocean.*pine|oak.*harbor|oak.*rid<br>ge|pine.*seed|sea grass|freed|mcreedy|sea-<br>grass|eelgrass|oyster.*grass|seagrass|grassland|sea-<br>grass|decreed|breed|agreed|haynes|hay.*barn|hayden|cottonwood|freed|cotto<br>ntail|reedgrass|hayan|woodfern|sandalwoods|wood satyr|wood borer|wood<br>nymph
Beneficiary	Subsistence	Building Material Sul	include	build|construct	
Beneficiary	Subsistence	Building Material Sul	near	subsistence|tribal|tribe|indigenous.*people|indian population|indian people|indigenous.*cultur|indigenous.*people|indian population|indian people|native cultur|native person|native american|native village|traditional use|traditional sustainable use|sustenance|traditional way|depend on the land|dependent on the land|dependent upon the land	
Beneficiary	Subsistence	Building Material Sul	exclude	horticultur|agricultur|alternativ|aqua.*cultur|monocultur|non-nativ|capacity	
Beneficiary	Recreational	Recreational	include	golf course|athletic field|baseball|football|soccer	
Beneficiary	Recreational	Recreational	exclude		
Beneficiary	Recreational	experiencrs and View	include	outdoor|natur|wildlife|animal|wildflower|out-of doors	pier|waterfront|boardwalk

Category	Class	SubClass	Word_type	S4	S5
Beneficiary	Subsistence	Timber and Fiber anc	exclude		
Beneficiary	Subsistence	Building Material Sul	include		
Beneficiary	Subsistence	Building Material Sul	near		
Beneficiary	Subsistence	Building Material Sul	exclude		
Beneficiary	Recreational	Recreational	include		
Beneficiary	Recreational	Recreational	exclude		
Beneficiary	Recreational	experiencrs and View	include	trail	family|families|children|visitor|touris|roamer|roaming

Category	Class	SubClass	Word_type	S6	S7
Beneficiary	Subsistence	Timber and Fiber anc	exclude		
Beneficiary	Subsistence	Building Material Sul	include		
Beneficiary	Subsistence	Building Material Sul	near		
Beneficiary	Subsistence	Building Material Sul	exclude		
Beneficiary	Recreational	Recreational	include		
Beneficiary	Recreational	Recreational	exclude		
Beneficiary	Recreational	experiencrs and View	include	collect|gather|pick	view|watcher|watching|witness

Category	Class	SubClass	Word_type	S8	S9
Beneficiary	Subsistence	Timber and Fiber anc	exclude		
Beneficiary	Subsistence	Building Material Sul	include		
Beneficiary	Subsistence	Building Material Sul	near		
Beneficiary	Subsistence	Building Material Sul	exclude		
Beneficiary	Recreational	Recreational	include		
Beneficiary	Recreational	Recreational	exclude		
Beneficiary	Recreational	experiencrs and View	include	scenic|scenery|horizon|skyline|vantage point|vistas|observation point|observation deck|observation platform|observation area|vista point|scenic vista|natural vista|beauty|beautiful|panorama|panoramic|viewscape|view scape|idyllic	sight

Category	Class	SubClass	Word_type	S10	S11
Beneficiary	Subsistence	Timber and Fiber anc	exclude		
Beneficiary	Subsistence	Building Material Sul	include		
Beneficiary	Subsistence	Building Material Sul	near		
Beneficiary	Subsistence	Building Material Sul	exclude		
Beneficiary	Recreational	Recreational	include		
Beneficiary	Recreational	Recreational	exclude		
Beneficiary	Recreational	experiencrs and View	include	outdoor|natur|wildlife|animal|bird|whale	sound|listen|sensation|sensory|auditory|noise|audible|scent|sensory|olfactory|auditory|smell|listen|noise|aroma|visual|insects.*hear|hear.*insects|fragrant|fragrance

Category	Class	SubClass	Word_type	S12	S13
Beneficiary	Subsistence	Timber and Fiber anc	exclude		
Beneficiary	Subsistence	Building Material Sul	include		
Beneficiary	Subsistence	Building Material Sul	near		
Beneficiary	Subsistence	Building Material Sul	exclude		
Beneficiary	Recreational	Recreational	include		
Beneficiary	Recreational	Recreational	exclude		
Beneficiary	Recreational	experiencrs and View	include	sunrise|sunset|eclipse|sundown|rainbow|twilight	

Category	Class	SubClass	Word_type	S1
Beneficiary	Recreational	experiencrs and View	near	
Beneficiary	Recreational	experiencrs and View	exclude	campaign|suppl|campbell|salmon.*camp|highigh|management.*issue|forsythe|jug.*bay|answers.*question|divert|wastewater|steelhead|diversion|trailer|traillii |hitchhike|ladder|passage barrier|barriers|bay scallop
Beneficiary	Recreational	Food Pickers and Gat	include	collect|pick|gather|recreat|edible|recreat|sport|visitor|touris
Beneficiary	Recreational	Food Pickers and Gat	near	food|berries|berry|mushroom|fruit|herbs|nut|roots|asparagus
Beneficiary	Recreational	Food Pickers and Gat	exclude	requirement|review|bayview|data|specimen|nutrient|nutria|minute|transport|cranberr|hunt|trap
Beneficiary	Recreational	Hunters	include	hunt|trap
Beneficiary	Recreational	Hunters	near	recreat|sport|opportunit|experienc|enjoy|relax|families|family|children|visitor|tours|permit|activit|activities

Category	Class	SubClass	Word_type	S2	S3
Beneficiary	Recreational	experiencrs and View	near	scenic|panoramic|aesthetic|experienc|enjoy|relax|observ|view|overlook|vista|opportun	
Beneficiary	Recreational	experiencrs and View	exclude	bayview|review|bayview|overview|in view of|over-view|over-view|peer|viewpoint|purview|viewed|observ.*study|professional|angler|fish|boat|hunt|swimmer|swimming|diver|diving|swam|collect|gather|pick|natural resource|naturally|broader view|broad view|often overlook|may be overlooked|overlook.*concern|	pierce|piermont|pierson|pieris|fishing pier|pierre|waterfront resident
Beneficiary	Recreational	Food Pickers and Gat	include	collect|pick|gather|recreat|edible|recreat|sport|visitor|touris	recreat|sport|opportunit|experienc|enjoy|relax|families|family|children|visitor|t ouris
Beneficiary	Recreational	Food Pickers and Gat	near	shellfish|clam|oyster|scallop|lobster|mussel|crab|shrimp|abalone|crawfish|crayf ish|crustacea	clammer|clamming|shellfisher|shellfishing|clam.*dig|dig.*clam|shellfish.*dig|dig *shellfish
Beneficiary	Recreational	Food Pickers and Gat	exclude	requirement|review|bayview|data|specimen|transport|fishery|fisheries|horseshoe crab|samples|clami|pickerel|oyster creek	indigenous|digest|fishery|fisheries
Beneficiary	Recreational	Hunters	include	hunt|trapper|trapping	waterfowl|turkey|deer|quail|duck|beaver|nutria|alligator|hogs|rabbit
Beneficiary	Recreational	Hunters	near		recreat|sport|permit

Category	Class	SubClass	Word_type	S4	S5
Beneficiary	Recreational	experiencrs and View	near		outdoor|natur|wildlife|animal|scenic|panoramic|wildflower|aesthetic|view|observ|overlook|vista|experienc|enjoy|relax|opportun
Beneficiary	Recreational	experiencrs and View	exclude	trailer|hitchhike|traillii|hunt	bayview|review|bayview|overview|in view of|over-view|over-view|peer|viewpoint|purview|viewed|observ.*study|professional|angler|fish|boat|hunt|swimmer|swimming|diver|diving|swam|collect|gather|pick|natural resource|naturally|broader view|broad view|often overlook|may overlook|overlook.*concern
Beneficiary	Recreational	Food Pickers and Gat	include	clammer|clamming|shellfisher|shellfishing|clam.*dig|dig.*clam|shellfish.*dig|dig*shellfish	collect|pick|gather|recreat|sport|trail|season|opportunit|experienc|enjoy|relax|families|family|children
Beneficiary	Recreational	Food Pickers and Gat	near		edible
Beneficiary	Recreational	Food Pickers and Gat	exclude	extract|commercial|business|product|industry|commodity|commodities|goods|harvest|profit|buy|livelihood|job|dealer|market|corporation|sale|sell|income|artisan|profession|indigenous|digest|fishery|fisheries|trade|trading|reproduct|productivity|salem|mysella|economus	
Beneficiary	Recreational	Hunters	include	game	hide|skin| fur | furs |fur-bear|fur bear|furbear|pelt
Beneficiary	Recreational	Hunters	near	animal|bird|wild|harvest	

<table>
<tr><th>Category</th><th>Class</th><th>SubClass</th><th>Word_type</th><th>S6</th><th>S7</th></tr>
<tr><td>Beneficiary</td><td>Recreational</td><td>experiencrs and View</td><td>near</td><td>natural material|flower|shells|seeds|natural object|plants|insects|fungi|mushroo</td><td>spectacle|natur|environment|ecosystem|estuary|coastal|landscape|ocean|shore|beach|water|accomodat|activit|aesthetic|ameniable|amenit|appreciat|artisan|artist|asset|attract|beaut|beneficial|benefit|bequest|bountiful|bounty|business|buy|care|caring|charisma|cherish|children|collect|comfort|commercial|commodities|commodity|goods|concern|conserv|corporation|critical|dealer|delicious|desired|desirable|destination|econom|edible|educat|encourag|endangered|enhance|enhancing|enjoy|encounter|enthusiast|experienc|exploit|extract|families|favorable|favor|feel|festiv|ceremon| fur | furs |fur-bear|fur bear|furbear|future|gather|generation|hallmark|harvest|healthy|heritage|hide|hunt|income|indigenous.*people|indian population|indian people|indigenous.*person|industry|initiative|inspir|interest|job|learn|legacy|leisure|livelihood|market|meat|native.*person|native american|observ|opportunit|participant|participate|pelt|pick|pleasant|popular|preserv|products|profession|profit|promote|promotion|purpose|protect|quality.*life|rare|recreat|relax|resource|restor|sale|save|saving|sell|sight|skins|special|specimen|spectacular|sport|steward|subsist|sustenance|threatened|together|tours|trade|trading|sustainabl|tradition|treasure|tribal|tribe|unique|valuable|value|valuing|view|visit|visitor|watch|well suited|well-suited|wonder|allure|alluring|mystique|option value|lifeblood|jewel|extraordinary|excellent|amaze|amazing|stunning|majestic|spectacular</td></tr>
<tr><td>Beneficiary</td><td>Recreational</td><td>experiencrs and View</td><td>exclude</td><td>transplant|gather.*for.*hunt|pickerel</td><td>bayview|viewcontent|views as issues|target view|view.*data|points of view|elevation view|known view|view this document|seek the view|solicit the view|reflect the view|review|bayview|interview|data.*network|aesthetic.*nuisance|aesthetic.*problem|aesthetic.*degradation|vista.*hermosa|vistamar|views.*agency|views.*department|sewage.*collection|aerial.*view.*modif|15.*visitor|treatment|keep.*louisiana|regulation|program.*beautification|beautification.*program|inspir.*problem|inspir.*strateg|program.*inspire.*action|conference.*inspir|tool.*inspir|siberian|chamisso|sandwort|pondweed|goal|success.*inspir|legislat|meadowbeauty|educat.*inspir|mission|initiative|agency.*funding|slough|program|slough|15.*visitor|plankton.*reduce|ad.*hoc|inspir.*steward|inspir.*teach|beautyberry|Harper.*beauty|meadow-beauty|meadow beauty|river beauty|beautiful jacob|spring beauty|overview|in view of|over-view|over- view|viewed|our view|species viewer|point of view|exhibit|landscape design|landscape ecology|built landscape|landscaper|purview|viewpoint|view point|negative view|their views|landscape scale|landscape perspective|temperature value|biomass value|salem|mysella|economus|newmarket|reproduc|productivity|broad view|broader view</td></tr>
<tr><td>Beneficiary</td><td>Recreational</td><td>Food Pickers and Gat</td><td>include</td><td></td><td></td></tr>
<tr><td>Beneficiary</td><td>Recreational</td><td>Food Pickers and Gat</td><td>near</td><td></td><td></td></tr>
<tr><td>Beneficiary</td><td>Recreational</td><td>Food Pickers and Gat</td><td>exclude</td><td></td><td></td></tr>
<tr><td>Beneficiary</td><td>Recreational</td><td>Hunters</td><td>include</td><td></td><td></td></tr>
<tr><td>Beneficiary</td><td>Recreational</td><td>Hunters</td><td>near</td><td></td><td></td></tr>
</table>

Category	Class	SubClass	Word_type	S8	S9
Beneficiary	Recreational	experiencrs and View	near		comfort|desirable|destination|enjoy|favorable|pleasant|popular|relax|special|spectacular|tours|treasure|well suited|well-suited|wonder|allure|alluring|mystique|extraordinary|excellent|amaze|amazing|stunning|majestic|spectacular|scenic
Beneficiary	Recreational	experiencrs and View	exclude	meadow beauty|river beauty|beautiful jacob|spring beauty|meadow-beauty|meadowbeauty|horizontal|deepwater horizon|may be over	
Beneficiary	Recreational	Food Pickers and Gat	include		
Beneficiary	Recreational	Food Pickers and Gat	near		
Beneficiary	Recreational	Food Pickers and Gat	exclude		
Beneficiary	Recreational	Hunters	include		
Beneficiary	Recreational	Hunters	near		

Category	Class	SubClass	Word_type	S10	S11
Beneficiary	Recreational	experiencrs and View	near	view|overlook|vista|watch|sight|observ	
Beneficiary	Recreational	experiencrs and View	exclude	bayview|review|bayview|overview|in view of|over-view|over-view|peer|viewpoint|purview|viewed|observ.*study|professional|angler|fish|boat|hunt|swimmer|swimming|diver|diving|swam|collect|gather|pick|natural resource|naturally|snowbird|broad view|broader view|often overlook|may overlook|overlook.*concern	aromaticum|listening post|environmentally sound|western sound|polycylic|george sound|west sound|south sound|pugetsound|sound science|sound improvement|Albemarle sound|pamlico sound|long island sound|art|educat|inspir|the sound|Puget sound|croaker|buffer|listening session|fluorescent|quiescent|crescent|audubon|15.*visitor|noxious|unpleasant|using|increasing|rising|losing|accessing|dressing|crossing|disposing|promising|passing|proposing|isinglass|licensing|assessing|raising|single|foreclosing|purchasing|collapsing|processing|data|monitor|measur|crucial|glisten|excessive|unnecesary|hearing|heart|pollut|shear|hear appeal|disruptive|disturb|escent
Beneficiary	Recreational	Food Pickers and Gat	include		
Beneficiary	Recreational	Food Pickers and Gat	near		
Beneficiary	Recreational	Food Pickers and Gat	exclude		
Beneficiary	Recreational	Hunters	include		
Beneficiary	Recreational	Hunters	near		

Category	Class	SubClass	Word_type	S12	S13
Beneficiary	Recreational	experiencrs and View	near		
Beneficiary	Recreational	experiencrs and View	exclude	rodent|staff|presentation|created.*volunteer|15.*visitor|pollution|pollutant|climate|temperature|marina|island|provision|15.*visitor|trout|smelt|sunset of|sunset on|biofog|to sunset|image|imaging|clouded|navigat|sunset of|sunset on|sunset in|sunsets on|sunsets in|infog|turbid|rainbow snake|after sunset|rainbow star|rainbow surf|sunset at|before sunset|sunset cove|sunrise to	
Beneficiary	Recreational	Food Pickers and Gat	include		
Beneficiary	Recreational	Food Pickers and Gat	near		
Beneficiary	Recreational	Food Pickers and Gat	exclude		
Beneficiary	Recreational	Hunters	include		
Beneficiary	Recreational	Hunters	near		

Category	Class	SubClass	Word_type	S1
Beneficiary	Recreational	Hunters	exclude	transport|riparian|buffer|vegetat|extrapolat|strap|entrap|strap|sediment.*trap|contam.*trap|pollut.*trap|trap.*contam|trap.*pollut|nutrient.*trap|trap.*nutrient|sand.*trap|trap.*sand|trap.*sediment|litter trap|fish trap|lobster trap|trap.*site|transport|lobster|shunt|hunts point
Beneficiary	Recreational	Anglers	include	angler|angling
Beneficiary	Recreational	Anglers	near	
Beneficiary	Recreational	Anglers	exclude	dangl|rangl|sangl|tangl|bangl|wangl
Beneficiary	Recreational	Waders and Swimme	include	snorkel|SCUBA|swim|swam|wade|diving|dive|diver|wading|bather|bathing
Beneficiary	Recreational	Waders and Swimme	exclude	diversi|diverse|divert|biodiversity|diving.*duck|wastewater|steelhead|diversion|flock|spoonbill|egret|waterfowl|wading.*bird|swamp|kwader|diverg|swimming crab|migrat.*swim|SWIM plan
Beneficiary	Recreational	Boaters	include	boat|canoe|kayak|rowing|sailing|sailor|jetski|jet.*ski|surfing|surfer|watercraft|paddle|parasail|sail.*boat
Beneficiary	Recreational	Boaters	near	
Beneficiary	Recreational	Boaters	exclude	growing|longboat.*pass|surface|boat.*method|borrow|burrow|crow|hrow|grow|volcano|rrow
Beneficiary	Inspirational	Inspirational	include	inspir|cultural.*significance|cherish|treasure|wonder|beaut|renowned
Beneficiary	Inspirational	Inspirational	near	
Beneficiary	Inspirational	Inspirational	exclude	treasurer|meadow beauty|river beauty|beautiful jacob|spring beauty|meadowbeauty|meadow-beauty|beautyberry|Harper.*beauty
Beneficiary	Inspirational	Spiritual and Ceremo	include	festival|observance|religi|ceremon|baptism|wedding|spirit|worship|prayer
Beneficiary	Inspirational	Spiritual and Ceremo	near	
Beneficiary	Inspirational	Spiritual and Ceremo	exclude	formal.*designation.*ceremony|entrepreneur|cooperation|spirit of trust|spirit lake
Beneficiary	Inspirational	Artists	include	poet|painter|artist|sculpture|sculptor|sculpting|carving|pottery|photographer|painting|artwork| art |mural|nature photog|outdoor photog|environmental photog|local photog
Beneficiary	Inspirational	Artists	near	
Beneficiary	Inspirational	Artists	exclude	sculpta|aerial photograph|digitiz|orthophotograph|sculptus|the art of|the art and science of|photo-voltaic|photovoltaic|popcorn flower|popcornflower
Beneficiary	Learning	Learning	include	learn
Beneficiary	Learning	Learning	near	
Beneficiary	Learning	Learning	exclude	clearnose
Beneficiary	Learning	Educators and Stude	include	educat|academ|teach|professor|student|elementary|middle.*school|junior.*high|secondary.*school|high.*school|school.*children|fieldtrip|field trip|outdoor lab|classroom|classes|internship|outdoor school|K-12

Category	Class	SubClass	Word_type	S2	S3
Beneficiary	Recreational	Hunters	exclude	extract|commercial|business|product|industry|commodity|commodities|goods|harvest|profit|buy|livelihood|job|dealer|market|corporation|sale|sell|income|artisan|profession|shunt|huntley|huntington|hunterdon|trade|trading|transport|riparian|buffer|vegetat|extrapolat|strap|entrap|strap|sediment.*trap|contam.*trap|pollut.*trap|trap.*contam|trap.*pollut|nutrient.*trap|trap.*nutrient|sand.*trap|trap.*sand|trap.*sediment|litter trap|fish trap|lobster trap|trap.*site|transport|lobster|shunt|hunts point	transport|duck farm|quahogs
Beneficiary	Recreational	Anglers	include	fisher|fishing	fly-fishing|fly fishing|fly fisher|fly-fisher|flyfisher|flyfishing
Beneficiary	Recreational	Anglers	near	recreat|sport|opportunit|experienc|enjoy|relax|families|family|children|visitor|tours|salt water|saltwater|salt-water|fresh water|freshwater|fresh-water	
Beneficiary	Recreational	Anglers	exclude	shellfish|kingfisher|fishery|fisheries|fort fisher	
Beneficiary	Recreational	Waders and Swimme	include		
Beneficiary	Recreational	Waders and Swimme	exclude		
Beneficiary	Recreational	Boaters	include	harbor|dock|marina|channel	
Beneficiary	Recreational	Boaters	near	traffic|wake|activit|moor|private|public|access|available|crowd	
Beneficiary	Recreational	Boaters	exclude	public safety|public support|Bight|haddock|curl|sour|tropic|paraguay|fiddle|swamp|docket|harbor seal|dock permit|New Bedford Harbor|zostera|publicize|publicity|publication	
Beneficiary	Inspirational	Inspirational	include	culture|cultural|historic	sense of place|sense of community|pride|way of life
Beneficiary	Inspirational	Inspirational	near	significan|value|valuable|valuing|important|vital|resource|need	
Beneficiary	Inspirational	Inspirational	exclude	agricultur|aquacultur|horticultur|silvicultur|multicultur|agri.*cultur|aqua.*cultur|culturing|data|abundance|biomass|habitat|temperature value|biomass value|cultured|historical range|historically	scotian pride
Beneficiary	Inspirational	Spiritual and Ceremo	include	community event|special event|popular.*event|free event	day
Beneficiary	Inspirational	Spiritual and Ceremo	near		event|sponsor|participate|participant
Beneficiary	Inspirational	Spiritual and Ceremo	exclude	storm event|precipitation event	storm event|precipitation event
Beneficiary	Inspirational	Artists	include	writer|writing|author	contest
Beneficiary	Inspirational	Artists	near	inspir|environment|outdoor|natur|visit|tour|creativ	christmas card|essay|poetry| art |photograph
Beneficiary	Inspirational	Artists	exclude	document|research|grant|authori|TMDL|plan	sculpta|aerial photograph|digitiz|orthophotograph|sculptus|the art of|the art and science of
Beneficiary	Learning	Learning	include	museum|visitor.*center|diorama| graphic	interpretive|interactive|educat
Beneficiary	Learning	Learning	near		trail|guide|center|program
Beneficiary	Learning	Learning	exclude		
Beneficiary	Learning	Educators and Stude	include	youth development	

Category	Class	SubClass	Word_type	S4	S5
Beneficiary	Recreational	Hunters	exclude	snowbird	askin|sking|salem|mysella|economus|newmarket|sulfur|peltandra|horticultur|agricultur|alternativ|aqua.*cultur|monocultur|non-nativ|pelto|apelt|peltu|pelta|pelte|provid.* no clue|provid.* notice|provid.* basis|provid.*expertise|pelti|refur|furc|iskin|skink|skin lesion|skin condition|subsist|tribal|tribe|indigenous.*people|indian population|indian people|indigenous.*cultur|indigenous.*people|indian population|indian people|native.*person|native american|traditional use|traditional sustainable use|sustenance|traditional way|depend on the land|dependent on the land|dependent upon the land|extract|commercial|business|product|industry|commodity|commodities|goods|harvest|profit|buy|livelihood|job|dealer|market|corporation|sale|sell|income|artisan|profession|trade|trading|worker
Beneficiary	Recreational	Anglers	include	bait	fishermen|fisherman|fishing
Beneficiary	Recreational	Anglers	near	salem|reproduc|productivity	
Beneficiary	Recreational	Anglers	exclude	commercial|business|industry|commodity|commodities|goods|profit|buy|livelihood|job|dealer|market|corporation|sale|sell|income|artisan|profession|trade|trading|worker|fishery|fisheries	commercial|business|industry|commodity|commodities|goods|profit|buy|livelihood|job|dealer|market|corporation|sale|sell|income|artisan|profession|trade|trading|worker|fishery|fisheries|subsist|tribal|tribe|indigenous.*people|indian population|indian people|indigenous.*cultur|indigenous.*people|indian population|indian people|native.*person|native american|traditional use|traditional sustainable use|sustenance|traditional way|depend on the land|dependent on the land|dependent upon the land
Beneficiary	Recreational	Waders and Swimme	include		
Beneficiary	Recreational	Waders and Swimme	exclude		
Beneficiary	Recreational	Boaters	include		
Beneficiary	Recreational	Boaters	near		
Beneficiary	Recreational	Boaters	exclude		
Beneficiary	Inspirational	Inspirational	include		
Beneficiary	Inspirational	Inspirational	near		
Beneficiary	Inspirational	Inspirational	exclude		
Beneficiary	Inspirational	Spiritual and Ceremo	include		
Beneficiary	Inspirational	Spiritual and Ceremo	near		
Beneficiary	Inspirational	Spiritual and Ceremo	exclude		
Beneficiary	Inspirational	Artists	include		
Beneficiary	Inspirational	Artists	near		
Beneficiary	Inspirational	Artists	exclude		
Beneficiary	Learning	Learning	include	natural history	program|center
Beneficiary	Learning	Learning	near		environment|outdoor|science|educat|nature|natural
Beneficiary	Learning	Learning	exclude		abatement|manage|the estuary program|national estuary program
Beneficiary	Learning	Educators and Stude	include		

Category	Class	SubClass	Word_type	S6	S7
Beneficiary	Recreational	Hunters	exclude		
Beneficiary	Recreational	Anglers	include		
Beneficiary	Recreational	Anglers	near		
Beneficiary	Recreational	Anglers	exclude		
Beneficiary	Recreational	Waders and Swimme	include		
Beneficiary	Recreational	Waders and Swimme	exclude		
Beneficiary	Recreational	Boaters	include		
Beneficiary	Recreational	Boaters	near		
Beneficiary	Recreational	Boaters	exclude		
Beneficiary	Inspirational	Inspirational	include		
Beneficiary	Inspirational	Inspirational	near		
Beneficiary	Inspirational	Inspirational	exclude		
Beneficiary	Inspirational	Spiritual and Ceremo	include		
Beneficiary	Inspirational	Spiritual and Ceremo	near		
Beneficiary	Inspirational	Spiritual and Ceremo	exclude		
Beneficiary	Inspirational	Artists	include		
Beneficiary	Inspirational	Artists	near		
Beneficiary	Inspirational	Artists	exclude		
Beneficiary	Learning	Learning	include	history tour|discovery lab|lab offering	
Beneficiary	Learning	Learning	near		
Beneficiary	Learning	Learning	exclude		
Beneficiary	Learning	Educators and Stude	include		

Category	Class	SubClass	Word_type	S8	S9
Beneficiary	Recreational	Hunters	exclude		
Beneficiary	Recreational	Anglers	include		
Beneficiary	Recreational	Anglers	near		
Beneficiary	Recreational	Anglers	exclude		
Beneficiary	Recreational	Waders and Swimme	include		
Beneficiary	Recreational	Waders and Swimme	exclude		
Beneficiary	Recreational	Boaters	include		
Beneficiary	Recreational	Boaters	near		
Beneficiary	Recreational	Boaters	exclude		
Beneficiary	Inspirational	Inspirational	include		
Beneficiary	Inspirational	Inspirational	near		
Beneficiary	Inspirational	Inspirational	exclude		
Beneficiary	Inspirational	Spiritual and Ceremo	include		
Beneficiary	Inspirational	Spiritual and Ceremo	near		
Beneficiary	Inspirational	Spiritual and Ceremo	exclude		
Beneficiary	Inspirational	Artists	include		
Beneficiary	Inspirational	Artists	near		
Beneficiary	Inspirational	Artists	exclude		
Beneficiary	Learning	Learning	include		
Beneficiary	Learning	Learning	near		
Beneficiary	Learning	Learning	exclude		
Beneficiary	Learning	Educators and Stude	include		

Category	Class	SubClass	Word_type	S10	S11
Beneficiary	Recreational	Hunters	exclude		
Beneficiary	Recreational	Anglers	include		
Beneficiary	Recreational	Anglers	near		
Beneficiary	Recreational	Anglers	exclude		
Beneficiary	Recreational	Waders and Swimme	include		
Beneficiary	Recreational	Waders and Swimme	exclude		
Beneficiary	Recreational	Boaters	include		
Beneficiary	Recreational	Boaters	near		
Beneficiary	Recreational	Boaters	exclude		
Beneficiary	Inspirational	Inspirational	include		
Beneficiary	Inspirational	Inspirational	near		
Beneficiary	Inspirational	Inspirational	exclude		
Beneficiary	Inspirational	Spiritual and Ceremo	include		
Beneficiary	Inspirational	Spiritual and Ceremo	near		
Beneficiary	Inspirational	Spiritual and Ceremo	exclude		
Beneficiary	Inspirational	Artists	include		
Beneficiary	Inspirational	Artists	near		
Beneficiary	Inspirational	Artists	exclude		
Beneficiary	Learning	Learning	include		
Beneficiary	Learning	Learning	near		
Beneficiary	Learning	Learning	exclude		
Beneficiary	Learning	Educators and Stude	include		

Category	Class	SubClass	Word_type	S12	S13
Beneficiary	Recreational	Hunters	exclude		
Beneficiary	Recreational	Anglers	include		
Beneficiary	Recreational	Anglers	near		
Beneficiary	Recreational	Anglers	exclude		
Beneficiary	Recreational	Waders and Swimme	include		
Beneficiary	Recreational	Waders and Swimme	exclude		
Beneficiary	Recreational	Boaters	include		
Beneficiary	Recreational	Boaters	near		
Beneficiary	Recreational	Boaters	exclude		
Beneficiary	Inspirational	Inspirational	include		
Beneficiary	Inspirational	Inspirational	near		
Beneficiary	Inspirational	Inspirational	exclude		
Beneficiary	Inspirational	Spiritual and Ceremo	include		
Beneficiary	Inspirational	Spiritual and Ceremo	near		
Beneficiary	Inspirational	Spiritual and Ceremo	exclude		
Beneficiary	Inspirational	Artists	include		
Beneficiary	Inspirational	Artists	near		
Beneficiary	Inspirational	Artists	exclude		
Beneficiary	Learning	Learning	include		
Beneficiary	Learning	Learning	near		
Beneficiary	Learning	Learning	exclude		
Beneficiary	Learning	Educators and Stude	include		

Category	Class	SubClass	Word_type	S1
Beneficiary	Learning	Educators and Stude	near	
Beneficiary	Learning	Educators and Stude	exclude	educational.*seminar|grf|educat.*center|educat.*plan
Beneficiary	Learning	Researchers	include	research|scien|specimen.*collect|sample.*collect|data.*collect|collect.*specimen|collect.*sample|collect.*data|gather.*data|data.*gather
Beneficiary	Learning	Researchers	near	promote|promotion|opportunit|encourag|accomodat|attract|interest|initiative|potential|purpose|permit|improve.*knowledge|enhance.*knowledge|knowledge *improve|knowledge.*enhance|improve.*learn|enhance.*learn|learn.*improve|learn.*enhance|permission|engage|visit|excellent|estuarine lab|natural lab|field lab|marine lab|enhanc.*understand|promoting|enhancing
Beneficiary	Learning	Researchers	exclude	Marine Protection, Research, and Sanctuaries Act|MPRSA|Research Reserve|scientific name
Beneficiary	Non-Use	Non-Use	include	non-use|nonuse|non use
Beneficiary	Non-Use	Non-Use	near	resource|opportunit|valu
Beneficiary	Non-Use	Non-Use	exclude	
Beneficiary	Non-Use	People Who Care (Ex	include	care|caring|conserv|appreciat|unique|endangered|threatened
Beneficiary	Non-Use	People Who Care (Ex	near	
Beneficiary	Non-Use	People Who Care (Ex	exclude	careful|carex|carey|personal care|careo|caren|aquarium care|lawn care|caret
Beneficiary	Non-Use	People Who Care (Option_Bequest)	include	bequest|option value|steward|landtrust|land trust|inheritance trust|sustainable|sustainability
Beneficiary	Non-Use	People Who Care (Option_Bequest)	near	
Beneficiary	Non-Use	People Who Care (Option_Bequest)	exclude	
Beneficiary	Humanity	All Humans	include	humanity|everyone|humankind|all ages|all people
Beneficiary	Humanity	All Humans	near	
Beneficiary	Humanity	All Humans	exclude	activit

Category	Class	SubClass	Word_type	S2	S3
Beneficiary	Learning	Educators and Stude	near		
Beneficiary	Learning	Educators and Stude	exclude		
Beneficiary	Learning	Researchers	include	research lab|visiting scientist|postdoc|post-doc|graduate student|graduate research	research.* use| use.*research| use.*scien|scien.* use|support.*research|enable.*research|enabling.*research|for research|scientific.*resource|resource.*scientific
Beneficiary	Learning	Researchers	near		
Beneficiary	Learning	Researchers	exclude		house|scientific name
Beneficiary	Non-Use	Non-Use	include		
Beneficiary	Non-Use	Non-Use	near		
Beneficiary	Non-Use	Non-Use	exclude		
Beneficiary	Non-Use	People Who Care (Ex	include	species of concern|species of special concern|endangered species|threatened species|ecologically sensitive|environmentally sensitive|sensitive ecosystem|sensitive aquatic|sensitive area|sensitive species	rare|protect|critical
Beneficiary	Non-Use	People Who Care (Ex	near		organism|vertebrate|invertebrate|species|animal|bird|fauna|flora|plant|wildlife|raptor|fauna|animal|mammal|wildlife|bird|owl|fowl|duck|sparrow|warbler|plover|falcon|osprey|hawk|eagle|pelican|geese|pidgeon|pigeon|turtle|tortoise|seal|whale|dolphin|porpoise|manatee|otter|coral|dragonfl|insect|beetle|entomolo|bug|deer|turkey|quail|waterfowl|duck|goose|reptile|alligator|amphibian|salamander|frog|toad|skink|bat|nutria|raccoon|hogs|rabbit|beaver|rookery|rookeries|butter|horseshoe crab|flora|plant|flower|root|grass|kelp|seaweed|vegetation|mangrove|reed|cypress|sphagnum|cattail|moss|palm|berry|berries|seeds|fruit|nuts|tree|sedge|moss|lichen|forest|pine|oak|cypress| elm|ash|maple|hickory|hardwood|cedar|habitat|ecosystem|environment|natur|estuar|bay|ecological|aquatic|marine|water|park|coast|shore|land|wetland|the reserve|floodplain|biological|basin|region| area|watershed
Beneficiary	Non-Use	People Who Care (Ex	exclude		rarely|hazard.*protect|protect.*hazard|flood.*protect|storm.*protect|shoreline.*protect|coastal.*protect|dune.*protect|wave.*protect|erosion.*protect|protect.*flood|protect.*storm|protect.*shoreline|protect.*coastal|protect.*dune|protect.*wave|protect.*erosion|protect public health|protect public safety|toad flax
Beneficiary	Non-Use	People Who Care (Option_Bequest)	include	future|next	intergeneration|inter-generation|grandchildren|grand-children
Beneficiary	Non-Use	People Who Care (Option_Bequest)	near	generation	
Beneficiary	Non-Use	People Who Care (Option_Bequest)	exclude	power|electric	
Beneficiary	Humanity	All Humans	include	quality of life|public health|human health|wellbeing|well being|well-being|general health|general welfare	safe
Beneficiary	Humanity	All Humans	near		
Beneficiary	Humanity	All Humans	exclude	department of public health|public health department	boat|safeguard|pilot|harbor|port|vessel|ship|maritime safety|hunter safety|wildlife safety|hunting safety

Category	Class	SubClass	Word_type	S4	S5
Beneficiary	Learning	Educators and Stude	near		
Beneficiary	Learning	Educators and Stude	exclude		
Beneficiary	Learning	Researchers	include		
Beneficiary	Learning	Researchers	near		
Beneficiary	Learning	Researchers	exclude		
Beneficiary	Non-Use	Non-Use	include		
Beneficiary	Non-Use	Non-Use	near		
Beneficiary	Non-Use	Non-Use	exclude		
Beneficiary	Non-Use	People Who Care (Ex	include	existence value|right to exist	
Beneficiary	Non-Use	People Who Care (Ex	near		
Beneficiary	Non-Use	People Who Care (Ex	exclude		
Beneficiary	Non-Use	People Who Care (Option_Bequest)	include	future|heritage|legacy|inherit	
Beneficiary	Non-Use	People Who Care (Option_Bequest)	near	enjoy|preserv|conserv|invest|protect|significan|value|valuable|valuing|important|vital|resource|need|coast|estuar| bay| lands|nature|natural|wild|the area|the region|our area|our region|the watershed|our watershed	
Beneficiary	Non-Use	People Who Care (Option_Bequest)	exclude	temperature value|biomass value|the regional	
Beneficiary	Humanity	All Humans	include		
Beneficiary	Humanity	All Humans	near		
Beneficiary	Humanity	All Humans	exclude		

Category	Class	SubClass	Word_type	S6	S7
Beneficiary	Learning	Educators and Stude	near		
Beneficiary	Learning	Educators and Stude	exclude		
Beneficiary	Learning	Researchers	include		
Beneficiary	Learning	Researchers	near		
Beneficiary	Learning	Researchers	exclude		
Beneficiary	Non-Use	Non-Use	include		
Beneficiary	Non-Use	Non-Use	near		
Beneficiary	Non-Use	Non-Use	exclude		
Beneficiary	Non-Use	People Who Care (Ex	include		
Beneficiary	Non-Use	People Who Care (Ex	near		
Beneficiary	Non-Use	People Who Care (Ex	exclude		
Beneficiary	Non-Use	People Who Care (Option_Bequest)	include		
Beneficiary	Non-Use	People Who Care (Option_Bequest)	near		
Beneficiary	Non-Use	People Who Care (Option_Bequest)	exclude		
Beneficiary	Humanity	All Humans	include		
Beneficiary	Humanity	All Humans	near		
Beneficiary	Humanity	All Humans	exclude		

Category	Class	SubClass	Word_type	S8	S9
Beneficiary	Learning	Educators and Stude	near		
Beneficiary	Learning	Educators and Stude	exclude		
Beneficiary	Learning	Researchers	include		
Beneficiary	Learning	Researchers	near		
Beneficiary	Learning	Researchers	exclude		
Beneficiary	Non-Use	Non-Use	include		
Beneficiary	Non-Use	Non-Use	near		
Beneficiary	Non-Use	Non-Use	exclude		
Beneficiary	Non-Use	People Who Care (Ex	include		
Beneficiary	Non-Use	People Who Care (Ex	near		
Beneficiary	Non-Use	People Who Care (Ex	exclude		
Beneficiary	Non-Use	People Who Care (Option_Bequest)	include		
Beneficiary	Non-Use	People Who Care (Option_Bequest)	near		
Beneficiary	Non-Use	People Who Care (Option_Bequest)	exclude		
Beneficiary	Humanity	All Humans	include		
Beneficiary	Humanity	All Humans	near		
Beneficiary	Humanity	All Humans	exclude		

Category	Class	SubClass	Word_type	S10	S11
Beneficiary	Learning	Educators and Stude	near		
Beneficiary	Learning	Educators and Stude	exclude		
Beneficiary	Learning	Researchers	include		
Beneficiary	Learning	Researchers	near		
Beneficiary	Learning	Researchers	exclude		
Beneficiary	Non-Use	Non-Use	include		
Beneficiary	Non-Use	Non-Use	near		
Beneficiary	Non-Use	Non-Use	exclude		
Beneficiary	Non-Use	People Who Care (Ex	include		
Beneficiary	Non-Use	People Who Care (Ex	near		
Beneficiary	Non-Use	People Who Care (Ex	exclude		
Beneficiary	Non-Use	People Who Care (Option_Bequest)	include		
Beneficiary	Non-Use	People Who Care (Option_Bequest)	near		
Beneficiary	Non-Use	People Who Care (Option_Bequest)	exclude		
Beneficiary	Humanity	All Humans	include		
Beneficiary	Humanity	All Humans	near		
Beneficiary	Humanity	All Humans	exclude		

Category	Class	SubClass	Word_type	S12	S13
Beneficiary	Learning	Educators and Stude	near		
Beneficiary	Learning	Educators and Stude	exclude		
Beneficiary	Learning	Researchers	include		
Beneficiary	Learning	Researchers	near		
Beneficiary	Learning	Researchers	exclude		
Beneficiary	Non-Use	Non-Use	include		
Beneficiary	Non-Use	Non-Use	near		
Beneficiary	Non-Use	Non-Use	exclude		
Beneficiary	Non-Use	People Who Care (Ex	include		
Beneficiary	Non-Use	People Who Care (Ex	near		
Beneficiary	Non-Use	People Who Care (Ex	exclude		
Beneficiary	Non-Use	People Who Care (Option_Bequest)	include		
Beneficiary	Non-Use	People Who Care (Option_Bequest)	near		
Beneficiary	Non-Use	People Who Care (Option_Bequest)	exclude		
Beneficiary	Humanity	All Humans	include		
Beneficiary	Humanity	All Humans	near		
Beneficiary	Humanity	All Humans	exclude		

Category	Class	SubClass	Word_type	S1
Environmen t	Environment	Environment	include	vegetation|nature|natural|habitat|the reserve|the sound|open space
Environmen t	Environment	Environment	near	
Environmen t	Environment	Environment	exclude	aquatic|terrestrial|river|stream|wetland|lake|pond|estuar|marine|ocean|grass|scrub|shrub|forest|agricult|agro|tundra|greenspace|marsh|swamp|tidal|SAV|ri parian
Environment	Aquatic	Aquatic	include	aquatic
Environment	Aquatic	Aquatic	near	
Environment	Aquatic	Aquatic	exclude	aquaticus|river|stream|bay|wetland|marsh|mangrove|estuary|groundwater|ocean|pond|reservoir
Environment	Aquatic	Rivers and Streams	include	river|creek|canal|stream|channel|riparian
Environment	Aquatic	Rivers and Streams	exclude	streamline|bloodstream|driver
Environment	Aquatic	Wetlands	include	wetland|bog|floodplain|depression|marsh|swamp|slough
Environment	Aquatic	Wetlands	exclude	patbogen|marshfield|marshall
Environment	Aquatic	Lakes and Ponds	include	lake
Environment	Aquatic	Lakes and Ponds	near	
Environment	Aquatic	Lakes and Ponds	exclude	lakeville|lake.*national
Environment	Aquatic	Estuaries and Near C	include	estuar|gulf|puget sound|bay
Environment	Aquatic	Estuaries and Near C	near	
Environment	Aquatic	Estuaries and Near C	exclude	embayment
Environment	Aquatic	Open Oceans and Se	include	ocean|open water|continental shelf|deep water
Environment	Aquatic	Open Oceans and Se	exclude	www|oceanography|http|open.*space|ocean.*council|open.*marsh|animal|national.*oceanic|ocean.*plan|ocean.*city|ocean.*park|legend|ocean.*county|ocean .*act|open.*watershed|ocean.*commission
Environment	Aquatic	Groundwater	include	groundwater|ground-water|ground water
Environment	Aquatic	Groundwater	exclude	protection.*act|action
Environment	Terrestrial	Terrestrial	include	terrestrial|upland|island|shell.*mound|shell.*midden|mountain|meadow|volcano
Environment	Terrestrial	Terrestrial	near	

Category	Class	SubClass	Word_type	S2	S3
Environmen t	Environment	Environment	include	environment|ecosystem|outdoor	flora|fauna|species|wildlife|watershed
Environmen t	Environment	Environment	near		
Environmen t	Environment	Environment	exclude	outdoor.*plan|ecosystem.*plan|aquatic|terrestrial|river|stream|wetland|lake|pond|estuar|marine|ocean|grass|scrub|shrub|forest|agricult|agro|tundra|greenspace|marsh|swamp|tidal|SAV|riparian	aquatic|terrestrial|river|stream|wetland|lake|pond|estuar|marine|ocean|grass|scrub|shrub|forest|agricult|agro|tundra|greenspace|marsh|swamp|tidal|SAV
Environment	Aquatic	Aquatic	include	water	benthic
Environment	Aquatic	Aquatic	near		
Environment	Aquatic	Aquatic	exclude	watershed|freewater|river|stream|bay|wetland|marsh|mangrove|estuary|groundwater|ocean|pond|reservoir	river|stream|bay|wetland|marsh|mangrove|estuary|groundwater|ocean|pond|reservoir
Environment	Aquatic	Rivers and Streams	include		
Environment	Aquatic	Rivers and Streams	exclude		
Environment	Aquatic	Wetlands	include	rush fen|sedge fen|shrub fen|marsh hay|salt hay	
Environment	Aquatic	Wetlands	exclude		
Environment	Aquatic	Lakes and Ponds	include	pond	reservoir
Environment	Aquatic	Lakes and Ponds	near		
Environment	Aquatic	Lakes and Ponds	exclude	ponder|respond|pondweed|pond.*turtle|mill.*pond|transpond	
Environment	Aquatic	Estuaries and Near C	include	tidal|tide	coral|reef|artificial.*reef|shipwreck
Environment	Aquatic	Estuaries and Near C	near		
Environment	Aquatic	Estuaries and Near C	exclude	brown tide|red tide|browntide|redtide	treefrog
Environment	Aquatic	Open Oceans and Se	include	sea	kelp.*forest
Environment	Aquatic	Open Oceans and Se	exclude	disease|search|season|level|seagrass|grant|seal|research|seablite|sea.*grass|roseate|seapurslane|seabeach|seabrook|seam|seat|seattle|sea oat	
Environment	Aquatic	Groundwater	include	aquifer	geyser
Environment	Aquatic	Groundwater	exclude		
Environment	Terrestrial	Terrestrial	include	land	plant
Environment	Terrestrial	Terrestrial	near		

Category	Class	SubClass	Word_type	S4	S5
Environmen t	Environment	Environment	include	resource|value|valuable|trading|mitigat|goods|econom|public access|conserv|protect|tourism|tourist|recreation|livelihood|visitor use|stewards|benefit	beneficial use|living resource|natural feature|natural resource|ecosystem service|ecological service|economic use|commercial use|recreational use|cultural.*resource|traditional use|sustainable tourism
Environmen t	Environment	Environment	near	ecosystem|environment|natur|renewable|ecological|the reserve|biological|the region|our region|the area|our area	
Environmen t	Environment	Environment	exclude	Department|sewer service|office|division|commission|bay scallop|lander|landing|landmark|southland|parking|parker|economical|protection agency|environmentally|temperature value|biomass value|the regional|aquatic|terrestrial|river|stream|wetland|lake|pond|estuar|marine|ocean|grass|scrub|shrub|forest|agricult|agro|tundra|greenspace|marsh|swamp|tidal |SAV|riparian	department|aquatic|terrestrial|river|stream|wetland|lake|pond|estuar|marine|ocean|grass|scrub|shrub|forest|agricult|agro|tundra|greenspace|marsh|swamp|tidal|SAV|riparian
Environment	Aquatic	Aquatic	include	TMDL|pollution|discharg|effluent|hydro|emission	fish|seahorse|aquaria|aquarium|seafood|salmon|oyster|pike|crab|lobster|mullet|mussel|bass|herring|grouper|snapper|alewife|flounder|abalone|scallop|clam|geoduck|shrimp|tarpon|shellfish|angler|angling|mollusk
Environment	Aquatic	Aquatic	near	water|marina|boat|ship	
Environment	Aquatic	Aquatic	exclude	river|stream|bay|wetland|marsh|mangrove|estuary|groundwater|ocean|pond|reservoir	spike|clami|pickerel|oyster creek|dangl|rangl|sangl|tangl|bangl|wangl|river|stream|bay|wetland|marsh|mangrove|estuary|groundwater|ocean|pond|reservoir
Environment	Aquatic	Rivers and Streams	include		
Environment	Aquatic	Rivers and Streams	exclude		
Environment	Aquatic	Wetlands	include		
Environment	Aquatic	Wetlands	exclude		
Environment	Aquatic	Lakes and Ponds	include	vernal.*pool|pool.*vernal|temporary.*pool|pool.*temporary	quarry
Environment	Aquatic	Lakes and Ponds	near		water|flood
Environment	Aquatic	Lakes and Ponds	exclude		
Environment	Aquatic	Estuaries and Near C	include	seagrass|eelgrass|oyster.*grass|sea.*grass|eel.*grass|mangrove|SAV	lagoon|delta|mudflat|mud.*flat
Environment	Aquatic	Estuaries and Near C	near		
Environment	Aquatic	Estuaries and Near C	exclude	save|saving|savanna	
Environment	Aquatic	Open Oceans and Se	include	marine	
Environment	Aquatic	Open Oceans and Se	exclude		
Environment	Aquatic	Groundwater	include	underground.*reservoir|reservoir.*underground|underground.*water|water.*underground	
Environment	Aquatic	Groundwater	exclude		
Environment	Terrestrial	Terrestrial	include	flower|sediment|soil|grass|fungi|fungus	terrestrial|the area|the region|our region|our area|the watershed|our watershed
Environment	Terrestrial	Terrestrial	near		

Category	Class	SubClass	Word_type	S6	S7
Environment	Environment	Environment	include		
Environment	Environment	Environment	near		
Environment	Environment	Environment	exclude		
Environment	Aquatic	Aquatic	include	game|hunting|hunts|hunter	water|recreation|discharg|enjoy|moor|charter|tours|cruise|port|harbor|access|traffic|channel|canal|launch
Environment	Aquatic	Aquatic	near	duck|waterfowl|goose|alligator	boat|ship|vessel|ferry|paddle
Environment	Aquatic	Aquatic	exclude	shunt|huntley|huntington|transport|scavenger hunt|river|stream|bay|wetland|marsh|mangrove|estuary|groundwater|ocean|hunts point|pond|reservoir	fish|stewardship|partnership|waterfowl|ownership|relationship|leadership|sponsorship|township|membership|shipped|rship|nship|support|harbor seal|new bedford harbor|bight|river|stream|bay|wetland|marsh|mangrove|estuary|groundwater|ocean|pond|reservoir
Environment	Aquatic	Rivers and Streams	include		
Environment	Aquatic	Rivers and Streams	exclude		
Environment	Aquatic	Wetlands	include		
Environment	Aquatic	Wetlands	exclude		
Environment	Aquatic	Lakes and Ponds	include		
Environment	Aquatic	Lakes and Ponds	near		
Environment	Aquatic	Lakes and Ponds	exclude		
Environment	Aquatic	Estuaries and Near C	include	bay|shore|coast	sunk
Environment	Aquatic	Estuaries and Near C	near		ship|vessel|barge
Environment	Aquatic	Estuaries and Near C	exclude	embayment|loblolly.*bay|coastal.*count|coastal.*city|coastal.*cities|coastal communit	
Environment	Aquatic	Open Oceans and Se	include		
Environment	Aquatic	Open Oceans and Se	exclude		`
Environment	Aquatic	Groundwater	include		
Environment	Aquatic	Groundwater	exclude		
Environment	Terrestrial	Terrestrial	include	tree	skin|hide| fur | furs |fur-bear|fur bear|furbear|meat|game
Environment	Terrestrial	Terrestrial	near		exploit|hunt|trap|harvest|company|corporation|trade

Category	Class	SubClass	Word_type	S8	S9
Environmen t	Environment	Environment	include		
Environmen t	Environment	Environment	near		
Environmen t	Environment	Environment	exclude		
Environment	Aquatic	Aquatic	include	watercraft|sailboat|tugboat|jetski|kayak|canoe|boating|boater|shipping channel	wildstock|wild.*stock|stock.*wild|wild.*catch|catch.*wild|wild.*caught|caught.* wild
Environment	Aquatic	Aquatic	near		fish|seahorse|aquaria|aquarium|seafood|salmon|shrimp|oyster|pike|crab|lobst er|mullet|mussel|bass|herring|grouper|snapper|alewife|flounder|abalone|scallo p|clam|geoduck|lake|river|stream
Environment	Aquatic	Aquatic	exclude	volcanoe|river|stream|bay|wetland|marsh|mangrove|estuary|groundwater|ocea	livestock|wildlife|wild animal|spike|clami|river|stream|bay|wetland|marsh|mangrove|estuary|ground water|ocean|pond|reservoir
Environment	Aquatic	Rivers and Streams	include		
Environment	Aquatic	Rivers and Streams	exclude		
Environment	Aquatic	Wetlands	include		
Environment	Aquatic	Wetlands	exclude		
Environment	Aquatic	Lakes and Ponds	include		
Environment	Aquatic	Lakes and Ponds	near		
Environment	Aquatic	Lakes and Ponds	exclude		
Environment	Aquatic	Estuaries and Near C	include		
Environment	Aquatic	Estuaries and Near C	near		
Environment	Aquatic	Estuaries and Near C	exclude		
Environment	Aquatic	Open Oceans and Se	include		
Environment	Aquatic	Open Oceans and Se	exclude		
Environment	Aquatic	Groundwater	include		
Environment	Aquatic	Groundwater	exclude		
Environment	Terrestrial	Terrestrial	include		
Environment	Terrestrial	Terrestrial	near		

Category	Class	SubClass	Word_type	S10	S11
Environmen t	Environment	Environment	include		
Environmen t	Environment	Environment	near		
Environmen t	Environment	Environment	exclude		
Environment	Aquatic	Aquatic	include		
Environment	Aquatic	Aquatic	near		
Environment	Aquatic	Aquatic	exclude		
Environment	Aquatic	Rivers and Streams	include		
Environment	Aquatic	Rivers and Streams	exclude		
Environment	Aquatic	Wetlands	include		
Environment	Aquatic	Wetlands	exclude		
Environment	Aquatic	Lakes and Ponds	include		
Environment	Aquatic	Lakes and Ponds	near		
Environment	Aquatic	Lakes and Ponds	exclude		
Environment	Aquatic	Estuaries and Near C	include		
Environment	Aquatic	Estuaries and Near C	near		
Environment	Aquatic	Estuaries and Near C	exclude		
Environment	Aquatic	Open Oceans and Se	include		
Environment	Aquatic	Open Oceans and Se	exclude		
Environment	Aquatic	Groundwater	include		
Environment	Aquatic	Groundwater	exclude		
Environment	Terrestrial	Terrestrial	include		
Environment	Terrestrial	Terrestrial	near		

Category	Class	SubClass	Word_type	S12	S13
Environmen t	Environment	Environment	include		
Environmen t	Environment	Environment	near		
Environmen t	Environment	Environment	exclude		
Environment	Aquatic	Aquatic	include		
Environment	Aquatic	Aquatic	near		
Environment	Aquatic	Aquatic	exclude		
Environment	Aquatic	Rivers and Streams	include		
Environment	Aquatic	Rivers and Streams	exclude		
Environment	Aquatic	Wetlands	include		
Environment	Aquatic	Wetlands	exclude		
Environment	Aquatic	Lakes and Ponds	include		
Environment	Aquatic	Lakes and Ponds	near		
Environment	Aquatic	Lakes and Ponds	exclude		
Environment	Aquatic	Estuaries and Near C	include		
Environment	Aquatic	Estuaries and Near C	near		
Environment	Aquatic	Estuaries and Near C	exclude		
Environment	Aquatic	Open Oceans and Se	include		
Environment	Aquatic	Open Oceans and Se	exclude		
Environment	Aquatic	Groundwater	include		
Environment	Aquatic	Groundwater	exclude		
Environment	Terrestrial	Terrestrial	include		
Environment	Terrestrial	Terrestrial	near		

<table>
<tr><th>Category</th><th>Class</th><th>SubClass</th><th>Word_type</th><th>S1</th></tr>
<tr><td>Environment</td><td>Terrestrial</td><td>Terrestrial</td><td>exclude</td><td>rhode.*island|terrestrial.*species|island.*sound|grass|scrub|shrub|forest|agricult|agro|tundra|greenspace</td></tr>
<tr><td>Environment</td><td>Terrestrial</td><td>Forests</td><td>include</td><td>forest</td></tr>
<tr><td>Environment</td><td>Terrestrial</td><td>Forests</td><td>exclude</td><td>laudholm|vincent|kelp.*forest</td></tr>
<tr><td>Environment</td><td>Terrestrial</td><td>Agroecosystems</td><td>include</td><td>orchard|vineyard</td></tr>
<tr><td>Environment</td><td>Terrestrial</td><td>Agroecosystems</td><td>near</td><td></td></tr>
<tr><td>Environment</td><td>Terrestrial</td><td>Agroecosystems</td><td>exclude</td><td>macrophy|micropool|close</td></tr>
<tr><td>Environment</td><td>Terrestrial</td><td>Created Greenspace</td><td>include</td><td>park|trail</td></tr>
<tr><td>Environment</td><td>Terrestrial</td><td>Created Greenspace</td><td>near</td><td></td></tr>
<tr><td>Environment</td><td>Terrestrial</td><td>Created Greenspace</td><td>exclude</td><td>spark|parking|parkway|national.*park|department.*park|district.*park|research.*park|industrial.*park|park.*service|parkland|trailer|traillii</td></tr>
<tr><td>Environment</td><td>Terrestrial</td><td>Grasslands</td><td>include</td><td>prairie|grassland</td></tr>
<tr><td>Environment</td><td>Terrestrial</td><td>Grasslands</td><td>exclude</td><td>grassflat crab|grass shrimp|grass frog|grasshopper</td></tr>
<tr><td>Environment</td><td>Terrestrial</td><td>Scrublands_Shrublan</td><td>include</td><td>sageland|dune|scrub|shrub|chaparral</td></tr>
<tr><td>Environment</td><td>Terrestrial</td><td>Barren_Rock and Sar</td><td>include</td><td>barren|desert|beach|rock</td></tr>
<tr><td>Environment</td><td>Terrestrial</td><td>Barren_Rock and Sar</td><td>near</td><td></td></tr>
<tr><td>Environment</td><td>Terrestrial</td><td>Barren_Rock and Sar</td><td>exclude</td><td>pine|rockport|rocky mountain|gabion|rockcress|rockfish|larock|rock crab|shamrock|rock shrimp|rock cancer|intertidal|rockport|rockaway|rocky|Rock Cove|rockingham|bedrock|rock fish|rock jasmine|rockport|rockaway|rocky|Rock Cove|sediment|channel|hammock|legend|dune|management.*description|report|inventory|list.*structure|burnunit|burn.*unit|facilit|forest.*manag|gis|document|data|spit|transport|site|adverse|bedrock|honey bee|clay creek|clayton|sandwich|runoff|sandwort|sandine|ersand|sandy|esand|dsand|sanddune|sand dune|sandspur|sander|ssand|sandwort|sandpiper|sand filter|esand|dsand|sand pine|sandusky|sand grass|sandplain|sandown|thousand|carex|carey|personal care|careo|careful|aquarium care|lawn care|wood.*smoke|sediment|channel|preservativ|pah|hab|hazard|highhigh</td></tr>
<tr><td>Environment</td><td>Terrestrial</td><td>Tundra</td><td>include</td><td>tundra</td></tr>
<tr><td>Environment</td><td>Terrestrial</td><td>Tundra</td><td>exclude</td><td>tundra vole|tundra swan</td></tr>
<tr><td>Environment</td><td>Terrestrial</td><td>Ice and Snow</td><td>include</td><td>glacier</td></tr>
</table>

Category	Class	SubClass	Word_type	S2	S3
Environment	Terrestrial	Terrestrial	exclude	island.*sound|grass|scrub|shrub|forest|agricult|agro|tundra|greenspace|marsh|swamp|portland|tideland|wetland|woodland|lander|southland|landing|landmark|system.*land|landbird|landmark	industr|business|product|commercial|company|factory|processing|treatment|power|sea|ocean|water|aquatic|wetland|estuar|marine|river|stream|marsh|plantation|transplant
Environment	Terrestrial	Forests	include	wood	pine|oak|cypress| elm| ash|maple|hickory|hardwood|cedar
Environment	Terrestrial	Forests	exclude	park|lakewood|kirkwood|woods hole|wood dock|wood preserv|woodneck|woodbury|wood stake|wooden|woodbridge|treated lumber|treated wood|wood stove|woodii|wood-lettuce|wildwood|woodpecker|woodward|wood duck|woodrat|woods snake|wood stork|woodcock|woodfern|sandalwoods|wood satyr|wood borer|wood nymph	cash|category|sewage|sanitary|acquatic|management.*area|fish|wash|dnr|mcbp|trash|shoreline|pineda|pinellas|legend|pine.*island|vash|elmer|elmst|ashe|coal ash|mash|sash|cash|bash|overwhelm|oakdale|oakley|croak|coal.*ash|delmarva|fashion|barrier.*island|water.*pollution|soak|pine.*beach|over.*whelmed|barrier.*beach|ashore|sand.*pine|shorebird|percent|metals|oakland|silverside|hash|develm|hairstreak|crash|ocean.*pine|oak.*harbor|oak.*ridge|croak
Environment	Terrestrial	Agroecosystems	include	crops|pasture|hay|cropland	agroecosystem|agro-ecosystem
Environment	Terrestrial	Agroecosystems	near		
Environment	Terrestrial	Agroecosystems	exclude	suhayda|thayer|macrophy|micropool|close|haye|hayhoe|hayn|haya|marsh hay|salt hay|microp	
Environment	Terrestrial	Created Greenspace	include	greenspace|greenway|green space|green way|garden|green roof|green infrastructure	airfield|lawn|golf|yard|airstrip|runway.*air|air.*runway
Environment	Terrestrial	Created Greenspace	near		
Environment	Terrestrial	Created Greenspace	exclude	shellfish garden|oyster garden	vineyard|boatyard|boat yard|shipyard|ship yard|building yard|schoolyard|school yard
Environment	Terrestrial	Grasslands	include		
Environment	Terrestrial	Grasslands	exclude		
Environment	Terrestrial	Scrublands_Shrublan	include		
Environment	Terrestrial	Barren_Rock and Sar	include	sand	mine|mining
Environment	Terrestrial	Barren_Rock and Sar	near		pit|land|area|site
Environment	Terrestrial	Barren_Rock and Sar	exclude	sandwich|runoff|sandwort|sandine|ersand|esand|dsand|sanddune|sand dune|sandspur|sander|sandy|ssand|sandwort|sandpiper|sand filter|esand|dsand|sand pine|sandusky|sand grass|sandplain|sandown|thousand|sand plain|sandplain|metalsand|sand lance|sandalwood	data.*min|determining|examining|determine|examine|promine|mineral|flumine a|shells|minen|mineu|amine|plaquemine|ermin|minello|deter-min|deter-min|minea|post-min|division|omine|smine|reclaim.*min|min.*reclaim|min.*reclam|reclam.*min|under- min|lettuce
Environment	Terrestrial	Tundra	include	alpine	
Environment	Terrestrial	Tundra	exclude	headwater|alpine.*forest|circaea|ssc|alpine.*lake| NJ|new jersey|alpine mountain sorrel|alpine hawk|alpine forget|alpine draba	
Environment	Terrestrial	Ice and Snow	include	snow	ice

Category	Class	SubClass	Word_type	S4	S5
Environment	Terrestrial	Terrestrial	exclude	sea|ocean|water|aquatic|wetland|estuar|marine|river|stream|marsh|grassroot|seagrass|eelgrass|oyster.*grass|sea.*grass|eel.*grass|mangrove|SAV|soft sediment|bay|soft-sediment	the regional
Environment	Terrestrial	Forests	include	pineland	tree.*stand|stand.*tree
Environment	Terrestrial	Forests	exclude		street
Environment	Terrestrial	Agroecosystems	include	agricultur|farm|silvicultur	plantation
Environment	Terrestrial	Agroecosystems	near		sugar|coffee
Environment	Terrestrial	Agroecosystems	exclude	farm shellfish|farm fish|farm oyster|farm clam|farm mussel|farm shrimp|shellfish farm|fish farm|oyster farm|clam farm|mussel farm|shrimp farm|aqua farm|farm market|farmer.*market|aquacultur|aquafarm	transplant
Environment	Terrestrial	Created Greenspace	include	field	
Environment	Terrestrial	Created Greenspace	near	football|soccer|athletic|baseball	
Environment	Terrestrial	Created Greenspace	exclude		
Environment	Terrestrial	Grasslands	include		
Environment	Terrestrial	Grasslands	exclude		
Environment	Terrestrial	Scrublands_Shrublan	include		
Environment	Terrestrial	Barren_Rock and Sar	include	quarry	limestone
Environment	Terrestrial	Barren_Rock and Sar	near		
Environment	Terrestrial	Barren_Rock and Sar	exclude	flood|water	
Environment	Terrestrial	Tundra	include		
Environment	Terrestrial	Tundra	exclude		
Environment	Terrestrial	Ice and Snow	include		

<table>
<tr><th>Category</th><th>Class</th><th>SubClass</th><th>Word_type</th><th>S6</th><th>S7</th></tr>
<tr><td>Environment</td><td>Terrestrial</td><td>Terrestrial</td><td>exclude</td><td>braintree|street</td><td>agency|askin|pickerel|by-product|byproduct|by product|chid|entrap|strap|trap.*gas|gas.*trap|sediment.*trap|contam.*trap|nutrient.*trap|pollut.*trap|trap.*contam|trap.*nutrient|trap.*pollut|sand.*trap|trap.*sand|trap.*sediment|litter trap|fish trap|especial|power.*generation|electric.*generation|generation plant|generation facilit|laboratory observ|field observ|lab observ|special issue|meadow beauty|river beauty|beautiful jacob|spring beauty|beautyberry|carex|carey|personal care|careo|aquarium care|lawn care|careful|exotic|extrapolat|askin|pickerel|invasive species|iskin|nuisance species|overview|in view of|over-view|over-view|peltandra|pelti|pelto|apelt|peltu|pelta|pelte|provid.* no clue|provid.* notice|provid.* basis|provid.*expertise|rarely|temperature value|biomass value|nonprofit|non-profit|refur|review|bayview|sensitive fern|shunt|huntley|huntington|hunterdon|sking|salem|mysella|economus|new market|skink|skin lesion|skin condition|strap|sulfur|specialist|tradeoff|trade-off|transport|workshop|duck|waterfowl|goose|alligator</td></tr>
<tr><td>Environment</td><td>Terrestrial</td><td>Forests</td><td>include</td><td></td><td></td></tr>
<tr><td>Environment</td><td>Terrestrial</td><td>Forests</td><td>exclude</td><td></td><td></td></tr>
<tr><td>Environment</td><td>Terrestrial</td><td>Agroecosystems</td><td>include</td><td></td><td></td></tr>
<tr><td>Environment</td><td>Terrestrial</td><td>Agroecosystems</td><td>near</td><td></td><td></td></tr>
<tr><td>Environment</td><td>Terrestrial</td><td>Agroecosystems</td><td>exclude</td><td></td><td></td></tr>
<tr><td>Environment</td><td>Terrestrial</td><td>Created Greenspace</td><td>include</td><td></td><td></td></tr>
<tr><td>Environment</td><td>Terrestrial</td><td>Created Greenspace</td><td>near</td><td></td><td></td></tr>
<tr><td>Environment</td><td>Terrestrial</td><td>Created Greenspace</td><td>exclude</td><td></td><td></td></tr>
<tr><td>Environment</td><td>Terrestrial</td><td>Grasslands</td><td>include</td><td></td><td></td></tr>
<tr><td>Environment</td><td>Terrestrial</td><td>Grasslands</td><td>exclude</td><td></td><td></td></tr>
<tr><td>Environment</td><td>Terrestrial</td><td>Scrublands_Shrublan</td><td>include</td><td></td><td></td></tr>
<tr><td>Environment</td><td>Terrestrial</td><td>Barren_Rock and Sar</td><td>include</td><td></td><td></td></tr>
<tr><td>Environment</td><td>Terrestrial</td><td>Barren_Rock and Sar</td><td>near</td><td></td><td></td></tr>
<tr><td>Environment</td><td>Terrestrial</td><td>Barren_Rock and Sar</td><td>exclude</td><td></td><td></td></tr>
<tr><td>Environment</td><td>Terrestrial</td><td>Tundra</td><td>include</td><td></td><td></td></tr>
<tr><td>Environment</td><td>Terrestrial</td><td>Tundra</td><td>exclude</td><td></td><td></td></tr>
<tr><td>Environment</td><td>Terrestrial</td><td>Ice and Snow</td><td>include</td><td></td><td></td></tr>
</table>

Category	Class	SubClass	Word_type	S8	S9
Environment	Terrestrial	Terrestrial	exclude		
Environment	Terrestrial	Forests	include		
Environment	Terrestrial	Forests	exclude		
Environment	Terrestrial	Agroecosystems	include		
Environment	Terrestrial	Agroecosystems	near		
Environment	Terrestrial	Agroecosystems	exclude		
Environment	Terrestrial	Created Greenspace	include		
Environment	Terrestrial	Created Greenspace	near		
Environment	Terrestrial	Created Greenspace	exclude		
Environment	Terrestrial	Grasslands	include		
Environment	Terrestrial	Grasslands	exclude		
Environment	Terrestrial	Scrublands_Shrublan	include		
Environment	Terrestrial	Barren_Rock and Sar	include		
Environment	Terrestrial	Barren_Rock and Sar	near		
Environment	Terrestrial	Barren_Rock and Sar	exclude		
Environment	Terrestrial	Tundra	include		
Environment	Terrestrial	Tundra	exclude		
Environment	Terrestrial	Ice and Snow	include		

Category	Class	SubClass	Word_type	S10	S11
Environment	Terrestrial	Terrestrial	exclude		
Environment	Terrestrial	Forests	include		
Environment	Terrestrial	Forests	exclude		
Environment	Terrestrial	Agroecosystems	include		
Environment	Terrestrial	Agroecosystems	near		
Environment	Terrestrial	Agroecosystems	exclude		
Environment	Terrestrial	Created Greenspace	include		
Environment	Terrestrial	Created Greenspace	near		
Environment	Terrestrial	Created Greenspace	exclude		
Environment	Terrestrial	Grasslands	include		
Environment	Terrestrial	Grasslands	exclude		
Environment	Terrestrial	Scrublands_Shrublan	include		
Environment	Terrestrial	Barren_Rock and Sar	include		
Environment	Terrestrial	Barren_Rock and Sar	near		
Environment	Terrestrial	Barren_Rock and Sar	exclude		
Environment	Terrestrial	Tundra	include		
Environment	Terrestrial	Tundra	exclude		
Environment	Terrestrial	Ice and Snow	include		

Category	Class	SubClass	Word_type	S12	S13
Environment	Terrestrial	Terrestrial	exclude		
Environment	Terrestrial	Forests	include		
Environment	Terrestrial	Forests	exclude		
Environment	Terrestrial	Agroecosystems	include		
Environment	Terrestrial	Agroecosystems	near		
Environment	Terrestrial	Agroecosystems	exclude		
Environment	Terrestrial	Created Greenspace	include		
Environment	Terrestrial	Created Greenspace	near		
Environment	Terrestrial	Created Greenspace	exclude		
Environment	Terrestrial	Grasslands	include		
Environment	Terrestrial	Grasslands	exclude		
Environment	Terrestrial	Scrublands_Shrublan	include		
Environment	Terrestrial	Barren_Rock and Sar	include		
Environment	Terrestrial	Barren_Rock and Sar	near		
Environment	Terrestrial	Barren_Rock and Sar	exclude		
Environment	Terrestrial	Tundra	include		
Environment	Terrestrial	Tundra	exclude		
Environment	Terrestrial	Ice and Snow	include		

<table>
<tr><th>Category</th><th>Class</th><th>SubClass</th><th>Word_type</th><th>S1</th></tr>
<tr><td>Environment</td><td>Terrestrial</td><td>Ice and Snow</td><td>exclude</td><td>headwater|circaea|ssc|past|historic|carve|quaternary|depress|advanced|retreated|deposited|Pleistocene|years ago|epoch|ice age</td></tr>
<tr><td>Environment</td><td>Atmospheric</td><td>Atmosphere</td><td>include</td><td>atmospher|skies| sky |cloud|the sky|skyline|sky line|dark sky</td></tr>
<tr><td>Environment</td><td>Atmospheric</td><td>Atmosphere</td><td>near</td><td></td></tr>
<tr><td>Environment</td><td>Atmospheric</td><td>Atmosphere</td><td>exclude</td><td>rural.*atmosphere|changing.*climate|welcoming.*atmosphere|climate.*friendly|cloud.*berry|cloud.*water|water.*cloud|turbid|atmospheric
science|NOAA|Atmospheric Administration|deposit|atmospheric fall|sky lupine</td></tr>
<tr><td>FEGS</td><td>Air</td><td>Air</td><td>include</td><td>air</td></tr>
<tr><td>FEGS</td><td>Air</td><td>Air</td><td>near</td><td>asthma|respiratory|health|safe|clean|breath|protect|restore|resource|quality|discharge|emission|load|carried|carry|point source|emission|reduce|reduction</td></tr>
<tr><td>FEGS</td><td>Air</td><td>Air</td><td>exclude</td><td>cair|pair|fair|air yam|air
plant|chair|questionnaire|airborne|affair|millionaire|dairy|act|airshed|National.*Weather.*Service|impair|repair|topic|federal|belleair|tmdl|air.*act|ciceet|prai
|airport|clean.*air.*act|national.*air.*standard|airplane|airstrip|muck|regulat.*air|lair|Air Resource Specialist|Clean Air Bond|potato|dair|air force|www|Air
Resources Lab|air rifle|air condition|airfield|airboat|stair|air station|nair|bair|fair|air yam|air plant|air temp|hair|aircraft|f air</td></tr>
<tr><td>FEGS</td><td>Atmospheric Phenon</td><td>Atmospheric Phenon</td><td>include</td><td>sunrise|sunset|eclipse|sundown|rainbow|twilight</td></tr>
<tr><td>FEGS</td><td>Atmospheric Phenon</td><td>Atmospheric Phenon</td><td>near</td><td></td></tr>
<tr><td>FEGS</td><td>Atmospheric Phenon</td><td>Atmospheric Phenon</td><td>exclude</td><td>rodent|staff|presentation|created.*volunteer|15.*visitor|pollution|pollutant|climate|temperature|marina|island|provision|15.*visitor|trout|smelt|sunset
of|sunset on|biofog|to sunset|image|imaging|clouded|navigat|sunset of|sunset on|sunset in|sunsets on|sunsets in|infog|turbid|rainbow snake|after
sunset|rainbow star|rainbow surf|sunset at|before sunset|sunset cove|sunrise to|rainbow dart|rainbow dam</td></tr>
<tr><td>FEGS</td><td>Depredators and (Pest) Predators</td><td>Depredators and (Pest) Predators</td><td>include</td><td>biologic control|biocontrol|biological control|natural population control|natural check|natural control|biologic means|biological means|biologic methods|biological
methods|natural pathogen control|natural pest control|natural disease control|depredator|pest predator|natural insect control|natural pest control</td></tr>
<tr><td>FEGS</td><td>Depredators and (Pest) Predators</td><td>Depredators and (Pest) Predators</td><td>near</td><td></td></tr>
<tr><td>FEGS</td><td>Depredators and (Pest) Predators</td><td>Depredators and (Pest) Predators</td><td>exclude</td><td></td></tr>
<tr><td>FEGS</td><td>Fauna</td><td>Fauna</td><td>include</td><td>raptor|fauna|animal|mammal|wildlife|bird|owl|fowl|duck|sparrow|warbler|plover|falcon|osprey|hawk|eagle|pelican|geese|pidgeon|pigeon|turtle|tortoise|sea
l|whale|dolphin|porpoise|manatee|otter|coral|dragonfl|insect|beetle|entomolo|bug|deer|turkey|quail|waterfowl|duck|goose|reptile|alligator|amphibian|sala
mander|frog|toad|skink|bat|nutria|raccoon|hogs|rabbit|beaver|rookery|rookeries|butter|horseshoe crab</td></tr>
</table>

Category	Class	SubClass	Word_type	S2	S3
Environment	Terrestrial	Ice and Snow	exclude	plover|egret|goose|geese|ssc|owl|snowpatch|snowberry|snowy<br>orchid|snowline|tsnow|snowbird|lepus americanus	service|practice|choice|office|advice|twice|appendice|notice|device|lice|police| voice|auspice|price|sacrifice|justice|indices|vicente|globicephala|jamaicensis|tip<br>.*of.*the.*iceberg|ser.*vice|rice|prac.*tice|mice|septicemia|kice|nice|ssc|ciceet<br>|vice|dice|pice|past|historic|carve|quaternary|depress|advanced|retreated|dep<br>osited|Pleistocene|years ago|epoch|ice age|tice|fice|icen|iceplant|ice plant
Environment	Atmospheric	Atmosphere	include	TMDL|pollution|discharg|emission|wind	air
Environment	Atmospheric	Atmosphere	near	air|atmospher	
Environment	Atmospheric	Atmosphere	exclude	pair|fair|air yam|air<br>plant|chair|questionnaire|airborne|affair|millionaire|dairy|act|airshed|National.<br>*Weather.*Service|impair|repair|topic|federal|belleair|tmdl|air.*act|ciceet|prair<br>|airport|clean.*air.*act|national.*air.*standard|airplane|airstrip|muck|regulat.*ai<br>r|lair|Air Resource Specialist|Clean Air Bond|potato|dair|air force|www|Air<br>Resources Lab|air rifle|air condition|airfield|airboat|stair|air<br>station|nair|bair|fair|air yam|air plant|air temp|hair|aircraft|cair	pair|fair|air yam|air<br>plant|chair|questionnaire|airborne|affair|millionaire|dairy|act|airshed|National.<br>*Weather.*Service|impair|repair|topic|federal|belleair|tmdl|air.*act|ciceet|prair<br>|airport|clean.*air.*act|national.*air.*standard|airplane|airstrip|muck|regulat.*ai<br>r|lair|Air Resource Specialist|Clean Air Bond|potato|dair|air force|www|Air<br>Resources Lab|air rifle|air condition|airfield|airboat|stair|air<br>station|nair|bair|fair|air yam|air plant|air temp|hair|aircraft|cair
FEGS	Air	Air	include	air	air
FEGS	Air	Air	near	relax|enjoy|special|unique|preserv|bounty|bountiful|benefit|recreat|valuable|t<br>ours|value|restore|resource|protect|asset|special|amenit|desirable|pleasant|he<br>althy|hallmark|recreat|opportunit|experienc|enjoy|relax|families|family|children<br>|visitor|touris	specimen.*collect|sample.*collect|data.*collect|collect.*specimen|collect.*samp<br>e|collect.*data|specimen.*gather|sample.*gather|data.*gather|gather.*specimen<br>|gather.*sample|gather.*data|collect.*research|research.*collect|scien.*collect|c<br>ollect.*scien
FEGS	Air	Air	exclude	cair|pair|fair|air yam|air<br>plant|chair|questionnaire|airborne|affair|millionaire|dairy|act|airshed|National.<br>*Weather.*Service|impair|repair|topic|federal|belleair|tmdl|air.*act|ciceet|prair<br>|airport|clean.*air.*act|national.*air.*standard|airplane|airstrip|muck|regulat.*ai<br>r|lair|Air Resource Specialist|Clean Air Bond|potato|dair|bair|air force|www|Air<br>Resources Lab|air rifle|air condition|airfield|airboat|stair|air<br>station|nair|bair|fair|air yam|air plant|air temp|hair|aircraft|temperature<br>value|biomass value	cair|pair|fair|air yam|air<br>plant|chair|questionnaire|airborne|affair|millionaire|dairy|act|airshed|National.<br>*Weather.*Service|impair|repair|topic|federal|belleair|tmdl|air.*act|ciceet|prair<br>|airport|clean.*air.*act|national.*air.*standard|airplane|airstrip|muck|regulat.*ai<br>r|lair|Air Resource Specialist|Clean Air Bond|potato|dair|bair|air force|www|Air<br>Resources Lab|air rifle|air condition|airfield|airboat|stair|air<br>station|nair|bair|fair|air yam|air plant|air temp|hair|aircraft
FEGS	Atmospheric Phenon	Atmospheric Phenon	include		
FEGS	Atmospheric Phenon	Atmospheric Phenon	near		
FEGS	Atmospheric Phenon	Atmospheric Phenon	exclude		
FEGS	Depredators and (Pest) Predators	Depredators and (Pest) Predators	include	organic<br>approach|organic.*garden|garden.*organic|farm.*organic|organic.*farm|agric.*or<br>ganic|organic.*agric	agricultural environmental management|IPM|integrated pest management
FEGS	Depredators and (Pest) Predators	Depredators and (Pest) Predators	near	pest|predator	
FEGS	Depredators and (Pest) Predators	Depredators and (Pest) Predators	exclude	organic pesticide|agricultural runoff|organic material	uipm|hipm
FEGS	Fauna	Fauna	include	pelt|tanning|tannery|hunt.*meat|meat.*hunt|trap.*meat|meat.*trap|shoot.*hu<br>nt|hunt.*shoot|hunt.*kill|kill.*hunt|hunt.*meat|trap.*meat|meat.*hunt|meat.*t<br>rap|hunt.*edible|trap.*edible|edible.*hunt|edible.*trap|hunt.*consumpt|trap.*c<br>onsumpt|consumpt.*hunt|consumpt.*trap|hunt.*eaten|trap.*eaten|eaten.*hunt<br>|eaten.*trap|subsist.*hunt|hunt.*subsist|trap.*subsist|subsist.*trap|bat<br>band|bird band|hunting|hunter|trapping season|bird life|signs of<br>wildlife|birding|birdwatch|bird watch|whale watch|whale sight|birder|hunts	skin|hide| fur | furs |fur-bear|fur bear|furbear|meat|game

Category	Class	SubClass	Word_type	S4	S5
Environment	Terrestrial	Ice and Snow	exclude		
Environment	Atmospheric	Atmosphere	include		
Environment	Atmospheric	Atmosphere	near		
Environment	Atmospheric	Atmosphere	exclude		
FEGS	Air	Air	include	atmos	
FEGS	Air	Air	near	emission.*to|release.*to|reduce|reduction|discharge.*to	
FEGS	Air	Air	exclude	deposition|load.*from|cooperative atmosphere|rural atmosphere|welcoming atmosphere|load.*of|atmospheric load|to.*bay	
FEGS	Atmospheric Phenon	Atmospheric Phenon	include		
FEGS	Atmospheric Phenon	Atmospheric Phenon	near		
FEGS	Atmospheric Phenon	Atmospheric Phenon	exclude		
FEGS	Depredators and (Pest) Predators	Depredators and (Pest) Predators	include		
FEGS	Depredators and (Pest) Predators	Depredators and (Pest) Predators	near		
FEGS	Depredators and (Pest) Predators	Depredators and (Pest) Predators	exclude		
FEGS	Fauna	Fauna	include	game|hunting|hunts|hunter	bioturbation

<table>
<tr><th>Category</th><th>Class</th><th>SubClass</th><th>Word_type</th><th>S6</th><th>S7</th></tr>
<tr><td>Environment</td><td>Terrestrial</td><td>Ice and Snow</td><td>exclude</td><td></td><td></td></tr>
<tr><td>Environment</td><td>Atmospheric</td><td>Atmosphere</td><td>include</td><td></td><td></td></tr>
<tr><td>Environment</td><td>Atmospheric</td><td>Atmosphere</td><td>near</td><td></td><td></td></tr>
<tr><td>Environment</td><td>Atmospheric</td><td>Atmosphere</td><td>exclude</td><td></td><td></td></tr>
<tr><td>FEGS</td><td>Air</td><td>Air</td><td>include</td><td></td><td></td></tr>
<tr><td>FEGS</td><td>Air</td><td>Air</td><td>near</td><td></td><td></td></tr>
<tr><td>FEGS</td><td>Air</td><td>Air</td><td>exclude</td><td></td><td></td></tr>
<tr><td>FEGS</td><td>Atmospheric Phenon</td><td>Atmospheric Phenon</td><td>include</td><td></td><td></td></tr>
<tr><td>FEGS</td><td>Atmospheric Phenon</td><td>Atmospheric Phenon</td><td>near</td><td></td><td></td></tr>
<tr><td>FEGS</td><td>Atmospheric Phenon</td><td>Atmospheric Phenon</td><td>exclude</td><td></td><td></td></tr>
<tr><td>FEGS</td><td>Depredators and (Pest) Predators</td><td>Depredators and (Pest) Predators</td><td>include</td><td></td><td></td></tr>
<tr><td>FEGS</td><td>Depredators and (Pest) Predators</td><td>Depredators and (Pest) Predators</td><td>near</td><td></td><td></td></tr>
<tr><td>FEGS</td><td>Depredators and (Pest) Predators</td><td>Depredators and (Pest) Predators</td><td>exclude</td><td></td><td></td></tr>
<tr><td>FEGS</td><td>Fauna</td><td>Fauna</td><td>include</td><td>raptor|fauna|animal|mammal|wildlife|bird|owl|fowl|duck|sparrow|warbler|plover|falcon|osprey|hawk|eagle|pelican|geese|pidgeon|pigeon|turtle|tortoise|seal|whale|dolphin|porpoise|manatee|otter|coral|dragonfl|insect|beetle|entomolo|bug|deer|turkey|quail|waterfowl|duck|goose|reptile|alligator|amphibian|salamander|frog|toad|skink|bat|nutria|raccoon|hogs|rabbit|beaver|rookery|rookeries|butter|horseshoe crab</td><td>bait</td></tr>
</table>

Category	Class	SubClass	Word_type	S8	S9
Environment	Terrestrial	Ice and Snow	exclude		
Environment	Atmospheric	Atmosphere	include		
Environment	Atmospheric	Atmosphere	near		
Environment	Atmospheric	Atmosphere	exclude		
FEGS	Air	Air	include		
FEGS	Air	Air	near		
FEGS	Air	Air	exclude		
FEGS	Atmospheric Phenon	Atmospheric Phenon	include		
FEGS	Atmospheric Phenon	Atmospheric Phenon	near		
FEGS	Atmospheric Phenon	Atmospheric Phenon	exclude		
FEGS	Depredators and (Pest) Predators	Depredators and (Pest) Predators	include		
FEGS	Depredators and (Pest) Predators	Depredators and (Pest) Predators	near		
FEGS	Depredators and (Pest) Predators	Depredators and (Pest) Predators	exclude		
FEGS	Fauna	Fauna	include	species of concern|species of special concern|endangered.*species|threatened.*species|species.*endangered|species.*threatened|protect.*species	marine.*species|terrestrial.*species|aquatic.*species

Category	Class	SubClass	Word_type	S10	S11
Environment	Terrestrial	Ice and Snow	exclude		
Environment	Atmospheric	Atmosphere	include		
Environment	Atmospheric	Atmosphere	near		
Environment	Atmospheric	Atmosphere	exclude		
FEGS	Air	Air	include		
FEGS	Air	Air	near		
FEGS	Air	Air	exclude		
FEGS	Atmospheric Phenon	Atmospheric Phenon	include		
FEGS	Atmospheric Phenon	Atmospheric Phenon	near		
FEGS	Atmospheric Phenon	Atmospheric Phenon	exclude		
FEGS	Depredators and (Pest) Predators	Depredators and (Pest) Predators	include		
FEGS	Depredators and (Pest) Predators	Depredators and (Pest) Predators	near		
FEGS	Depredators and (Pest) Predators	Depredators and (Pest) Predators	exclude		
FEGS	Fauna	Fauna	include		

Category	Class	SubClass	Word_type	S12	S13
Environment	Terrestrial	Ice and Snow	exclude		
Environment	Atmospheric	Atmosphere	include		
Environment	Atmospheric	Atmosphere	near		
Environment	Atmospheric	Atmosphere	exclude		
FEGS	Air	Air	include		
FEGS	Air	Air	near		
FEGS	Air	Air	exclude		
FEGS	Atmospheric Phenon	Atmospheric Phenon	include		
FEGS	Atmospheric Phenon	Atmospheric Phenon	near		
FEGS	Atmospheric Phenon	Atmospheric Phenon	exclude		
FEGS	Depredators and (Pest) Predators	Depredators and (Pest) Predators	include		
FEGS	Depredators and (Pest) Predators	Depredators and (Pest) Predators	near		
FEGS	Depredators and (Pest) Predators	Depredators and (Pest) Predators	exclude		
FEGS	Fauna	Fauna	include		

<table>
<tr><th>Category</th><th>Class</th><th>SubClass</th><th>Word_type</th><th>S1</th></tr>
<tr><td>FEGS</td><td>Fauna</td><td>Fauna</td><td>near</td><td>accomodat|activit|aesthetic|ameniable|amenit|appreciat|artisan|artist|asset|attract|beaut|beneficial|benefit|bequest|biodiversity|bountiful|bounty|business|b [illegible]
uy|care|caring|charisma|cherish|children|collect|comfort|commercial|commodities|commodity|goods|concern|conserv|corporation|critical|dealer|delicious|des [illegible]
ired|desirable|destination|econom|edible|educat|encourag|endangered|enhance|enhancing|enjoy|encounter|enthusiast|experienc|exploit|extract|families|favo [illegible]
rable|favor|feel|festiv|ceremon| fur | furs |fur-bear|fur
bear|furbear|future|gather|generation|hallmark|harvest|healthy|heritage|hide|hunt|income|indigenous.*people|indian population|indian
people|indigenous.*person|industry|initiative|inspir|interest|job|learn|legacy|leisure|livelihood|market|meat|native.*person|native
american|observ|opportunit|participant|participate|pelt|pick|pleasant|popular|preserv|products|profession|profit|promote|promotion|purpose|protect|quality| [illegible]
.*life|rare|recreat|relax|resource|restor|sale|save|saving|sell|sight|skins|special|specimen|spectacular|sport|steward|subsist|sustenance|threatened|together| [illegible]
tours|trade|trading|sustainabl|tradition|treasure|tribal|tribe|unique|valuable|value|valuing|view|visit|visitor|watch|well suited|well-
suited|wonder|allure|alluring|mystique|option value|lifeblood|jewel|extraordinary|excellent|amaze|amazing|stunning|majestic|spectacular</td></tr>
<tr><td>FEGS</td><td>Fauna</td><td>Fauna</td><td>exclude</td><td>toad flax|goosefoot|snowbird|geoduck|domestic animal|production of animal|production of plants and animal|turtle cove|turtleback|animal
production|insecticide|otter point creek|bullfrog creek|buttercup|buttermilk|butterwort|abutter|butter clam|rookerybay|rookery
bay|batbing|bato|batk|tbat|batsto|bata|abat|bate|bath|bati|batu|blackbird creek|dairy|c-
hawk|hawken|redbug|lowl|batr|muskin|sealant|sealing|sealed|hawkey|hawkin|hawken|knowl|combat|butterfish|butterfly orchid|manatee county|animal
feeding|animal waste|CAFO|batt|agency|askin|pickerel|by-product|byproduct|by
product|chid|entrap|strap|trap.*gas|gas.*trap|sediment.*trap|contam.*trap|nutrient.*trap|pollut.*trap|trap.*contam|trap.*nutrient|trap.*pollut|sand.*trap|tra [illegible]
p.*sand|trap.*sediment|litter trap|fish trap|especial|power.*generation|electric.*generation|generation plant|generation facilit|laboratory observ|field observ|lab [illegible]
observ|special issue|meadow beauty|river beauty|beautiful jacob|spring beauty|beautyberry|carex|carey|personal care|careo|aquarium care|lawn
care|careful|exotic|extrapolat|askin|pickerel|invasive species|iskin|nuisance species|overview|in view of|over-view|over-
view|peltandra|pelti|pelto|apelt|peltu|pelta|pelte|provid.* no clue|provid.* notice|provid.* basis|provid.*expertise|rarely|temperature value|biomass
value|nonprofit|non-profit|refur|review|bayview|sensitive fern|shunt|huntley|huntington|hunterdon|sking|salem|mysella|economus|newmarket|skink|skin
lesion|skin condition|strap|sulfur|specialist|tradeoff|trade-off|transport|workshop</td></tr>
</table>

Category	Class	SubClass	Word_type	S2	S3
FEGS	Fauna	Fauna	near		exploit|hunt|trap|harvest|company|corporation|trade
FEGS	Fauna	Fauna	exclude	scavenger hunt|fish.*trap|trap.*fish|agency|askin|pickerel|by-product|byproduct|by product|chid|entrap|strap|trap.*gas|gas.*trap|sediment.*trap|contam.*trap|nutrient.*trap|pollut.*trap|trap.*contam|trap.*nutrient|trap.*pollut|sand.*trap|trap.*sand|trap.*sediment|litter trap|fish trap|especial|power.*generation|electric.*generation|generation plant|generation facilit|laboratory observ|field observ|lab observ|special issue|meadow beauty|river beauty|beautiful jacob|spring beauty|beautyberry|carex|carey|personal care|careo|aquarium care|lawn care|careful|exotic|extrapolat|askin|pickerel|invasive species|iskin|nuisance species|overview|in view of|over-view|over-view|peltandra|pelti|pelto|apelt|peltu|pelta|pelte|provid.* no clue|provid.* notice|provid.* basis|provid.*expertise|rarely|temperature value|biomass value|nonprofit|non-profit|refur|review|bayview|sensitive fern|shunt|huntley|huntington|hunterdon|sking|salem|mysella|economus|new market|skink|skin lesion|skin condition|strap|sulfur|specialist|tradeoff|trade-off|transport|workshop	agency|askin|pickerel|by-product|byproduct|by product|chid|entrap|strap|trap.*gas|gas.*trap|sediment.*trap|contam.*trap|nutrient.*trap|pollut.*trap|trap.*contam|trap.*nutrient|trap.*pollut|sand.*trap|trap.*sand|trap.*sediment|litter trap|fish trap|especial|power.*generation|electric.*generation|generation plant|generation facilit|laboratory observ|field observ|lab observ|special issue|meadow beauty|river beauty|beautiful jacob|spring beauty|beautyberry|carex|carey|personal care|careo|aquarium care|lawn care|careful|exotic|extrapolat|askin|pickerel|invasive species|iskin|nuisance species|overview|in view of|over-view|over-view|peltandra|pelti|pelto|apelt|peltu|pelta|pelte|provid.* no clue|provid.* notice|provid.* basis|provid.*expertise|rarely|temperature value|biomass value|nonprofit|non-profit|refur|review|bayview|sensitive fern|shunt|huntley|huntington|hunterdon|sking|salem|mysella|economus|new market|skink|skin lesion|skin condition|strap|sulfur|specialist|tradeoff|trade-off|transport|workshop

Category	Class	SubClass	Word_type	S4	S5
FEGS	Fauna	Fauna	near	wild|harvest|raptor|fauna|animal|mammal|wildlife|bird|owl|fowl|duck|sparrow|warbler|plover|falcon|osprey|hawk|eagle|pelican|geese|pidgeon|pigeon|turtle|tortoise|seal|whale|dolphin|porpoise|manatee|otter|coral|dragonfl|insect|beetle|entomolo|bug|deer|turkey|quail|waterfowl|duck|goose|reptile|alligator|amphibian|salamander|frog|toad|skink|bat|nutria|raccoon|hogs|rabbit|beaver|rookery|rookeries|butter|marine.*species|terrestrial.*species|aquatic.*species|horseshoe crab|recreation|sport|resource	
FEGS	Fauna	Fauna	exclude	shunt|huntley|huntington|transport|scavenger hunt	

Category	Class	SubClass	Word_type	S6	S7
FEGS	Fauna	Fauna	near	essential habitat|critical habitat|feeding ground	worm
FEGS	Fauna	Fauna	exclude	toad flax|goosefoot|snowbird|geoduck|domestic animal|production of animal|production of plants and animal|turtle cove|turtleback|animal production|otter point creek|bullfrog creek|insecticide|buttercup|buttermilk|butterwort|abutter|butter clam|rookerybay|rookery bay|batbing|bato|batk|tbat|batsto|bata|abat|bate|bath|bati|batu|blackbird creek|dairy|c-hawk|hawken|redbug|lowl|batr|muskin|sealant|sealing|sealed|hawkey|hawkin|hawken|knowl|combat|butterfish|butterfly orchid|manatee county|animal feeding|animal waste|CAFO|batt|agency|askin|pickerel|by-product|byproduct|by product|chid|entrap|strap|gas.*trap|trap.*gas|sediment.*trap|contam.*trap|nutrient.*trap|pollut.*trap|trap.*contam|trap.*nutrient|trap.*pollut|sand.*trap|trap.*sand|trap.*sediment|litter trap|fish trap|especial|power.*generation|electric.*generation|generation plant|generation facilit|laboratory observ|field observ|lab observ|special issue|meadow beauty|river beauty|beautiful jacob|spring beauty|beautyberry|carex|carey|personal care|careo|aquarium care|lawn care|careful|exotic|extrapolat|askin|pickerel|invasive species|iskin|nuisance species|overview|in view of|over-view|over-view|peltandra|pelti|pelto|apelt|peltu|pelta|pelte|provid.* no clue|provid.* notice|provid.* basis|provid.*expertise|rarely|temperature value|biomass value|nonprofit|non-profit|refur|review|bayview|sensitive fern|shunt|huntley|huntington|hunterdon|sking|salem|mysella|economus|new market|skink|skin lesion|skin condition|strap|sulfur|specialist|tradeoff|trade-off|transport|workshop	

<table>
<tr><th>Category</th><th>Class</th><th>SubClass</th><th>Word_type</th><th>S8</th><th>S9</th></tr>
<tr><td>FEGS</td><td>Fauna</td><td>Fauna</td><td>near</td><td></td><td>accomodat|activit|aesthetic|ameniable|amenit|appreciat|artisan|artist|asset|attract|beaut|beneficial|benefit|bequest|biodiversity|bountiful|bounty|business|buy|care|caring|charisma|cherish|children|collect|comfort|commercial|commodities|commodity|goods|concern|conserv|corporation|critical|dealer|delicious|desired|desirable|destination|econom|edible|educat|encourag|endangered|enhance|enhancing|enjoy|encounter|enthusiast|experienc|exploit|extract|families|favorable|favor|feel|festiv|ceremon| fur | furs |fur-bear|fur bear|furbear|future|gather|generation|hallmark|harvest|healthy|heritage|hide|hunt|income|indigenous.*people|indian population|indian people|indigenous.*person|industry|initiative|inspir|interest|job|learn|legacy|leisure|livelihood|market|meat|native.*person|native american|observ|opportunit|participant|participate|pelt|pick|pleasant|popular|preserv|products|profession|profit|promote|promotion|purpose|protect|quality.*life|rare|recreat|relax|resource|restor|sale|save|saving|sell|sight|skins|special|specimen|spectacular|sport|steward|subsist|sustenance|threatened|together|tours|trade|trading|sustainabl|tradition|treasure|tribal|tribe|unique|valuable|value|valuing|view|visit|visitor|watch|well suited|well-suited|wonder|allure|alluring|mystique|option value|lifeblood|jewel|extraordinary|excellent|amaze|amazing|stunning|majestic|spectacular</td></tr>
<tr><td>FEGS</td><td>Fauna</td><td>Fauna</td><td>exclude</td><td>flora|plant|flower|root|grass|kelp|seaweed|vegetation|mangrove|reed|cypress|sphagnum|cattail|moss|palm|berry|berries|seeds|fruit|nuts|tree|sedge|moss|lichen|forest|pine|oak|cypress| elm| ash|maple|hickory|hardwood|cedar</td><td>flora|plant|flower|root|grass|kelp|seaweed|vegetation|mangrove|reed|cypress|sphagnum|cattail|moss|palm|berry|berries|seeds|fruit|nuts|tree|sedge|moss|lichen|forest|pine|oak|cypress| elm| ash|maple|hickory|hardwood|cedar|goosefoot|snowbird|geoduck|domestic animal|production of animal|production of plants and animal|turtle cove|turtleback|animal production|insecticide|otter point creek|bullfrog creek|buttercup|buttermilk|butterwort|abutter|butter clam|rookerybay|rookery bay|batbing|bato|batk|tbat|batsto|bata|abat|bate|bath|bati|batu|blackbird creek|dairy|c-hawk|hawken|redbug|lowl|batr|muskin|sealant|sealing|sealed|hawkey|hawkin|hawken|knowl|combat|butterfish|butterfly orchid|manatee county|animal feeding|animal waste|CAFO|batt|agency|askin|pickerel|by-product|byproduct|by product|chid|entrap|strap|trap.*gas|gas.*trap|sediment.*trap|contam.*trap|nutrient.*trap|pollut.*trap|trap.*contam|trap.*nutrient|trap.*pollut|sand.*trap|trap.*sand|trap.*sediment|litter trap|fish trap|especial|power.*generation|electric.*generation|generation plant|generation facilit|laboratory observ|field observ|lab observ|special issue|meadow beauty|river beauty|beautiful jacob|spring beauty|beautyberry|carex|carey|personal care|careo|aquarium care|lawn care|careful|exotic|extrapolat|askin|pickerel|invasive species|iskin|nuisance species|overview|in view of|over-view|over-view|peltandra|pelti|pelto|apelt|peltu|pelta|pelte|provid.* no clue|provid.* notice|provid.* basis|provid.*expertise|rarely|temperature value|biomass value|nonprofit|non-profit|refur|review|bayview|sensitive fern|shunt|huntley|huntington|hunterdon|sking|salem|mysella|economus|new market|skink|skin lesion|skin condition|strap|sulfur|specialist|tradeoff|trade-off|transport|workshop</td></tr>
</table>

Category	Class	SubClass	Word_type	S10	S11
FEGS	Fauna	Fauna	near		
FEGS	Fauna	Fauna	exclude		

Category	Class	SubClass	Word_type	S12	S13
FEGS	Fauna	Fauna	near		
FEGS	Fauna	Fauna	exclude		

Category	Class	SubClass	Word_type	S1
FEGS	FEGS	FEGS	include	resource|value|valuable|trading|mitigat|goods|econom|public access|conserv|protect|tourism|tourist|recreation|livelihood|visitor use|stewards|bounty|bountiful
FEGS	FEGS	FEGS	near	ecosystem|environment|natur|estuar|renewable|bay|ecological|field|aquatic|marine|water|park|coast|shore|land|wetland|the reserve|floodplain|biological|basin|the region|our region|the area|our area
FEGS	FEGS	FEGS	exclude	Department|sewer service|office|division|commission|bay scallop|lander|landing|landmark|southland|parking|parker|economical|protection agency|environmentally|temperature value|biomass value|the regional|valuable tool
FEGS	Fiber	Fiber	include	fiber
FEGS	Fiber	Fiber	near	provid|service|provision|collect|gather|environment|ecosystem|natur|subsist|use|benefit|product|industr|mill|harvest|pottery|goods
FEGS	Fiber	Fiber	exclude	fiberglass|issues|journal|national.*park.*service|elderberry|15.*visitor|silvicultur|cause|focused|optic|million|millimeter|millennia|milliliter|millirem|milligram|miller|mill.*creek|reproduc|productiv|fiber content|cotton
FEGS	Fish	Fish	include	fish|seahorse|aquaria|aquarium|seafood|salmon|shrimp|oyster|pike|crab|lobster|mullet|mussel|bass|herring|grouper|snapper|alewife|flounder|abalone|scallop|clam|geoduck|mollusk
FEGS	Fish	Fish	near	indust|commercial|recreat|artisan|harvest|collect|gather|stock|sustain|product|manag.*future|manag.*sustain|manag.*stock|catch|dig|nets|traps

Category	Class	SubClass	Word_type	S2	S3
FEGS	FEGS	FEGS	include	beneficial use|living resource|natural feature|natural resource|ecosystem service|ecological service|economic use|commercial use|recreational use|cultural.*resource|traditional use|sustainable tourism	
FEGS	FEGS	FEGS	near		
FEGS	FEGS	FEGS	exclude	department	
FEGS	Fiber	Fiber	include	salt hay|reed|grass|marsh hay|turpentine	vegetative cutting|branch cutting
FEGS	Fiber	Fiber	near	subsist|mill|paper|fabric|textile|pottery|goods	
FEGS	Fiber	Fiber	exclude	grassroot|grass-root|paper mulberry|sea grass|freed|mcreedy|sea-grass|eelgrass|oyster.*grass|seagrass|grassland|sea-grass|decreed|breed|agreed|haynes|hay.*barn|hayden|cottonwood|freed|cottontail|reedgrass|hayan|issues|journal|scotton|invasive|non.*native|nonnative|national.*park.*service|elderberry|15.*visitor|cause|focused|million|millimeter|millennia|milliliter|millirem|milligram|miller|mill.*creek|millet|shrimp|data|dredg|hopper|farm|government.*industr	
FEGS	Fish	Fish	include	fish|seahorse|aquaria|aquarium|seafood|shrimp|salmon|oyster|pike|crab|lobster|mullet|mussel|bass|herring|grouper|snapper|alewife|flounder|abalone|scallop|clam|geoduck	bait
FEGS	Fish	Fish	near	accomodat|activit|aesthetic|ameniable|amenit|appreciat|artisan|artist|asset|attract|beaut|beneficial|benefit|bequest|biodiversity|bountiful|bounty|business|buy|care|caring|charisma|cherish|children|collect|comfort|commercial|commodities|commodity|goods|concern|conserv|corporation|critical|dealer|delicious|desired|desirable|destination|econom|edible|educat|encourag|endangered|enhance|enhancing|enjoy|encounter|enthusiast|experienc|exploit|extract|families|favorable|favor|feel|festiv|ceremon| fur | furs |fur-bear|fur bear|furbear|future|gather|generation|hallmark|harvest|healthy|heritage|hide|hunt|income|indigenous.*people|indian population|indian people|indigenous.*person|industry|initiative|inspir|interest|job|learn|legacy|leisure|livelihood|market|meat|native.*person|native american|observ|opportunit|participant|participate|pelt|pick|pleasant|popular|preserv|products|profession|profit|promote|promotion|purpose|protect|quality.*life|rare|recreat|relax|resource|restor|sale|save|saving|sell|sight|skins|special|specimen|spectacular|sport|steward|subsist|sustenance|threatened|together|tours|trade|trading|sustainabl|tradition|treasure|tribal|tribe|unique|valuable|value|valuing|valuing|view|visit|visitor|watch|well suited|well-suited|wonder|allure|alluring|mystique|option value|lifeblood|jewel|extraordinary|excellent|amaze|amazing|stunning|majestic|spectacular	fish|shrimp

Category	Class	SubClass	Word_type	S4	S5
FEGS	FEGS	FEGS	include		
FEGS	FEGS	FEGS	near		
FEGS	FEGS	FEGS	exclude		
FEGS	Fiber	Fiber	include		
FEGS	Fiber	Fiber	near		
FEGS	Fiber	Fiber	exclude		
FEGS	Fish	Fish	include	fish|seahorse|aquaria|aquarium|seafood|salmon|shrimp|oyster|pike|crab|lobster|mullet|mussel|bass|herring|grouper|snapper|alewife|flounder|abalone|scallop|clam|geoduck	seafood|fishery|fisheries|angler|angling|shellfish garden|oyster garden|shellfish farm|oyster farm|fishing|fisherman|fishermen|shellfish
FEGS	Fish	Fish	near	essential habitat|critical habitat|feeding ground	

Category	Class	SubClass	Word_type	S6	S7
FEGS	FEGS	FEGS	include		
FEGS	FEGS	FEGS	near		
FEGS	FEGS	FEGS	exclude		
FEGS	Fiber	Fiber	include		
FEGS	Fiber	Fiber	near		
FEGS	Fiber	Fiber	exclude		
FEGS	Fish	Fish	include	wildstock|wild.*stock|stock.*wild|wild.*catch|catch.*wild|wild.*caught|caught.*wild	
FEGS	Fish	Fish	near	fish|seahorse|aquaria|aquarium|seafood|salmon|shrimp|oyster|pike|crab|lobster|mullet|mussel|bass|herring|grouper|snapper|alewife|flounder|abalone|scallop|clam|geoduck|lake|river|stream	

Category	Class	SubClass	Word_type	S8	S9
FEGS	FEGS	FEGS	include		
FEGS	FEGS	FEGS	near		
FEGS	FEGS	FEGS	exclude		
FEGS	Fiber	Fiber	include		
FEGS	Fiber	Fiber	near		
FEGS	Fiber	Fiber	exclude		
FEGS	Fish	Fish	include		
FEGS	Fish	Fish	near		

Category	Class	SubClass	Word_type	S10	S11
FEGS	FEGS	FEGS	include		
FEGS	FEGS	FEGS	near		
FEGS	FEGS	FEGS	exclude		
FEGS	Fiber	Fiber	include		
FEGS	Fiber	Fiber	near		
FEGS	Fiber	Fiber	exclude		
FEGS	Fish	Fish	include		
FEGS	Fish	Fish	near		

Category	Class	SubClass	Word_type	S12	S13
FEGS	FEGS	FEGS	include		
FEGS	FEGS	FEGS	near		
FEGS	FEGS	FEGS	exclude		
FEGS	Fiber	Fiber	include		
FEGS	Fiber	Fiber	near		
FEGS	Fiber	Fiber	exclude		
FEGS	Fish	Fish	include		
FEGS	Fish	Fish	near		

<table>
<tr><th>Category</th><th>Class</th><th>SubClass</th><th>Word_type</th><th>S1</th></tr>
<tr><td>FEGS</td><td>Fish</td><td>Fish</td><td>exclude</td><td>oyster shells|ambassador|salmon falls|indig|reclam|clami|horseshoe crab|oystercatcher|agency|askin|pickerel|by-product|byproduct|by
product|chid|entrap|strap|sediment.*trap|contam.*trap|nutrient.*trap|pollut.*trap|trap.*contam|trap.*nutrient|trap.*pollut|sand.*trap|trap.*sand|trap.*sedim
ent|litter trap|especial|power.*generation|electric.*generation|generation plant|generation facilit|laboratory observ|field observ|lab observ|special issue|meadow
beauty|river beauty|beautiful jacob|spring beauty|beautyberry|carex|carey|personal care|careo|aquarium care|lawn
care|careful|exotic|extrapolat|askin|pickerel|invasive species|iskin|nuisance species|overview|in view of|over-view|over-
view|peltandra|pelti|pelto|apelt|peltu|pelta|pelte|provid.* no clue|provid.* notice|provid.* basis|provid.*expertise|rarely|temperature value|biomass
value|nonprofit|non-profit|refur|review|bayview|sensitive fern|shunt|huntley|huntington|hunterdon|sking|salem|mysella|economus|newmarket|skink|skin
lesion|skin condition|strap|sulfur|specialist|tradeoff|trade-off|transport|workshop|clami|pickerel|oyster creek|spike|kingfisher|fort fisher|fish river</td></tr>
<tr><td>FEGS</td><td>Flora</td><td>Flora</td><td>include</td><td>flora|plant|flower|root|grass|kelp|seaweed|vegetation|mangrove|reed|cypress|sphagnum|cattail|moss|palm|berry|berries|seeds|fruit|nuts|tree|sedge|moss|l
ichen|pine|oak|cypress| elm| ash|maple|hickory|hardwood|cedar|daffodil|tulip</td></tr>
<tr><td>FEGS</td><td>Flora</td><td>Flora</td><td>near</td><td>accomodat|activit|aesthetic|ameniable|amenit|appreciat|artisan|artist|asset|attract|beaut|beneficial|benefit|bequest|biodiversity|bountiful|bounty|business|b
uy|care|caring|charisma|cherish|children|collect|comfort|commercial|commodities|commodity|goods|concern|conserv|corporation|critical|dealer|delicious|des
ired|desirable|destination|econom|edible|educat|encourag|endangered|enhance|enhancing|enjoy|encounter|enthusiast|experienc|exploit|extract|families|favo
rable|favor|feel|festiv|ceremon| fur | furs |fur-bear|fur
bear|furbear|future|gather|generation|hallmark|harvest|healthy|heritage|hide|hunt|income|indigenous.*people|indian population|indian
people|indigenous.*person|industry|initiative|inspir|interest|job|learn|legacy|leisure|livelihood|market|meat|native.*person|native
american|observ|opportunit|participant|participate|pelt|pick|pleasant|popular|preserv|products|profession|profit|promote|promotion|purpose|protect|quality
.*life|rare|recreat|relax|resource|restor|sale|save|saving|sell|sight|skins|special|specimen|spectacular|sport|steward|subsist|sustenance|threatened|together|
tours|trade|trading|sustainabl|tradition|treasure|tribal|tribe|unique|valuable|value|valuing|valuing|view|visit|visitor|watch|well suited|well-
suited|wonder|allure|alluring|mystique|option value|lifeblood|jewel|extraordinary|excellent|amaze|amazing|stunning|majestic|spectacular</td></tr>
</table>

Category	Class	SubClass	Word_type	S2	S3
FEGS	Fish	Fish	exclude	oyster shells|reclam|clami|horseshoe crab|oystercatcher|agency|askin|pickerel|by-product|byproduct|by product|chid|entrap|strap|sediment.*trap|contam.*trap|nutrient.*trap|pollut.*trap|trap.*contam|trap.*nutrient|trap.*pollut|sand.*trap|trap.*sand|trap.*sediment|litter trap|especial|power.*generation|electric.*generation|generation plant|generation facilit|laboratory observ|field observ|lab observ|special issue|meadow beauty|river beauty|beautiful jacob|spring beauty|beautyberry|carex|carey|personal care|careo|aquarium care|lawn care|careful|exotic|extrapolat|askin|pickerel|invasive species|iskin|nuisance species|overview|in view of|over-view|over-view|peltandra|pelti|pelto|apelt|peltu|pelta|pelte|provid.* no clue|provid.* notice|provid.* basis|provid.*expertise|rarely|temperature value|biomass value|nonprofit|non-profit|refur|review|bayview|sensitive fern|shunt|huntley|huntington|hunterdon|sking|salem|mysella|economus|new market|skink|skin lesion|skin condition|strap|sulfur|specialist|tradeoff|trade-off|transport|workshop|clami|pickerel|oyster creek|kingfisher|fort fisher|fish river	
FEGS	Flora	Flora	include	cranberr|berry|berries|seeds|fruit|nuts|edible.*plant	sav
FEGS	Flora	Flora	near	wild|collect|gather|harvest|pick|subsist	accomodat|activit|aesthetic|ameniable|amenit|appreciat|artisan|artist|asset|attract|beaut|beneficial|benefit|bequest|biodiversity|bountiful|bounty|business|buy|care|caring|charisma|cherish|children|collect|comfort|commercial|commodities|commodity|goods|concern|conserv|corporation|critical|dealer|delicious|desired|desirable|destination|econom|edible|educat|encourag|endangered|enhance|enhancing|enjoy|encounter|enthusiast|experienc|exploit|extract|families|favorable|favor|feel|festiv|ceremon| fur | furs |fur-bear|fur bear|furbear|future|gather|generation|hallmark|harvest|healthy|heritage|hide|hunt|income|indigenous.*people|indian population|indian people|indigenous.*person|industry|initiative|inspir|interest|job|learn|legacy|leisure|livelihood|market|meat|native.*person|native american|observ|opportunit|participant|participate|pelt|pick|pleasant|popular|preserv|products|profession|profit|promote|promotion|purpose|protect|quality.*life|rare|recreat|relax|resource|restor|sale|sell|sight|skins|special|specimen|spectacular|sport|steward|subsist|sustenance|threatened|together|tours|trade|trading|sustainabl|tradition|treasure|tribal|tribe|unique|valuable|value|valuing|view|visit|visitor|watch|well suited|well-suited|wonder|allure|alluring|mystique|option value|lifeblood|jewel|extraordinary|excellent|amaze|amazing|stunning|majestic|spectacular

Category	Class	SubClass	Word_type	S4	S5
FEGS	Fish	Fish	exclude	kingfisher|fort fisher|fish river	
FEGS	Flora	Flora	include	species of concern|species of special concern|endangered.*species|threatened.*species|species.*endangered|species.*threatened|protect.*species	native.*plant|plant.*native|native.*flora|flora.*native
FEGS	Flora	Flora	near		

Category	Class	SubClass	Word_type	S6	S7
FEGS	Fish	Fish	exclude	livestock|wildlife|wild animal|spike|clami	
FEGS	Flora	Flora	include	forestry	marine.*species|terrestrial.*species|aquatic.*species
FEGS	Flora	Flora	near		accomodat|activit|aesthetic|ameniable|amenit|appreciat|artisan|artist|asset|attract|beaut|beneficial|benefit|bequest|biodiversity|bountiful|bounty|business|buy|care|caring|charisma|cherish|children|collect|comfort|commercial|commodities|commodity|goods|concern|conserv|corporation|critical|dealer|delicious|desired|desirable|destination|econom|edible|educat|encourag|endangered|enhance|enhancing|enjoy|encounter|enthusiast|experienc|exploit|extract|families|favorable|favor|feel|festiv|ceremon| fur | furs |fur-bear|fur bear|furbear|future|gather|generation|hallmark|harvest|healthy|heritage|hide|hunt|income|indigenous.*people|indian population|indian people|indigenous.*person|industry|initiative|inspir|interest|job|learn|legacy|leisure|livelihood|market|meat|native.*person|native american|observ|opportunit|participant|participate|pelt|pick|pleasant|popular|preserv|products|profession|profit|promote|promotion|purpose|protect|quality.*life|rare|recreat|relax|resource|restor|sale|save|saving|sell|sight|skins|special|specimen|spectacular|sport|steward|subsist|sustenance|threatened|together|tours|trade|trading|sustainabl|tradition|treasure|tribal|tribe|unique|valuable|value|valuing|valuing|view|visit|visitor|watch|well suited|well-suited|wonder|allure|alluring|mystique|option value|lifeblood|jewel|extraordinary|excellent|amaze|amazing|stunning|majestic|spectacular

Category	Class	SubClass	Word_type	S8	S9
FEGS	Fish	Fish	exclude		
FEGS	Flora	Flora	include		
FEGS	Flora	Flora	near		

Category	Class	SubClass	Word_type	S10	S11
FEGS	Fish	Fish	exclude		
FEGS	Flora	Flora	include		
FEGS	Flora	Flora	near		

Category	Class	SubClass	Word_type	S12	S13
FEGS	Fish	Fish	exclude		
FEGS	Flora	Flora	include		
FEGS	Flora	Flora	near		

Category	Class	SubClass	Word_type	S1
FEGS	Flora	Flora	exclude	processing plant|aguirre plant|pauciflora|plant.*upgrade|upgrade.*plant|planted|planting|plantation|grassroot|grass-root|braintree|package<br>plant|transplant|power plant|carene|treatment plant|invasive plant|carex|carey|personal care|careo|aquarium care|lawn<br>care|careful|decreed|breed|hayden|cottonwood|freed|reedgrass|agreed|elderberry|cash|category|sewage|sanitary|acquatic|management.*area|fish|wash|dnr<br>|mcbp|trash|shoreline|pineda|pinellas|legend|pine.*island|pine.*barren|vash|elmer|elm st|ashe|coal<br>ash|mash|sash|cash|bash|overwhelm|oakdale|oakley|croak|coal.*ash|delmarva|fashion|pineland|barrier.*island|water.*pollution|soak|pine.*beach|over.*whel<br>med|barrier.*beach|ashore|sand.*pine|shorebird|percent|metals|oakland|silverside|hash|develm|hairstreak|crash|pine.*hills|ocean.*pine|oak.*harbor|oak.*rid<br>ge|table|figure|characterized.*as|cdmo|unacceptable|seed|runoff|pollut|data|erosion|seem|timber|logging|logger|lumber|dash|Wash|eash|lash|mash|rash|u<br>ash|especial|power.*generation|electric.*generation|generation plant|generation facilit|laboratory observ|field observ|lab observ|special issue|meadow<br>beauty|river beauty|beautiful jacob|spring<br>beauty|careful|specialist|Department|mossler|thompson|sedge.*wren|sedge.*island|sea.*sedge|subtidal|submergent.*moss|moss.*point|highhigh|moss.*landin<br>g|landmark|mosseler|cranberr|delineation|turf|street|braintree|agency|askin|pickerel|by-product|byproduct|by<br>product|chid|entrap|strap|sediment.*trap|contam.*trap|nutrient.*trap|pollut.*trap|trap.*contam|trap.*nutrient|trap.*pollut|sand.*trap|trap.*sand|trap.*sedim<br>ent|litter trap|fish trap|especial|power.*generation|electric.*generation|generation plant|generation facilit|laboratory observ|field observ|lab observ|special<br>issue|meadow beauty|river beauty|beautiful jacob|spring beauty|careful|exotic|extrapolat|askin|pickerel|invasive species|iskin|nuisance species|overview|in<br>view of|over-view|over- view|peltandra|pelti|pelto|apelt|peltu|pelta|pelte|provid.* no clue|provid.* notice|provid.* basis|provid.*expertise|rarely|temperature<br>value|biomass value|nonprofit|non-profit|refur|review|bayview|sensitive<br>fern|shunt|huntley|huntington|hunterdon|sking|salem|mysella|economus|newmarket|skink|skin lesion|skin condition|strap|sulfur|specialist|tradeoff|trade-<br>off|transport|workshop|grass shrimp
FEGS	Fungi	Fungi	include	fungus|fungi|mushroom
FEGS	Fungi	Fungi	near	accomodat|activit|aesthetic|ameniable|amenit|appreciat|artisan|artist|asset|attract|beaut|beneficial|benefit|bequest|biodiversity|bountiful|bounty|business|b<br>uy|care|caring|charisma|cherish|children|collect|comfort|commercial|commodities|commodity|goods|concern|conserv|corporation|critical|dealer|delicious|des<br>ired|desirable|destination|econom|edible|educat|encourag|endangered|enhance|enhancing|enjoy|encounter|enthusiast|experienc|exploit|extract|families|favo<br>rable|favor|feel|festiv|ceremon| fur | furs |fur-bear|fur<br>bear|furbear|future|gather|generation|hallmark|harvest|healthy|heritage|hide|hunt|income|indigenous.*people|indian population|indian<br>people|indigenous.*person|industry|initiative|inspir|interest|job|learn|legacy|leisure|livelihood|market|meat|native.*person|native<br>american|observ|opportunit|participant|participate|pelt|pick|pleasant|popular|preserv|products|profession|profit|promote|promotion|purpose|protect|quality<br>.*life|rare|recreat|relax|resource|restor|sale|save|saving|sell|sight|skins|special|specimen|spectacular|sport|steward|subsist|sustenance|threatened|together| tours|trade|trading|sustainabl|tradition|treasure|tribal|tribe|unique|valuable|value|valuing|view|visit|visitor|watch|well suited|well-<br>suited|wonder|allure|alluring|mystique|option value|lifeblood|jewel|extraordinary|excellent|amaze|amazing|stunning|majestic|spectacular
FEGS	Fungi	Fungi	exclude	fungicide|chemical|oak.*death|mushroomed|agency|askin|pickerel|by-product|byproduct|by<br>product|chid|entrap|strap|sediment.*trap|contam.*trap|pollut.*trap|trap.*contam|trap.*pollut|trap.*nutrient|nutrient.*trap|sand.*trap|trap.*sand|trap.*sedim<br>ent|litter trap|fish trap|especial|power.*generation|electric.*generation|generation plant|generation facilit|laboratory observ|field observ|lab observ|special<br>issue|meadow beauty|river beauty|beautiful jacob|spring beauty|beautyberry|carex|carey|personal care|careo|aquarium care|lawn<br>care|careful|exotic|extrapolat|askin|pickerel|invasive species|iskin|nuisance species|overview|in view of|over-view|over-<br>view|peltandra|pelti|pelto|apelt|peltu|pelta|pelte|pelta|pelte|provid.* no clue|provid.* notice|provid.* basis|provid.*expertise|rarely|temperature<br>value|biomass value|nonprofit|non-profit|refur|review|bayview|sensitive<br>fern|shunt|huntley|huntington|hunterdon|sking|salem|mysella|economus|newmarket|skink|skin lesion|skin condition|strap|sulfur|specialist|tradeoff|trade-<br>off|transport|workshop
FEGS	Land	Land	include	land|habitat

Category	Class	SubClass	Word_type	S2	S3
FEGS	Flora	Flora	exclude	County SEED|oyster|seeding|dispers|pumpkinseed	save|saving|savanna|agency|askin|pickerel|by-product|byproduct|by product|chid|entrap|strap|sediment.*trap|contam.*trap|nutrient.*trap|pollut.*trap|trap.*contam|trap.*nutrient|trap.*pollut|sand.*trap|trap.*sand|trap.*sediment|litter trap|fish trap|especial|power.*generation|electric.*generation|generation plant|generation facilit|laboratory observ|field observ|lab observ|special issue|meadow beauty|river beauty|beautiful jacob|spring beauty|beautyberry|carex|carey|personal care|careo|aquarium care|lawn care|careful|exotic|extrapolat|askin|pickerel|invasive species|iskin|nuisance species|overview|in view of|over-view|over-view|peltandra|pelti|pelto|apelt|peltu|pelta|pelte|provid.* no clue|provid.* notice|provid.* basis|provid.*expertise|rarely|temperature value|biomass value|nonprofit|non-profit|refur|review|bayview|sensitive fern|shunt|huntley|huntington|hunterdon|sking|salem|mysella|economus|new market|skink|skin lesion|skin condition|strap|sulfur|specialist|tradeoff|trade-off|transport|workshop
FEGS	Fungi	Fungi	include	fungus|fungi|mushroom	
FEGS	Fungi	Fungi	near	collect|gather|cook|sustenance|wild	
FEGS	Fungi	Fungi	exclude	fungicide|chemical|oak.*death|mushroomed	
FEGS	Land	Land	include	sprawl|public land|easement|land trust|disposal site|landtrust	coast|shore|estuar|marsh|bay|wetland|floodplain|acres|channel|creek|river|waterfront|forest|grassland

Category	Class	SubClass	Word_type	S4	S5
FEGS	Flora	Flora	exclude	raptor|fauna|animal|mammal|wildlife|bird|owl|fowl|duck|sparrow|warbler|plover|falcon|osprey|hawk|eagle|pelican|geese|pidgeon|pigeon|turtle|tortoise|seal|whale|dolphin|porpoise|manatee|otter|coral|dragonfl|insect|beetle|entomolo|bug|deer|turkey|quail|waterfowl|duck|goose|reptile|alligator|amphibian|salamander|frog|toad|skink|bat|nutria|raccoon|hogs|rabbit|beaver|rookery|rookeries|butter|horseshoe crab	pauciflora|processing plant|aguirre plant
FEGS	Fungi	Fungi	include		
FEGS	Fungi	Fungi	near		
FEGS	Fungi	Fungi	exclude		
FEGS	Land	Land	include	land-loss trend|land develop|develop land|developing land|developed land	unpaved parking|land preserv

<table>
<tr><th>Category</th><th>Class</th><th>SubClass</th><th>Word_type</th><th>S6</th><th>S7</th></tr>
<tr><td>FEGS</td><td>Flora</td><td>Flora</td><td>exclude</td><td></td><td>raptor|fauna|animal|mammal|wildlife|bird|owl|fowl|duck|sparrow|warbler|plc
ver|falcon|osprey|hawk|eagle|pelican|geese|pidgeon|pigeon|turtle|tortoise|sea
l|whale|dolphin|porpoise|manatee|otter|coral|dragonfl|insect|beetle|entomolo
|bug|deer|turkey|quail|waterfowl|duck|goose|reptile|alligator|amphibian|sala
mander|frog|toad|skink|bat|nutria|raccoon|hogs|rabbit|beaver|rookery|rookeri
es|butter|horseshoe
crab|plant.*upgrade|upgrade.*plant|planted|planting|plantation|grassroot|grass-
root|braintree|package plant|transplant|power plant|carene|treatment
plant|invasive plant|carex|carey|personal care|careo|aquarium care|lawn
care|careful|decreed|breed|hayden|cottonwood|freed|reedgrass|agreed|elderb
erry|cash|category|sewage|sanitary|acquatic|management.*area|fish|wash|dnr|
mcbp|trash|shoreline|pineda|pinellas|legend|pine.*island|pine.*barren|vash|el
mer|elm st|ashe|coal
ash|mash|sash|cash|bash|overwhelm|oakdale|oakley|croak|coal.*ash|delmarva
|fashion|pineland|barrier.*island|water.*pollution|soak|pine.*beach|over.*whel
med|barrier.*beach|ashore|sand.*pine|shorebird|percent|metals|oakland|silver
side|hash|develm|hairstreak|crash|pine.*hills|ocean.*pine|oak.*harbor|oak.*rid
ge|table|figure|characterized.*as|cdmo|unacceptable|seed|runoff|pollut|data|e
rosion|seem|timber|logging|logger|lumber|dash|Wash|eash|lash|mash|rash|ua
sh|especial|power.*generation|electric.*generation|generation plant|generation
facilit|laboratory observ|field observ|lab observ|special issue|meadow
beauty|river beauty|beautiful jacob|spring
beauty|careful|specialist|Department|mossler|thompson|sedge.*wren|sedge.*isl
and|sea.*sedge|subtidal|submergent.*moss|moss.*point|highhigh|moss.*landin
g|landmark|mosseler|cranberr|delineation|turf|street|braintree|agency|askin|pi
ckerel|by-product|byproduct|by
product|chid|entrap|strap|sediment.*trap|contam.*trap|nutrient.*trap|pollut.*t
rap|trap.*contam|trap.*nutrient|trap.*pollut|sand.*trap|trap.*sand|trap.*sedim
ent|litter trap|fish
trap|especial|power.*generation|electric.*generation|generation
plant|generation facilit|laboratory observ|field observ|lab observ|special
issue|meadow beauty|river beauty|beautiful jacob|spring
beauty|careful|exotic|extrapolat|askin|pickerel|invasive species|iskin|nuisance</td></tr>
<tr><td>FEGS</td><td>Fungi</td><td>Fungi</td><td>include</td><td></td><td></td></tr>
<tr><td>FEGS</td><td>Fungi</td><td>Fungi</td><td>near</td><td></td><td></td></tr>
<tr><td>FEGS</td><td>Fungi</td><td>Fungi</td><td>exclude</td><td></td><td></td></tr>
<tr><td>FEGS</td><td>Land</td><td>Land</td><td>include</td><td></td><td></td></tr>
</table>

Category	Class	SubClass	Word_type	S8	S9
FEGS	Flora	Flora	exclude		
FEGS	Fungi	Fungi	include		
FEGS	Fungi	Fungi	near		
FEGS	Fungi	Fungi	exclude		
FEGS	Land	Land	include		

Category	Class	SubClass	Word_type	S10	S11
FEGS	Flora	Flora	exclude		
FEGS	Fungi	Fungi	include		
FEGS	Fungi	Fungi	near		
FEGS	Fungi	Fungi	exclude		
FEGS	Land	Land	include		

Category	Class	SubClass	Word_type	S12	S13
FEGS	Flora	Flora	exclude		
FEGS	Fungi	Fungi	include		
FEGS	Fungi	Fungi	near		
FEGS	Fungi	Fungi	exclude		
FEGS	Land	Land	include		

Category	Class	SubClass	Word_type	S1
FEGS	Land	Land	near	smart growth|residential develop|commercial develop|sustainable develop|sustainable growth|future develop|new develop|proposed develop|compact develop|convert|filled|drained|ditched|filling|draining|acquisition|acquire|reclaim|reclamation|conversion|suitable for develop|cleared for|urban develop|develop undisturb|develop natural|developing undisturb|developing natural|developed undisturb|developed natural|impact develop|housing develop|pattern of develop|development pattern|proposed development|new construction|replace|replacing
FEGS	Land	Land	exclude	upland|landing|landmark|overland|landscap|highland|mainland|england|landing|landmark|cleveland|maryland|portland|cumberland|lander|oakland|stormwate r|rainwater|workshop|greenland|gland|system.*land|landbird|landing|landmark|poorly drained|well-drained|drainage basin|drain.*watershed|watershed.*drain|drain.*groundwater|groundwater.*drain|reservoir.*drain|drain.*reservoir
FEGS	Natural Materials	Natural Materials	include	natural.*material|material.*natural|material.*environment|material.*ecosystem|ecosystem.*material|environment.*material|aquatic.*material|material.*aquatic
FEGS	Natural Materials	Natural Materials	near	collect|amass|accumulate|gather|artistic|inspir|invent|imagin|ornament|jewelry|pottery

Category	Class	SubClass	Word_type	S2	S3
FEGS	Land	Land	near		smart growth|residential develop|commercial develop|sustainable develop|sustainable growth|future develop|new develop|proposed develop|compact develop|convert|filled|drained|ditched|filling|draining|acquisition|acquire|reclaim|reclamation|conversion|suitable for develop|cleared for|urban develop|develop undisturb|develop natural|developing undisturb|developing natural|developed undisturb|developed natural|impact develop|housing develop|pattern of develop|development pattern|proposed development|new construction
FEGS	Land	Land	exclude	frontier|dock|nantasket|issues|contaminat|highhigh|wbwp.*aww|permit.*requirement|application.*conference|15.*visitor	low tide|high tide|tidal|poorly drained|well-drained|drainage basin|drain.*watershed|watershed.*drain|drain.*groundwater|groundwater.*drain|reservoir.*drain|drain.*reservoir
FEGS	Natural Materials	Natural Materials	include	sand|shells|acorns|stick|clay|rock|fossil|stone|bones|feather	ornament|jewelry|pottery|brick
FEGS	Natural Materials	Natural Materials	near	aesthetic|artisan|artist|beneficial|benefit|bequest|bountiful|bounty|business|buy|cherish|children|collect|comfort|commercial|commodities|commodity|goods|concern|corporation|dealer|desired|desirable|destination|econom|educat|enhance|enhancing|encourag|enjoy|encounter|enthusiast|experienc|exploit|extract|families|favorable|favor|feel|festiv|ceremon|future|gather|generation|hallmark|harvest|heritage|income|indigenous.*people|indian population|indian people|indigenous.*person|industry|initiative|inspir|interest|job|learn|legacy|leisure|livelihood|market|native.*person|native american|observ|opportunit|participant|participate|pick|pleasant|popular|preserv|products|profession|profit|promote|promotion|purpose|resource|restor|sale|sell|special|sport|steward|subsist|trade|trading|sustainabl|tradition|treasure|tribal|tribe|valuable|value|valuing|well suited|well-suited|pottery|brick|ornament|jewelry	natur|organic|environment|ecosystem

Category	Class	SubClass	Word_type	S4	S5
FEGS	Land	Land	near		
FEGS	Land	Land	exclude		
FEGS	Natural Materials	Natural Materials	include	natural material|maple.*syrup|maple.*sugar|honey|shell mining|collecting coral|collect coral|coral.*jewelry|jewelry.*coral|souvenir.*coral|coral.*souvenir|natural object|mining|mine|quarry	fill|geotube|bag
FEGS	Natural Materials	Natural Materials	near		sand|gravel|rock|stone

Category	Class	SubClass	Word_type	S6	S7
FEGS	Land	Land	near		
FEGS	Land	Land	exclude		
FEGS	Natural Materials	Natural Materials	include	material.*place|place.*material|material.*dredg|dredg.*material	firewood|campfire
FEGS	Natural Materials	Natural Materials	near	benef|enhance|enhancing|create|creation|creating	

Category	Class	SubClass	Word_type	S8	S9
FEGS	Land	Land	near		
FEGS	Land	Land	exclude		
FEGS	Natural Materials	Natural Materials	include		
FEGS	Natural Materials	Natural Materials	near		

Category	Class	SubClass	Word_type	S10	S11
FEGS	Land	Land	near		
FEGS	Land	Land	exclude		
FEGS	Natural Materials	Natural Materials	include		
FEGS	Natural Materials	Natural Materials	near		

Category	Class	SubClass	Word_type	S12	S13
FEGS	Land	Land	near		
FEGS	Land	Land	exclude		
FEGS	Natural Materials	Natural Materials	include		
FEGS	Natural Materials	Natural Materials	near		

Category	Class	SubClass	Word_type	S1
FEGS	Natural Materials	Natural Materials	exclude	recycled material|informational material|educational material|instructional material|organic material|exhibit material|department.*of|environmental.*protection.*agency|conservation.*service|manag|monitor|data|research|degrad|loss|regulat|federal|law|enforce|officer|pollut|protect|project|agenc|govern|fund|stress|critical|restor|conserv|pesticide|invasive|nuisance|non.*native|nonnative|goal|runoff|protect|estuaries.*national.*significance|debris|strateg|damag|land.*use|analy|evaluat|brown.*tide|maintainance|assess|impact|investigate|policy|official|development|inform|zoning|trend|use.*conflict|research.*ecosystem.*assessment|guideline|objective|critical.*issue|resource.*conservation.*development|catalog|tours.*attendees|opportunit.*business|andrewsrock|research|assess|monitor|resource.*program|percent|action.*plan|invasive|nonnative|action.*item|debris|issue|figure15|policies|director.*natural|mitigation.*bank|seamless.*protect|land.*manag|plan|15.*visitor|natural.*flow|nutrient|dred|workshop|sediment|hab|imaging|sample|migrant|highhigh|report|inventory
FEGS	Open Space	Open Space	include	open.*space|space.*open|green.*space|space.*green|nature.*preserv|open area|wildland
FEGS	Open Space	Open Space	near	aesthetic|appreciat|heritage|together|enjoy|recreation|commercial|view|beaut|value|inspir|relax|leisure|econom|activit|feel|opportunit|tour|visit|bounty|bountiful|enhance|enhancing
FEGS	Open Space	Open Space	exclude	lose|loss|lost|restrict|federal|govern|manage|stress|preserv|conserv|law|fund|acqui|permit|plan|grant|text|bylaw|municipalit|analy|legislat|map|sprawl|program|conference|steward|goal|action|osrd|decreas|develop|population|division|emission|coliform|highigh|management.*issue|forsythe|jug.*ba|answers.*question|seagrass|program|plan|legislat|division|streetscape|street.*scape|highigh|management.*issue|forsythe|jug.*bay|answers.*question|seagrass|conserv|plan|legislat|program|manage|municipal|department|control|dissemination|office|agenc|maintain|parking.*lot|ocean.*park|street.*park|parks.*committee|parking.*facility|coliform|division.*park|highigh|management.*issue|forsythe|jug.*bay|kendall|interactive.*designer|highigh|management.*issue|forsythe|jug.*bay|answers.*question|seagrass|15.*visitor|parking|parker|parked|developed open space.*palustrine|palustrine.*developed open space|developed open space.*medium intensity developed|medium intensity developed.*developed open space
FEGS	Pollinators	Pollinators	include	pollinat|pollen
FEGS	Pollinators	Pollinators	near	
FEGS	Pollinators	Pollinators	exclude	ssc|lack.*educat|pollen history|cross-pollin

Category	Class	SubClass	Word_type	S2	S3
FEGS	Natural Materials	Natural Materials	exclude	clay brook|boneset|sand dollar|sand coco|stone lab|fossil fuel|sand barrier|sandflat|sand flat|sand accumulation|sticker|nonstick|capstone|keystone|cornerstone|touchstone|milestone|rocky mountain|gabion|rockcress|rockfish|larock|rock crab|shamrock|rock shrimp|rock cancer|rockport|rockaway|rocky|Rock Cove|rockingham|bedrock|rock fish|rock jasmine|rockport|rockaway|rocky|Rock Cove|sediment|channel|hammock|legend|dune|management.*description|report|inventory|list.*structure|burnunit|burn.*unit|facilit|forest.*manag|gis|document|data|spit|transport|site|adverse|bedrock|honey bee|clay creek|clayton|sandwich|runoff|sandwort|sandine|ersand|esand|dsand|sanddune|sandy|sand dune|sandspur|sander|ssand|sandwort|sandpiper|sand filter|esand|dsand|sand pine|sandusky|sand grass|sandplain|sandown|thousand|carex|carey|personal care|careo|aquarium care|lawn care|careful|wood.*smoke|sediment|channel|preservativ|pah|hab|hazard|highhigh|agency|askin|pickerel|by-product|byproduct|by product|chid|entrap|strap|sediment.*trap|sand.*trap|trap.*sand|trap.*sediment|litter trap|fish trap|especial|power.*generation|electric.*generation|generation plant|generation facilit|laboratory observ|field observ|lab observ|special issue|meadow beauty|river beauty|beautiful jacob|spring beauty|careful|exotic|extrapolat|askin|pickerel|invasive species|iskin|nuisance species|overview|in view of|over-view|over-view|peltandra|pelti|pelto|apelt|peltu|pelta|pelte|provid.* no clue|provid.* notice|provid.* basis|provid.*expertise|rarely|temperature value|biomass value|nonprofit|non-profit|refur|review|bayview|sensitive fern|shunt|huntley|huntington|hunterdon|sking|salem|mysella|economus|new market|skink|skin lesion|skin condition|strap|sulfur|specialist|tradeoff|trade-off|transport	figure15|policies|loss.*value|15.*visitor|highhigh|ornamental.*species
FEGS	Open Space	Open Space	include	open space|vast expanse|green space|greenspace	
FEGS	Open Space	Open Space	near		
FEGS	Open Space	Open Space	exclude	developed open space.*palustrine|palustrine.*developed open space|developed open space.*medium intensity developed|medium intensity developed.*developed open space	
FEGS	Pollinators	Pollinators	include	bee|butter|hummingbird|bat	
FEGS	Pollinators	Pollinators	near	flower|flora|crop|garden|farm|agricultur|native plant	
FEGS	Pollinators	Pollinators	exclude	chobee|been|beef|beetle|tombigbee|okeechobee|beeplant|ssc|lack.*educat|beet|bee county|bee education|document|ssc|lack.*educat|microp|batbing|bato|batk|tbat|batsto|bata|abat|bate|bath|bati|batu|batr|combat|butterfish|butterfly orchid|batt|buttercup|butter clam|butterwort|abutter|buttermilk|butterfly-pea|bat star|butterfly fish|butterweed|hummingbird flower|beec|beev|bee fly|beea	

<table>
<tr><th>Category</th><th>Class</th><th>SubClass</th><th>Word_type</th><th>S4</th><th>S5</th></tr>
<tr><td>FEGS</td><td>Natural Materials</td><td>Natural Materials</td><td>exclude</td><td>honey bee|suckle|locust|dew|mahoney|mesquite|timber|lumber|log|remediat.*min|data.*min|determining|examining|determine|examine|promine|mineral|fluminea|omine|minen|mineu|amine|plaquemine|ermin|minello|deter-min|deter-min|minea|post-min|division|smine|reclaim.*min|min.*reclaim|min.*reclam|reclam.*min|under-min|lettuce</td><td>sand flat|sand barrier|sandflat|sand accumulation|popcorn bag|capstone|keystone|cornerstone|touchstone|milestone|rockport|rocky mountain|gabion|rockcress|rockfish|larock|rock crab|shamrock|rock shrimp|rock cancer|rockport|rockaway|rocky|Rock Cove|rockingham|bedrock|rock fish|rock jasmine|rockport|rockaway|rocky|Rock Cove|sediment|channel|hammock|legend|dune|management.*description|report|inventory|list.*structure|burnunit|burn.*unit|facilit|forest.*manag|gis|document|data|spit|transport|site|adverse|bedrock|honey bee|clay creek|clayton|sandwich|runoff|sandwort|sandine|ersand|esand|dsand|sandy|sanddune|sand dune|sandspur|sander|ssand|sandwort|sandpiper|sand filter|esand|dsand|sand pine|sandusky|sand grass|sandplain|sandown|thousand|carex|carey|personal care|careo|careful|aquarium care|lawn care|wood.*smoke|sediment|channel|preservativ|pah|hab|hazard|highhigh</td></tr>
<tr><td>FEGS</td><td>Open Space</td><td>Open Space</td><td>include</td><td></td><td></td></tr>
<tr><td>FEGS</td><td>Open Space</td><td>Open Space</td><td>near</td><td></td><td></td></tr>
<tr><td>FEGS</td><td>Open Space</td><td>Open Space</td><td>exclude</td><td></td><td></td></tr>
<tr><td>FEGS</td><td>Pollinators</td><td>Pollinators</td><td>include</td><td></td><td></td></tr>
<tr><td>FEGS</td><td>Pollinators</td><td>Pollinators</td><td>near</td><td></td><td></td></tr>
<tr><td>FEGS</td><td>Pollinators</td><td>Pollinators</td><td>exclude</td><td></td><td></td></tr>
</table>

Category	Class	SubClass	Word_type	S6	S7
FEGS	Natural Materials	Natural Materials	exclude	recycled material|informational material|educational material|instructional material|organic material|exhibit material|recreat	
FEGS	Open Space	Open Space	include		
FEGS	Open Space	Open Space	near		
FEGS	Open Space	Open Space	exclude		
FEGS	Pollinators	Pollinators	include		
FEGS	Pollinators	Pollinators	near		
FEGS	Pollinators	Pollinators	exclude		

Category	Class	SubClass	Word_type	S8	S9
FEGS	Natural Materials	Natural Materials	exclude		
FEGS	Open Space	Open Space	include		
FEGS	Open Space	Open Space	near		
FEGS	Open Space	Open Space	exclude		
FEGS	Pollinators	Pollinators	include		
FEGS	Pollinators	Pollinators	near		
FEGS	Pollinators	Pollinators	exclude		

Category	Class	SubClass	Word_type	S10	S11
FEGS	Natural Materials	Natural Materials	exclude		
FEGS	Open Space	Open Space	include		
FEGS	Open Space	Open Space	near		
FEGS	Open Space	Open Space	exclude		
FEGS	Pollinators	Pollinators	include		
FEGS	Pollinators	Pollinators	near		
FEGS	Pollinators	Pollinators	exclude		

Category	Class	SubClass	Word_type	S12	S13
FEGS	Natural Materials	Natural Materials	exclude		
FEGS	Open Space	Open Space	include		
FEGS	Open Space	Open Space	near		
FEGS	Open Space	Open Space	exclude		
FEGS	Pollinators	Pollinators	include		
FEGS	Pollinators	Pollinators	near		
FEGS	Pollinators	Pollinators	exclude		

<table>
<tr><th>Category</th><th>Class</th><th>SubClass</th><th>Word_type</th><th>S1</th></tr>
<tr><td>FEGS</td><td>Presence of the Envi</td><td>Presence of the Envi</td><td>include</td><td>essay|contest| art|accomodat|activit|ameniable|appreciat|attract|beneficial|benefit|bequest|biodiversity|bountiful|bounty|business|buy|care|caring|charisma|cherish|children|commercial|concern|conserv|corporation|critical|desired|desirable|destination|econom|educat|encourag|enhance|enhancing|enjoy|encounter|enthusiast|experienc|families|favorable|favor|feel|festiv|ceremon|future|generation|hallmark|healthy|heritage|income|indigenous.*people|indian population|indian people|indigenous.*person|industry|initiative|inspir|interest|job|learn|legacy|leisure|livelihood|native.*person|native american|opportunit|participant|participate|pleasant|popular|preserv|pride|profession|profit|promote|promotion|purpose|protect|quality.*life|recreat|relax|restor|save|saving|special|sport|steward|together|tours|sustainabl|tradition|treasure|tribal|tribe|unique|valuable|value|valuing|visit|visitor|well suited|well-suited|wonder|allure|alluring|mystique|option value|lifeblood|jewel|extraordinary|excellent|amaze|amazing|stunning|majestic|spectacular|way of life|sense of place|tour guide</td></tr>
<tr><td>FEGS</td><td>Presence of the Envi</td><td>Presence of the Envi</td><td>near</td><td>ecosystem|environment|natur|estuar|bay|ecological|outdoor|wilderness|watershed|coast|shore|wetland|parks|region|land|out-of doors</td></tr>
<tr><td>FEGS</td><td>Presence of the Envi</td><td>Presence of the Envi</td><td>exclude</td><td>landfill|product|goods|environmentally|aerial photograph|digitiz|orthophotograph|bay scallop|agency|askin|pickerel|by-product|byproduct|by product|chid|entrap|strap|sediment.*trap|contam.*trap|pollut.*trap|trap.*contam|trap.*pollut|trap.*nutrient|nutrient.*trap|sand.*trap|trap.*sand|trap.*sediment|litter trap|fish trap|especial|power.*generation|electric.*generation|generation plant|generation facilit|laboratory observ|field observ|lab observ|special issue|meadow beauty|river beauty|beautiful jacob|spring beauty|beautyberry|carex|carey|personal care|careo|aquarium care|lawn care|careful|exotic|extrapolat|askin|pickerel|invasive species|iskin|nuisance species|overview|in view of|over-view|over- view|peltandra|pelti|pelto|apelt|peltu|pelta|pelte|provid.* no clue|provid.* notice|provid.* basis|provid.*expertise|rarely|temperature value|biomass value|nonprofit|non-profit|refur|review|bayview|sensitive fern|shunt|huntley|huntington|hunterdon|sking|salem|mysella|economus|newmarket|skink|skin lesion|skin condition|strap|sulfur|specialist|tradeoff|trade-off|transport|workshop|regional|interested part|toxic of concern|toxics of concern|contaminant of concern|contaminants of concern</td></tr>
<tr><td>FEGS</td><td>Soil</td><td>Soil</td><td>include</td><td>soil|dirt</td></tr>
<tr><td>FEGS</td><td>Soil</td><td>Soil</td><td>near</td><td>quality|decompos|replenish|resource|productivity|microbe|microorg|fung|rich|health|nutrient|fertile|drainage|farm|agricultur|suitable|suitability|moisture|capacity|arable|condition|good|prime|production|productive|well-drained|formation|organic</td></tr>
</table>

Category	Class	SubClass	Word_type	S2	S3
FEGS	Presence of the Envi	Presence of the Envi	include	hike|hiking|rock climb|bike|biking|bicycl|camping|camper|camps|campground|outing|sightsee|beach.*activit|sight-see|beach.*go|picnic|snowmobil|skiing|sledding|ski area|ski resort|snow sport|trail|rec.*hunt|sport.*hunt|hunt.*rec|hunt.*sport|off-road vehicle|nature tour|outdoor program|environmental program|nature photograph|outdoor photog|local photog|outdoor writ|nature writ|environmental writ|waterfront|marina|field trip|interpretive tour|interpretive program|interpretive material|boardwalk|ecotouris|eco-touris|field activit	research|scientific|science|scientist
FEGS	Presence of the Envi	Presence of the Envi	near		improve.*learn|enhance.*learn|learn.*improve|learn.*enhance|improve.*knowledge|enhance.*knowledge|knowledge.*improve|knowledge.*enhance|permit|opportunit|encourag|accomodat|attract|interest|initiative|promote|reference site|specimen.*collect|sample.*collect|data.*collect|collect.*specimen|collect.*sample|collect.*data|specimen.*gather|sample.*gather|data.*gather|gather.*specimen|gather.*sample|gather.*data|collect.*research|research.*collect|scien.*collect|collect.*scien
FEGS	Presence of the Envi	Presence of the Envi	exclude	campaign|suppl|campbell|salmon.*camp|highigh|management.*issue|forsythe|jug.*bay|answers.*question|15.*visitor|steelhead|trailer|traillii|hitchhike|ladder|passage barrier|scenic|view|camp.*dresser|camp.*mckee|zostera	
FEGS	Soil	Soil	include	soil|dirt	soil|dirt
FEGS	Soil	Soil	near	promot.*research|research.*promot|promot.*science|science.*promot|science.*potential|potential.*science|research.*potential|potential.*research|science.*purpose|purpose.*science|research.*purpose|purpose.*research|improve.*knowledge|enhance.*knowledge|knowledge.*improve|knowledge.*enhance|improve.*learn|enhance.*learn|learn.*improve|learn.*enhance	accomodat|ameniable|amenit|appreciat|asset|attract|beneficial|benefit|bequest|bountiful|bounty|business|buy|collect|comfort|commercial|commodities|commodity|goods|concern|conserv|corporation|critical|desired|desirable|destination|econom|educat|encourag|enhance|enhancing|enjoy|encounter|enthusiast|experienc|exploit|extract|families|favorable|favor|feel|festiv|ceremon|future|gather|generation|hallmark|harvest|healthy|heritage|income|indigenous.*people|indian population|indian people|indigenous.*person|industry|initiative|inspir|interest|job|learn|legacy|leisure|livelihood|native.*person|native american|opportunit|participen|participate|pleasant|popular|preserv|products|profession|profit|promote|promotion|purpose|protect|quality.*life|rare|resource|restor|sale|save|saving|sell|special|steward|subsist|sustenance|together|sustainabl|tradition|treasure|tribal|tribe|unique|valuable|value|valuing|well suited|well-suited|wonder|option value|lifeblood|extraordinary

<table>
<tr><th>Category</th><th>Class</th><th>SubClass</th><th>Word_type</th><th>S4</th><th>S5</th></tr>
<tr><td>FEGS</td><td>Presence of the Envi</td><td>Presence of the Envi</td><td>include</td><td>flood|storm|shoreline|coastal|dune|wave|erosion|hazard</td><td>nitrogen|carbon|air|atmosphere|water|sediment|erosion</td></tr>
<tr><td>FEGS</td><td>Presence of the Envi</td><td>Presence of the Envi</td><td>near</td><td>buffer|filter|control|protect|retain|retention|attenuat|mitigat</td><td>fixation|sink|replenish|recycle|sequestration|remove|removal|service|improve|f
ilter|purify|buffer|clean|clear|natural.*process|process.*natural|sequester|natur
al.*treat|treat.*natural</td></tr>
<tr><td>FEGS</td><td>Presence of the Envi</td><td>Presence of the Envi</td><td>exclude</td><td>related.*actions|action.*plan|15.*visitor|applicant|non.*native|flood.*hazard.*co
ntrol|dredg|mussel|oyster|mollusk|restore|county.*control|control.*district|filte
r.*strip|bmp|sewage|pollut|discharge|impervious|potential.*prob|potential.*iss
ue|cfda|diking</td><td>ciceet|pollut|total.*nitrogen.*loading|tmdl|hydrocarbon|ciceet|pollut|goal|plan|
implement|problem|impact|challenge|action|manage|assess|department|water.
*quality|agency|inhibit|monitor|load|treatment|data|mandat|develop|act|test|
activit|criteria|measur|inform|filter|hydrocarbon|tmdl|regulat|radiocarbon|toxic
|algal|algae|hypoxia|eutrophic|septic|pollution|contam|metal|excess|discharge
|gas|brown.*tide|investigat|pcb|nonpoint|non.*point|concentration|storm.*wat
er|stormwater|carbonate|slough|parameter|sample|15.*visitor|pair|fair|air
yam|air
plant|chair|questionnaire|airborne|affair|millionaire|dairy|act|airshed|National.
*Weather.*Service|impair|repair|topic|federal|belleair|tmdl|air.*act|ciceet|prair
|airport|clean.*air.*act|national.*air.*standard|airplane|airstrip|muck|regulat.*ai
r|lair|Air Resource Specialist|Clean Air Bond|potato|dair|air force|www|Air
Resources Lab|air rifle|air condition|airfield|rural atmosphere|cooperative
atmosphere|welcoming|clean water act</td></tr>
<tr><td>FEGS</td><td>Soil</td><td>Soil</td><td>include</td><td></td><td></td></tr>
<tr><td>FEGS</td><td>Soil</td><td>Soil</td><td>near</td><td></td><td></td></tr>
</table>

Category	Class	SubClass	Word_type	S6	S7
FEGS	Presence of the Envi	Presence of the Envi	include	absorb.*pollut|filter.*pollut|clean.*air|purify.*air|remove.*pollut|reduce.*pollut|reduce.*smog|remove.*smog|corridor|shade|shading|stabiliz|filter.*sediment|groundwater|improve.*water|clarif|filter.*water|clean.*air|purify.*air|buffer|filter|control|protect|retain|retention|fixation|sink|replenish|recycle|sequestration|sequester|filter|purify|buffer|clean|natural.*process|process.*natural|buffer.*erosion|natural.*treat|treat.*natural	bioswale|bio-swale|rain.*garden|bioretention|wind.*break|break.*wind|denitrif|absorb.*pollut|filter.*pollut|purify.*air|remove.*smog|filter.*sediment|filter.*water|purify.*air|natur.*process|process.*natur|natur.*filter|filter.*natur|natur.*filtrat|filtrat.*natur|infiltrate|infiltration|drainfield|sequester carbon|carbon sequest|buffer.*erosion|purify.*water|urban buffer|carbon capture
FEGS	Presence of the Envi	Presence of the Envi	near	tree|forest|vegetat|pine|oak|cypress| elm|ash|maple|hickory|hardwood|riparian|canopy|tree|forest|wetland|natural|vegetat|plants|flora|marsh|green|estuar|land|stream|river	
FEGS	Presence of the Envi	Presence of the Envi	exclude	transplant|nightshade|marshfield|marshall|braintree|in the process|dash|Wash|eash|lash|mash|rash|uash|cash|delineation|turf|pesticide|fungicide|fertilizer|street|clean water act|clean air act	restor|drain|train|brain|grain
FEGS	Soil	Soil	include		
FEGS	Soil	Soil	near		

Category	Class	SubClass	Word_type	S8	S9
FEGS	Presence of the Envi	Presence of the Envi	include	buffer	
FEGS	Presence of the Envi	Presence of the Envi	near	visual|sound|erosion|zone	
FEGS	Presence of the Envi	Presence of the Envi	exclude		
FEGS	Soil	Soil	include		
FEGS	Soil	Soil	near		

Category	Class	SubClass	Word_type	S10	S11
FEGS	Presence of the Envi	Presence of the Envi	include		
FEGS	Presence of the Envi	Presence of the Envi	near		
FEGS	Presence of the Envi	Presence of the Envi	exclude		
FEGS	Soil	Soil	include		
FEGS	Soil	Soil	near		

Category	Class	SubClass	Word_type	S12	S13
FEGS	Presence of the Envi	Presence of the Envi	include		
FEGS	Presence of the Envi	Presence of the Envi	near		
FEGS	Presence of the Envi	Presence of the Envi	exclude		
FEGS	Soil	Soil	include		
FEGS	Soil	Soil	near		

Category	Class	SubClass	Word_type	S1
FEGS	Soil	Soil	exclude	infertile|unproductive|poor|disturbed|anaerobic|drainfield|drainage basin|drainage<br>ditch|substrate|filter|retention|buffer|infiltration|runoff|sedimentation|nonpoint|categor|implement|eelgrass|oyster.*grass|seagrass|problem|impact|particle|c<br>hallenge|action|manage|goal|sediment.*trap|contam.*trap|pollut.*trap|trap.*contam|trap.*pollut|sand.*trap|trap.*sand|assess|department|water.*quality|age<br>ncy|inhibit|monitor|load|treatment|conserv|data|mandat|develop|act|test|activit|compound|criteria|measur|inform|filter|tmdl|metal|regulat|hydrocarbon|ra<br>diocarbon|construct|toxic|discharge|transport|contamin|impair|guide|stud|pollution|contamin|regulat|nonpoint|non.*point|research|septic|bulkhead|seafood<br>|exceed|deplete|water.*clarity|standard|index|target|program|marine.*sediment.*trap|contam.*trap|pollut.*trap|trap.*contam|trap.*pollut|sand.*trap|trap.*s<br>and|deposit|bottom sedime|resuspen
FEGS	Sounds and Scents	Sounds and Scents	include	sound
FEGS	Sounds and Scents	Sounds and Scents	near	listen|hear|sensation|sensory|auditory|noise|audible
FEGS	Sounds and Scents	Sounds and Scents	exclude	buffer|listening<br>session|long|manage|management|program|alternative|technology|protocol|habitat|study|measure|debris|involv|crescent|puget.*sound.*action|sound.*practi<br>ce|disruptive|15.*visitor|glisten|noxious|unpleasant|excessive|unneccesary|hearing|heart|pollut|listening|shear|hear appeal|disruptive|disturb
FEGS	Substrate	Substrate	include	substrate|creat.*surface|provid.*surface
FEGS	Substrate	Substrate	near	construct|benefit|nourish|replenish|stabiliz|econom|produce|create|value|valuable|valuing|restor|base|support|suitable|deposit

Category	Class	SubClass	Word_type	S2	S3
FEGS	Soil	Soil	exclude	drainfield|substrate|filter|retention|buffer|infiltration|runoff|sedimentation|nonpoint|categor|implement|eelgrass|oyster.*grass|seagrass|problem|impact|particle|challenge|action|manage|goal|sediment.*trap|contam.*trap|pollut.*trap|trap.*contam|trap.*pollut|sand.*trap|trap.*sand|assess|department|water.*quality|agency|inhibit|monitor|load|treatment|conserv|data|mandat|develop|act|test|activit|compound|criteria|measur|inform|filter|tmdl|metal|regulat|hydrocarbon|radiocarbon|construct|toxic|discharge|transport|contamin|impair|guide|stud|pollution|contamin|regulat|nonpoint|non.*point|research|septic|bulkhead|seafood|exceed|deplete|water.*clarity|standard|index|target|program|marine.*sediment.*trap|contam.*trap|pollut.*trap|trap.*contam|trap.*pollut|sand.*trap|trap.*sand|deposit|bottom sedime|resuspen	infertile|unproductive|poor|disturbed|anaerobic|drainfield|substrate|filter|retention|buffer|infiltration|runoff|sedimentation|nonpoint|categor|implement|eelgrass|oyster.*grass|seagrass|problem|impact|particle|challenge|action|manage|goal|sediment.*trap|contam.*trap|pollut.*trap|trap.*contam|trap.*pollut|sand.*trap|trap.*sand|assess|department|water.*quality|agency|inhibit|monitor|load|treatment|conserv|data|mandat|develop|act|test|activit|compound|criteria|measur|inform|filter|tmdl|metal|regulat|hydrocarbon|radiocarbon|construct|toxic|discharge|transport|contamin|impair|guide|stud|pollution|contamin|regulat|nonpoint|non.*point|research|septic|bulkhead|seafood|exceed|deplete|water.*clarity|standard|index|target|program|marine.*sediment.*trap|contam.*trap|pollut.*trap|trap.*contam|trap.*pollut|sand.*trap|trap.*sand|deposit|bottom sedime|resuspen|agency|askin|pickerel|by-product|byproduct|by product|chid|entrap|strap|sediment.*trap|contam.*trap|pollut.*trap|trap.*contam|trap.*pollut|sand.*trap|trap.*sand|trap.*sediment|litter trap|fish trap|especial|power.*generation|electric.*generation|generation plant|generation facilit|laboratory observ|field observ|lab observ|special issue|meadow beauty|river beauty|beautiful jacob|spring beauty|beautyberry|carex|carey|personal care|careo|aquarium care|lawn care|careful|exotic|extrapolat|askin|pickerel|invasive species|iskin|nuisance species|overview|in view of|over-view|over-view|peltandra|pelti|pelto|apelt|peltu|pelta|pelte|provid.* no clue|provid.* notice|provid.* basis|provid.*expertise|rarely|temperature value|biomass value|nonprofit|non-profit|refur|review|bayview|sensitive fern|shunt|huntley|huntington|hunterdon|sking|salem|mysella|economus|new market|skink|skin lesion|skin condition|strap|sulfur|specialist|tradeoff|trade-off|transport|workshop
FEGS	Sounds and Scents	Sounds and Scents	include	scent|sensory|olfactory|auditory|smell|listen|noise|aroma|audible|odor|fragrant|fragrance	listen|noise
FEGS	Sounds and Scents	Sounds and Scents	near	environment|ecosystem|natur|flora|flower|plant|berry|berries|estuary|beach|bird.*sing|sing.*bird|song.*bird|bird.*song|croak|chirp|rustl|splash|thunder|song|waves|ocean|natur|cricket|insect|wildlife|environ|bird|wood|forest|grass|comfort|enjoy|pleasant	bird.*sing|sing.*bird|song.*bird|bird.*song|croak|chirp|rustle|rustling|splash|thunder|song|waves|ocean|cricket|insect|wildlife|environ|bird|wood|forest|grass|cicada|katydid|cricket|birds|singing|howl|frog
FEGS	Sounds and Scents	Sounds and Scents	exclude	transplant|polycylic|grassroot|grass-root|aromaticum|aromatic hydrocarbon|buffer|listening session|fluorescent|quiescent|crescent|audubon|15.*visitor|noxious|unpleasant|using|increasing|rising|losing|accessing|dressing|crossing|disposing|promising|passing|proposing|isinglass|licensing|assessing|raising|single|foreclosing|purchasing|collapsing|processing|data|monitor|measur|crucial|glisten|excessive|unneccesary|hearing|heart|pollut|shear|hear appeal|disruptive|disturb|escent|pigeonwing|odora|delairea|odore|odoru|odori|odorv|ethofum|theodor|crab-scented brittle	grassroot|grass-root|buffer|listening session|fluorescent|quiescent|crescent|audubon|15.*visitor|noxious|unpleasant|using|increasing|rising|losing|accessing|dressing|crossing|disposing|promising|passing|proposing|isinglass|licensing|assessing|raising|single|foreclosing|purchasing|collapsing|processing|data|monitor|measur|crucial|glisten|excessive|unneccesary|hearing|heart|pollut|shear|hear appeal|disruptive|disturb|escent
FEGS	Substrate	Substrate	include	sand|beach	oyster.*reef
FEGS	Substrate	Substrate	near	nourish|replenish|restor	construct|benefit|nourish|replenish|stabiliz|econom|commercial|produce|create|value|valuable|valuing|restor|provid

Category	Class	SubClass	Word_type	S4	S5
FEGS	Soil	Soil	exclude		
FEGS	Sounds and Scents	Sounds and Scents	include	rural atmosphere|serene|serenity|owl call|bird call	hear
FEGS	Sounds and Scents	Sounds and Scents	near		bird.*sing|sing.*bird|song.*bird|bird.*song|croak|chirp|rustle|rustling|splash|thunder|song|waves|ocean|cricket|insect|cicada|katydid|cricket|birds|wind|singing|howl|frog|noise|sound|audible
FEGS	Sounds and Scents	Sounds and Scents	exclude		the sound|Puget sound|croaker|buffer|listening session|fluorescent|quiescent|crescent|audubon|15.*visitor|noxious|unpleasant|using|increasing|rising|losing|accessing|dressing|crossing|disposing|promising|passing|proposing|isinglass|licensing|assessing|raising|single|foreclosing|purchasing|collapsing|processing|data|monitor|measur|crucial|glisten|excessive|unnecesary|hearing|heart|pollut|shear|hear appeal|disruptive|disturb|escent
FEGS	Substrate	Substrate	include	substrate|creat.*surface|provid.*surface	substrate|creat.*surface|provid.*surface|stabiliz|base
FEGS	Substrate	Substrate	near	farm|agricultur|aquacultur|hatchery|garden	clay|chalk|bedrock|limestone|granite|marble|shells|reef

Category	Class	SubClass	Word_type	S6	S7
FEGS	Soil	Soil	exclude		
FEGS	Sounds and Scents	Sounds and Scents	include	bird.*sing|sing.*bird|song.*bird|bird.*song|croak|chirp|rustle|rustling|splash|song|cricket|cicada|katydid|singing|noise|howl	odor|smell
FEGS	Sounds and Scents	Sounds and Scents	near	enjoy|pleasant|watch|observ|audible	ameliorate|mitigat|concern
FEGS	Sounds and Scents	Sounds and Scents	exclude	croaker|rsing|nsing|ssing|asing|esing|osing|ising|using|ysing|buffer|listening session|fluorescent|quiescent|crescent|audubon|15.*visitor|noxious|unpleasant|using|increasing|rising|losing|accessing|dressing|crossing|disposing|promising|passing|proposing|isinglass|licensing|assessing|raising|single|foreclosing|purchasing|collapsing|processing|data|monitor|measur|crucial|glisten|excessive|unnecesary|hearing|heart|pollut|shear|hear appeal|disruptive|disturb|escent	odora|delairea|odore|odoru|odori|odorv|ethofum|theodor
FEGS	Substrate	Substrate	include	muck.*estuar|estuar.*muck|muck.*wetland|wetland.*muck	
FEGS	Substrate	Substrate	near	important|vital|importance|essential|critical	

Category	Class	SubClass	Word_type	S8	S9
FEGS	Soil	Soil	exclude		
FEGS	Sounds and Scents	Sounds and Scents	include		
FEGS	Sounds and Scents	Sounds and Scents	near		
FEGS	Sounds and Scents	Sounds and Scents	exclude		
FEGS	Substrate	Substrate	include		
FEGS	Substrate	Substrate	near		

Category	Class	SubClass	Word_type	S10	S11
FEGS	Soil	Soil	exclude		
FEGS	Sounds and Scents	Sounds and Scents	include		
FEGS	Sounds and Scents	Sounds and Scents	near		
FEGS	Sounds and Scents	Sounds and Scents	exclude		
FEGS	Substrate	Substrate	include		
FEGS	Substrate	Substrate	near		

Category	Class	SubClass	Word_type	S12	S13
FEGS	Soil	Soil	exclude		
FEGS	Sounds and Scents	Sounds and Scents	include		
FEGS	Sounds and Scents	Sounds and Scents	near		
FEGS	Sounds and Scents	Sounds and Scents	exclude		
FEGS	Substrate	Substrate	include		
FEGS	Substrate	Substrate	near		

<table>
<tr><th>Category</th><th>Class</th><th>SubClass</th><th>Word_type</th><th>S1</th></tr>
<tr><td>FEGS</td><td>Substrate</td><td>Substrate</td><td>exclude</td><td>artificial substrate|substrateg|surface water|surface flow|surface-
water|substrateg|change|priority|municipalit|www|contaminat|pollut|toxic|toxin|salamander|claytonia|permit|report|goal|study|dredg|monitor|metal|identif|
project|inform|creek|construction|sewer|waste|marblemount|durante.*park|dnrec|boring|barrier.*island|manag.*plan|identify|15.*visitor|groundwater|water
suppl|nursery|spawn|background|groundwork|impervious|water quality|groundcover|ground cover|lightning|disposal|middle
ground|middleground|mining|staging</td></tr>
<tr><td>FEGS</td><td>Timber</td><td>Timber</td><td>include</td><td>timber|logging|lumber</td></tr>
<tr><td>FEGS</td><td>Timber</td><td>Timber</td><td>near</td><td></td></tr>
<tr><td>FEGS</td><td>Timber</td><td>Timber</td><td>exclude</td><td>timber
oat|clogging|woody|invasive|nonnative|non.*native|manage|plan|goal|legislat|fish|threat.*watershed|watershed.*threat|threat.*habitat|habitat.*threat|harmf
ul|marine|http|sewage|sanitary|acquatic|plastic|management.*area|runoff|stormwater|dredg|greek|ocean|sea|coast|septic|university|mcbp|landed|landing|la
ndmark|council|forestry.*service|forestablish|shoreline|geologic|forest.*stewardship|lakewood|aquatic.*structure|survey|perservativ|woodneck|woods.*hole|us
da|forest.*service|kirkwood|toxic|toxin|barrier.*island|water.*pollution|beachwood|treated.*timber|treated.*wood|hollywood|wood.*neck|barrier.*beach|shor
ebird|percent|metals|brentwood|silverside|loblolly|contamin|department.*of.*forest|psu|project.*park.*water|forest.*comittee|baywood|aquatic.*inventory|w
ood.*stove|legend|habitat.*conversion.*loss.*forest.*cover|kelp.*forest|table|figure|characterized.*as|forest.*learning.*shelter|vincent|cdmo|unnaceptable|15.*
visitor|wooden.*marco|litter.*retard|treated lumber|rattle|snake|data|timber creek|timberneck|timber support|joist|joe timberland</td></tr>
<tr><td>FEGS</td><td>Viewscapes</td><td>Viewscapes</td><td>include</td><td>view</td></tr>
</table>

Category	Class	SubClass	Word_type	S2	S3
FEGS	Substrate	Substrate	exclude	thousand|sandwich|runoff|sandy|www|conflict|shifting|practice|manag|proposal|permit|contaminat|pollut|toxic|toxin|sandpiper|sandplain|state|sandi|sandy|permit|report|cranberry|goal|sander|sanding|study|deposit|project|inform|land fill|association|council|convert|rsite|document|sewage|uranium|plutonium|americium|sand.*pond|hard.*shoreline|figure|generat|radio|excavat|sand.*dune|sandbass|sand.*lance|percent|costsand|durante.*park|dnrec|sand.*eel|boring|barrier.*island|sand.*fencing|sandy.*hook|sand.*pit|sand.*movement|sandhill|manag.*plan|identify|sand.*hill|barrier.*beach|15.*visitor|water suppl|disposal|mining|sandwich|runoff|sandwort|sandine|ersand|esand|dsand|sandy|sanddune|sand dune|sandspur|sander|ssand|sandwort|sandpiper|sand filter|esand|dsand|sand pine|sandusky|sand grass|sandplain|sandown|thousand	locations.*study.*area|restoration.*progress|emerson.*point.*preserv|durante.*park|dnrec|identify|oyster.*restoration.*field|barrier.*island|15.*visitor
FEGS	Timber	Timber	include	wood harvest|wood product|forest product	pine|wood|pulp|tree|timber|oak| elm|lumber|cedar|pine|oak|cypress| elm|ash|maple|hickory|hardwood|cordwood
FEGS	Timber	Timber	near		extract|commercial|business|product|industry|commodity|commodities|harvest|profit|buy|livelihood|job|dealer|market|corporation|sale|sell|income|artisan|profession|trade|trading|subsist|tribal|tribe|indigenous.*people|indian population|indian people|indigenous.*cultur|indigenous.*people|indian population|indian people|native.*person|native american|traditional use|traditional sustainable use|sustenance|goods
FEGS	Timber	Timber	exclude		street|trademark|braintree|park|woody|lakewood|kirkwood|woodpecker|woodshole|wood dock|wood preserv|woodneck|woodbury|wood stake|wooden|wood tick|woodland|wood turtle|tree frog|woodbridge|treated lumber|treated wood|wood stove|reproduct|horticultur|agricultur|alternativ|aqua.*cultur|monocultur|non-nativ|cash|category|sewage|sanitary|acquatic|management.*area|fish|mash|wash|dnr|mcbp|trash|shoreline|pineda|pinellas|legend|pine.*island|pine.*barren|vash|elmer|elm st|ashe|coal ash|mash|sash|cash|bash|overwhelm|oakdale|oakley|croak|coal.*ash|delmarva|fashion|pineland|barrier.*island|water.*pollution|soak|pine.*beach|over.*whelmed|barrier.*beach|ashore|sand.*pine|shorebird|percent|metals|oakland|silverside|hash|develm|hairstreak|crash|pine.*hills|ocean.*pine|oak.*harbor|oak.*ridge|pine.*seed|sea grass|freed|mcreedy|sea-grass|eelgrass|oyster.*grass|seagrass|grassland|sea-grass|decreed|breed|agreed|haynes|hay.*barn|hayden|cottonwood|freed|cottontail|reedgrass|hayan|salem|mysella|economus|woodfern|sandalwoods|wood satyr|wood borer|wood nymph
FEGS	Viewscapes	Viewscapes	include	view|aesthetically pleasing	scenic|scenery|horizon|skyline|vantage point|vistas|observation point|observation deck|observation platform|observation area|vista point|scenic vista|natural vista|beauty|beautiful|panorama|panoramic|viewscape|view scape|idyllic

Category	Class	SubClass	Word_type	S4	S5
FEGS	Substrate	Substrate	exclude	artificial substrate|substrateg|surface water|surface flow|surface-water|soil|sediment|background|groundwork|groundwater|impervious|water quality|groundcover|ground cover|lightning|disposal|middle ground|middleground|mining|staging	artificial reef|artificial substrate|substrateg|surface water|surface flow|surface-water|database|data base|soil|sediment|background|groundwork|groundwater|impervious|water quality|groundcover|ground cover|lightning|disposal|middle ground|middleground|mining|staging
FEGS	Timber	Timber	include		
FEGS	Timber	Timber	near		
FEGS	Timber	Timber	exclude		
FEGS	Viewscapes	Viewscapes	include	sight	beautification

Category	Class	SubClass	Word_type	S6	S7
FEGS	Substrate	Substrate	exclude		
FEGS	Timber	Timber	include		
FEGS	Timber	Timber	near		
FEGS	Timber	Timber	exclude		
FEGS	Viewscapes	Viewscapes	include	rolling hill|rolling farmland|rolling landscape	lush|majestic|aesthetic|visually pleasing

Category	Class	SubClass	Word_type	S8	S9
FEGS	Substrate	Substrate	exclude		
FEGS	Timber	Timber	include		
FEGS	Timber	Timber	near		
FEGS	Timber	Timber	exclude		
FEGS	Viewscapes	Viewscapes	include		

Category	Class	SubClass	Word_type	S10	S11
FEGS	Substrate	Substrate	exclude		
FEGS	Timber	Timber	include		
FEGS	Timber	Timber	near		
FEGS	Timber	Timber	exclude		
FEGS	Viewscapes	Viewscapes	include		

Category	Class	SubClass	Word_type	S12	S13
FEGS	Substrate	Substrate	exclude		
FEGS	Timber	Timber	include		
FEGS	Timber	Timber	near		
FEGS	Timber	Timber	exclude		
FEGS	Viewscapes	Viewscapes	include		

<table>
<tr><th>Category</th><th>Class</th><th>SubClass</th><th>Word_type</th><th>S1</th></tr>
<tr><td>FEGS</td><td>Viewscapes</td><td>Viewscapes</td><td>near</td><td>accomodat|activit|aesthetic|ameniable|amenit|appreciat|artisan|artist|asset|attract|beaut|beneficial|benefit|bequest|bountiful|bounty|business|buy|care|cari
ng|charisma|cherish|children|collect|comfort|commercial|commodities|commodity|goods|concern|conserv|corporation|critical|dealer|delicious|desired|desirab
le|destination|econom|edible|educat|encourag|endangered|enhance|enhancing|enjoy|encounter|enthusiast|experienc|exploit|extract|families|favorable|favor
|feel|festiv|ceremon| fur | furs |fur-bear|fur
bear|furbear|future|gather|generation|hallmark|harvest|healthy|heritage|hide|hunt|income|indigenous.*people|indian population|indian
people|indigenous.*person|industry|initiative|inspir|interest|job|learn|legacy|leisure|livelihood|market|meat|native.*person|native
american|observ|opportunit|participant|participate|pelt|pick|pleasant|popular|preserv|products|profession|profit|promote|promotion|purpose|protect|quality
.*life|rare|recreat|relax|resource|restor|sale|save|saving|sell|sight|skins|special|specimen|spectacular|sport|steward|subsist|sustenance|threatened|together|
tours|trade|trading|sustainabl|tradition|treasure|tribal|tribe|unique|valuable|value|valuing|view|visit|visitor|watch|well suited|well-
suited|wonder|allure|alluring|mystique|option value|lifeblood|jewel|extraordinary|excellent|amaze|amazing|stunning|majestic|spectacular</td></tr>
<tr><td>FEGS</td><td>Viewscapes</td><td>Viewscapes</td><td>exclude</td><td>riverview|viewcontent|views as issues|target view|view.*data|points of view|elevation view|known view|view this document|seek the view|solicit the view|reflect
the
view|review|bayview|interview|data.*network|aesthetic.*nuisance|aesthetic.*problem|aesthetic.*degradation|vista.*hermosa|vistamar|views.*agency|views.*de
partment|sewage.*collection|aerial.*view.*modif|15.*visitor|treatment|keep.*louisiana|regulation|program.*beautification|beautification.*program|inspir.*probl
em|inspir.*strateg|program.*inspire.*action|conference.*inspir|tool.*inspir|siberian|chamisso|sandwort|pondweed|goal|success.*inspir|legislat|meadowbeauty|
educat.*inspir|mission|initiative|agency.*funding|slough|program|slough|15.*visitor|plankton.*reduce|ad.*hoc|inspir.*steward|inspir.*teach|beautyberry|Harpe
r.*beauty|meadow-beauty|overview|in view of|over-view|over- view|viewed|our view|species viewer|wildlife viewing|point of view|exhibit|landscape
design|landscape ecology|built landscape|landscaper|purview|viewpoint|view point|negative view|their views|landscape scale|landscape
perspective|salem|mysella|economus</td></tr>
<tr><td>FEGS</td><td>Water</td><td>Water</td><td>include</td><td>rain.*garden|rain.*barrel|store.*rain|rain.*store|storage.*rain|rain.*storage|cistern|water resource|water source|water use|water usage|water
conservation|conserve water|water shortage|water storage|water demand|rainwater harvest|water supply|water-supply|water demand|demand for
water|demands for water|water well|supply well|municipal well|domestic well|private well|public well|artesian well|shallow well|groundwater well|water
withdraw|receiving water bod|reclaimed water|reclaim water|water reclamation|water pump|clean water|clean air and water|wastewater.*compliance</td></tr>
<tr><td>FEGS</td><td>Water</td><td>Water</td><td>near</td><td></td></tr>
<tr><td>FEGS</td><td>Water</td><td>Water</td><td>exclude</td><td>swells|wells reserve|wells bay|town of wells|clean water act|sundew</td></tr>
<tr><td>FEGS</td><td>Weather</td><td>Weather</td><td>include</td><td>weather|climate|precipitation|temperature|rain|snow|sun|cloud|summer|fall|winter|spring|autumn|heat|humid</td></tr>
<tr><td>FEGS</td><td>Weather</td><td>Weather</td><td>near</td><td>comfort|desirable|destination|enjoy|favorable|pleasant|popular|relax|special|spectacular|tours|treasure|well suited|well-
suited|wonder|allure|alluring|mystique|extraordinary|excellent|amaze|amazing|stunning|majestic|spectacular|condition|moderate|mild</td></tr>
</table>

Category	Class	SubClass	Word_type	S2	S3
FEGS	Viewscapes	Viewscapes	near	natur|environment|ecosystem|estuar|coastal|landscape|ocean|shore|beach|water|forest|woodland|grass|green|farmland|hills|mountain|wetland|creek|river|stream|lake|pond	
FEGS	Viewscapes	Viewscapes	exclude	viewcontent|views as issues|target view|view.*data|points of view|elevation view|known view|view this document|seek the view|solicit the view|reflect the view|review|bayview|interview|data.*network|aesthetic.*nuisance|aesthetic.*problem|aesthetic.*degradation|vista.*hermosa|vistamar|views.*agency|views.*department|sewage.*collection|aerial.*view.*modif|15.*visitor|treatment|keep.*louisiana|regulation|program.*beautification|beautification.*program|inspir.*problem|inspir.*strateg|program.*inspire.*action|conference.*inspir|tool.*inspir|siberian|chamisso|sandwort|pondweed|goal|success.*inspir|legislat|meadowbeauty|educat.*inspir|mission|initiative|agency.*funding|slough|program|slough|15.*visitor|plankton.*reduce|ad.*hoc|inspir.*steward|inspir.*teach|beautyberry|Harper.*beauty|meadow-beauty|overview|in view of|over-view|over- view|viewed|our view|species viewer|wildlife viewing|point of view|view the|views the|chula vista|viewing|exhibit|landscape design|landscape ecology|built landscape|landscaper|purview|viewpoint|view point|negative view|their views|landscape scale|landscape perspective	meadow beauty|river beauty|beautiful jacob|spring beauty|meadow
FEGS	Water	Water	include	water|stream|river|creek|estuar|bay|reservoir|lake	aquifer|hydro elect|hydroelect|hydro-elect|hydropower|hydro-power|dam.*electric|electric.*dam|cooling system|energy.*dam|dam.*energy
FEGS	Water	Water	near	subsist|drink|intake	
FEGS	Water	Water	exclude	stormwater|sample|evaluat|collectively|ordinance|disposal|demonstrat|data|monitor|watershed|waterfowl|watershed|wastewater|permit|division.*water|water supply paper|sundew	
FEGS	Weather	Weather	include	sunbath|sun bath|sun-bath|seasonal visitor	fog
FEGS	Weather	Weather	near		form|generate|create|add|scenic|special|aesthetic|view|visual|scene|scenic|inspir|relax|gaze|gazing|enjoy|special|unique|charisma|enjoy|preserv|view|beaut|bounty|bountiful|observ|benefit|recreat|valuable|watch|tours|aesthetic|value|rare|relax|attract|scenic|sight|restore|resource|protect|asset|special|participant|amenit|desirable|pleasant|healthy|hallmark|recreat|opportunit|experienc|enjoy|relax|families|family|children|visitor|touris

Category	Class	SubClass	Word_type	S4	S5
FEGS	Viewscapes	Viewscapes	near	comfort|desirable|destination|enjoy|favorable|pleasant|popular|relax|special|sp<br>ectacular|tours|treasure|well suited|well-<br>suited|wonder|allure|alluring|mystique|extraordinary|excellent|amaze|amazing| stunning|majestic|spectacular|scenic|pleasing	
FEGS	Viewscapes	Viewscapes	exclude	[illegible]beauty|meadow-beauty|horizontal|deepwater horizon|may be overlooked|overlook.*concern|beautyberry|Harper.*beauty	
FEGS	Water	Water	include	water|stream|river|creek|estuar|bay|marina|boat|ship|the<br>sound|wetland|marine|reservoir|lake	water|stream|river|creek|estuar|bay|recreation|discharg|enjoy|moor|charter|t<br>ours|cruise|port|harbor|access|traffic|channel|canal|launch|reservoir|lake
FEGS	Water	Water	near	discharge|emission|point source|dilute|dilution|TMDL|daily<br>load|ballast|divert|dispos.*material|dispos.*waste|waste.*dispos|material.*disp<br>os|dredg.*dispos|dispos.*dredg|dispos.*debris|debris.*dispos|place.*dredge|dre<br>dge.*place|placing.*dredg|dredg.*placing|pollution<br>from|contamin.*load|pollut.*load|sediment.*load|dump.*dredg|dredg.*dump|ef<br>fluent	boat|ship|vessel|ferry|paddle
FEGS	Water	Water	exclude	stormwater|spoil<br>deposition|sediment.*deposition|deposition.*sediment|stormwater|sample|eval<br>uat|collectively|ordinance|demonstrat|data|monitor|watershed|waterfowl|divisi<br>on.*water|water supply paper|pumpout|pump<br>out|stewardship|partnership|waterfowl|ownership|relationship|leadership|spon<br>sorship|township|membership|shipped|rship|nship|zostera|sundew	stewardship|partnership|waterfowl|ownership|relationship|leadership|sponsors<br>hip|township|membership|shipped|rship|nship|support|harbor seal|new<br>bedford harbor|bight|sundew|boat-tailed grackle
FEGS	Weather	Weather	include		
FEGS	Weather	Weather	near		

Category	Class	SubClass	Word_type	S6	S7
FEGS	Viewscapes	Viewscapes	near		natur|environment|ecosystem|estuar|coastal|landscape|ocean|shore|beach|water|forest|woodland|grass|green|farmland|hills|mountain|wetland|creek|river|stream|lake|pond
FEGS	Viewscapes	Viewscapes	exclude		flush|plush|ilush|slush|visualize
FEGS	Water	Water	include	water.*receiv|ocean.*receiv|bay.*receiv|receiv.*water|receiv.*ocean|receiv.*bay|stream.*receiv|receiv.*stream|river.*receiv|receiv.*river|lake.*receiv|receiv.*lake|pond.*receiv|receiv.*pond	irrigat|watercraft|sailboat|tugboat|jetski|kayak|canoe|boating|boater|shipping channel|stream channel
FEGS	Water	Water	near	dischar|pollut|waste|contaminant|effluent	
FEGS	Water	Water	exclude	sundew	volcano
FEGS	Weather	Weather	include		
FEGS	Weather	Weather	near		

Category	Class	SubClass	Word_type	S8	S9
FEGS	Viewscapes	Viewscapes	near		
FEGS	Viewscapes	Viewscapes	exclude		
FEGS	Water	Water	include	water	water|stream|river|creek|estuar|channel|canal|wetland|reservoir|lake
FEGS	Water	Water	near	promot.*research|research.*promot|promot.*science|science.*promot|science.*potential|potential.*science|research.*potential|potential.*research|science.*purpose|purpose.*science|research.*purpose|purpose.*research|improve.*knowledge|enhance.*knowledge|knowledge.*improve|knowledge.*enhance|improve.*learn|enhance.*learn|learn.*improve|learn.*enhance	navigat|navigab|transport
FEGS	Water	Water	exclude	stormwater|watershed|wastewater|sundew	sundew
FEGS	Weather	Weather	include		
FEGS	Weather	Weather	near		

Category	Class	SubClass	Word_type	S10	S11
FEGS	Viewscapes	Viewscapes	near		
FEGS	Viewscapes	Viewscapes	exclude		
FEGS	Water	Water	include	snorkel|SCUBA|swim|swam|wade|diving|dive|diver|wading|bather|bathing	stream|river|creek|estuar|channel|canal|wetland|reservoir|lake
FEGS	Water	Water	near		livestock.*water|water.*livestock|cattle.*water|cattle.*livestock
FEGS	Water	Water	exclude	diversi|diverse|divert|biodiversity|diving.*duck|wastewater|steelhead|diversion|flock|spoonbill|egret|waterfowl|wading bird|swamp|kwader|diverg|swimming crab	
FEGS	Weather	Weather	include		
FEGS	Weather	Weather	near		

Category	Class	SubClass	Word_type	S12	S13
FEGS	Viewscapes	Viewscapes	near		
FEGS	Viewscapes	Viewscapes	exclude		
FEGS	Water	Water	include	water	wastewater|water.*discharge|discharge.*water
FEGS	Water	Water	near	protect|conserv|preserv|effluent	compliance|compliant
FEGS	Water	Water	exclude	sundew|watershed|waterfowl	
FEGS	Weather	Weather	include		
FEGS	Weather	Weather	near		

<table>
<tr><th>Category</th><th>Class</th><th>SubClass</th><th>Word_type</th><th>S1</th></tr>
<tr><td>FEGS</td><td>Weather</td><td>Weather</td><td>exclude</td><td>theat|train|winter salt marsh|rainwater kill|sundew|suny|fall river|freshwater spring|falls within|staff|summer
camp|worker|sunken|program|session|employ|wave climate|wintering|fallout|summer flounder|winter flounder|outfall|attractant|fall below|fall within|fall
under|offseason|off-season|wintergreen|spring beauty|climate change|changing climate|climate
adapt|analysis|study|temperature.*water|water.*temperature|stream.*temperature|temperature.*stream|temperature.*impair|impair.*temperature|climate.*ac
t|temperature.*manag|manag.*temperature|weather.*station|collect.*rain|map|lumber.*landing|landmark|growing.*season.*moss|water.*collect|15.*visitor|tra
in|drain|brain|grain|plover|Commission|season.*fire|fire.*season|touris.*season|season.*touris|especially|specialty|monitor|data|egret|season.*fish|fish.*seaso
n|management|rain garden|raingarden|closure|hunt.*season|season.*hun|seasonal wetland|all.*weather|terrain|rainbow|rainier|workshop|suisun|block
sun|Sunday|unit|sunfish|sunrise|sunset|tsunami|sundown|clouded|cloud.*water|turbid|sunset of|sunset on|biofog|to
sunset|image|imaging|clouded|navigat|sunset of|sunset on|sunset in|sunsets on|sunsets in|infog|turbid|cloud.*water|rainbow snake|cloudberry|after
sunset|rainbow star|rainbow surf|sunset at|before sunset|mccloud|agency|askin|pickerel|by-product|byproduct|by
product|chid|entrap|strap|sediment.*trap|contam.*trap|pollut.*trap|trap.*contam|trap.*pollut|nutrient.*trap|trap.*nutrient|sand.*trap|trap.*sand|trap.*sedim
ent|litter trap|fish trap|especial|power.*generation|electric.*generation|generation plant|generation facilit|laboratory observ|field observ|lab observ|special
issue|meadow beauty|river beauty|beautiful jacob|spring beauty|beautyberry|carex|carey|personal care|careo|aquarium care|lawn
care|careful|exotic|extrapolat|askin|pickerel|invasive species|iskin|nuisance species|overview|in view of|over-view|over-
view|peltandra|pelti|pelto|apelt|peltu|pelta|pelte|provid.* no clue|provid.* notice|provid.* basis|provid.*expertise|rarely|temperature value|biomass
value|nonprofit|non-profit|refur|review|bayview|sensitive fern|shunt|huntley|huntington|hunterdon|sking|salem|mysella|economus|newmarket|skink|skin
lesion|skin condition|strap|sulfur|specialist|tradeoff|trade-off|transport|workshop</td></tr>
<tr><td>FEGS</td><td>Wind</td><td>Wind</td><td>include</td><td>wind</td></tr>
<tr><td>FEGS</td><td>Wind</td><td>Wind</td><td>near</td><td>boat|surfer|surfing|energy|electric|power|sport|turbine</td></tr>
<tr><td>FEGS</td><td>Wind</td><td>Wind</td><td>exclude</td><td>windscreen|dwind|window|real.*time.*system|sediment|data|tax.*credit|bottle|defense|energy.*transfer|mooring|wind.*erosion|pollut|gis|wind.*biolog|abso
b.*wind|overflow|xxxxxxx|15.*visitor|stratification|stratify|circulate|mixing|circulation</td></tr>
</table>

Category	Class	SubClass	Word_type	S2	S3
FEGS	Weather	Weather	exclude		biofog|pollution|pollutant|climate|temperature|navigation|15.*visitor
FEGS	Wind	Wind	include	fly.*kite|kite.*fly|kite surf	
FEGS	Wind	Wind	near		
FEGS	Wind	Wind	exclude	butterfly|white-tailed kite	

Category	Class	SubClass	Word_type	S4	S5
FEGS	Weather	Weather	exclude		
FEGS	Wind	Wind	include		
FEGS	Wind	Wind	near		
FEGS	Wind	Wind	exclude		

Category	Class	SubClass	Word_type	S6	S7
FEGS	Weather	Weather	exclude		
FEGS	Wind	Wind	include		
FEGS	Wind	Wind	near		
FEGS	Wind	Wind	exclude		

Category	Class	SubClass	Word_type	S8	S9
FEGS	Weather	Weather	exclude		
FEGS	Wind	Wind	include		
FEGS	Wind	Wind	near		
FEGS	Wind	Wind	exclude		

Category	Class	SubClass	Word_type	S10	S11
FEGS	Weather	Weather	exclude		
FEGS	Wind	Wind	include		
FEGS	Wind	Wind	near		
FEGS	Wind	Wind	exclude		

Category	Class	SubClass	Word_type	S12	S13
FEGS	Weather	Weather	exclude		
FEGS	Wind	Wind	include		
FEGS	Wind	Wind	near		
FEGS	Wind	Wind	exclude		
