# Supplementary material for: Who Benefits from National Estuaries? Applying the FEGS Classification System to Identify Ecosystem Services and their Beneficiaries
Source: Int J Environ Res Public Health. 2019 Jul 3;16(13):2351. doi: 10.3390/ijerph16132351 (PMC6651245; doi:10.3390/ijerph16132351)
Supplement: Supplementary file 1 [file ijerph-16-02351-s001.zip › S2_FEGS_Combinations_Counts.xlsx.pdf]

AppendixB_FEGS_Combinations_Counts.xlsx

This excel file contains counts of the number of management plans flagged as matching key words to the given subclasses in each row.

**Beneficiary_SubClass**	Subclass of Beneficiaries from the FEGS Classification System
**Ecosystem_SubClass**	Subclass of Ecosystems from the FEGS Classification System
**FEGS_SubClass**	Subclass of FEGS types from the FEGS Classification System
**NEP_Count**	Number of NEP management plans (out of 28) with atleast one sentence flagged as containing synonym keywords associated with that combination of subclasses
**NERR_Count**	Number of NERR management plans (out of 29) with atleast one sentence flagged as containing synonym keywords associated with that combination of subclasses

Beneficiary_SubClass	Ecosystem_SubClass	FEGS_SubClass	NEP_Count	NERR_Count
All Humans	Aquatic	Air	2	0
All Humans	Atmosphere	Air	3	0
Commercial_Military Transportation	Atmosphere	Air	2	2
Commercial_Military Transportation	Terrestrial	Air	1	0
Commerical_Industrial	Aquatic	Air	1	0
Commerical_Industrial	Atmosphere	Air	4	1
Educators and Students	Atmosphere	Air	1	0
Electric and other Energy Generators	Atmosphere	Air	2	0
Electric and other Energy Generators	Terrestrial	Air	1	0
Experiencers and Viewers	Atmosphere	Air	3	2
Government and Municipal and Residential	Aquatic	Air	1	0
Government and Municipal and Residential	Atmosphere	Air	9	7
Government and Municipal and Residential	Terrestrial	Air	0	1
Industrial Dischargers	Aquatic	Air	1	1
Industrial Dischargers	Atmosphere	Air	1	2
Industrial Processors	Aquatic	Air	0	1
Industrial Processors	Atmosphere	Air	3	1
People Who Care (Existence)	Atmosphere	Air	7	8
People Who Care (Existence)	Estuaries and Near Coastal Marine	Air	3	0
People Who Care (Option_Bequest)	Aquatic	Air	2	0
People Who Care (Option_Bequest)	Atmosphere	Air	2	0
Recreational	Atmosphere	Air	0	2
Researchers	Atmosphere	Air	1	0
Resource-Dependent Businesses	Aquatic	Air	1	0
Resource-Dependent Businesses	Atmosphere	Air	2	0
Experiencers and Viewers	Atmosphere	Atmospheric Phenomena	0	1
Experiencers and Viewers	Barren_Rock and Sand	Atmospheric Phenomena	0	2
Experiencers and Viewers	Estuaries and Near Coastal Marine	Atmospheric Phenomena	2	4
Experiencers and Viewers	Lakes and Ponds	Atmospheric Phenomena	0	1
Experiencers and Viewers	Rivers and Streams	Atmospheric Phenomena	0	2
Agricultural	Agroecosystems	Depredators and (Pest) Predators	1	0
Agricultural	Aquatic	Depredators and (Pest) Predators	1	0
Agricultural	Estuaries and Near Coastal Marine	Depredators and (Pest) Predators	1	0
Agricultural Processors	Agroecosystems	Depredators and (Pest) Predators	1	0
All Humans	Wetlands	Depredators and (Pest) Predators	1	0
Commercial_Military Transportation	Aquatic	Depredators and (Pest) Predators	1	0
Educators and Students	Agroecosystems	Depredators and (Pest) Predators	1	0
Educators and Students	Aquatic	Depredators and (Pest) Predators	2	0
Educators and Students	Terrestrial	Depredators and (Pest) Predators	1	0
Farmers	Agroecosystems	Depredators and (Pest) Predators	2	0
Farmers	Estuaries and Near Coastal Marine	Depredators and (Pest) Predators	1	0
Government and Municipal and Residential	Agroecosystems	Depredators and (Pest) Predators	2	0
Government and Municipal and Residential	Aquatic	Depredators and (Pest) Predators	2	0
Government and Municipal and Residential	Created Greenspace	Depredators and (Pest) Predators	3	0
Government and Municipal and Residential	Estuaries and Near Coastal Marine	Depredators and (Pest) Predators	1	0
Government and Municipal and Residential	Forests	Depredators and (Pest) Predators	1	1
Government and Municipal and Residential	Terrestrial	Depredators and (Pest) Predators	2	0
Industrial Processors	Created Greenspace	Depredators and (Pest) Predators	1	0
Learning	Agroecosystems	Depredators and (Pest) Predators	1	0
Learning	Created Greenspace	Depredators and (Pest) Predators	2	0
People Who Care (Existence)	Agroecosystems	Depredators and (Pest) Predators	1	0
People Who Care (Existence)	Aquatic	Depredators and (Pest) Predators	1	0
People Who Care (Existence)	Estuaries and Near Coastal Marine	Depredators and (Pest) Predators	1	0
Recreational	Created Greenspace	Depredators and (Pest) Predators	3	0
Researchers	Terrestrial	Depredators and (Pest) Predators	1	0
Residential Property Owners	Agroecosystems	Depredators and (Pest) Predators	1	0
Residential Property Owners	Created Greenspace	Depredators and (Pest) Predators	3	0
Residential Property Owners	Terrestrial	Depredators and (Pest) Predators	2	0
Resource-Dependent Businesses	Created Greenspace	Depredators and (Pest) Predators	3	0
Resource-Dependent Businesses	Terrestrial	Depredators and (Pest) Predators	3	0
Agricultural	Agroecosystems	Fauna	8	8
All Humans	Aquatic	Fauna	3	1
All Humans	Created Greenspace	Fauna	1	0
All Humans	Rivers and Streams	Fauna	1	0
All Humans	Terrestrial	Fauna	1	2
Aquaculturists	Aquatic	Fauna	2	3
Aquaculturists	Estuaries and Near Coastal Marine	Fauna	1	1
Aquaculturists	Lakes and Ponds	Fauna	1	0
Aquaculturists	Open Oceans and Seas	Fauna	3	2
Aquaculturists	Rivers and Streams	Fauna	2	1
Artists	Created Greenspace	Fauna	0	1
Artists	Estuaries and Near Coastal Marine	Fauna	1	0
Artists	Rivers and Streams	Fauna	1	0
Artists	Terrestrial	Fauna	0	1
Boaters	Aquatic	Fauna	12	20
Boaters	Estuaries and Near Coastal Marine	Fauna	2	5
Boaters	Open Oceans and Seas	Fauna	1	0

Boaters	Rivers and Streams	Fauna	0	1
Boaters	Terrestrial	Fauna	0	1
Boaters	Wetlands	Fauna	0	2
Commercial_Military Transportation	Aquatic	Fauna	0	1
Commercial_Military Transportation	Rivers and Streams	Fauna	1	0
Commercial_Military Transportation	Terrestrial	Fauna	0	2
Commerical_Industrial	Agroecosystems	Fauna	2	0
Commerical_Industrial	Aquatic	Fauna	2	1
Commerical_Industrial	Estuaries and Near Coastal Marine	Fauna	3	1
Commerical_Industrial	Forests	Fauna	0	1
Commerical_Industrial	Open Oceans and Seas	Fauna	1	1
Commerical_Industrial	Terrestrial	Fauna	2	1
Commerical_Industrial	Wetlands	Fauna	0	1
Educators and Students	Aquatic	Fauna	5	8
Educators and Students	Barren_Rock and Sand	Fauna	0	1
Educators and Students	Created Greenspace	Fauna	0	1
Educators and Students	Estuaries and Near Coastal Marine	Fauna	5	15
Educators and Students	Forests	Fauna	0	1
Educators and Students	Lakes and Ponds	Fauna	2	0
Educators and Students	Open Oceans and Seas	Fauna	1	4
Educators and Students	Rivers and Streams	Fauna	0	1
Educators and Students	Scrublands_Shrublands	Fauna	1	0
Educators and Students	Terrestrial	Fauna	1	3
Educators and Students	Wetlands	Fauna	0	4
Experiencers and Viewers	Aquatic	Fauna	13	25
Experiencers and Viewers	Barren_Rock and Sand	Fauna	2	11
Experiencers and Viewers	Created Greenspace	Fauna	4	21
Experiencers and Viewers	Estuaries and Near Coastal Marine	Fauna	12	19
Experiencers and Viewers	Forests	Fauna	1	7
Experiencers and Viewers	Grasslands	Fauna	1	2
Experiencers and Viewers	Open Oceans and Seas	Fauna	3	4
Experiencers and Viewers	Rivers and Streams	Fauna	5	4
Experiencers and Viewers	Scrublands_Shrublands	Fauna	1	1
Experiencers and Viewers	Terrestrial	Fauna	6	16
Experiencers and Viewers	Wetlands	Fauna	5	10
Food Extractors	Agroecosystems	Fauna	0	1
Food Extractors	Aquatic	Fauna	16	22
Food Extractors	Estuaries and Near Coastal Marine	Fauna	16	14
Food Extractors	Open Oceans and Seas	Fauna	0	1
Food Extractors	Rivers and Streams	Fauna	3	3
Food Extractors	Terrestrial	Fauna	2	4
Food Extractors	Wetlands	Fauna	4	3
Food Pickers and Gatherers	Aquatic	Fauna	2	1
Food Pickers and Gatherers	Estuaries and Near Coastal Marine	Fauna	1	1
Food Subsisters	Aquatic	Fauna	1	1
Food Subsisters	Estuaries and Near Coastal Marine	Fauna	0	2
Food Subsisters	Forests	Fauna	2	2
Food Subsisters	Open Oceans and Seas	Fauna	0	1
Food Subsisters	Rivers and Streams	Fauna	1	0
Food Subsisters	Terrestrial	Fauna	0	2
Foresters	Forests	Fauna	3	2
Fur_Hide Trappers and Hunters	Agroecosystems	Fauna	2	1
Fur_Hide Trappers and Hunters	Aquatic	Fauna	6	12
Fur_Hide Trappers and Hunters	Estuaries and Near Coastal Marine	Fauna	4	3
Fur_Hide Trappers and Hunters	Forests	Fauna	1	0
Fur_Hide Trappers and Hunters	Rivers and Streams	Fauna	0	3
Fur_Hide Trappers and Hunters	Terrestrial	Fauna	3	5
Fur_Hide Trappers and Hunters	Wetlands	Fauna	1	3
Government and Municipal and Residential	Aquatic	Fauna	17	24
Government and Municipal and Residential	Barren_Rock and Sand	Fauna	1	2
Government and Municipal and Residential	Created Greenspace	Fauna	2	8
Government and Municipal and Residential	Estuaries and Near Coastal Marine	Fauna	20	24
Government and Municipal and Residential	Forests	Fauna	0	2
Government and Municipal and Residential	Lakes and Ponds	Fauna	2	3
Government and Municipal and Residential	Open Oceans and Seas	Fauna	4	7
Government and Municipal and Residential	Rivers and Streams	Fauna	6	5
Government and Municipal and Residential	Scrublands_Shrublands	Fauna	2	0
Government and Municipal and Residential	Terrestrial	Fauna	15	26
Government and Municipal and Residential	Wetlands	Fauna	4	5
Hunters	Agroecosystems	Fauna	2	1
Hunters	Aquatic	Fauna	6	23
Hunters	Barren_Rock and Sand	Fauna	2	3
Hunters	Created Greenspace	Fauna	0	11
Hunters	Estuaries and Near Coastal Marine	Fauna	14	20
Hunters	Forests	Fauna	4	9
Hunters	Grasslands	Fauna	0	2
Hunters	Lakes and Ponds	Fauna	2	4
Hunters	Open Oceans and Seas	Fauna	1	2

Hunters	Rivers and Streams	Fauna	1	7
Hunters	Terrestrial	Fauna	11	26
Hunters	Wetlands	Fauna	4	14
Inspirational	Created Greenspace	Fauna	0	1
Inspirational	Estuaries and Near Coastal Marine	Fauna	0	1
Inspirational	Forests	Fauna	0	1
Inspirational	Open Oceans and Seas	Fauna	1	2
Inspirational	Terrestrial	Fauna	0	3
Learning	Aquatic	Fauna	3	6
Learning	Barren_Rock and Sand	Fauna	0	1
Learning	Created Greenspace	Fauna	0	3
Learning	Estuaries and Near Coastal Marine	Fauna	5	7
Learning	Forests	Fauna	0	2
Learning	Open Oceans and Seas	Fauna	0	2
Learning	Terrestrial	Fauna	6	8
Learning	Wetlands	Fauna	0	1
Military_Coast Guard	Terrestrial	Fauna	0	1
Military_Coast Guard	Wetlands	Fauna	1	0
People Who Care (Existence)	Agroecosystems	Fauna	1	3
People Who Care (Existence)	Aquatic	Fauna	25	29
People Who Care (Existence)	Barren_Rock and Sand	Fauna	4	9
People Who Care (Existence)	Created Greenspace	Fauna	1	4
People Who Care (Existence)	Estuaries and Near Coastal Marine	Fauna	26	29
People Who Care (Existence)	Forests	Fauna	8	14
People Who Care (Existence)	Grasslands	Fauna	0	1
People Who Care (Existence)	Lakes and Ponds	Fauna	4	5
People Who Care (Existence)	Open Oceans and Seas	Fauna	15	21
People Who Care (Existence)	Rivers and Streams	Fauna	19	11
People Who Care (Existence)	Scrublands_Shrublands	Fauna	1	5
People Who Care (Existence)	Terrestrial	Fauna	26	28
People Who Care (Existence)	Wetlands	Fauna	14	17
People Who Care (Option_Bequest)	Aquatic	Fauna	3	4
People Who Care (Option_Bequest)	Created Greenspace	Fauna	0	1
People Who Care (Option_Bequest)	Estuaries and Near Coastal Marine	Fauna	3	10
People Who Care (Option_Bequest)	Forests	Fauna	0	1
People Who Care (Option_Bequest)	Lakes and Ponds	Fauna	1	1
People Who Care (Option_Bequest)	Open Oceans and Seas	Fauna	1	3
People Who Care (Option_Bequest)	Rivers and Streams	Fauna	3	0
People Who Care (Option_Bequest)	Terrestrial	Fauna	5	13
People Who Care (Option_Bequest)	Wetlands	Fauna	2	1
Pharmaceutical and Food Supplement Suppliers	Aquatic	Fauna	2	1
Pharmaceutical and Food Supplement Suppliers	Estuaries and Near Coastal Marine	Fauna	2	1
Recreational	Aquatic	Fauna	20	25
Recreational	Barren_Rock and Sand	Fauna	0	1
Recreational	Created Greenspace	Fauna	3	10
Recreational	Estuaries and Near Coastal Marine	Fauna	14	17
Recreational	Forests	Fauna	0	1
Recreational	Open Oceans and Seas	Fauna	1	2
Recreational	Rivers and Streams	Fauna	1	1
Recreational	Terrestrial	Fauna	4	13
Recreational	Wetlands	Fauna	7	6
Researchers	Aquatic	Fauna	1	1
Researchers	Estuaries and Near Coastal Marine	Fauna	2	17
Researchers	Lakes and Ponds	Fauna	0	1
Researchers	Open Oceans and Seas	Fauna	0	3
Researchers	Rivers and Streams	Fauna	5	3
Researchers	Scrublands_Shrublands	Fauna	0	1
Researchers	Terrestrial	Fauna	0	4
Researchers	Wetlands	Fauna	0	1
Residential Property Owners	Agroecosystems	Fauna	1	1
Residential Property Owners	Aquatic	Fauna	2	0
Residential Property Owners	Estuaries and Near Coastal Marine	Fauna	0	3
Residential Property Owners	Terrestrial	Fauna	4	6
Resource-Dependent Businesses	Aquatic	Fauna	1	0
Resource-Dependent Businesses	Created Greenspace	Fauna	0	1
Resource-Dependent Businesses	Estuaries and Near Coastal Marine	Fauna	3	2
Resource-Dependent Businesses	Forests	Fauna	1	0
Resource-Dependent Businesses	Open Oceans and Seas	Fauna	1	0
Resource-Dependent Businesses	Terrestrial	Fauna	1	1
Spiritual and Ceremonial Participants and Participants of Celebration	Barren_Rock and Sand	Fauna	0	1
Spiritual and Ceremonial Participants and Participants of Celebration	Created Greenspace	Fauna	1	0
Spiritual and Ceremonial Participants and Participants of Celebration	Estuaries and Near Coastal Marine	Fauna	0	5
Spiritual and Ceremonial Participants and Participants of Celebration	Open Oceans and Seas	Fauna	0	1
Subsistence	Estuaries and Near Coastal Marine	Fauna	0	1
Subsistence	Open Oceans and Seas	Fauna	0	1
Subsistence	Terrestrial	Fauna	0	1
Subsistence	Wetlands	Fauna	0	1
Timber and Fiber and Fur_Hide Subsisters	Aquatic	Fauna	1	2

Timber and Fiber and Fur_Hide Subsisters	Terrestrial	Fauna	2	3
Timber and Fiber and Ornamental Extractors	Aquatic	Fauna	1	0
Agricultural	Agroecosystems	Fiber	1	0
Agricultural Processors	Agroecosystems	Fiber	1	0
Artists	Rivers and Streams	Fiber	0	1
Artists	Terrestrial	Fiber	0	2
Building Material Subsisters	Estuaries and Near Coastal Marine	Fiber	0	1
Building Material Subsisters	Terrestrial	Fiber	0	1
Farmers	Agroecosystems	Fiber	2	0
Farmers	Terrestrial	Fiber	1	0
Government and Municipal and Residential	Agroecosystems	Fiber	2	0
Government and Municipal and Residential	Terrestrial	Fiber	1	0
Industrial Processors	Aquatic	Fiber	1	0
Industrial Processors	Estuaries and Near Coastal Marine	Fiber	0	1
Industrial Processors	Terrestrial	Fiber	0	1
Industrial Processors	Wetlands	Fiber	0	1
People Who Care (Existence)	Agroecosystems	Fiber	2	0
People Who Care (Existence)	Terrestrial	Fiber	1	0
Subsistence	Aquatic	Fiber	0	1
Subsistence	Estuaries and Near Coastal Marine	Fiber	0	2
Subsistence	Terrestrial	Fiber	0	2
Subsistence	Wetlands	Fiber	0	1
All Humans	Aquatic	Fish	20	7
All Humans	Barren_Rock and Sand	Fish	3	0
All Humans	Estuaries and Near Coastal Marine	Fish	12	0
All Humans	Open Oceans and Seas	Fish	5	1
All Humans	Rivers and Streams	Fish	2	0
All Humans	Wetlands	Fish	0	1
Anglers	Aquatic	Fish	27	29
Anglers	Barren_Rock and Sand	Fish	1	7
Anglers	Created Greenspace	Fish	3	3
Anglers	Estuaries and Near Coastal Marine	Fish	23	25
Anglers	Ice and Snow	Fish	0	3
Anglers	Lakes and Ponds	Fish	4	2
Anglers	Open Oceans and Seas	Fish	8	10
Anglers	Rivers and Streams	Fish	12	16
Anglers	Wetlands	Fish	8	9
Aquaculturists	Aquatic	Fish	18	14
Aquaculturists	Estuaries and Near Coastal Marine	Fish	9	4
Aquaculturists	Lakes and Ponds	Fish	0	2
Aquaculturists	Open Oceans and Seas	Fish	7	2
Aquaculturists	Rivers and Streams	Fish	3	1
Aquaculturists	Terrestrial	Fish	6	1
Aquaculturists	Wetlands	Fish	1	2
Artists	Aquatic	Fish	0	1
Boaters	Aquatic	Fish	14	13
Boaters	Estuaries and Near Coastal Marine	Fish	6	5
Boaters	Wetlands	Fish	0	1
Commerical_Industrial	Aquatic	Fish	23	21
Commerical_Industrial	Estuaries and Near Coastal Marine	Fish	5	5
Commerical_Industrial	Rivers and Streams	Fish	2	1
Commerical_Industrial	Wetlands	Fish	0	1
Educators and Students	Aquatic	Fish	18	18
Educators and Students	Estuaries and Near Coastal Marine	Fish	7	8
Educators and Students	Open Oceans and Seas	Fish	2	4
Educators and Students	Rivers and Streams	Fish	2	0
Educators and Students	Wetlands	Fish	1	3
Experiencers and Viewers	Aquatic	Fish	11	16
Experiencers and Viewers	Barren_Rock and Sand	Fish	0	2
Experiencers and Viewers	Estuaries and Near Coastal Marine	Fish	6	5
Experiencers and Viewers	Open Oceans and Seas	Fish	2	0
Experiencers and Viewers	Rivers and Streams	Fish	1	1
Experiencers and Viewers	Wetlands	Fish	1	2
Food Extractors	Aquatic	Fish	28	29
Food Extractors	Barren_Rock and Sand	Fish	4	4
Food Extractors	Created Greenspace	Fish	2	2
Food Extractors	Estuaries and Near Coastal Marine	Fish	28	29
Food Extractors	Forests	Fish	8	5
Food Extractors	Lakes and Ponds	Fish	8	5
Food Extractors	Open Oceans and Seas	Fish	26	25
Food Extractors	Rivers and Streams	Fish	21	19
Food Extractors	Terrestrial	Fish	24	19
Food Extractors	Wetlands	Fish	14	10
Food Pickers and Gatherers	Aquatic	Fish	22	21
Food Pickers and Gatherers	Barren_Rock and Sand	Fish	1	0
Food Pickers and Gatherers	Estuaries and Near Coastal Marine	Fish	18	16
Food Pickers and Gatherers	Forests	Fish	0	1
Food Pickers and Gatherers	Open Oceans and Seas	Fish	1	1

Food Pickers and Gatherers	Rivers and Streams	Fish	0	1
Food Pickers and Gatherers	Terrestrial	Fish	3	2
Food Pickers and Gatherers	Wetlands	Fish	2	3
Food Subsisters	Aquatic	Fish	8	18
Food Subsisters	Estuaries and Near Coastal Marine	Fish	7	10
Food Subsisters	Open Oceans and Seas	Fish	1	1
Food Subsisters	Rivers and Streams	Fish	2	1
Food Subsisters	Wetlands	Fish	0	1
Government and Municipal and Residential	Aquatic	Fish	25	24
Government and Municipal and Residential	Barren_Rock and Sand	Fish	2	0
Government and Municipal and Residential	Created Greenspace	Fish	0	1
Government and Municipal and Residential	Estuaries and Near Coastal Marine	Fish	20	21
Government and Municipal and Residential	Ice and Snow	Fish	0	2
Government and Municipal and Residential	Lakes and Ponds	Fish	2	1
Government and Municipal and Residential	Open Oceans and Seas	Fish	11	5
Government and Municipal and Residential	Rivers and Streams	Fish	8	3
Government and Municipal and Residential	Wetlands	Fish	5	2
Industrial Processors	Aquatic	Fish	16	11
Industrial Processors	Estuaries and Near Coastal Marine	Fish	5	2
Industrial Processors	Open Oceans and Seas	Fish	3	0
Industrial Processors	Rivers and Streams	Fish	3	0
Inspirational	Aquatic	Fish	5	4
Inspirational	Estuaries and Near Coastal Marine	Fish	2	1
Learning	Aquatic	Fish	13	19
Learning	Estuaries and Near Coastal Marine	Fish	7	12
Learning	Open Oceans and Seas	Fish	4	5
Learning	Rivers and Streams	Fish	0	1
Learning	Wetlands	Fish	1	1
People Who Care (Existence)	Aquatic	Fish	25	28
People Who Care (Existence)	Barren_Rock and Sand	Fish	1	0
People Who Care (Existence)	Estuaries and Near Coastal Marine	Fish	27	29
People Who Care (Existence)	Forests	Fish	1	1
People Who Care (Existence)	Lakes and Ponds	Fish	2	1
People Who Care (Existence)	Open Oceans and Seas	Fish	10	5
People Who Care (Existence)	Rivers and Streams	Fish	11	3
People Who Care (Existence)	Terrestrial	Fish	9	7
People Who Care (Existence)	Wetlands	Fish	9	7
People Who Care (Option_Bequest)	Aquatic	Fish	19	21
People Who Care (Option_Bequest)	Estuaries and Near Coastal Marine	Fish	13	7
People Who Care (Option_Bequest)	Open Oceans and Seas	Fish	3	1
People Who Care (Option_Bequest)	Rivers and Streams	Fish	4	1
People Who Care (Option_Bequest)	Wetlands	Fish	2	1
Pharmaceutical and Food Supplement Suppliers	Aquatic	Fish	6	0
Pharmaceutical and Food Supplement Suppliers	Estuaries and Near Coastal Marine	Fish	2	0
Pharmaceutical and Food Supplement Suppliers	Open Oceans and Seas	Fish	2	0
Recreational	Aquatic	Fish	25	27
Recreational	Created Greenspace	Fish	1	0
Recreational	Estuaries and Near Coastal Marine	Fish	17	14
Recreational	Forests	Fish	0	1
Recreational	Open Oceans and Seas	Fish	6	4
Recreational	Rivers and Streams	Fish	3	4
Researchers	Aquatic	Fish	12	15
Researchers	Estuaries and Near Coastal Marine	Fish	7	7
Researchers	Open Oceans and Seas	Fish	0	1
Researchers	Rivers and Streams	Fish	1	0
Researchers	Terrestrial	Fish	1	0
Residential Property Owners	Aquatic	Fish	7	7
Residential Property Owners	Open Oceans and Seas	Fish	0	1
Residential Property Owners	Rivers and Streams	Fish	0	1
Resource-Dependent Businesses	Aquatic	Fish	20	14
Resource-Dependent Businesses	Estuaries and Near Coastal Marine	Fish	8	5
Resource-Dependent Businesses	Open Oceans and Seas	Fish	0	2
Resource-Dependent Businesses	Rivers and Streams	Fish	0	1
Resource-Dependent Businesses	Terrestrial	Fish	0	1
Spiritual and Ceremonial Participants and Participants of Celebration	Aquatic	Fish	4	4
Spiritual and Ceremonial Participants and Participants of Celebration	Open Oceans and Seas	Fish	0	1
Spiritual and Ceremonial Participants and Participants of Celebration	Rivers and Streams	Fish	1	1
Subsistence	Aquatic	Fish	7	15
Subsistence	Estuaries and Near Coastal Marine	Fish	1	4
Subsistence	Open Oceans and Seas	Fish	1	0
Subsistence	Rivers and Streams	Fish	0	1
Subsistence	Wetlands	Fish	0	1
Timber and Fiber and Ornamental Extractors	Aquatic	Fish	4	1
Timber and Fiber and Ornamental Extractors	Estuaries and Near Coastal Marine	Fish	3	0
Transporters of Goods	Aquatic	Fish	8	2
Transporters of Goods	Estuaries and Near Coastal Marine	Fish	0	1
Waders and Swimmers and Divers	Aquatic	Fish	9	1
Waders and Swimmers and Divers	Estuaries and Near Coastal Marine	Fish	2	2

Agricultural	Agroecosystems	Flora	9	11
All Humans	Estuaries and Near Coastal Marine	Flora	1	0
All Humans	Terrestrial	Flora	3	4
Aquaculturists	Aquatic	Flora	0	2
Aquaculturists	Estuaries and Near Coastal Marine	Flora	1	1
Aquaculturists	Open Oceans and Seas	Flora	0	1
Artists	Created Greenspace	Flora	0	1
Artists	Terrestrial	Flora	0	2
Boaters	Aquatic	Flora	4	4
Boaters	Estuaries and Near Coastal Marine	Flora	5	1
Boaters	Forests	Flora	1	0
Boaters	Terrestrial	Flora	1	0
CAFO Operators	Terrestrial	Flora	0	1
Commercial_Military Transportation	Agroecosystems	Flora	1	2
Commercial_Military Transportation	Forests	Flora	1	2
Commercial_Military Transportation	Terrestrial	Flora	0	1
Commerical_Industrial	Aquatic	Flora	2	2
Commerical_Industrial	Estuaries and Near Coastal Marine	Flora	1	3
Commerical_Industrial	Forests	Flora	2	1
Commerical_Industrial	Open Oceans and Seas	Flora	2	1
Commerical_Industrial	Rivers and Streams	Flora	1	0
Commerical_Industrial	Scrublands_Shrublands	Flora	0	1
Commerical_Industrial	Terrestrial	Flora	1	0
Commerical_Industrial	Wetlands	Flora	2	2
Educators and Students	Aquatic	Flora	1	1
Educators and Students	Created Greenspace	Flora	0	1
Educators and Students	Estuaries and Near Coastal Marine	Flora	5	15
Educators and Students	Forests	Flora	1	5
Educators and Students	Grasslands	Flora	0	1
Educators and Students	Lakes and Ponds	Flora	1	0
Educators and Students	Rivers and Streams	Flora	1	0
Educators and Students	Scrublands_Shrublands	Flora	1	3
Educators and Students	Terrestrial	Flora	6	8
Educators and Students	Wetlands	Flora	0	2
Experiencers and Viewers	Aquatic	Flora	1	2
Experiencers and Viewers	Barren_Rock and Sand	Flora	0	2
Experiencers and Viewers	Created Greenspace	Flora	0	10
Experiencers and Viewers	Estuaries and Near Coastal Marine	Flora	3	7
Experiencers and Viewers	Forests	Flora	1	3
Experiencers and Viewers	Grasslands	Flora	0	1
Experiencers and Viewers	Scrublands_Shrublands	Flora	0	2
Experiencers and Viewers	Terrestrial	Flora	4	19
Experiencers and Viewers	Wetlands	Flora	0	2
Farmers	Agroecosystems	Flora	5	3
Farmers	Grasslands	Flora	0	1
Farmers	Wetlands	Flora	1	0
Food Extractors	Aquatic	Flora	9	5
Food Extractors	Estuaries and Near Coastal Marine	Flora	2	2
Food Pickers and Gatherers	Aquatic	Flora	1	1
Food Pickers and Gatherers	Forests	Flora	0	2
Food Pickers and Gatherers	Open Oceans and Seas	Flora	0	2
Food Subsisters	Aquatic	Flora	0	1
Food Subsisters	Forests	Flora	0	2
Food Subsisters	Open Oceans and Seas	Flora	0	1
Food Subsisters	Terrestrial	Flora	0	3
Food Subsisters	Wetlands	Flora	0	1
Foresters	Agroecosystems	Flora	5	1
Foresters	Aquatic	Flora	2	2
Foresters	Estuaries and Near Coastal Marine	Flora	0	3
Foresters	Forests	Flora	13	19
Foresters	Rivers and Streams	Flora	1	1
Foresters	Terrestrial	Flora	2	1
Foresters	Wetlands	Flora	1	3
Government and Municipal and Residential	Agroecosystems	Flora	5	4
Government and Municipal and Residential	Aquatic	Flora	4	5
Government and Municipal and Residential	Created Greenspace	Flora	4	3
Government and Municipal and Residential	Estuaries and Near Coastal Marine	Flora	15	15
Government and Municipal and Residential	Forests	Flora	10	12
Government and Municipal and Residential	Grasslands	Flora	0	1
Government and Municipal and Residential	Lakes and Ponds	Flora	2	1
Government and Municipal and Residential	Open Oceans and Seas	Flora	2	1
Government and Municipal and Residential	Rivers and Streams	Flora	5	0
Government and Municipal and Residential	Terrestrial	Flora	14	17
Government and Municipal and Residential	Wetlands	Flora	2	2
Industrial Processors	Aquatic	Flora	1	1
Industrial Processors	Estuaries and Near Coastal Marine	Flora	0	1
Industrial Processors	Forests	Flora	3	1
Inspirational	Estuaries and Near Coastal Marine	Flora	0	1

Inspirational	Forests	Flora	0	2
Learning	Created Greenspace	Flora	1	3
Learning	Estuaries and Near Coastal Marine	Flora	6	9
Learning	Forests	Flora	0	2
Learning	Open Oceans and Seas	Flora	1	0
Learning	Scrublands_Shrublands	Flora	0	1
Learning	Terrestrial	Flora	5	13
Learning	Wetlands	Flora	0	3
Livestock Grazers	Agroecosystems	Flora	2	2
Livestock Grazers	Aquatic	Flora	1	1
Livestock Grazers	Forests	Flora	1	3
Livestock Grazers	Grasslands	Flora	0	2
Livestock Grazers	Scrublands_Shrublands	Flora	0	1
Livestock Grazers	Terrestrial	Flora	2	2
Livestock Grazers	Wetlands	Flora	0	2
Military_Coast Guard	Estuaries and Near Coastal Marine	Flora	2	0
Military_Coast Guard	Terrestrial	Flora	0	1
People Who Care (Existence)	Agroecosystems	Flora	1	4
People Who Care (Existence)	Aquatic	Flora	18	19
People Who Care (Existence)	Barren_Rock and Sand	Flora	2	3
People Who Care (Existence)	Created Greenspace	Flora	0	1
People Who Care (Existence)	Estuaries and Near Coastal Marine	Flora	27	29
People Who Care (Existence)	Forests	Flora	11	19
People Who Care (Existence)	Grasslands	Flora	1	4
People Who Care (Existence)	Lakes and Ponds	Flora	2	3
People Who Care (Existence)	Open Oceans and Seas	Flora	5	6
People Who Care (Existence)	Rivers and Streams	Flora	6	4
People Who Care (Existence)	Scrublands_Shrublands	Flora	3	6
People Who Care (Existence)	Terrestrial	Flora	21	28
People Who Care (Existence)	Wetlands	Flora	11	16
People Who Care (Option_Bequest)	Aquatic	Flora	1	1
People Who Care (Option_Bequest)	Created Greenspace	Flora	3	3
People Who Care (Option_Bequest)	Estuaries and Near Coastal Marine	Flora	8	13
People Who Care (Option_Bequest)	Forests	Flora	2	3
People Who Care (Option_Bequest)	Grasslands	Flora	0	3
People Who Care (Option_Bequest)	Terrestrial	Flora	4	8
People Who Care (Option_Bequest)	Wetlands	Flora	1	8
Pharmaceutical and Food Supplement Suppliers	Terrestrial	Flora	0	1
Recreational	Aquatic	Flora	1	1
Recreational	Created Greenspace	Flora	1	1
Recreational	Estuaries and Near Coastal Marine	Flora	10	7
Recreational	Forests	Flora	1	5
Recreational	Grasslands	Flora	0	1
Recreational	Open Oceans and Seas	Flora	1	0
Recreational	Terrestrial	Flora	5	7
Recreational	Wetlands	Flora	0	3
Researchers	Estuaries and Near Coastal Marine	Flora	1	20
Researchers	Forests	Flora	1	0
Researchers	Terrestrial	Flora	0	4
Researchers	Wetlands	Flora	0	1
Residential Property Owners	Aquatic	Flora	1	1
Residential Property Owners	Estuaries and Near Coastal Marine	Flora	1	1
Residential Property Owners	Forests	Flora	3	2
Residential Property Owners	Terrestrial	Flora	2	3
Resource-Dependent Businesses	Created Greenspace	Flora	1	1
Resource-Dependent Businesses	Estuaries and Near Coastal Marine	Flora	3	2
Resource-Dependent Businesses	Open Oceans and Seas	Flora	1	0
Resource-Dependent Businesses	Terrestrial	Flora	8	9
Resource-Dependent Businesses	Wetlands	Flora	2	4
Spiritual and Ceremonial Participants and Participants of Celebration	Estuaries and Near Coastal Marine	Flora	0	1
Spiritual and Ceremonial Participants and Participants of Celebration	Open Oceans and Seas	Flora	0	1
Spiritual and Ceremonial Participants and Participants of Celebration	Rivers and Streams	Flora	1	0
Spiritual and Ceremonial Participants and Participants of Celebration	Terrestrial	Flora	1	0
Subsistence	Agroecosystems	Flora	1	0
Subsistence	Open Oceans and Seas	Flora	0	1
Timber and Fiber and Ornamental Extractors	Aquatic	Flora	1	0
Timber and Fiber and Ornamental Extractors	Forests	Flora	2	4
Timber and Fiber and Ornamental Extractors	Terrestrial	Flora	6	5
Agricultural	Agroecosystems	Fungi	1	0
Educators and Students	Agroecosystems	Fungi	1	0
Experiencers and Viewers	Forests	Fungi	0	1
Experiencers and Viewers	Rivers and Streams	Fungi	0	1
Agricultural	Agroecosystems	Land	16	19
Agricultural	Aquatic	Land	1	1
Agricultural	Estuaries and Near Coastal Marine	Land	1	3
Agricultural	Forests	Land	5	1
Agricultural	Grasslands	Land	1	1
Agricultural	Lakes and Ponds	Land	0	1

Agricultural	Rivers and Streams	Land	2	2
Agricultural	Scrublands_Shrublands	Land	0	1
Agricultural	Terrestrial	Land	1	3
Agricultural	Wetlands	Land	3	5
All Humans	Estuaries and Near Coastal Marine	Land	1	0
All Humans	Terrestrial	Land	2	1
All Humans	Wetlands	Land	0	1
Anglers	Terrestrial	Land	0	1
Aquaculturists	Estuaries and Near Coastal Marine	Land	1	0
Aquaculturists	Terrestrial	Land	2	0
Boaters	Aquatic	Land	2	4
Boaters	Estuaries and Near Coastal Marine	Land	3	3
Boaters	Rivers and Streams	Land	1	3
Boaters	Terrestrial	Land	0	4
Commercial_Military Transportation	Agroecosystems	Land	1	0
Commercial_Military Transportation	Aquatic	Land	2	0
Commercial_Military Transportation	Estuaries and Near Coastal Marine	Land	6	7
Commercial_Military Transportation	Forests	Land	1	0
Commercial_Military Transportation	Rivers and Streams	Land	1	1
Commercial_Military Transportation	Terrestrial	Land	3	5
Commerical_Industrial	Agroecosystems	Land	2	1
Commerical_Industrial	Aquatic	Land	1	0
Commerical_Industrial	Estuaries and Near Coastal Marine	Land	4	7
Commerical_Industrial	Forests	Land	1	0
Commerical_Industrial	Rivers and Streams	Land	1	2
Commerical_Industrial	Terrestrial	Land	4	21
Commerical_Industrial	Wetlands	Land	4	1
Educators and Students	Agroecosystems	Land	0	1
Educators and Students	Estuaries and Near Coastal Marine	Land	3	14
Educators and Students	Lakes and Ponds	Land	0	1
Educators and Students	Rivers and Streams	Land	1	0
Educators and Students	Terrestrial	Land	6	16
Electric and other Energy Generators	Aquatic	Land	0	1
Electric and other Energy Generators	Estuaries and Near Coastal Marine	Land	1	1
Experiencers and Viewers	Aquatic	Land	2	3
Experiencers and Viewers	Barren_Rock and Sand	Land	0	1
Experiencers and Viewers	Created Greenspace	Land	0	3
Experiencers and Viewers	Estuaries and Near Coastal Marine	Land	1	2
Experiencers and Viewers	Forests	Land	0	1
Experiencers and Viewers	Rivers and Streams	Land	0	2
Experiencers and Viewers	Terrestrial	Land	3	7
Experiencers and Viewers	Wetlands	Land	0	1
Farmers	Agroecosystems	Land	13	13
Farmers	Aquatic	Land	3	4
Farmers	Estuaries and Near Coastal Marine	Land	2	1
Farmers	Forests	Land	1	2
Farmers	Grasslands	Land	0	1
Farmers	Scrublands_Shrublands	Land	0	2
Farmers	Terrestrial	Land	3	5
Farmers	Wetlands	Land	3	1
Foresters	Agroecosystems	Land	1	4
Foresters	Forests	Land	1	4
Foresters	Terrestrial	Land	1	0
Foresters	Wetlands	Land	1	0
Government and Municipal and Residential	Agroecosystems	Land	9	7
Government and Municipal and Residential	Aquatic	Land	14	8
Government and Municipal and Residential	Barren_Rock and Sand	Land	3	2
Government and Municipal and Residential	Created Greenspace	Land	7	6
Government and Municipal and Residential	Estuaries and Near Coastal Marine	Land	25	27
Government and Municipal and Residential	Forests	Land	8	14
Government and Municipal and Residential	Grasslands	Land	0	3
Government and Municipal and Residential	Lakes and Ponds	Land	3	3
Government and Municipal and Residential	Rivers and Streams	Land	13	9
Government and Municipal and Residential	Scrublands_Shrublands	Land	3	3
Government and Municipal and Residential	Terrestrial	Land	25	28
Government and Municipal and Residential	Wetlands	Land	20	19
Hunters	Aquatic	Land	0	1
Hunters	Terrestrial	Land	0	1
Industrial Dischargers	Estuaries and Near Coastal Marine	Land	3	2
Industrial Dischargers	Rivers and Streams	Land	0	1
Industrial Dischargers	Scrublands_Shrublands	Land	0	1
Industrial Dischargers	Terrestrial	Land	6	1
Industrial Dischargers	Wetlands	Land	2	3
Industrial Processors	Agroecosystems	Land	1	0
Industrial Processors	Barren_Rock and Sand	Land	1	2
Industrial Processors	Estuaries and Near Coastal Marine	Land	3	2
Inspirational	Estuaries and Near Coastal Marine	Land	1	1
Inspirational	Terrestrial	Land	1	4

Irrigators	Estuaries and Near Coastal Marine	Land	2	0
Irrigators	Rivers and Streams	Land	1	0
Irrigators	Terrestrial	Land	2	0
Irrigators	Wetlands	Land	0	1
Learning	Created Greenspace	Land	0	1
Learning	Estuaries and Near Coastal Marine	Land	3	19
Learning	Terrestrial	Land	6	25
Learning	Wetlands	Land	0	2
Livestock Grazers	Agroecosystems	Land	2	1
Livestock Grazers	Estuaries and Near Coastal Marine	Land	1	0
Livestock Grazers	Forests	Land	0	1
Livestock Grazers	Grasslands	Land	0	1
Livestock Grazers	Scrublands_Shrublands	Land	0	1
Livestock Grazers	Terrestrial	Land	2	1
Livestock Grazers	Wetlands	Land	1	1
Military_Coast Guard	Estuaries and Near Coastal Marine	Land	2	3
Military_Coast Guard	Rivers and Streams	Land	2	0
Military_Coast Guard	Terrestrial	Land	1	1
Military_Coast Guard	Wetlands	Land	1	0
People Who Care (Existence)	Agroecosystems	Land	3	6
People Who Care (Existence)	Aquatic	Land	8	6
People Who Care (Existence)	Created Greenspace	Land	3	1
People Who Care (Existence)	Estuaries and Near Coastal Marine	Land	25	26
People Who Care (Existence)	Forests	Land	3	2
People Who Care (Existence)	Rivers and Streams	Land	10	9
People Who Care (Existence)	Scrublands_Shrublands	Land	0	1
People Who Care (Existence)	Terrestrial	Land	22	28
People Who Care (Existence)	Wetlands	Land	13	14
People Who Care (Option_Bequest)	Agroecosystems	Land	2	0
People Who Care (Option_Bequest)	Aquatic	Land	1	1
People Who Care (Option_Bequest)	Created Greenspace	Land	1	1
People Who Care (Option_Bequest)	Estuaries and Near Coastal Marine	Land	7	16
People Who Care (Option_Bequest)	Forests	Land	0	1
People Who Care (Option_Bequest)	Grasslands	Land	0	1
People Who Care (Option_Bequest)	Rivers and Streams	Land	2	2
People Who Care (Option_Bequest)	Terrestrial	Land	15	28
People Who Care (Option_Bequest)	Wetlands	Land	1	3
Recreational	Created Greenspace	Land	2	2
Recreational	Estuaries and Near Coastal Marine	Land	3	2
Recreational	Rivers and Streams	Land	1	2
Recreational	Terrestrial	Land	4	12
Recreational	Wetlands	Land	0	1
Researchers	Agroecosystems	Land	1	0
Researchers	Aquatic	Land	0	1
Researchers	Estuaries and Near Coastal Marine	Land	2	10
Researchers	Forests	Land	1	0
Researchers	Terrestrial	Land	0	11
Researchers	Wetlands	Land	1	2
Residential Property Owners	Agroecosystems	Land	4	5
Residential Property Owners	Aquatic	Land	1	2
Residential Property Owners	Estuaries and Near Coastal Marine	Land	10	11
Residential Property Owners	Forests	Land	6	3
Residential Property Owners	Rivers and Streams	Land	2	2
Residential Property Owners	Terrestrial	Land	14	17
Residential Property Owners	Wetlands	Land	2	3
Resource-Dependent Businesses	Aquatic	Land	0	1
Resource-Dependent Businesses	Estuaries and Near Coastal Marine	Land	6	3
Resource-Dependent Businesses	Rivers and Streams	Land	1	1
Resource-Dependent Businesses	Terrestrial	Land	2	1
Timber and Fiber and Ornamental Extractors	Terrestrial	Land	0	1
Transporters of People	Barren_Rock and Sand	Land	1	0
Transporters of People	Scrublands_Shrublands	Land	1	0
Transporters of People	Terrestrial	Land	0	3
Waders and Swimmers and Divers	Estuaries and Near Coastal Marine	Land	1	2
Waders and Swimmers and Divers	Terrestrial	Land	2	2
Wastewater Treatment Plant Operators	Estuaries and Near Coastal Marine	Land	0	1
Artists	Aquatic	Natural Materials	0	1
Commercial_Military Transportation	Aquatic	Natural Materials	1	0
Commercial_Military Transportation	Barren_Rock and Sand	Natural Materials	2	3
Commercial_Military Transportation	Estuaries and Near Coastal Marine	Natural Materials	2	0
Commerical_Industrial	Barren_Rock and Sand	Natural Materials	2	2
Commerical_Industrial	Estuaries and Near Coastal Marine	Natural Materials	2	1
Commerical_Industrial	Terrestrial	Natural Materials	1	2
Experiencers and Viewers	Aquatic	Natural Materials	1	0
Experiencers and Viewers	Barren_Rock and Sand	Natural Materials	2	5
Experiencers and Viewers	Estuaries and Near Coastal Marine	Natural Materials	2	2
Experiencers and Viewers	Rivers and Streams	Natural Materials	1	1
Experiencers and Viewers	Terrestrial	Natural Materials	0	2

Experiencers and Viewers	Wetlands	Natural Materials	0	1
Food Extractors	Aquatic	Natural Materials	5	6
Food Extractors	Estuaries and Near Coastal Marine	Natural Materials	1	0
Government and Municipal and Residential	Aquatic	Natural Materials	0	1
Government and Municipal and Residential	Barren_Rock and Sand	Natural Materials	10	4
Government and Municipal and Residential	Estuaries and Near Coastal Marine	Natural Materials	5	0
Government and Municipal and Residential	Rivers and Streams	Natural Materials	1	0
Government and Municipal and Residential	Terrestrial	Natural Materials	9	0
Industrial Dischargers	Terrestrial	Natural Materials	3	1
Industrial Processors	Aquatic	Natural Materials	6	4
Industrial Processors	Barren_Rock and Sand	Natural Materials	15	4
Industrial Processors	Estuaries and Near Coastal Marine	Natural Materials	4	4
Industrial Processors	Lakes and Ponds	Natural Materials	4	1
Industrial Processors	Open Oceans and Seas	Natural Materials	1	1
Industrial Processors	Rivers and Streams	Natural Materials	7	2
Industrial Processors	Terrestrial	Natural Materials	4	2
Industrial Processors	Wetlands	Natural Materials	2	3
Military_Coast Guard	Estuaries and Near Coastal Marine	Natural Materials	2	1
Military_Coast Guard	Rivers and Streams	Natural Materials	1	0
People Who Care (Existence)	Barren_Rock and Sand	Natural Materials	5	1
People Who Care (Existence)	Estuaries and Near Coastal Marine	Natural Materials	5	1
People Who Care (Existence)	Forests	Natural Materials	2	3
People Who Care (Existence)	Terrestrial	Natural Materials	3	4
People Who Care (Existence)	Wetlands	Natural Materials	4	3
Subsistence	Estuaries and Near Coastal Marine	Natural Materials	0	1
Timber and Fiber and Fur_Hide Subsisters	Aquatic	Natural Materials	0	2
Timber and Fiber and Fur_Hide Subsisters	Forests	Natural Materials	3	5
Timber and Fiber and Fur_Hide Subsisters	Terrestrial	Natural Materials	1	3
Timber and Fiber and Fur_Hide Subsisters	Wetlands	Natural Materials	0	2
Agricultural	Agroecosystems	Open Space	2	1
Anglers	Aquatic	Open Space	0	1
Anglers	Terrestrial	Open Space	0	1
Commerical_Industrial	Agroecosystems	Open Space	1	0
Commerical_Industrial	Created Greenspace	Open Space	1	0
Commerical_Industrial	Terrestrial	Open Space	0	1
Educators and Students	Created Greenspace	Open Space	1	0
Experiencers and Viewers	Aquatic	Open Space	0	1
Experiencers and Viewers	Created Greenspace	Open Space	1	0
Experiencers and Viewers	Terrestrial	Open Space	0	1
Farmers	Agroecosystems	Open Space	2	2
Government and Municipal and Residential	Agroecosystems	Open Space	5	2
Government and Municipal and Residential	Aquatic	Open Space	10	3
Government and Municipal and Residential	Barren_Rock and Sand	Open Space	1	0
Government and Municipal and Residential	Created Greenspace	Open Space	6	3
Government and Municipal and Residential	Estuaries and Near Coastal Marine	Open Space	11	7
Government and Municipal and Residential	Forests	Open Space	5	3
Government and Municipal and Residential	Grasslands	Open Space	0	1
Government and Municipal and Residential	Lakes and Ponds	Open Space	1	0
Government and Municipal and Residential	Rivers and Streams	Open Space	7	2
Government and Municipal and Residential	Terrestrial	Open Space	16	15
Government and Municipal and Residential	Wetlands	Open Space	6	3
Hunters	Aquatic	Open Space	0	1
Hunters	Terrestrial	Open Space	0	1
People Who Care (Existence)	Agroecosystems	Open Space	1	0
People Who Care (Existence)	Aquatic	Open Space	0	1
People Who Care (Existence)	Barren_Rock and Sand	Open Space	0	1
People Who Care (Existence)	Estuaries and Near Coastal Marine	Open Space	2	0
People Who Care (Existence)	Forests	Open Space	0	1
People Who Care (Existence)	Terrestrial	Open Space	2	0
People Who Care (Option_Bequest)	Aquatic	Open Space	0	1
People Who Care (Option_Bequest)	Estuaries and Near Coastal Marine	Open Space	1	0
Recreational	Agroecosystems	Open Space	1	0
Recreational	Aquatic	Open Space	1	0
Recreational	Created Greenspace	Open Space	4	1
Recreational	Estuaries and Near Coastal Marine	Open Space	1	0
Recreational	Forests	Open Space	1	0
Recreational	Terrestrial	Open Space	1	1
Residential Property Owners	Agroecosystems	Open Space	0	1
Residential Property Owners	Created Greenspace	Open Space	1	0
Residential Property Owners	Estuaries and Near Coastal Marine	Open Space	1	0
Residential Property Owners	Terrestrial	Open Space	1	0
Resource-Dependent Businesses	Agroecosystems	Open Space	1	0
Resource-Dependent Businesses	Terrestrial	Open Space	1	0
Agricultural	Agroecosystems	Pollinators	1	1
Artists	Terrestrial	Pollinators	0	1
Experiencers and Viewers	Created Greenspace	Pollinators	0	2
Experiencers and Viewers	Terrestrial	Pollinators	0	1
Government and Municipal and Residential	Aquatic	Pollinators	0	1

Government and Municipal and Residential	Created Greenspace	Pollinators	0	1
Learning	Created Greenspace	Pollinators	0	2
Learning	Terrestrial	Pollinators	0	2
People Who Care (Existence)	Wetlands	Pollinators	0	1
Recreational	Created Greenspace	Pollinators	0	2
Researchers	Agroecosystems	Pollinators	0	1
Agricultural	Agroecosystems	Presence of the Environment	25	24
Agricultural	Aquatic	Presence of the Environment	9	2
Agricultural	Estuaries and Near Coastal Marine	Presence of the Environment	10	11
Agricultural	Forests	Presence of the Environment	4	4
Agricultural	Groundwater	Presence of the Environment	2	4
Agricultural	Lakes and Ponds	Presence of the Environment	0	1
Agricultural	Rivers and Streams	Presence of the Environment	5	1
Agricultural	Scrublands_Shrublands	Presence of the Environment	1	2
Agricultural	Terrestrial	Presence of the Environment	8	4
Agricultural	Wetlands	Presence of the Environment	10	5
Agricultural Processors	Agroecosystems	Presence of the Environment	5	2
Agricultural Processors	Aquatic	Presence of the Environment	0	1
Agricultural Processors	Estuaries and Near Coastal Marine	Presence of the Environment	0	1
All Humans	Agroecosystems	Presence of the Environment	2	0
All Humans	Aquatic	Presence of the Environment	10	4
All Humans	Barren_Rock and Sand	Presence of the Environment	2	2
All Humans	Created Greenspace	Presence of the Environment	0	9
All Humans	Estuaries and Near Coastal Marine	Presence of the Environment	25	21
All Humans	Groundwater	Presence of the Environment	1	0
All Humans	Lakes and Ponds	Presence of the Environment	0	1
All Humans	Open Oceans and Seas	Presence of the Environment	5	4
All Humans	Rivers and Streams	Presence of the Environment	8	1
All Humans	Terrestrial	Presence of the Environment	19	15
All Humans	Wetlands	Presence of the Environment	4	5
Anglers	Aquatic	Presence of the Environment	6	10
Anglers	Estuaries and Near Coastal Marine	Presence of the Environment	6	6
Anglers	Rivers and Streams	Presence of the Environment	2	0
Aquaculturists	Aquatic	Presence of the Environment	5	2
Aquaculturists	Estuaries and Near Coastal Marine	Presence of the Environment	14	6
Aquaculturists	Open Oceans and Seas	Presence of the Environment	1	3
Aquaculturists	Rivers and Streams	Presence of the Environment	2	0
Aquaculturists	Terrestrial	Presence of the Environment	1	2
Aquaculturists	Wetlands	Presence of the Environment	2	1
Artists	Created Greenspace	Presence of the Environment	1	6
Artists	Estuaries and Near Coastal Marine	Presence of the Environment	6	8
Artists	Open Oceans and Seas	Presence of the Environment	2	1
Artists	Rivers and Streams	Presence of the Environment	1	0
Artists	Terrestrial	Presence of the Environment	1	6
Boaters	Aquatic	Presence of the Environment	25	25
Boaters	Barren_Rock and Sand	Presence of the Environment	3	3
Boaters	Created Greenspace	Presence of the Environment	7	10
Boaters	Estuaries and Near Coastal Marine	Presence of the Environment	21	19
Boaters	Forests	Presence of the Environment	2	0
Boaters	Lakes and Ponds	Presence of the Environment	1	1
Boaters	Open Oceans and Seas	Presence of the Environment	2	4
Boaters	Rivers and Streams	Presence of the Environment	11	10
Boaters	Terrestrial	Presence of the Environment	8	12
Boaters	Wetlands	Presence of the Environment	8	11
CAFO Operators	Agroecosystems	Presence of the Environment	4	3
CAFO Operators	Aquatic	Presence of the Environment	3	0
CAFO Operators	Estuaries and Near Coastal Marine	Presence of the Environment	2	1
CAFO Operators	Rivers and Streams	Presence of the Environment	1	0
CAFO Operators	Terrestrial	Presence of the Environment	1	0
CAFO Operators	Wetlands	Presence of the Environment	1	0
Commercial_Military Transportation	Agroecosystems	Presence of the Environment	1	2
Commercial_Military Transportation	Aquatic	Presence of the Environment	9	7
Commercial_Military Transportation	Barren_Rock and Sand	Presence of the Environment	1	0
Commercial_Military Transportation	Created Greenspace	Presence of the Environment	3	11
Commercial_Military Transportation	Estuaries and Near Coastal Marine	Presence of the Environment	24	19
Commercial_Military Transportation	Forests	Presence of the Environment	3	2
Commercial_Military Transportation	Lakes and Ponds	Presence of the Environment	2	0
Commercial_Military Transportation	Open Oceans and Seas	Presence of the Environment	0	3
Commercial_Military Transportation	Rivers and Streams	Presence of the Environment	9	5
Commercial_Military Transportation	Terrestrial	Presence of the Environment	11	11
Commercial_Military Transportation	Wetlands	Presence of the Environment	4	7
Commerical_Industrial	Agroecosystems	Presence of the Environment	12	10
Commerical_Industrial	Aquatic	Presence of the Environment	17	8
Commerical_Industrial	Barren_Rock and Sand	Presence of the Environment	3	1
Commerical_Industrial	Created Greenspace	Presence of the Environment	6	3
Commerical_Industrial	Estuaries and Near Coastal Marine	Presence of the Environment	27	25
Commerical_Industrial	Forests	Presence of the Environment	5	5
Commerical_Industrial	Groundwater	Presence of the Environment	6	2

Commerical_Industrial	Lakes and Ponds	Presence of the Environment	1	0
Commerical_Industrial	Open Oceans and Seas	Presence of the Environment	6	3
Commerical_Industrial	Rivers and Streams	Presence of the Environment	9	8
Commerical_Industrial	Scrublands_Shrublands	Presence of the Environment	2	2
Commerical_Industrial	Terrestrial	Presence of the Environment	24	21
Commerical_Industrial	Wetlands	Presence of the Environment	6	7
Educators and Students	Agroecosystems	Presence of the Environment	6	3
Educators and Students	Aquatic	Presence of the Environment	27	29
Educators and Students	Barren_Rock and Sand	Presence of the Environment	4	6
Educators and Students	Created Greenspace	Presence of the Environment	13	28
Educators and Students	Estuaries and Near Coastal Marine	Presence of the Environment	28	29
Educators and Students	Forests	Presence of the Environment	1	10
Educators and Students	Grasslands	Presence of the Environment	1	1
Educators and Students	Ice and Snow	Presence of the Environment	0	2
Educators and Students	Lakes and Ponds	Presence of the Environment	1	3
Educators and Students	Open Oceans and Seas	Presence of the Environment	14	22
Educators and Students	Rivers and Streams	Presence of the Environment	14	18
Educators and Students	Scrublands_Shrublands	Presence of the Environment	0	1
Educators and Students	Terrestrial	Presence of the Environment	24	29
Educators and Students	Wetlands	Presence of the Environment	13	23
Electric and other Energy Generators	Agroecosystems	Presence of the Environment	2	1
Electric and other Energy Generators	Aquatic	Presence of the Environment	6	1
Electric and other Energy Generators	Atmosphere	Presence of the Environment	2	0
Electric and other Energy Generators	Created Greenspace	Presence of the Environment	0	2
Electric and other Energy Generators	Estuaries and Near Coastal Marine	Presence of the Environment	12	11
Electric and other Energy Generators	Lakes and Ponds	Presence of the Environment	0	2
Electric and other Energy Generators	Rivers and Streams	Presence of the Environment	3	1
Electric and other Energy Generators	Terrestrial	Presence of the Environment	1	4
Electric and other Energy Generators	Wetlands	Presence of the Environment	2	0
Experiencers and Viewers	Agroecosystems	Presence of the Environment	1	3
Experiencers and Viewers	Aquatic	Presence of the Environment	25	29
Experiencers and Viewers	Atmosphere	Presence of the Environment	3	5
Experiencers and Viewers	Barren_Rock and Sand	Presence of the Environment	19	22
Experiencers and Viewers	Created Greenspace	Presence of the Environment	22	29
Experiencers and Viewers	Estuaries and Near Coastal Marine	Presence of the Environment	27	29
Experiencers and Viewers	Forests	Presence of the Environment	8	24
Experiencers and Viewers	Grasslands	Presence of the Environment	0	6
Experiencers and Viewers	Ice and Snow	Presence of the Environment	0	7
Experiencers and Viewers	Lakes and Ponds	Presence of the Environment	4	10
Experiencers and Viewers	Open Oceans and Seas	Presence of the Environment	13	15
Experiencers and Viewers	Rivers and Streams	Presence of the Environment	16	24
Experiencers and Viewers	Scrublands_Shrublands	Presence of the Environment	4	12
Experiencers and Viewers	Terrestrial	Presence of the Environment	18	26
Experiencers and Viewers	Wetlands	Presence of the Environment	12	26
Farmers	Agroecosystems	Presence of the Environment	18	18
Farmers	Aquatic	Presence of the Environment	1	0
Farmers	Estuaries and Near Coastal Marine	Presence of the Environment	7	3
Farmers	Forests	Presence of the Environment	3	1
Farmers	Groundwater	Presence of the Environment	2	0
Farmers	Lakes and Ponds	Presence of the Environment	1	0
Farmers	Rivers and Streams	Presence of the Environment	5	2
Farmers	Scrublands_Shrublands	Presence of the Environment	1	1
Farmers	Terrestrial	Presence of the Environment	8	8
Farmers	Wetlands	Presence of the Environment	8	6
Food Extractors	Aquatic	Presence of the Environment	22	19
Food Extractors	Estuaries and Near Coastal Marine	Presence of the Environment	28	28
Food Extractors	Open Oceans and Seas	Presence of the Environment	1	1
Food Extractors	Rivers and Streams	Presence of the Environment	6	2
Food Extractors	Terrestrial	Presence of the Environment	1	1
Food Extractors	Wetlands	Presence of the Environment	3	1
Food Pickers and Gatherers	Aquatic	Presence of the Environment	3	3
Food Pickers and Gatherers	Estuaries and Near Coastal Marine	Presence of the Environment	4	2
Food Pickers and Gatherers	Open Oceans and Seas	Presence of the Environment	2	0
Food Pickers and Gatherers	Rivers and Streams	Presence of the Environment	0	1
Food Pickers and Gatherers	Terrestrial	Presence of the Environment	2	3
Food Pickers and Gatherers	Wetlands	Presence of the Environment	1	1
Food Subsisters	Aquatic	Presence of the Environment	2	3
Food Subsisters	Estuaries and Near Coastal Marine	Presence of the Environment	2	7
Food Subsisters	Forests	Presence of the Environment	0	1
Food Subsisters	Open Oceans and Seas	Presence of the Environment	0	1
Food Subsisters	Terrestrial	Presence of the Environment	0	2
Food Subsisters	Wetlands	Presence of the Environment	0	1
Foresters	Agroecosystems	Presence of the Environment	1	4
Foresters	Aquatic	Presence of the Environment	1	0
Foresters	Estuaries and Near Coastal Marine	Presence of the Environment	1	2
Foresters	Forests	Presence of the Environment	9	13
Foresters	Rivers and Streams	Presence of the Environment	1	0
Foresters	Terrestrial	Presence of the Environment	0	2

Foresters	Wetlands	Presence of the Environment	1	2
Fur_Hide Trappers and Hunters	Aquatic	Presence of the Environment	1	0
Fur_Hide Trappers and Hunters	Estuaries and Near Coastal Marine	Presence of the Environment	1	2
Fur_Hide Trappers and Hunters	Terrestrial	Presence of the Environment	0	2
Government and Municipal and Residential	Agroecosystems	Presence of the Environment	23	18
Government and Municipal and Residential	Aquatic	Presence of the Environment	27	29
Government and Municipal and Residential	Atmosphere	Presence of the Environment	12	7
Government and Municipal and Residential	Barren_Rock and Sand	Presence of the Environment	15	14
Government and Municipal and Residential	Created Greenspace	Presence of the Environment	27	27
Government and Municipal and Residential	Estuaries and Near Coastal Marine	Presence of the Environment	28	29
Government and Municipal and Residential	Forests	Presence of the Environment	18	16
Government and Municipal and Residential	Grasslands	Presence of the Environment	2	3
Government and Municipal and Residential	Groundwater	Presence of the Environment	12	4
Government and Municipal and Residential	Ice and Snow	Presence of the Environment	2	4
Government and Municipal and Residential	Lakes and Ponds	Presence of the Environment	14	9
Government and Municipal and Residential	Open Oceans and Seas	Presence of the Environment	17	19
Government and Municipal and Residential	Rivers and Streams	Presence of the Environment	25	23
Government and Municipal and Residential	Scrublands_Shrublands	Presence of the Environment	7	7
Government and Municipal and Residential	Terrestrial	Presence of the Environment	28	29
Government and Municipal and Residential	Wetlands	Presence of the Environment	26	28
Hunters	Agroecosystems	Presence of the Environment	1	1
Hunters	Aquatic	Presence of the Environment	5	14
Hunters	Created Greenspace	Presence of the Environment	0	7
Hunters	Estuaries and Near Coastal Marine	Presence of the Environment	7	8
Hunters	Forests	Presence of the Environment	0	1
Hunters	Rivers and Streams	Presence of the Environment	1	4
Hunters	Terrestrial	Presence of the Environment	0	7
Hunters	Wetlands	Presence of the Environment	0	1
Industrial Dischargers	Agroecosystems	Presence of the Environment	2	1
Industrial Dischargers	Aquatic	Presence of the Environment	9	0
Industrial Dischargers	Atmosphere	Presence of the Environment	1	0
Industrial Dischargers	Estuaries and Near Coastal Marine	Presence of the Environment	7	4
Industrial Dischargers	Groundwater	Presence of the Environment	4	3
Industrial Dischargers	Open Oceans and Seas	Presence of the Environment	1	1
Industrial Dischargers	Rivers and Streams	Presence of the Environment	3	0
Industrial Dischargers	Terrestrial	Presence of the Environment	8	6
Industrial Dischargers	Wetlands	Presence of the Environment	4	3
Industrial Processors	Agroecosystems	Presence of the Environment	0	1
Industrial Processors	Aquatic	Presence of the Environment	5	5
Industrial Processors	Barren_Rock and Sand	Presence of the Environment	1	0
Industrial Processors	Estuaries and Near Coastal Marine	Presence of the Environment	18	11
Industrial Processors	Groundwater	Presence of the Environment	2	0
Industrial Processors	Open Oceans and Seas	Presence of the Environment	2	2
Industrial Processors	Rivers and Streams	Presence of the Environment	7	1
Industrial Processors	Terrestrial	Presence of the Environment	7	7
Industrial Processors	Wetlands	Presence of the Environment	3	0
Inspirational	Aquatic	Presence of the Environment	4	9
Inspirational	Barren_Rock and Sand	Presence of the Environment	0	1
Inspirational	Created Greenspace	Presence of the Environment	2	10
Inspirational	Estuaries and Near Coastal Marine	Presence of the Environment	23	28
Inspirational	Forests	Presence of the Environment	0	2
Inspirational	Lakes and Ponds	Presence of the Environment	0	3
Inspirational	Open Oceans and Seas	Presence of the Environment	3	7
Inspirational	Rivers and Streams	Presence of the Environment	5	9
Inspirational	Terrestrial	Presence of the Environment	16	24
Inspirational	Wetlands	Presence of the Environment	1	6
Irrigators	Aquatic	Presence of the Environment	2	1
Irrigators	Estuaries and Near Coastal Marine	Presence of the Environment	2	1
Irrigators	Groundwater	Presence of the Environment	3	0
Irrigators	Rivers and Streams	Presence of the Environment	2	0
Irrigators	Terrestrial	Presence of the Environment	4	0
Irrigators	Wetlands	Presence of the Environment	3	1
Learning	Agroecosystems	Presence of the Environment	10	3
Learning	Aquatic	Presence of the Environment	21	25
Learning	Atmosphere	Presence of the Environment	1	1
Learning	Barren_Rock and Sand	Presence of the Environment	7	6
Learning	Created Greenspace	Presence of the Environment	12	29
Learning	Estuaries and Near Coastal Marine	Presence of the Environment	28	29
Learning	Forests	Presence of the Environment	6	12
Learning	Grasslands	Presence of the Environment	1	2
Learning	Groundwater	Presence of the Environment	2	0
Learning	Lakes and Ponds	Presence of the Environment	2	4
Learning	Open Oceans and Seas	Presence of the Environment	11	22
Learning	Rivers and Streams	Presence of the Environment	17	13
Learning	Scrublands_Shrublands	Presence of the Environment	0	3
Learning	Terrestrial	Presence of the Environment	24	29
Learning	Wetlands	Presence of the Environment	11	23
Livestock Grazers	Agroecosystems	Presence of the Environment	5	6

Livestock Grazers	Aquatic	Presence of the Environment	1	1
Livestock Grazers	Estuaries and Near Coastal Marine	Presence of the Environment	2	1
Livestock Grazers	Forests	Presence of the Environment	1	1
Livestock Grazers	Grasslands	Presence of the Environment	0	1
Livestock Grazers	Rivers and Streams	Presence of the Environment	2	0
Livestock Grazers	Scrublands_Shrublands	Presence of the Environment	0	1
Livestock Grazers	Terrestrial	Presence of the Environment	5	6
Livestock Grazers	Wetlands	Presence of the Environment	1	1
Military_Coast Guard	Aquatic	Presence of the Environment	2	3
Military_Coast Guard	Created Greenspace	Presence of the Environment	1	1
Military_Coast Guard	Estuaries and Near Coastal Marine	Presence of the Environment	20	16
Military_Coast Guard	Lakes and Ponds	Presence of the Environment	1	0
Military_Coast Guard	Open Oceans and Seas	Presence of the Environment	1	0
Military_Coast Guard	Rivers and Streams	Presence of the Environment	2	2
Military_Coast Guard	Terrestrial	Presence of the Environment	3	6
Military_Coast Guard	Wetlands	Presence of the Environment	3	4
Municipal Drinking Water Plant Operators	Aquatic	Presence of the Environment	8	8
Municipal Drinking Water Plant Operators	Estuaries and Near Coastal Marine	Presence of the Environment	4	4
Municipal Drinking Water Plant Operators	Forests	Presence of the Environment	1	1
Municipal Drinking Water Plant Operators	Groundwater	Presence of the Environment	4	2
Municipal Drinking Water Plant Operators	Rivers and Streams	Presence of the Environment	1	1
Municipal Drinking Water Plant Operators	Terrestrial	Presence of the Environment	5	0
Municipal Drinking Water Plant Operators	Wetlands	Presence of the Environment	6	4
Non-Use	Wetlands	Presence of the Environment	1	0
People Who Care (Existence)	Agroecosystems	Presence of the Environment	22	20
People Who Care (Existence)	Aquatic	Presence of the Environment	28	29
People Who Care (Existence)	Atmosphere	Presence of the Environment	4	3
People Who Care (Existence)	Barren_Rock and Sand	Presence of the Environment	15	13
People Who Care (Existence)	Created Greenspace	Presence of the Environment	15	23
People Who Care (Existence)	Estuaries and Near Coastal Marine	Presence of the Environment	28	29
People Who Care (Existence)	Forests	Presence of the Environment	22	26
People Who Care (Existence)	Grasslands	Presence of the Environment	4	6
People Who Care (Existence)	Groundwater	Presence of the Environment	5	1
People Who Care (Existence)	Ice and Snow	Presence of the Environment	2	3
People Who Care (Existence)	Lakes and Ponds	Presence of the Environment	15	14
People Who Care (Existence)	Open Oceans and Seas	Presence of the Environment	25	29
People Who Care (Existence)	Rivers and Streams	Presence of the Environment	27	25
People Who Care (Existence)	Scrublands_Shrublands	Presence of the Environment	6	11
People Who Care (Existence)	Terrestrial	Presence of the Environment	27	29
People Who Care (Existence)	Wetlands	Presence of the Environment	27	28
People Who Care (Option_Bequest)	Agroecosystems	Presence of the Environment	7	6
People Who Care (Option_Bequest)	Aquatic	Presence of the Environment	20	21
People Who Care (Option_Bequest)	Atmosphere	Presence of the Environment	2	1
People Who Care (Option_Bequest)	Barren_Rock and Sand	Presence of the Environment	4	2
People Who Care (Option_Bequest)	Created Greenspace	Presence of the Environment	6	17
People Who Care (Option_Bequest)	Estuaries and Near Coastal Marine	Presence of the Environment	28	29
People Who Care (Option_Bequest)	Forests	Presence of the Environment	8	9
People Who Care (Option_Bequest)	Grasslands	Presence of the Environment	0	3
People Who Care (Option_Bequest)	Groundwater	Presence of the Environment	2	0
People Who Care (Option_Bequest)	Lakes and Ponds	Presence of the Environment	3	4
People Who Care (Option_Bequest)	Open Oceans and Seas	Presence of the Environment	9	16
People Who Care (Option_Bequest)	Rivers and Streams	Presence of the Environment	18	14
People Who Care (Option_Bequest)	Scrublands_Shrublands	Presence of the Environment	1	5
People Who Care (Option_Bequest)	Terrestrial	Presence of the Environment	26	29
People Who Care (Option_Bequest)	Wetlands	Presence of the Environment	18	22
Recreational	Agroecosystems	Presence of the Environment	6	2
Recreational	Aquatic	Presence of the Environment	21	23
Recreational	Barren_Rock and Sand	Presence of the Environment	9	13
Recreational	Created Greenspace	Presence of the Environment	16	28
Recreational	Estuaries and Near Coastal Marine	Presence of the Environment	28	29
Recreational	Forests	Presence of the Environment	6	12
Recreational	Grasslands	Presence of the Environment	0	1
Recreational	Groundwater	Presence of the Environment	4	0
Recreational	Lakes and Ponds	Presence of the Environment	3	1
Recreational	Open Oceans and Seas	Presence of the Environment	12	18
Recreational	Rivers and Streams	Presence of the Environment	13	14
Recreational	Scrublands_Shrublands	Presence of the Environment	3	6
Recreational	Terrestrial	Presence of the Environment	24	29
Recreational	Wetlands	Presence of the Environment	13	24
Researchers	Agroecosystems	Presence of the Environment	2	3
Researchers	Aquatic	Presence of the Environment	14	28
Researchers	Barren_Rock and Sand	Presence of the Environment	1	1
Researchers	Created Greenspace	Presence of the Environment	1	11
Researchers	Estuaries and Near Coastal Marine	Presence of the Environment	26	29
Researchers	Forests	Presence of the Environment	1	5
Researchers	Grasslands	Presence of the Environment	0	1
Researchers	Groundwater	Presence of the Environment	1	1
Researchers	Lakes and Ponds	Presence of the Environment	1	2

Researchers	Open Oceans and Seas	Presence of the Environment	7	12
Researchers	Rivers and Streams	Presence of the Environment	6	12
Researchers	Scrublands_Shrublands	Presence of the Environment	0	4
Researchers	Terrestrial	Presence of the Environment	21	29
Researchers	Wetlands	Presence of the Environment	7	17
Residential Property Owners	Agroecosystems	Presence of the Environment	15	11
Residential Property Owners	Aquatic	Presence of the Environment	18	12
Residential Property Owners	Barren_Rock and Sand	Presence of the Environment	0	3
Residential Property Owners	Created Greenspace	Presence of the Environment	4	5
Residential Property Owners	Estuaries and Near Coastal Marine	Presence of the Environment	27	24
Residential Property Owners	Forests	Presence of the Environment	4	3
Residential Property Owners	Groundwater	Presence of the Environment	3	0
Residential Property Owners	Open Oceans and Seas	Presence of the Environment	1	2
Residential Property Owners	Rivers and Streams	Presence of the Environment	10	9
Residential Property Owners	Scrublands_Shrublands	Presence of the Environment	2	2
Residential Property Owners	Terrestrial	Presence of the Environment	28	28
Residential Property Owners	Wetlands	Presence of the Environment	8	13
Resource-Dependent Businesses	Agroecosystems	Presence of the Environment	4	0
Resource-Dependent Businesses	Aquatic	Presence of the Environment	25	20
Resource-Dependent Businesses	Barren_Rock and Sand	Presence of the Environment	8	6
Resource-Dependent Businesses	Created Greenspace	Presence of the Environment	10	15
Resource-Dependent Businesses	Estuaries and Near Coastal Marine	Presence of the Environment	27	29
Resource-Dependent Businesses	Forests	Presence of the Environment	2	2
Resource-Dependent Businesses	Lakes and Ponds	Presence of the Environment	1	2
Resource-Dependent Businesses	Open Oceans and Seas	Presence of the Environment	10	7
Resource-Dependent Businesses	Rivers and Streams	Presence of the Environment	8	6
Resource-Dependent Businesses	Terrestrial	Presence of the Environment	25	28
Resource-Dependent Businesses	Wetlands	Presence of the Environment	9	5
Spiritual and Ceremonial Participants and Participants of Celebration	Aquatic	Presence of the Environment	3	0
Spiritual and Ceremonial Participants and Participants of Celebration	Barren_Rock and Sand	Presence of the Environment	1	4
Spiritual and Ceremonial Participants and Participants of Celebration	Created Greenspace	Presence of the Environment	4	4
Spiritual and Ceremonial Participants and Participants of Celebration	Estuaries and Near Coastal Marine	Presence of the Environment	15	19
Spiritual and Ceremonial Participants and Participants of Celebration	Lakes and Ponds	Presence of the Environment	0	1
Spiritual and Ceremonial Participants and Participants of Celebration	Open Oceans and Seas	Presence of the Environment	1	2
Spiritual and Ceremonial Participants and Participants of Celebration	Rivers and Streams	Presence of the Environment	5	5
Spiritual and Ceremonial Participants and Participants of Celebration	Terrestrial	Presence of the Environment	4	6
Spiritual and Ceremonial Participants and Participants of Celebration	Wetlands	Presence of the Environment	1	3
Subsistence	Aquatic	Presence of the Environment	2	3
Subsistence	Estuaries and Near Coastal Marine	Presence of the Environment	6	17
Subsistence	Forests	Presence of the Environment	0	1
Subsistence	Open Oceans and Seas	Presence of the Environment	2	2
Subsistence	Rivers and Streams	Presence of the Environment	1	0
Subsistence	Terrestrial	Presence of the Environment	2	8
Subsistence	Wetlands	Presence of the Environment	0	1
Timber and Fiber and Fur_Hide Subsisters	Aquatic	Presence of the Environment	0	3
Timber and Fiber and Fur_Hide Subsisters	Rivers and Streams	Presence of the Environment	0	2
Timber and Fiber and Fur_Hide Subsisters	Terrestrial	Presence of the Environment	0	4
Timber and Fiber and Fur_Hide Subsisters	Wetlands	Presence of the Environment	0	1
Timber and Fiber and Ornamental Extractors	Agroecosystems	Presence of the Environment	1	2
Timber and Fiber and Ornamental Extractors	Aquatic	Presence of the Environment	3	0
Timber and Fiber and Ornamental Extractors	Created Greenspace	Presence of the Environment	1	2
Timber and Fiber and Ornamental Extractors	Estuaries and Near Coastal Marine	Presence of the Environment	6	9
Timber and Fiber and Ornamental Extractors	Forests	Presence of the Environment	2	5
Timber and Fiber and Ornamental Extractors	Scrublands_Shrublands	Presence of the Environment	1	2
Timber and Fiber and Ornamental Extractors	Terrestrial	Presence of the Environment	2	5
Timber and Fiber and Ornamental Extractors	Wetlands	Presence of the Environment	0	3
Transporters of Goods	Aquatic	Presence of the Environment	1	0
Transporters of Goods	Estuaries and Near Coastal Marine	Presence of the Environment	1	1
Transporters of Goods	Rivers and Streams	Presence of the Environment	1	1
Transporters of Goods	Terrestrial	Presence of the Environment	0	1
Transporters of People	Agroecosystems	Presence of the Environment	0	2
Transporters of People	Aquatic	Presence of the Environment	3	1
Transporters of People	Barren_Rock and Sand	Presence of the Environment	1	3
Transporters of People	Created Greenspace	Presence of the Environment	2	0
Transporters of People	Estuaries and Near Coastal Marine	Presence of the Environment	7	14
Transporters of People	Forests	Presence of the Environment	0	5
Transporters of People	Open Oceans and Seas	Presence of the Environment	1	2
Transporters of People	Rivers and Streams	Presence of the Environment	2	4
Transporters of People	Scrublands_Shrublands	Presence of the Environment	1	1
Transporters of People	Terrestrial	Presence of the Environment	1	10
Transporters of People	Wetlands	Presence of the Environment	2	1
Waders and Swimmers and Divers	Aquatic	Presence of the Environment	2	2
Waders and Swimmers and Divers	Barren_Rock and Sand	Presence of the Environment	1	0
Waders and Swimmers and Divers	Estuaries and Near Coastal Marine	Presence of the Environment	4	3
Wastewater Treatment Plant Operators	Aquatic	Presence of the Environment	11	4
Wastewater Treatment Plant Operators	Estuaries and Near Coastal Marine	Presence of the Environment	13	5
Wastewater Treatment Plant Operators	Forests	Presence of the Environment	1	0
Wastewater Treatment Plant Operators	Groundwater	Presence of the Environment	2	2

Wastewater Treatment Plant Operators	Lakes and Ponds	Presence of the Environment	3	0
Wastewater Treatment Plant Operators	Open Oceans and Seas	Presence of the Environment	1	0
Wastewater Treatment Plant Operators	Rivers and Streams	Presence of the Environment	10	0
Wastewater Treatment Plant Operators	Terrestrial	Presence of the Environment	3	1
Wastewater Treatment Plant Operators	Wetlands	Presence of the Environment	9	0
Water Subsisters	Aquatic	Presence of the Environment	3	3
Water Subsisters	Created Greenspace	Presence of the Environment	6	0
Water Subsisters	Estuaries and Near Coastal Marine	Presence of the Environment	0	2
Water Subsisters	Groundwater	Presence of the Environment	1	0
Water Subsisters	Rivers and Streams	Presence of the Environment	2	1
Water Subsisters	Terrestrial	Presence of the Environment	2	5
Water Subsisters	Wetlands	Presence of the Environment	0	1
Agricultural	Agroecosystems	Soil	2	2
Agricultural	Estuaries and Near Coastal Marine	Soil	0	1
Agricultural	Terrestrial	Soil	1	2
Farmers	Agroecosystems	Soil	4	3
Farmers	Aquatic	Soil	1	0
Farmers	Forests	Soil	0	1
Farmers	Rivers and Streams	Soil	0	1
Farmers	Terrestrial	Soil	2	1
Foresters	Terrestrial	Soil	0	1
Government and Municipal and Residential	Agroecosystems	Soil	1	0
Government and Municipal and Residential	Aquatic	Soil	1	0
Government and Municipal and Residential	Terrestrial	Soil	1	1
Industrial Dischargers	Aquatic	Soil	1	0
Industrial Dischargers	Wetlands	Soil	1	0
People Who Care (Existence)	Aquatic	Soil	1	2
People Who Care (Existence)	Open Oceans and Seas	Soil	1	0
People Who Care (Existence)	Rivers and Streams	Soil	0	1
People Who Care (Existence)	Terrestrial	Soil	2	2
People Who Care (Existence)	Wetlands	Soil	1	1
Experiencers and Viewers	Aquatic	Sounds and Scents	1	5
Experiencers and Viewers	Estuaries and Near Coastal Marine	Sounds and Scents	0	3
Experiencers and Viewers	Terrestrial	Sounds and Scents	0	2
Farmers	Agroecosystems	Sounds and Scents	1	0
Government and Municipal and Residential	Agroecosystems	Sounds and Scents	1	0
Government and Municipal and Residential	Aquatic	Sounds and Scents	2	0
Government and Municipal and Residential	Estuaries and Near Coastal Marine	Sounds and Scents	1	0
Commercial_Military Transportation	Barren_Rock and Sand	Substrate	0	1
Educators and Students	Aquatic	Substrate	0	2
Educators and Students	Estuaries and Near Coastal Marine	Substrate	0	3
Educators and Students	Wetlands	Substrate	0	2
Experiencers and Viewers	Open Oceans and Seas	Substrate	0	1
Experiencers and Viewers	Scrublands_Shrublands	Substrate	0	1
Food Extractors	Aquatic	Substrate	3	2
Food Extractors	Estuaries and Near Coastal Marine	Substrate	2	1
Food Pickers and Gatherers	Aquatic	Substrate	0	1
Food Pickers and Gatherers	Estuaries and Near Coastal Marine	Substrate	0	1
Government and Municipal and Residential	Aquatic	Substrate	1	1
Government and Municipal and Residential	Barren_Rock and Sand	Substrate	2	2
Government and Municipal and Residential	Estuaries and Near Coastal Marine	Substrate	4	3
Government and Municipal and Residential	Terrestrial	Substrate	0	1
Government and Municipal and Residential	Wetlands	Substrate	1	1
Learning	Barren_Rock and Sand	Substrate	0	1
Learning	Estuaries and Near Coastal Marine	Substrate	1	1
Learning	Wetlands	Substrate	1	0
People Who Care (Existence)	Barren_Rock and Sand	Substrate	2	0
People Who Care (Existence)	Estuaries and Near Coastal Marine	Substrate	7	3
People Who Care (Existence)	Open Oceans and Seas	Substrate	0	1
People Who Care (Existence)	Scrublands_Shrublands	Substrate	1	1
People Who Care (Existence)	Terrestrial	Substrate	1	1
People Who Care (Existence)	Wetlands	Substrate	1	0
People Who Care (Option_Bequest)	Estuaries and Near Coastal Marine	Substrate	2	0
People Who Care (Option_Bequest)	Open Oceans and Seas	Substrate	3	0
Agricultural Processors	Agroecosystems	Timber	1	2
Commercial_Military Transportation	Wetlands	Timber	0	1
Commerical_Industrial	Agroecosystems	Timber	1	0
Commerical_Industrial	Forests	Timber	3	4
Farmers	Agroecosystems	Timber	3	3
Foresters	Agroecosystems	Timber	2	3
Foresters	Forests	Timber	1	8
Foresters	Wetlands	Timber	0	1
Government and Municipal and Residential	Agroecosystems	Timber	1	2
Government and Municipal and Residential	Forests	Timber	1	5
Government and Municipal and Residential	Scrublands_Shrublands	Timber	0	3
Industrial Processors	Forests	Timber	5	2
Industrial Processors	Wetlands	Timber	1	0
Military_Coast Guard	Forests	Timber	0	1

People Who Care (Existence)	Forests	Timber	1	5
People Who Care (Option_Bequest)	Forests	Timber	0	4
Residential Property Owners	Forests	Timber	1	0
Residential Property Owners	Terrestrial	Timber	1	1
Resource-Dependent Businesses	Agroecosystems	Timber	2	0
Resource-Dependent Businesses	Forests	Timber	1	0
Timber and Fiber and Ornamental Extractors	Agroecosystems	Timber	2	6
Timber and Fiber and Ornamental Extractors	Aquatic	Timber	2	1
Timber and Fiber and Ornamental Extractors	Estuaries and Near Coastal Marine	Timber	3	6
Timber and Fiber and Ornamental Extractors	Forests	Timber	8	19
Timber and Fiber and Ornamental Extractors	Scrublands_Shrublands	Timber	1	0
Timber and Fiber and Ornamental Extractors	Terrestrial	Timber	6	13
Timber and Fiber and Ornamental Extractors	Wetlands	Timber	1	4
Transporters of Goods	Forests	Timber	2	0
Agricultural	Agroecosystems	Viewscapes	4	1
All Humans	Created Greenspace	Viewscapes	0	1
Boaters	Aquatic	Viewscapes	1	0
Boaters	Estuaries and Near Coastal Marine	Viewscapes	3	6
Commercial_Military Transportation	Barren_Rock and Sand	Viewscapes	0	1
Commercial_Military Transportation	Estuaries and Near Coastal Marine	Viewscapes	0	1
Commercial_Military Transportation	Rivers and Streams	Viewscapes	0	1
Experiencers and Viewers	Agroecosystems	Viewscapes	3	2
Experiencers and Viewers	Aquatic	Viewscapes	10	10
Experiencers and Viewers	Atmosphere	Viewscapes	6	2
Experiencers and Viewers	Barren_Rock and Sand	Viewscapes	3	6
Experiencers and Viewers	Created Greenspace	Viewscapes	10	19
Experiencers and Viewers	Estuaries and Near Coastal Marine	Viewscapes	22	24
Experiencers and Viewers	Forests	Viewscapes	2	4
Experiencers and Viewers	Lakes and Ponds	Viewscapes	0	3
Experiencers and Viewers	Open Oceans and Seas	Viewscapes	4	4
Experiencers and Viewers	Rivers and Streams	Viewscapes	9	12
Experiencers and Viewers	Scrublands_Shrublands	Viewscapes	1	1
Experiencers and Viewers	Terrestrial	Viewscapes	15	21
Experiencers and Viewers	Wetlands	Viewscapes	7	12
Farmers	Agroecosystems	Viewscapes	2	0
Government and Municipal and Residential	Agroecosystems	Viewscapes	1	0
Government and Municipal and Residential	Aquatic	Viewscapes	2	0
Government and Municipal and Residential	Barren_Rock and Sand	Viewscapes	0	1
Government and Municipal and Residential	Created Greenspace	Viewscapes	0	2
Government and Municipal and Residential	Estuaries and Near Coastal Marine	Viewscapes	5	3
Government and Municipal and Residential	Forests	Viewscapes	1	2
Government and Municipal and Residential	Lakes and Ponds	Viewscapes	1	0
Government and Municipal and Residential	Open Oceans and Seas	Viewscapes	0	1
Government and Municipal and Residential	Rivers and Streams	Viewscapes	2	1
Government and Municipal and Residential	Terrestrial	Viewscapes	5	2
Government and Municipal and Residential	Wetlands	Viewscapes	1	1
Inspirational	Agroecosystems	Viewscapes	0	1
Inspirational	Aquatic	Viewscapes	2	0
Inspirational	Barren_Rock and Sand	Viewscapes	0	1
Inspirational	Created Greenspace	Viewscapes	1	2
Inspirational	Estuaries and Near Coastal Marine	Viewscapes	13	3
Inspirational	Rivers and Streams	Viewscapes	2	0
Inspirational	Terrestrial	Viewscapes	8	1
Learning	Barren_Rock and Sand	Viewscapes	0	1
Learning	Created Greenspace	Viewscapes	0	4
Learning	Estuaries and Near Coastal Marine	Viewscapes	1	7
Learning	Rivers and Streams	Viewscapes	0	1
People Who Care (Existence)	Aquatic	Viewscapes	0	1
People Who Care (Existence)	Forests	Viewscapes	0	1
People Who Care (Existence)	Rivers and Streams	Viewscapes	1	1
People Who Care (Existence)	Terrestrial	Viewscapes	3	2
People Who Care (Existence)	Wetlands	Viewscapes	0	1
People Who Care (Option_Bequest)	Estuaries and Near Coastal Marine	Viewscapes	3	5
Recreational	Aquatic	Viewscapes	7	3
Recreational	Estuaries and Near Coastal Marine	Viewscapes	10	5
Recreational	Forests	Viewscapes	1	0
Recreational	Rivers and Streams	Viewscapes	2	3
Recreational	Scrublands_Shrublands	Viewscapes	0	1
Recreational	Terrestrial	Viewscapes	1	2
Recreational	Wetlands	Viewscapes	2	2
Residential Property Owners	Aquatic	Viewscapes	2	0
Residential Property Owners	Rivers and Streams	Viewscapes	2	2
Residential Property Owners	Terrestrial	Viewscapes	3	0
Resource-Dependent Businesses	Estuaries and Near Coastal Marine	Viewscapes	1	1
Resource-Dependent Businesses	Terrestrial	Viewscapes	1	0
Spiritual and Ceremonial Participants and Participants of Celebration	Terrestrial	Viewscapes	0	1
Agricultural	Agroecosystems	Water	18	14
Agricultural	Aquatic	Water	10	3

Agricultural	Estuaries and Near Coastal Marine	Water	6	5
Agricultural	Forests	Water	1	0
Agricultural	Groundwater	Water	2	1
Agricultural	Lakes and Ponds	Water	0	1
Agricultural	Rivers and Streams	Water	4	1
Agricultural	Terrestrial	Water	0	1
Agricultural	Wetlands	Water	1	2
Agricultural Processors	Aquatic	Water	2	2
All Humans	Aquatic	Water	19	12
All Humans	Barren_Rock and Sand	Water	2	1
All Humans	Estuaries and Near Coastal Marine	Water	6	1
All Humans	Groundwater	Water	2	1
All Humans	Lakes and Ponds	Water	0	1
All Humans	Open Oceans and Seas	Water	1	0
All Humans	Rivers and Streams	Water	4	1
All Humans	Terrestrial	Water	1	0
All Humans	Wetlands	Water	2	1
Anglers	Aquatic	Water	9	8
Anglers	Estuaries and Near Coastal Marine	Water	7	1
Aquaculturists	Aquatic	Water	1	2
Aquaculturists	Estuaries and Near Coastal Marine	Water	3	0
Aquaculturists	Open Oceans and Seas	Water	6	2
Artists	Aquatic	Water	2	5
Boaters	Aquatic	Water	27	29
Boaters	Barren_Rock and Sand	Water	7	6
Boaters	Created Greenspace	Water	8	12
Boaters	Estuaries and Near Coastal Marine	Water	26	29
Boaters	Forests	Water	2	4
Boaters	Lakes and Ponds	Water	4	6
Boaters	Open Oceans and Seas	Water	14	13
Boaters	Rivers and Streams	Water	20	26
Boaters	Terrestrial	Water	17	22
Boaters	Wetlands	Water	10	14
CAFO Operators	Agroecosystems	Water	2	1
CAFO Operators	Aquatic	Water	5	0
CAFO Operators	Estuaries and Near Coastal Marine	Water	3	0
CAFO Operators	Rivers and Streams	Water	4	2
CAFO Operators	Wetlands	Water	1	2
Commercial_Military Transportation	Agroecosystems	Water	1	0
Commercial_Military Transportation	Aquatic	Water	21	22
Commercial_Military Transportation	Barren_Rock and Sand	Water	1	2
Commercial_Military Transportation	Created Greenspace	Water	0	1
Commercial_Military Transportation	Estuaries and Near Coastal Marine	Water	18	15
Commercial_Military Transportation	Forests	Water	1	1
Commercial_Military Transportation	Lakes and Ponds	Water	1	2
Commercial_Military Transportation	Open Oceans and Seas	Water	5	6
Commercial_Military Transportation	Rivers and Streams	Water	16	17
Commercial_Military Transportation	Terrestrial	Water	11	12
Commercial_Military Transportation	Wetlands	Water	6	4
Commerical_Industrial	Agroecosystems	Water	1	1
Commerical_Industrial	Aquatic	Water	23	14
Commerical_Industrial	Created Greenspace	Water	0	1
Commerical_Industrial	Estuaries and Near Coastal Marine	Water	13	8
Commerical_Industrial	Groundwater	Water	5	2
Commerical_Industrial	Lakes and Ponds	Water	0	3
Commerical_Industrial	Open Oceans and Seas	Water	4	1
Commerical_Industrial	Rivers and Streams	Water	10	4
Commerical_Industrial	Terrestrial	Water	5	0
Commerical_Industrial	Wetlands	Water	1	0
Educators and Students	Aquatic	Water	27	28
Educators and Students	Barren_Rock and Sand	Water	1	0
Educators and Students	Created Greenspace	Water	3	1
Educators and Students	Estuaries and Near Coastal Marine	Water	12	15
Educators and Students	Lakes and Ponds	Water	1	1
Educators and Students	Open Oceans and Seas	Water	3	0
Educators and Students	Rivers and Streams	Water	5	1
Educators and Students	Terrestrial	Water	3	1
Educators and Students	Wetlands	Water	2	2
Electric and other Energy Generators	Aquatic	Water	15	7
Electric and other Energy Generators	Estuaries and Near Coastal Marine	Water	6	5
Electric and other Energy Generators	Groundwater	Water	1	0
Electric and other Energy Generators	Lakes and Ponds	Water	3	4
Electric and other Energy Generators	Open Oceans and Seas	Water	2	1
Electric and other Energy Generators	Rivers and Streams	Water	6	2
Electric and other Energy Generators	Terrestrial	Water	5	2
Experiencers and Viewers	Aquatic	Water	20	16
Experiencers and Viewers	Barren_Rock and Sand	Water	5	5
Experiencers and Viewers	Created Greenspace	Water	1	8

Experiencers and Viewers	Estuaries and Near Coastal Marine	Water	15	17
Experiencers and Viewers	Groundwater	Water	1	0
Experiencers and Viewers	Open Oceans and Seas	Water	2	4
Experiencers and Viewers	Rivers and Streams	Water	3	5
Experiencers and Viewers	Terrestrial	Water	0	6
Experiencers and Viewers	Wetlands	Water	0	1
Farmers	Agroecosystems	Water	11	8
Farmers	Aquatic	Water	5	1
Farmers	Created Greenspace	Water	1	0
Farmers	Estuaries and Near Coastal Marine	Water	3	1
Farmers	Groundwater	Water	1	0
Farmers	Lakes and Ponds	Water	1	0
Farmers	Rivers and Streams	Water	3	2
Farmers	Terrestrial	Water	5	3
Farmers	Wetlands	Water	2	3
Food Extractors	Aquatic	Water	8	1
Food Extractors	Wetlands	Water	1	0
Food Pickers and Gatherers	Aquatic	Water	1	0
Food Pickers and Gatherers	Wetlands	Water	1	0
Government and Municipal and Residential	Agroecosystems	Water	9	0
Government and Municipal and Residential	Aquatic	Water	28	29
Government and Municipal and Residential	Barren_Rock and Sand	Water	9	4
Government and Municipal and Residential	Created Greenspace	Water	12	9
Government and Municipal and Residential	Estuaries and Near Coastal Marine	Water	27	29
Government and Municipal and Residential	Forests	Water	7	3
Government and Municipal and Residential	Groundwater	Water	20	13
Government and Municipal and Residential	Ice and Snow	Water	1	0
Government and Municipal and Residential	Lakes and Ponds	Water	9	6
Government and Municipal and Residential	Open Oceans and Seas	Water	10	7
Government and Municipal and Residential	Rivers and Streams	Water	24	21
Government and Municipal and Residential	Terrestrial	Water	19	20
Government and Municipal and Residential	Wetlands	Water	13	13
Industrial Dischargers	Agroecosystems	Water	2	0
Industrial Dischargers	Aquatic	Water	21	11
Industrial Dischargers	Estuaries and Near Coastal Marine	Water	17	10
Industrial Dischargers	Groundwater	Water	3	1
Industrial Dischargers	Lakes and Ponds	Water	2	1
Industrial Dischargers	Open Oceans and Seas	Water	3	1
Industrial Dischargers	Rivers and Streams	Water	16	3
Industrial Dischargers	Terrestrial	Water	7	3
Industrial Dischargers	Wetlands	Water	5	1
Industrial Processors	Aquatic	Water	11	9
Industrial Processors	Estuaries and Near Coastal Marine	Water	5	4
Industrial Processors	Forests	Water	1	0
Industrial Processors	Groundwater	Water	3	2
Industrial Processors	Open Oceans and Seas	Water	1	0
Industrial Processors	Rivers and Streams	Water	7	0
Irrigators	Agroecosystems	Water	12	3
Irrigators	Aquatic	Water	17	8
Irrigators	Created Greenspace	Water	4	0
Irrigators	Estuaries and Near Coastal Marine	Water	10	4
Irrigators	Groundwater	Water	7	0
Irrigators	Ice and Snow	Water	2	0
Irrigators	Lakes and Ponds	Water	3	3
Irrigators	Rivers and Streams	Water	11	2
Irrigators	Terrestrial	Water	9	2
Irrigators	Wetlands	Water	2	1
Learning	Aquatic	Water	21	22
Learning	Estuaries and Near Coastal Marine	Water	8	13
Learning	Groundwater	Water	2	0
Learning	Open Oceans and Seas	Water	1	0
Learning	Rivers and Streams	Water	4	2
Learning	Terrestrial	Water	5	1
Livestock Grazers	Agroecosystems	Water	4	1
Livestock Grazers	Aquatic	Water	1	0
Livestock Grazers	Created Greenspace	Water	1	0
Livestock Grazers	Rivers and Streams	Water	2	0
Livestock Grazers	Terrestrial	Water	2	0
Livestock Grazers	Wetlands	Water	1	1
Military_Coast Guard	Aquatic	Water	7	4
Military_Coast Guard	Estuaries and Near Coastal Marine	Water	11	6
Military_Coast Guard	Lakes and Ponds	Water	2	1
Military_Coast Guard	Open Oceans and Seas	Water	2	1
Military_Coast Guard	Rivers and Streams	Water	1	2
Military_Coast Guard	Wetlands	Water	2	2
Municipal Drinking Water Plant Operators	Aquatic	Water	24	20
Municipal Drinking Water Plant Operators	Barren_Rock and Sand	Water	1	1
Municipal Drinking Water Plant Operators	Created Greenspace	Water	2	1

Municipal Drinking Water Plant Operators	Estuaries and Near Coastal Marine	Water	16	9
Municipal Drinking Water Plant Operators	Forests	Water	2	1
Municipal Drinking Water Plant Operators	Groundwater	Water	13	4
Municipal Drinking Water Plant Operators	Ice and Snow	Water	1	0
Municipal Drinking Water Plant Operators	Lakes and Ponds	Water	3	2
Municipal Drinking Water Plant Operators	Open Oceans and Seas	Water	3	2
Municipal Drinking Water Plant Operators	Rivers and Streams	Water	14	6
Municipal Drinking Water Plant Operators	Terrestrial	Water	13	5
Municipal Drinking Water Plant Operators	Wetlands	Water	5	3
People Who Care (Existence)	Agroecosystems	Water	11	3
People Who Care (Existence)	Aquatic	Water	28	29
People Who Care (Existence)	Created Greenspace	Water	1	2
People Who Care (Existence)	Estuaries and Near Coastal Marine	Water	28	29
People Who Care (Existence)	Forests	Water	2	0
People Who Care (Existence)	Groundwater	Water	15	10
People Who Care (Existence)	Lakes and Ponds	Water	6	4
People Who Care (Existence)	Open Oceans and Seas	Water	11	1
People Who Care (Existence)	Rivers and Streams	Water	18	14
People Who Care (Existence)	Scrublands_Shrublands	Water	0	2
People Who Care (Existence)	Terrestrial	Water	19	16
People Who Care (Existence)	Wetlands	Water	14	19
People Who Care (Option_Bequest)	Aquatic	Water	14	17
People Who Care (Option_Bequest)	Created Greenspace	Water	0	1
People Who Care (Option_Bequest)	Estuaries and Near Coastal Marine	Water	16	12
People Who Care (Option_Bequest)	Groundwater	Water	5	0
People Who Care (Option_Bequest)	Rivers and Streams	Water	3	2
People Who Care (Option_Bequest)	Terrestrial	Water	2	1
People Who Care (Option_Bequest)	Wetlands	Water	3	1
Recreational	Aquatic	Water	26	28
Recreational	Barren_Rock and Sand	Water	1	0
Recreational	Created Greenspace	Water	7	3
Recreational	Estuaries and Near Coastal Marine	Water	19	12
Recreational	Forests	Water	1	0
Recreational	Groundwater	Water	1	0
Recreational	Open Oceans and Seas	Water	0	3
Recreational	Rivers and Streams	Water	6	9
Recreational	Terrestrial	Water	3	3
Recreational	Wetlands	Water	3	2
Researchers	Aquatic	Water	7	16
Researchers	Created Greenspace	Water	1	0
Researchers	Estuaries and Near Coastal Marine	Water	10	22
Researchers	Open Oceans and Seas	Water	2	0
Researchers	Rivers and Streams	Water	2	2
Researchers	Terrestrial	Water	3	1
Residential Property Owners	Agroecosystems	Water	2	0
Residential Property Owners	Aquatic	Water	21	15
Residential Property Owners	Created Greenspace	Water	1	3
Residential Property Owners	Estuaries and Near Coastal Marine	Water	11	3
Residential Property Owners	Groundwater	Water	4	0
Residential Property Owners	Rivers and Streams	Water	4	2
Residential Property Owners	Terrestrial	Water	5	4
Residential Property Owners	Wetlands	Water	1	1
Resource-Dependent Businesses	Aquatic	Water	13	11
Resource-Dependent Businesses	Created Greenspace	Water	1	1
Resource-Dependent Businesses	Estuaries and Near Coastal Marine	Water	14	5
Resource-Dependent Businesses	Groundwater	Water	2	0
Resource-Dependent Businesses	Lakes and Ponds	Water	3	1
Resource-Dependent Businesses	Rivers and Streams	Water	1	1
Resource-Dependent Businesses	Terrestrial	Water	3	2
Spiritual and Ceremonial Participants and Participants of Celebration	Aquatic	Water	2	0
Spiritual and Ceremonial Participants and Participants of Celebration	Estuaries and Near Coastal Marine	Water	1	2
Subsistence	Aquatic	Water	1	0
Subsistence	Estuaries and Near Coastal Marine	Water	1	0
Subsistence	Wetlands	Water	0	1
Transporters of Goods	Aquatic	Water	12	9
Transporters of Goods	Created Greenspace	Water	1	2
Transporters of Goods	Estuaries and Near Coastal Marine	Water	10	12
Transporters of Goods	Lakes and Ponds	Water	0	1
Transporters of Goods	Open Oceans and Seas	Water	2	1
Transporters of Goods	Rivers and Streams	Water	6	7
Transporters of Goods	Terrestrial	Water	4	3
Transporters of Goods	Wetlands	Water	1	1
Transporters of People	Aquatic	Water	11	11
Transporters of People	Estuaries and Near Coastal Marine	Water	2	4
Transporters of People	Rivers and Streams	Water	3	8
Transporters of People	Terrestrial	Water	2	3
Transporters of People	Wetlands	Water	1	2
Waders and Swimmers and Divers	Aquatic	Water	22	23

Waders and Swimmers and Divers	Barren_Rock and Sand	Water	11	7
Waders and Swimmers and Divers	Created Greenspace	Water	1	2
Waders and Swimmers and Divers	Estuaries and Near Coastal Marine	Water	19	16
Waders and Swimmers and Divers	Forests	Water	3	2
Waders and Swimmers and Divers	Groundwater	Water	2	0
Waders and Swimmers and Divers	Lakes and Ponds	Water	3	1
Waders and Swimmers and Divers	Open Oceans and Seas	Water	7	5
Waders and Swimmers and Divers	Rivers and Streams	Water	11	5
Waders and Swimmers and Divers	Terrestrial	Water	6	9
Waders and Swimmers and Divers	Wetlands	Water	2	5
Wastewater Treatment Plant Operators	Aquatic	Water	17	6
Wastewater Treatment Plant Operators	Estuaries and Near Coastal Marine	Water	7	3
Wastewater Treatment Plant Operators	Groundwater	Water	3	1
Wastewater Treatment Plant Operators	Open Oceans and Seas	Water	2	0
Wastewater Treatment Plant Operators	Rivers and Streams	Water	8	1
Wastewater Treatment Plant Operators	Terrestrial	Water	1	0
Wastewater Treatment Plant Operators	Wetlands	Water	3	0
Water Subsisters	Aquatic	Water	8	8
Water Subsisters	Created Greenspace	Water	8	1
Water Subsisters	Estuaries and Near Coastal Marine	Water	1	0
Water Subsisters	Groundwater	Water	1	0
Water Subsisters	Rivers and Streams	Water	2	2
Water Subsisters	Terrestrial	Water	1	4
Water Subsisters	Wetlands	Water	0	1
Agricultural	Estuaries and Near Coastal Marine	Weather	0	1
Anglers	Aquatic	Weather	4	3
Anglers	Ice and Snow	Weather	0	1
Boaters	Aquatic	Weather	3	2
Boaters	Estuaries and Near Coastal Marine	Weather	0	1
Experiencers and Viewers	Aquatic	Weather	1	3
Experiencers and Viewers	Estuaries and Near Coastal Marine	Weather	3	5
Experiencers and Viewers	Scrublands_Shrublands	Weather	0	2
Experiencers and Viewers	Terrestrial	Weather	1	1
Experiencers and Viewers	Wetlands	Weather	0	1
Farmers	Aquatic	Weather	1	0
Farmers	Lakes and Ponds	Weather	0	1
Farmers	Terrestrial	Weather	0	1
Government and Municipal and Residential	Aquatic	Weather	0	2
Government and Municipal and Residential	Estuaries and Near Coastal Marine	Weather	1	0
Government and Municipal and Residential	Rivers and Streams	Weather	1	0
Government and Municipal and Residential	Terrestrial	Weather	0	1
People Who Care (Existence)	Estuaries and Near Coastal Marine	Weather	2	0
People Who Care (Existence)	Forests	Weather	0	1
People Who Care (Existence)	Scrublands_Shrublands	Weather	0	1
Recreational	Aquatic	Weather	4	3
Recreational	Estuaries and Near Coastal Marine	Weather	3	4
Recreational	Wetlands	Weather	0	1
Boaters	Aquatic	Wind	5	6
Boaters	Estuaries and Near Coastal Marine	Wind	3	4
Boaters	Rivers and Streams	Wind	1	0
Electric and other Energy Generators	Estuaries and Near Coastal Marine	Wind	1	2
Electric and other Energy Generators	Open Oceans and Seas	Wind	1	0
Government and Municipal and Residential	Estuaries and Near Coastal Marine	Wind	2	0
Government and Municipal and Residential	Open Oceans and Seas	Wind	1	0
People Who Care (Option_Bequest)	Estuaries and Near Coastal Marine	Wind	1	0
Agricultural	Agroecosystems	FEGS	21	20
Agricultural	Terrestrial	FEGS	1	0
Agricultural Processors	Agroecosystems	FEGS	4	2
Agricultural Processors	Aquatic	FEGS	0	1
Agricultural Processors	Estuaries and Near Coastal Marine	FEGS	0	2
Agricultural Processors	Rivers and Streams	FEGS	1	0
All Humans	Agroecosystems	FEGS	2	0
All Humans	Aquatic	FEGS	9	0
All Humans	Created Greenspace	FEGS	0	2
All Humans	Estuaries and Near Coastal Marine	FEGS	11	7
All Humans	Lakes and Ponds	FEGS	0	1
All Humans	Open Oceans and Seas	FEGS	2	0
All Humans	Rivers and Streams	FEGS	4	1
All Humans	Terrestrial	FEGS	2	0
All Humans	Wetlands	FEGS	1	1
Anglers	Aquatic	FEGS	1	0
Anglers	Estuaries and Near Coastal Marine	FEGS	5	0
Aquaculturists	Aquatic	FEGS	1	1
Aquaculturists	Open Oceans and Seas	FEGS	1	1
Artists	Rivers and Streams	FEGS	1	0
Boaters	Aquatic	FEGS	1	2
Boaters	Estuaries and Near Coastal Marine	FEGS	5	1
Boaters	Terrestrial	FEGS	1	1

CAFO Operators	Agroecosystems	FEGS	1	0
CAFO Operators	Estuaries and Near Coastal Marine	FEGS	1	0
CAFO Operators	Rivers and Streams	FEGS	1	0
CAFO Operators	Terrestrial	FEGS	1	0
Commercial_Military Transportation	Aquatic	FEGS	2	1
Commercial_Military Transportation	Estuaries and Near Coastal Marine	FEGS	1	7
Commercial_Military Transportation	Terrestrial	FEGS	2	1
Commercial_Military Transportation	Wetlands	FEGS	0	1
Commerical_Industrial	Agroecosystems	FEGS	4	0
Commerical_Industrial	Aquatic	FEGS	17	7
Commerical_Industrial	Barren_Rock and Sand	FEGS	0	1
Commerical_Industrial	Created Greenspace	FEGS	0	1
Commerical_Industrial	Estuaries and Near Coastal Marine	FEGS	17	12
Commerical_Industrial	Forests	FEGS	0	1
Commerical_Industrial	Lakes and Ponds	FEGS	0	1
Commerical_Industrial	Open Oceans and Seas	FEGS	1	4
Commerical_Industrial	Rivers and Streams	FEGS	1	0
Commerical_Industrial	Terrestrial	FEGS	3	2
Commerical_Industrial	Wetlands	FEGS	1	3
Educators and Students	Agroecosystems	FEGS	0	2
Educators and Students	Aquatic	FEGS	4	7
Educators and Students	Barren_Rock and Sand	FEGS	1	0
Educators and Students	Created Greenspace	FEGS	1	1
Educators and Students	Estuaries and Near Coastal Marine	FEGS	27	29
Educators and Students	Forests	FEGS	0	2
Educators and Students	Open Oceans and Seas	FEGS	5	5
Educators and Students	Rivers and Streams	FEGS	1	2
Educators and Students	Terrestrial	FEGS	1	9
Educators and Students	Wetlands	FEGS	0	4
Electric and other Energy Generators	Aquatic	FEGS	1	2
Electric and other Energy Generators	Estuaries and Near Coastal Marine	FEGS	3	2
Electric and other Energy Generators	Lakes and Ponds	FEGS	0	1
Electric and other Energy Generators	Wetlands	FEGS	0	1
Experiencers and Viewers	Aquatic	FEGS	4	5
Experiencers and Viewers	Barren_Rock and Sand	FEGS	0	1
Experiencers and Viewers	Created Greenspace	FEGS	0	8
Experiencers and Viewers	Estuaries and Near Coastal Marine	FEGS	15	12
Experiencers and Viewers	Forests	FEGS	1	0
Experiencers and Viewers	Open Oceans and Seas	FEGS	1	1
Experiencers and Viewers	Rivers and Streams	FEGS	0	1
Experiencers and Viewers	Terrestrial	FEGS	2	3
Experiencers and Viewers	Wetlands	FEGS	1	1
Farmers	Agroecosystems	FEGS	11	7
Farmers	Terrestrial	FEGS	2	0
Farmers	Wetlands	FEGS	1	0
Food Extractors	Aquatic	FEGS	9	6
Food Extractors	Estuaries and Near Coastal Marine	FEGS	9	5
Food Extractors	Wetlands	FEGS	1	0
Food Pickers and Gatherers	Aquatic	FEGS	0	1
Food Pickers and Gatherers	Estuaries and Near Coastal Marine	FEGS	1	1
Food Pickers and Gatherers	Open Oceans and Seas	FEGS	2	0
Food Pickers and Gatherers	Rivers and Streams	FEGS	0	1
Food Pickers and Gatherers	Terrestrial	FEGS	1	2
Food Subsisters	Aquatic	FEGS	1	1
Food Subsisters	Wetlands	FEGS	0	1
Foresters	Agroecosystems	FEGS	0	3
Foresters	Forests	FEGS	3	9
Fur_Hide Trappers and Hunters	Estuaries and Near Coastal Marine	FEGS	0	2
Fur_Hide Trappers and Hunters	Terrestrial	FEGS	0	2
Government and Municipal and Residential	Agroecosystems	FEGS	8	4
Government and Municipal and Residential	Aquatic	FEGS	28	29
Government and Municipal and Residential	Barren_Rock and Sand	FEGS	4	5
Government and Municipal and Residential	Created Greenspace	FEGS	11	12
Government and Municipal and Residential	Estuaries and Near Coastal Marine	FEGS	28	29
Government and Municipal and Residential	Forests	FEGS	4	4
Government and Municipal and Residential	Ice and Snow	FEGS	0	1
Government and Municipal and Residential	Lakes and Ponds	FEGS	2	1
Government and Municipal and Residential	Open Oceans and Seas	FEGS	16	15
Government and Municipal and Residential	Rivers and Streams	FEGS	12	7
Government and Municipal and Residential	Scrublands_Shrublands	FEGS	0	1
Government and Municipal and Residential	Terrestrial	FEGS	27	29
Government and Municipal and Residential	Wetlands	FEGS	10	12
Hunters	Aquatic	FEGS	0	1
Hunters	Estuaries and Near Coastal Marine	FEGS	2	0
Industrial Dischargers	Open Oceans and Seas	FEGS	0	1
Industrial Dischargers	Terrestrial	FEGS	2	2
Industrial Dischargers	Wetlands	FEGS	0	1
Industrial Processors	Agroecosystems	FEGS	1	0

Industrial Processors	Aquatic	FEGS	2	1
Industrial Processors	Estuaries and Near Coastal Marine	FEGS	2	5
Industrial Processors	Forests	FEGS	0	1
Industrial Processors	Lakes and Ponds	FEGS	0	1
Industrial Processors	Rivers and Streams	FEGS	2	0
Industrial Processors	Terrestrial	FEGS	3	0
Inspirational	Aquatic	FEGS	0	1
Inspirational	Created Greenspace	FEGS	0	1
Inspirational	Estuaries and Near Coastal Marine	FEGS	6	7
Inspirational	Forests	FEGS	0	5
Inspirational	Open Oceans and Seas	FEGS	2	1
Inspirational	Rivers and Streams	FEGS	0	2
Inspirational	Terrestrial	FEGS	1	5
Inspirational	Wetlands	FEGS	0	2
Irrigators	Rivers and Streams	FEGS	1	0
Learning	Agroecosystems	FEGS	0	1
Learning	Aquatic	FEGS	2	4
Learning	Barren_Rock and Sand	FEGS	1	0
Learning	Created Greenspace	FEGS	0	2
Learning	Estuaries and Near Coastal Marine	FEGS	12	22
Learning	Forests	FEGS	0	1
Learning	Groundwater	FEGS	1	0
Learning	Open Oceans and Seas	FEGS	4	5
Learning	Rivers and Streams	FEGS	3	1
Learning	Terrestrial	FEGS	0	5
Learning	Wetlands	FEGS	1	2
Livestock Grazers	Agroecosystems	FEGS	2	2
Livestock Grazers	Aquatic	FEGS	0	1
Livestock Grazers	Estuaries and Near Coastal Marine	FEGS	2	0
Livestock Grazers	Terrestrial	FEGS	3	0
Livestock Grazers	Wetlands	FEGS	0	1
Military_Coast Guard	Estuaries and Near Coastal Marine	FEGS	3	2
Municipal Drinking Water Plant Operators	Aquatic	FEGS	3	5
Municipal Drinking Water Plant Operators	Forests	FEGS	0	1
Municipal Drinking Water Plant Operators	Rivers and Streams	FEGS	1	0
Municipal Drinking Water Plant Operators	Wetlands	FEGS	1	0
Non-Use	Wetlands	FEGS	1	0
People Who Care (Existence)	Agroecosystems	FEGS	5	5
People Who Care (Existence)	Aquatic	FEGS	28	29
People Who Care (Existence)	Barren_Rock and Sand	FEGS	1	1
People Who Care (Existence)	Created Greenspace	FEGS	15	13
People Who Care (Existence)	Estuaries and Near Coastal Marine	FEGS	28	29
People Who Care (Existence)	Forests	FEGS	8	14
People Who Care (Existence)	Grasslands	FEGS	0	1
People Who Care (Existence)	Groundwater	FEGS	9	4
People Who Care (Existence)	Lakes and Ponds	FEGS	5	7
People Who Care (Existence)	Open Oceans and Seas	FEGS	16	22
People Who Care (Existence)	Rivers and Streams	FEGS	26	24
People Who Care (Existence)	Scrublands_Shrublands	FEGS	0	2
People Who Care (Existence)	Terrestrial	FEGS	27	29
People Who Care (Existence)	Wetlands	FEGS	27	28
People Who Care (Option_Bequest)	Agroecosystems	FEGS	1	0
People Who Care (Option_Bequest)	Aquatic	FEGS	16	15
People Who Care (Option_Bequest)	Barren_Rock and Sand	FEGS	1	1
People Who Care (Option_Bequest)	Created Greenspace	FEGS	1	4
People Who Care (Option_Bequest)	Estuaries and Near Coastal Marine	FEGS	28	29
People Who Care (Option_Bequest)	Forests	FEGS	2	8
People Who Care (Option_Bequest)	Grasslands	FEGS	0	3
People Who Care (Option_Bequest)	Groundwater	FEGS	2	0
People Who Care (Option_Bequest)	Lakes and Ponds	FEGS	0	1
People Who Care (Option_Bequest)	Open Oceans and Seas	FEGS	4	4
People Who Care (Option_Bequest)	Rivers and Streams	FEGS	3	5
People Who Care (Option_Bequest)	Terrestrial	FEGS	20	29
People Who Care (Option_Bequest)	Wetlands	FEGS	4	10
Recreational	Agroecosystems	FEGS	4	3
Recreational	Aquatic	FEGS	24	27
Recreational	Barren_Rock and Sand	FEGS	7	4
Recreational	Created Greenspace	FEGS	12	10
Recreational	Estuaries and Near Coastal Marine	FEGS	28	29
Recreational	Forests	FEGS	4	1
Recreational	Groundwater	FEGS	2	0
Recreational	Lakes and Ponds	FEGS	1	0
Recreational	Open Oceans and Seas	FEGS	13	18
Recreational	Rivers and Streams	FEGS	11	7
Recreational	Scrublands_Shrublands	FEGS	3	5
Recreational	Terrestrial	FEGS	20	26
Recreational	Wetlands	FEGS	12	20
Researchers	Aquatic	FEGS	3	5

Researchers	Created Greenspace	FEGS	0	1
Researchers	Estuaries and Near Coastal Marine	FEGS	10	24
Researchers	Open Oceans and Seas	FEGS	2	1
Researchers	Rivers and Streams	FEGS	0	1
Researchers	Terrestrial	FEGS	7	10
Researchers	Wetlands	FEGS	1	1
Residential Property Owners	Agroecosystems	FEGS	3	4
Residential Property Owners	Aquatic	FEGS	3	2
Residential Property Owners	Estuaries and Near Coastal Marine	FEGS	9	3
Residential Property Owners	Open Oceans and Seas	FEGS	0	1
Residential Property Owners	Rivers and Streams	FEGS	1	0
Residential Property Owners	Terrestrial	FEGS	14	18
Residential Property Owners	Wetlands	FEGS	1	2
Resource-Dependent Businesses	Agroecosystems	FEGS	2	1
Resource-Dependent Businesses	Aquatic	FEGS	8	3
Resource-Dependent Businesses	Created Greenspace	FEGS	0	1
Resource-Dependent Businesses	Estuaries and Near Coastal Marine	FEGS	14	7
Resource-Dependent Businesses	Forests	FEGS	0	1
Resource-Dependent Businesses	Lakes and Ponds	FEGS	0	1
Resource-Dependent Businesses	Open Oceans and Seas	FEGS	2	1
Resource-Dependent Businesses	Rivers and Streams	FEGS	1	0
Resource-Dependent Businesses	Terrestrial	FEGS	7	5
Resource-Dependent Businesses	Wetlands	FEGS	0	2
Spiritual and Ceremonial Participants and Participants of Celebration	Estuaries and Near Coastal Marine	FEGS	0	1
Subsistence	Aquatic	FEGS	1	2
Subsistence	Estuaries and Near Coastal Marine	FEGS	1	1
Subsistence	Forests	FEGS	0	1
Subsistence	Open Oceans and Seas	FEGS	1	2
Subsistence	Rivers and Streams	FEGS	1	1
Subsistence	Terrestrial	FEGS	0	1
Subsistence	Wetlands	FEGS	0	2
Timber and Fiber and Ornamental Extractors	Aquatic	FEGS	4	1
Timber and Fiber and Ornamental Extractors	Estuaries and Near Coastal Marine	FEGS	1	0
Timber and Fiber and Ornamental Extractors	Terrestrial	FEGS	2	0
Transporters of Goods	Estuaries and Near Coastal Marine	FEGS	0	1
Transporters of Goods	Terrestrial	FEGS	1	0
Wastewater Treatment Plant Operators	Aquatic	FEGS	1	0
Water Subsisters	Aquatic	FEGS	1	1
Water Subsisters	Rivers and Streams	FEGS	0	1
People Who Care (Existence)	Environment	Air	1	0
Experiencers and Viewers	Environment	Atmospheric Phenomena	1	4
All Humans	Environment	Depredators and (Pest) Predators	1	0
Commercial_Military Transportation	Environment	Depredators and (Pest) Predators	0	1
Commerical_Industrial	Environment	Depredators and (Pest) Predators	1	0
Educators and Students	Environment	Depredators and (Pest) Predators	2	0
Government and Municipal and Residential	Environment	Depredators and (Pest) Predators	3	0
Learning	Environment	Depredators and (Pest) Predators	3	0
People Who Care (Existence)	Environment	Depredators and (Pest) Predators	3	0
Researchers	Environment	Depredators and (Pest) Predators	0	1
Residential Property Owners	Environment	Depredators and (Pest) Predators	2	0
Resource-Dependent Businesses	Environment	Depredators and (Pest) Predators	2	0
Agricultural Processors	Environment	Fauna	1	0
All Humans	Environment	Fauna	3	1
Artists	Environment	Fauna	0	3
Boaters	Environment	Fauna	3	5
Commercial_Military Transportation	Environment	Fauna	1	2
Commerical_Industrial	Environment	Fauna	6	8
Educators and Students	Environment	Fauna	13	25
Experiencers and Viewers	Environment	Fauna	15	23
Food Extractors	Environment	Fauna	5	6
Food Subsisters	Environment	Fauna	0	1
Fur_Hide Trappers and Hunters	Environment	Fauna	6	12
Government and Municipal and Residential	Environment	Fauna	19	26
Hunters	Environment	Fauna	19	29
Inspirational	Environment	Fauna	0	2
Learning	Environment	Fauna	10	19
Military_Coast Guard	Environment	Fauna	0	3
People Who Care (Existence)	Environment	Fauna	27	28
People Who Care (Option_Bequest)	Environment	Fauna	9	17
Recreational	Environment	Fauna	17	25
Researchers	Environment	Fauna	3	7
Residential Property Owners	Environment	Fauna	5	5
Resource-Dependent Businesses	Environment	Fauna	3	7
Spiritual and Ceremonial Participants and Participants of Celebration	Environment	Fauna	1	5
Subsistence	Environment	Fauna	0	2
Timber and Fiber and Ornamental Extractors	Environment	Fauna	1	3
Farmers	Environment	Fiber	2	0
Government and Municipal and Residential	Environment	Fiber	3	0

People Who Care (Existence)	Environment	Fiber	2	0
All Humans	Environment	Fish	3	0
Anglers	Environment	Fish	11	21
Aquaculturists	Environment	Fish	5	0
Boaters	Environment	Fish	0	1
Educators and Students	Environment	Fish	2	1
Experiencers and Viewers	Environment	Fish	2	1
Food Extractors	Environment	Fish	27	25
Food Pickers and Gatherers	Environment	Fish	4	4
Food Subsisters	Environment	Fish	4	4
Government and Municipal and Residential	Environment	Fish	9	4
Learning	Environment	Fish	4	0
People Who Care (Existence)	Environment	Fish	22	23
People Who Care (Option_Bequest)	Environment	Fish	4	0
Pharmaceutical and Food Supplement Suppliers	Environment	Fish	2	0
Recreational	Environment	Fish	6	10
Researchers	Environment	Fish	1	2
Spiritual and Ceremonial Participants and Participants of Celebration	Environment	Fish	0	1
Timber and Fiber and Ornamental Extractors	Environment	Fish	1	0
All Humans	Environment	Flora	4	0
CAFO Operators	Environment	Flora	0	1
Commercial_Military Transportation	Environment	Flora	1	2
Commerical_Industrial	Environment	Flora	3	5
Educators and Students	Environment	Flora	2	14
Experiencers and Viewers	Environment	Flora	2	15
Food Pickers and Gatherers	Environment	Flora	1	2
Government and Municipal and Residential	Environment	Flora	10	8
Industrial Processors	Environment	Flora	1	1
Learning	Environment	Flora	3	4
Military_Coast Guard	Environment	Flora	1	1
People Who Care (Existence)	Environment	Flora	28	28
People Who Care (Option_Bequest)	Environment	Flora	2	9
Pharmaceutical and Food Supplement Suppliers	Environment	Flora	0	1
Recreational	Environment	Flora	1	5
Researchers	Environment	Flora	0	3
Residential Property Owners	Environment	Flora	2	1
Resource-Dependent Businesses	Environment	Flora	4	4
Spiritual and Ceremonial Participants and Participants of Celebration	Environment	Flora	1	0
Timber and Fiber and Ornamental Extractors	Environment	Flora	1	1
Experiencers and Viewers	Environment	Fungi	0	1
Recreational	Environment	Fungi	0	1
Commercial_Military Transportation	Environment	Land	3	1
Commerical_Industrial	Environment	Land	4	0
Educators and Students	Environment	Land	1	1
Electric and other Energy Generators	Environment	Land	0	1
Farmers	Environment	Land	1	2
Government and Municipal and Residential	Environment	Land	21	25
Hunters	Environment	Land	0	1
Industrial Dischargers	Environment	Land	1	0
Irrigators	Environment	Land	1	0
Learning	Environment	Land	2	22
Military_Coast Guard	Environment	Land	0	2
People Who Care (Existence)	Environment	Land	23	26
People Who Care (Option_Bequest)	Environment	Land	2	12
Recreational	Environment	Land	2	1
Residential Property Owners	Environment	Land	5	5
Transporters of People	Environment	Land	3	0
Commercial_Military Transportation	Environment	Natural Materials	1	0
Commerical_Industrial	Environment	Natural Materials	1	0
Experiencers and Viewers	Environment	Natural Materials	0	5
Government and Municipal and Residential	Environment	Natural Materials	3	3
Hunters	Environment	Natural Materials	0	1
Industrial Processors	Environment	Natural Materials	13	8
Military_Coast Guard	Environment	Natural Materials	1	0
People Who Care (Existence)	Environment	Natural Materials	3	1
Timber and Fiber and Fur_Hide Subsisters	Environment	Natural Materials	1	7
Commercial_Military Transportation	Environment	Open Space	1	0
Commerical_Industrial	Environment	Open Space	1	1
Educators and Students	Environment	Open Space	0	1
Experiencers and Viewers	Environment	Open Space	0	1
Farmers	Environment	Open Space	2	1
Government and Municipal and Residential	Environment	Open Space	20	17
Military_Coast Guard	Environment	Open Space	1	0
People Who Care (Existence)	Environment	Open Space	8	2
People Who Care (Option_Bequest)	Environment	Open Space	1	1
Recreational	Environment	Open Space	3	2
Residential Property Owners	Environment	Open Space	2	1
Resource-Dependent Businesses	Environment	Open Space	1	1

Learning	Environment	Pollinators	0	2
Agricultural	Environment	Presence of the Environment	1	0
Agricultural Processors	Environment	Presence of the Environment	3	0
All Humans	Environment	Presence of the Environment	23	26
Anglers	Environment	Presence of the Environment	0	1
Aquaculturists	Environment	Presence of the Environment	3	2
Artists	Environment	Presence of the Environment	6	11
Boaters	Environment	Presence of the Environment	15	13
CAFO Operators	Environment	Presence of the Environment	7	1
Commercial_Military Transportation	Environment	Presence of the Environment	14	18
Commerical_Industrial	Environment	Presence of the Environment	27	28
Educators and Students	Environment	Presence of the Environment	28	29
Electric and other Energy Generators	Environment	Presence of the Environment	11	6
Experiencers and Viewers	Environment	Presence of the Environment	24	28
Farmers	Environment	Presence of the Environment	4	2
Food Extractors	Environment	Presence of the Environment	1	3
Food Pickers and Gatherers	Environment	Presence of the Environment	3	4
Food Subsisters	Environment	Presence of the Environment	1	2
Foresters	Environment	Presence of the Environment	0	1
Government and Municipal and Residential	Environment	Presence of the Environment	28	29
Hunters	Environment	Presence of the Environment	2	12
Industrial Dischargers	Environment	Presence of the Environment	8	3
Industrial Processors	Environment	Presence of the Environment	13	11
Inspirational	Environment	Presence of the Environment	10	18
Irrigators	Environment	Presence of the Environment	1	3
Learning	Environment	Presence of the Environment	28	29
Livestock Grazers	Environment	Presence of the Environment	1	2
Military_Coast Guard	Environment	Presence of the Environment	7	6
Municipal Drinking Water Plant Operators	Environment	Presence of the Environment	2	2
People Who Care (Existence)	Environment	Presence of the Environment	28	29
People Who Care (Option_Bequest)	Environment	Presence of the Environment	28	29
Recreational	Environment	Presence of the Environment	28	29
Researchers	Environment	Presence of the Environment	26	29
Residential Property Owners	Environment	Presence of the Environment	21	21
Resource-Dependent Businesses	Environment	Presence of the Environment	28	29
Spiritual and Ceremonial Participants and Participants of Celebration	Environment	Presence of the Environment	8	16
Subsistence	Environment	Presence of the Environment	2	3
Timber and Fiber and Fur_Hide Subsisters	Environment	Presence of the Environment	0	1
Timber and Fiber and Ornamental Extractors	Environment	Presence of the Environment	2	2
Transporters of People	Environment	Presence of the Environment	6	21
Wastewater Treatment Plant Operators	Environment	Presence of the Environment	4	3
Commerical_Industrial	Environment	Soil	1	0
People Who Care (Existence)	Environment	Soil	1	2
All Humans	Environment	Sounds and Scents	1	0
Experiencers and Viewers	Environment	Sounds and Scents	2	4
Recreational	Environment	Sounds and Scents	1	0
Resource-Dependent Businesses	Environment	Sounds and Scents	1	0
Educators and Students	Environment	Substrate	1	1
Food Pickers and Gatherers	Environment	Substrate	0	1
Government and Municipal and Residential	Environment	Substrate	0	1
People Who Care (Existence)	Environment	Substrate	1	0
Commercial_Military Transportation	Environment	Timber	0	1
Commerical_Industrial	Environment	Timber	3	5
Foresters	Environment	Timber	0	1
Government and Municipal and Residential	Environment	Timber	1	2
Industrial Processors	Environment	Timber	2	3
People Who Care (Option_Bequest)	Environment	Timber	0	1
Residential Property Owners	Environment	Timber	0	2
Resource-Dependent Businesses	Environment	Timber	0	1
Timber and Fiber and Ornamental Extractors	Environment	Timber	6	16
All Humans	Environment	Viewscapes	1	1
Artists	Environment	Viewscapes	0	1
Boaters	Environment	Viewscapes	1	0
Commercial_Military Transportation	Environment	Viewscapes	0	1
Experiencers and Viewers	Environment	Viewscapes	26	27
Government and Municipal and Residential	Environment	Viewscapes	4	8
Inspirational	Environment	Viewscapes	6	13
Learning	Environment	Viewscapes	0	4
People Who Care (Existence)	Environment	Viewscapes	2	4
People Who Care (Option_Bequest)	Environment	Viewscapes	2	3
Recreational	Environment	Viewscapes	5	8
Residential Property Owners	Environment	Viewscapes	2	1
Transporters of People	Environment	Viewscapes	0	1
All Humans	Environment	Water	4	1
Artists	Environment	Water	1	1
Boaters	Environment	Water	18	27
Commercial_Military Transportation	Environment	Water	4	4
Commerical_Industrial	Environment	Water	2	4

Educators and Students	Environment	Water	4	3
Electric and other Energy Generators	Environment	Water	8	1
Experiencers and Viewers	Environment	Water	3	4
Farmers	Environment	Water	1	1
Government and Municipal and Residential	Environment	Water	17	16
Industrial Dischargers	Environment	Water	9	2
Industrial Processors	Environment	Water	1	1
Irrigators	Environment	Water	15	5
Learning	Environment	Water	5	2
Municipal Drinking Water Plant Operators	Environment	Water	5	4
People Who Care (Existence)	Environment	Water	27	29
People Who Care (Option_Bequest)	Environment	Water	3	2
Recreational	Environment	Water	4	3
Researchers	Environment	Water	2	4
Residential Property Owners	Environment	Water	5	4
Resource-Dependent Businesses	Environment	Water	2	2
Transporters of Goods	Environment	Water	1	1
Waders and Swimmers and Divers	Environment	Water	14	16
Wastewater Treatment Plant Operators	Environment	Water	5	1
Water Subsisters	Environment	Water	4	2
Educators and Students	Environment	Weather	1	1
Experiencers and Viewers	Environment	Weather	0	1
Government and Municipal and Residential	Environment	Weather	3	2
Hunters	Environment	Weather	0	1
People Who Care (Existence)	Environment	Weather	1	1
People Who Care (Option_Bequest)	Environment	Weather	1	0
Recreational	Environment	Weather	4	5
Residential Property Owners	Environment	Weather	1	1
Resource-Dependent Businesses	Environment	Weather	0	1
Spiritual and Ceremonial Participants and Participants of Celebration	Environment	Weather	0	1
Boaters	Environment	Wind	1	1
Electric and other Energy Generators	Environment	Wind	2	3
Experiencers and Viewers	Environment	Wind	2	3
Unknown Beneficiary	Aquatic	Air	6	4
Unknown Beneficiary	Atmosphere	Air	20	16
Unknown Beneficiary	Estuaries and Near Coastal Marine	Air	6	2
Unknown Beneficiary	Forests	Air	0	1
Unknown Beneficiary	Terrestrial	Air	2	1
Unknown Beneficiary	Agroecosystems	Depredators and (Pest) Predators	3	0
Unknown Beneficiary	Aquatic	Depredators and (Pest) Predators	2	0
Unknown Beneficiary	Created Greenspace	Depredators and (Pest) Predators	3	0
Unknown Beneficiary	Estuaries and Near Coastal Marine	Depredators and (Pest) Predators	2	0
Unknown Beneficiary	Terrestrial	Depredators and (Pest) Predators	4	2
Unknown Beneficiary	Wetlands	Depredators and (Pest) Predators	0	1
Unknown Beneficiary	Aquatic	Fauna	20	24
Unknown Beneficiary	Barren_Rock and Sand	Fauna	3	2
Unknown Beneficiary	Created Greenspace	Fauna	4	4
Unknown Beneficiary	Estuaries and Near Coastal Marine	Fauna	27	29
Unknown Beneficiary	Forests	Fauna	5	10
Unknown Beneficiary	Lakes and Ponds	Fauna	2	2
Unknown Beneficiary	Open Oceans and Seas	Fauna	7	10
Unknown Beneficiary	Rivers and Streams	Fauna	13	10
Unknown Beneficiary	Scrublands_Shrublands	Fauna	1	2
Unknown Beneficiary	Terrestrial	Fauna	18	21
Unknown Beneficiary	Wetlands	Fauna	13	19
Unknown Beneficiary	Agroecosystems	Fiber	1	0
Unknown Beneficiary	Estuaries and Near Coastal Marine	Fiber	1	0
Unknown Beneficiary	Aquatic	Fish	28	28
Unknown Beneficiary	Barren_Rock and Sand	Fish	11	4
Unknown Beneficiary	Created Greenspace	Fish	1	1
Unknown Beneficiary	Estuaries and Near Coastal Marine	Fish	28	26
Unknown Beneficiary	Forests	Fish	2	3
Unknown Beneficiary	Lakes and Ponds	Fish	5	4
Unknown Beneficiary	Open Oceans and Seas	Fish	20	16
Unknown Beneficiary	Rivers and Streams	Fish	19	12
Unknown Beneficiary	Terrestrial	Fish	15	11
Unknown Beneficiary	Wetlands	Fish	16	20
Unknown Beneficiary	Aquatic	Flora	17	13
Unknown Beneficiary	Barren_Rock and Sand	Flora	4	3
Unknown Beneficiary	Created Greenspace	Flora	3	4
Unknown Beneficiary	Estuaries and Near Coastal Marine	Flora	27	29
Unknown Beneficiary	Forests	Flora	12	19
Unknown Beneficiary	Grasslands	Flora	2	3
Unknown Beneficiary	Lakes and Ponds	Flora	2	3
Unknown Beneficiary	Open Oceans and Seas	Flora	3	8
Unknown Beneficiary	Rivers and Streams	Flora	13	6
Unknown Beneficiary	Scrublands_Shrublands	Flora	4	10
Unknown Beneficiary	Terrestrial	Flora	24	27

Unknown Beneficiary	Wetlands	Flora	12	18
Unknown Beneficiary	Terrestrial	Fungi	2	1
Unknown Beneficiary	Wetlands	Fungi	1	0
Unknown Beneficiary	Aquatic	Land	17	17
Unknown Beneficiary	Barren_Rock and Sand	Land	2	5
Unknown Beneficiary	Created Greenspace	Land	2	6
Unknown Beneficiary	Estuaries and Near Coastal Marine	Land	28	27
Unknown Beneficiary	Forests	Land	8	13
Unknown Beneficiary	Grasslands	Land	0	4
Unknown Beneficiary	Lakes and Ponds	Land	5	3
Unknown Beneficiary	Rivers and Streams	Land	20	14
Unknown Beneficiary	Scrublands_Shrublands	Land	1	4
Unknown Beneficiary	Terrestrial	Land	27	29
Unknown Beneficiary	Wetlands	Land	20	25
Unknown Beneficiary	Aquatic	Natural Materials	9	2
Unknown Beneficiary	Barren_Rock and Sand	Natural Materials	10	8
Unknown Beneficiary	Estuaries and Near Coastal Marine	Natural Materials	12	5
Unknown Beneficiary	Lakes and Ponds	Natural Materials	1	1
Unknown Beneficiary	Open Oceans and Seas	Natural Materials	1	2
Unknown Beneficiary	Rivers and Streams	Natural Materials	4	2
Unknown Beneficiary	Terrestrial	Natural Materials	9	5
Unknown Beneficiary	Wetlands	Natural Materials	5	2
Unknown Beneficiary	Aquatic	Open Space	1	0
Unknown Beneficiary	Barren_Rock and Sand	Open Space	0	1
Unknown Beneficiary	Created Greenspace	Open Space	4	0
Unknown Beneficiary	Estuaries and Near Coastal Marine	Open Space	2	4
Unknown Beneficiary	Forests	Open Space	0	1
Unknown Beneficiary	Rivers and Streams	Open Space	1	2
Unknown Beneficiary	Terrestrial	Open Space	0	2
Unknown Beneficiary	Wetlands	Open Space	0	2
Unknown Beneficiary	Agroecosystems	Pollinators	1	0
Unknown Beneficiary	Created Greenspace	Pollinators	1	3
Unknown Beneficiary	Terrestrial	Pollinators	1	5
Unknown Beneficiary	Wetlands	Pollinators	0	1
Unknown Beneficiary	Agroecosystems	Presence of the Environment	4	5
Unknown Beneficiary	Aquatic	Presence of the Environment	28	29
Unknown Beneficiary	Atmosphere	Presence of the Environment	6	10
Unknown Beneficiary	Barren_Rock and Sand	Presence of the Environment	24	17
Unknown Beneficiary	Created Greenspace	Presence of the Environment	20	25
Unknown Beneficiary	Estuaries and Near Coastal Marine	Presence of the Environment	28	29
Unknown Beneficiary	Forests	Presence of the Environment	23	29
Unknown Beneficiary	Grasslands	Presence of the Environment	5	11
Unknown Beneficiary	Groundwater	Presence of the Environment	21	18
Unknown Beneficiary	Ice and Snow	Presence of the Environment	6	3
Unknown Beneficiary	Lakes and Ponds	Presence of the Environment	18	16
Unknown Beneficiary	Open Oceans and Seas	Presence of the Environment	27	29
Unknown Beneficiary	Rivers and Streams	Presence of the Environment	28	29
Unknown Beneficiary	Scrublands_Shrublands	Presence of the Environment	15	19
Unknown Beneficiary	Terrestrial	Presence of the Environment	28	29
Unknown Beneficiary	Wetlands	Presence of the Environment	28	29
Unknown Beneficiary	Aquatic	Soil	4	1
Unknown Beneficiary	Estuaries and Near Coastal Marine	Soil	4	4
Unknown Beneficiary	Forests	Soil	1	6
Unknown Beneficiary	Open Oceans and Seas	Soil	0	1
Unknown Beneficiary	Rivers and Streams	Soil	0	4
Unknown Beneficiary	Scrublands_Shrublands	Soil	1	6
Unknown Beneficiary	Terrestrial	Soil	11	13
Unknown Beneficiary	Tundra	Soil	0	1
Unknown Beneficiary	Wetlands	Soil	1	5
Unknown Beneficiary	Aquatic	Sounds and Scents	1	0
Unknown Beneficiary	Barren_Rock and Sand	Sounds and Scents	1	0
Unknown Beneficiary	Aquatic	Substrate	4	6
Unknown Beneficiary	Barren_Rock and Sand	Substrate	12	12
Unknown Beneficiary	Estuaries and Near Coastal Marine	Substrate	19	15
Unknown Beneficiary	Forests	Substrate	0	3
Unknown Beneficiary	Lakes and Ponds	Substrate	1	3
Unknown Beneficiary	Open Oceans and Seas	Substrate	0	10
Unknown Beneficiary	Rivers and Streams	Substrate	1	4
Unknown Beneficiary	Scrublands_Shrublands	Substrate	1	4
Unknown Beneficiary	Terrestrial	Substrate	4	5
Unknown Beneficiary	Wetlands	Substrate	5	7
Unknown Beneficiary	Forests	Timber	4	6
Unknown Beneficiary	Terrestrial	Timber	0	4
Unknown Beneficiary	Agroecosystems	Viewscapes	1	0
Unknown Beneficiary	Aquatic	Viewscapes	7	2
Unknown Beneficiary	Barren_Rock and Sand	Viewscapes	1	2
Unknown Beneficiary	Estuaries and Near Coastal Marine	Viewscapes	12	6
Unknown Beneficiary	Forests	Viewscapes	1	3

Unknown Beneficiary	Lakes and Ponds	Viewscapes	2	0
Unknown Beneficiary	Open Oceans and Seas	Viewscapes	1	1
Unknown Beneficiary	Rivers and Streams	Viewscapes	2	5
Unknown Beneficiary	Terrestrial	Viewscapes	4	6
Unknown Beneficiary	Wetlands	Viewscapes	3	2
Unknown Beneficiary	Agroecosystems	Water	2	0
Unknown Beneficiary	Aquatic	Water	28	29
Unknown Beneficiary	Barren_Rock and Sand	Water	7	5
Unknown Beneficiary	Created Greenspace	Water	8	6
Unknown Beneficiary	Estuaries and Near Coastal Marine	Water	28	29
Unknown Beneficiary	Forests	Water	7	3
Unknown Beneficiary	Groundwater	Water	13	9
Unknown Beneficiary	Ice and Snow	Water	2	2
Unknown Beneficiary	Lakes and Ponds	Water	14	6
Unknown Beneficiary	Open Oceans and Seas	Water	23	18
Unknown Beneficiary	Rivers and Streams	Water	27	26
Unknown Beneficiary	Scrublands_Shrublands	Water	2	0
Unknown Beneficiary	Terrestrial	Water	24	26
Unknown Beneficiary	Wetlands	Water	20	16
Unknown Beneficiary	Aquatic	Weather	5	4
Unknown Beneficiary	Estuaries and Near Coastal Marine	Weather	9	11
Unknown Beneficiary	Forests	Weather	1	0
Unknown Beneficiary	Ice and Snow	Weather	0	1
Unknown Beneficiary	Lakes and Ponds	Weather	1	1
Unknown Beneficiary	Open Oceans and Seas	Weather	1	7
Unknown Beneficiary	Rivers and Streams	Weather	7	1
Unknown Beneficiary	Scrublands_Shrublands	Weather	0	3
Unknown Beneficiary	Terrestrial	Weather	2	8
Unknown Beneficiary	Wetlands	Weather	2	4
Agricultural	Environment	FEGS	1	0
Agricultural Processors	Environment	FEGS	4	0
All Humans	Environment	FEGS	20	15
Aquaculturists	Environment	FEGS	1	0
Artists	Environment	FEGS	0	2
Boaters	Environment	FEGS	2	1
CAFO Operators	Environment	FEGS	6	1
Commercial_Military Transportation	Environment	FEGS	7	7
Commerical_Industrial	Environment	FEGS	14	14
Educators and Students	Environment	FEGS	19	29
Electric and other Energy Generators	Environment	FEGS	3	5
Experiencers and Viewers	Environment	FEGS	8	21
Food Extractors	Environment	FEGS	3	4
Food Subsisters	Environment	FEGS	2	5
Government and Municipal and Residential	Environment	FEGS	27	29
Hunters	Environment	FEGS	0	3
Industrial Dischargers	Environment	FEGS	1	0
Industrial Processors	Environment	FEGS	3	1
Inspirational	Environment	FEGS	22	29
Irrigators	Environment	FEGS	1	0
Learning	Environment	FEGS	27	29
Livestock Grazers	Environment	FEGS	0	1
Military_Coast Guard	Environment	FEGS	3	0
Non-Use	Environment	FEGS	1	0
People Who Care (Existence)	Environment	FEGS	28	29
People Who Care (Option_Bequest)	Environment	FEGS	23	29
Pharmaceutical and Food Supplement Suppliers	Environment	FEGS	1	0
Recreational	Environment	FEGS	21	29
Researchers	Environment	FEGS	15	28
Residential Property Owners	Environment	FEGS	21	17
Resource-Dependent Businesses	Environment	FEGS	16	19
Subsistence	Environment	FEGS	8	20
Transporters of Goods	Environment	FEGS	2	0
Transporters of People	Environment	FEGS	1	2
Unknown Beneficiary	Agroecosystems	FEGS	15	9
Unknown Beneficiary	Aquatic	FEGS	28	29
Unknown Beneficiary	Barren_Rock and Sand	FEGS	10	5
Unknown Beneficiary	Created Greenspace	FEGS	2	10
Unknown Beneficiary	Estuaries and Near Coastal Marine	FEGS	28	29
Unknown Beneficiary	Forests	FEGS	12	20
Unknown Beneficiary	Grasslands	FEGS	1	3
Unknown Beneficiary	Groundwater	FEGS	8	3
Unknown Beneficiary	Lakes and Ponds	FEGS	5	8
Unknown Beneficiary	Open Oceans and Seas	FEGS	27	29
Unknown Beneficiary	Rivers and Streams	FEGS	24	21
Unknown Beneficiary	Scrublands_Shrublands	FEGS	4	6
Unknown Beneficiary	Terrestrial	FEGS	28	29
Unknown Beneficiary	Wetlands	FEGS	28	29
Unknown Beneficiary	Environment	Air	4	2

Unknown Beneficiary	Environment	Depredators and (Pest) Predators	11	3
Unknown Beneficiary	Environment	Fauna	26	29
Unknown Beneficiary	Environment	Fiber	1	1
Unknown Beneficiary	Environment	Fish	26	27
Unknown Beneficiary	Environment	Flora	22	23
Unknown Beneficiary	Environment	Land	27	28
Unknown Beneficiary	Environment	Natural Materials	14	5
Unknown Beneficiary	Environment	Open Space	3	1
Unknown Beneficiary	Environment	Presence of the Environment	28	29
Unknown Beneficiary	Environment	Soil	3	4
Unknown Beneficiary	Environment	Sounds and Scents	1	0
Unknown Beneficiary	Environment	Substrate	7	7
Unknown Beneficiary	Environment	Timber	1	1
Unknown Beneficiary	Environment	Viewscapes	7	11
Unknown Beneficiary	Environment	Water	27	29
Unknown Beneficiary	Environment	Weather	7	15
Unknown Beneficiary	Environment	FEGS	28	29
